# Commercial fishing patterns influence odontocete whale-longline interactions in the Southern Ocean

**DOI:** 10.1038/s41598-018-36389-x

**Published:** 2019-02-13

**Authors:** Paul Tixier, Paul Burch, Gaetan Richard, Karin Olsson, Dirk Welsford, Mary-Anne Lea, Mark A. Hindell, Christophe Guinet, Anais Janc, Nicolas Gasco, Guy Duhamel, Maria Ching Villanueva, Lavinia Suberg, Rhys Arangio, Marta Söffker, John P. Y. Arnould

**Affiliations:** 1 School of Life and Environmental Sciences (Burwood campus), Deakin University, Geelong, Victoria Australia; 2 Oceans and Atmosphere, Commonwealth Scientific and Industrial Research Organisation (CSIRO), Hobart, Tasmania Australia; 3 Centre d’Etudes Biologiques de Chizé (CEBC), UMR 7372 Université de la Rochelle-CNRS, Villiers-en-Bois, France; 4 Centre for Environment, Fisheries & Aquaculture Science, Lowestoft, United Kingdom; 5 Department of Zoology, Tel Aviv University and Intra-University Institute, Eilat, Israel; 6 Department of the Environment, Australian Antarctic Division, Kingston, Tasmania Australia; 7 Ecology and Biodiversity Centre, Institute for Marine and Antarctic Studies, University of Tasmania, Hobart, Tasmania Australia; 8 Département Adaptations du vivant, UMR BOREA, Museum National d’Histoire Naturelle, Paris, France; 9 Laboratoire de Biologie Halieutique (STH-LBH), IFREMER, ZI de la Pointe du Diable, BP 70, 29280 Plouzané, France; 10 Coalition of Legal Toothfish Operators (COLTO), Perth, Australia

## Abstract

The emergence of longline fishing around the world has been concomitant with an increase in depredation-interactions by odontocete whales (removal of fish caught on hooks), resulting in substantial socio-economic and ecological impacts. The extent, trends and underlying mechanisms driving these interactions remain poorly known. Using long-term (2003–2017) datasets from seven major Patagonian toothfish (*Dissostichus eleginoides*) longline fisheries, this study assessed the levels and inter-annual trends of sperm whale (*Physeter macrocephalus*) and/or killer whale (*Orcinus orca*) interactions as proportions of fishing time (days) and fishing area (spatial cells). The role of fishing patterns in explaining between-fisheries variations of probabilities of odontocete interactions was investigated. While interaction levels remained globally stable since the early 2000s, they varied greatly between fisheries from 0 to >50% of the fishing days and area. Interaction probabilities were influenced by the seasonal concentration of fishing effort, size of fishing areas, density of vessels, their mobility and the depth at which they operated. The results suggest that between-fisheries variations of interaction probabilities are largely explained by the extent to which vessels provide whales with opportunities for interactions. Determining the natural distribution of whales will, therefore, allow fishers to implement better strategies of spatio-temporal avoidance of depredation.

## Introduction

Over the last 60 years, the world’s commercial fisheries have undergone substantial changes in distribution, intensity, regulations and technology^1^. Fishing techniques have evolved towards greater efficiency but declines in catch per unit effort, paired with environmental impacts, have led some fisheries to increase target selectivity in their technological development. A number of trawling and gillnetting fisheries have progressively switched to longlining as a more selective fishing technique^2–4^. However, the emergence of longline fishing throughout the world oceans is concomitant with increasing reports of depredation interactions by marine top-predators, primarily odontocete (toothed) whales^5–10^, with fishing vessels.

Depredation interactions, hereafter termed interactions, are a form of human-wildlife conflict that occurs when wild species consume a resource caught or raised/grown by humans. Here, odontocetes directly remove fish from hooks on longlines, which results in a combination of socio-economic and conservation impacts. Socio-economic impacts include financial losses and increased fishing time for humans. Conservation impacts for the depredated fish include inaccurate stock assessments due to difficulties in estimating the amount of fish taken by odontocetes. For the depredating species, conservation impacts include negative effects due to increased risks of injury caused by fishing gear or lethal responses from fishers, increased dependency to depredation and alteration of natural energy intake balances, and positive effects from artificial food provisioning^8,10–20^.

While odontocete interactions have been increasingly reported over the past decade, it is unclear whether the issue is actually increasing in frequency and intensity^10^. In addition, the mechanisms leading whales to change from natural foraging behaviours to depredation are poorly understood. This change may be driven by two processes, occurring either separately or together. Firstly, depredation may be a purely opportunistic behaviour simply resulting from the spatio-temporal overlap of fishing operations with the natural distribution of whales and their normal prey. Secondly, depredation may be an active behaviour occurring when whales modify their natural distribution by actively searching for fishing vessels or by following them over great distances^21^.

Whether interactions result from opportunistic or active behaviour, their occurrence may be highly dependent upon the extent to which fishing vessels provide odontocetes with opportunities to depredate, and therefore the spatio-temporal patterns of fishing operations. The present study used this hypothesis to investigate the influence of fishing patterns of different commercial fisheries in the Southern Ocean on the levels of interaction between fishing vessels and two odontocete species: killer whales (*Orcinus orca*); and sperm whales (*Physeter macrocephalus*). These commercial fisheries operating in the waters of southern Chile, and around the Falklands, South Georgia, Prince Edward and Marion islands (hereafter “PEMI”), Crozet islands, Kerguelen islands, and Heard and MacDonald islands (hereafter “HIMI”) all use demersal longlines to catch Patagonian toothfish (*Dissostichus eleginoides*). Patagonian toothfish longline fisheries emerged as commercial fisheries in the 1980s-2000s, replacing existing bottom-trawling fisheries, and have all been subject to killer and/or sperm whale depredation interactions since the first years following their commencement^22–29^. These fisheries have now become the primary economic activity of Southern Ocean^30,31^ but greatly vary in size of fleets and fishing area, length of fishing seasons, quotas and longline fishing system. For instance, fisheries operating in Chile, the Falklands and PEMI predominantly use the trotline system (longlines with clusters of hooks) equipped with “cachalotera”, a fish protection device developed to reduce odontocete depredation and seabird mortality^32^, whereas the other fisheries use the autoline system (weighted longlines with individual hooks to reduce seabird mortality). Most fisheries also experienced substantial Illegal Unreported and Unregulated (IUU) fishing in the 1980s and 1990s, resulting in an over-harvest of local fish stocks and impacts on seabird and whale populations interacting with illegal vessels^30,33–39^.

Depredation by killer whales and sperm whales represent a major challenge for the economic viability of the toothfish fisheries, for the assessment of fish stocks and their management, and for the conservation of whale populations in the Southern Ocean^29^. Determining the role of fishing patterns in explaining variations in the level of whale interaction with vessels would bring important insights for fisheries to minimize depredation by adjusting their spacio-temporal fishing patterns. Therefore, the aims of this study were to: i) assess the level and annual trends of whale-fishing vessel interaction, both locally and globally in the Southern Ocean; and ii) examine the effect of variations in spatio-temporal fishing patterns on observed interaction levels.

## Results

### Spatial and temporal variations in interaction levels

**Figure 1:**
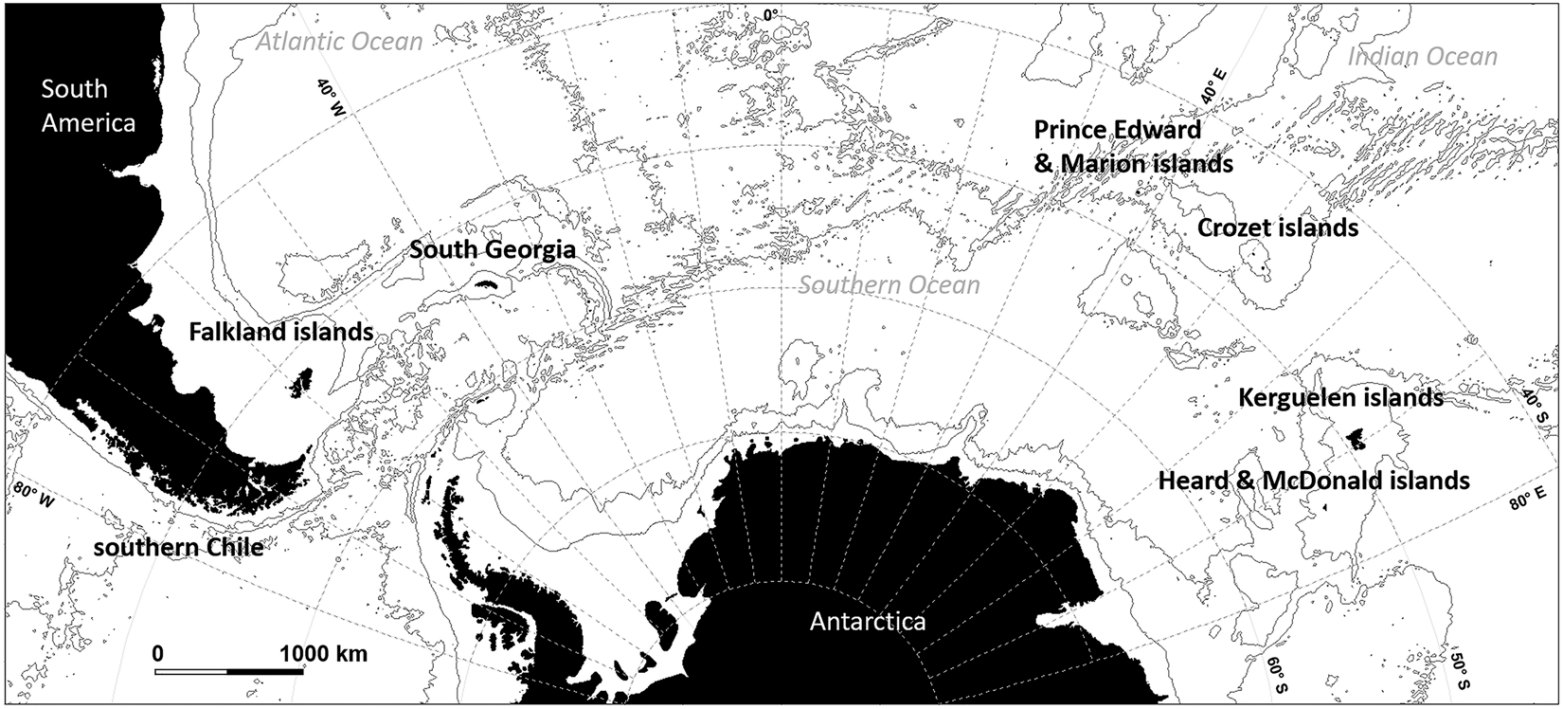
Location of areas where the seven commercial Patagonian toothfish demersal fisheries used in the study operate in the Southern Ocean.

**Figure 2:**
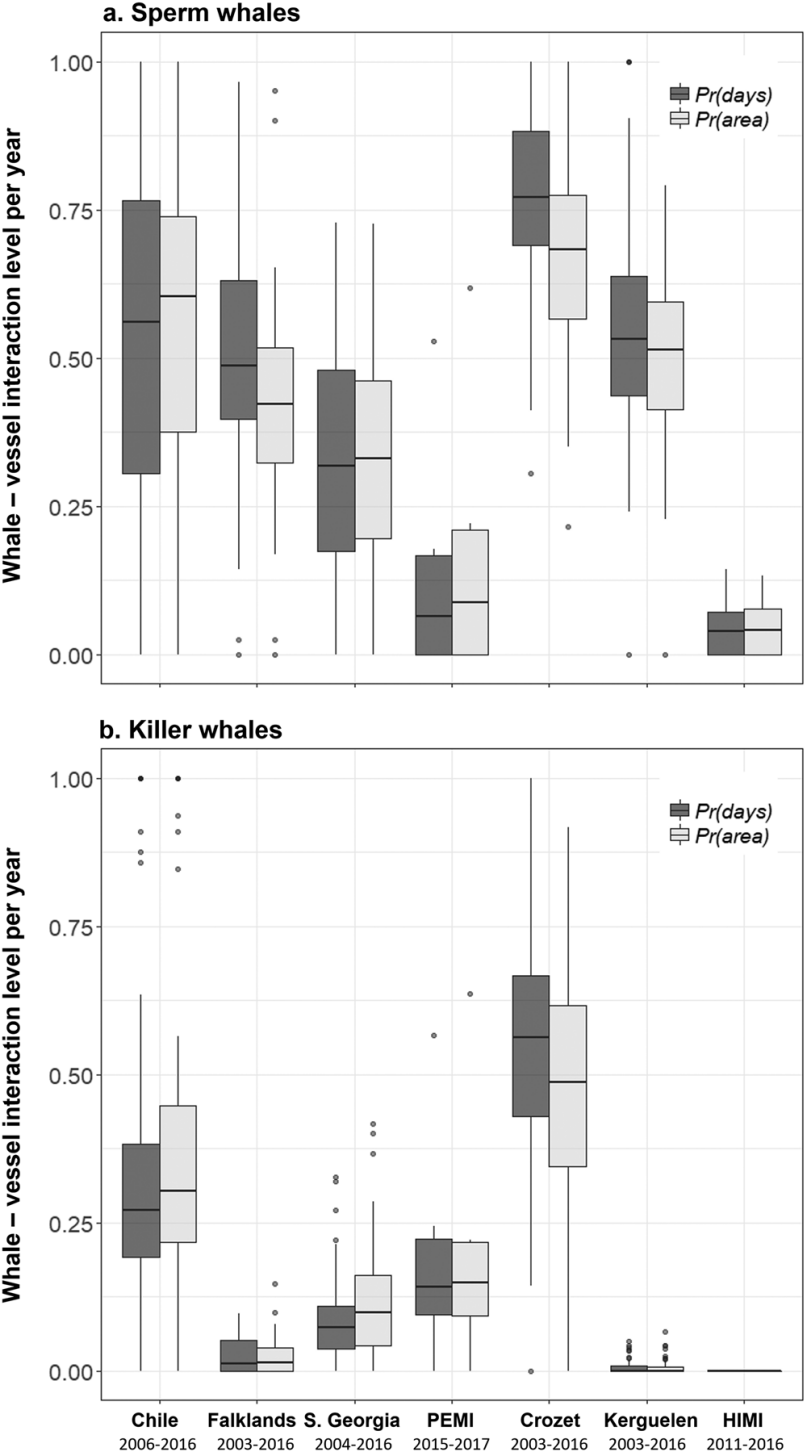
Between-fisheries variations of whale-vessel interaction levels in the Southern Ocean. Boxplots were calculated from *Pr(days)* and *Pr(area)* observed values per vessel per year in fisheries for (**a**) sperm whales and (**b**) killer whales.

Data from a total of 97,688 longline sets hauled in the seven study areas/fisheries (southern Chile, the Falklands, South Georgia, PEMI, Crozet, Kerguelen, HIMI, Fig. 1), were available for this study. Confirmed depredation interactions by killer whales occurred during hauling of 8,271 sets (8.5%) and 30,875 sets (31.6%) for sperm whales. The mean level of interactions per vessel per year varied between the seven fisheries for both sperm whales and killer whales. *Pr(days)* and *Pr(area)* were the highest for vessels that operated in Crozet, for both sperm whales (0.77 ± 0.02 of fishing days, 0.68 ± 0.02 of the fishing area with depredation, n = 96 vessels per year, Fig. 2a) and killer whales (0.55 ± 0.02 of fishing days, 0.49 ± 0.02 of the fishing area with interactions per vessel per year, n = 96 vessels per year, Fig. 2b). HIMI was the only fishery where killer whale interactions were never recorded. Vessels that operated in HIMI also had the lowest mean *Pr(days)* and *Pr(area)* for sperm whales (0.04 ± 0.01 of fishing days, 0.05 ± 0.01 of the fishing area, n = 20 vessels per year, Fig. 2).

**Figure 3:**
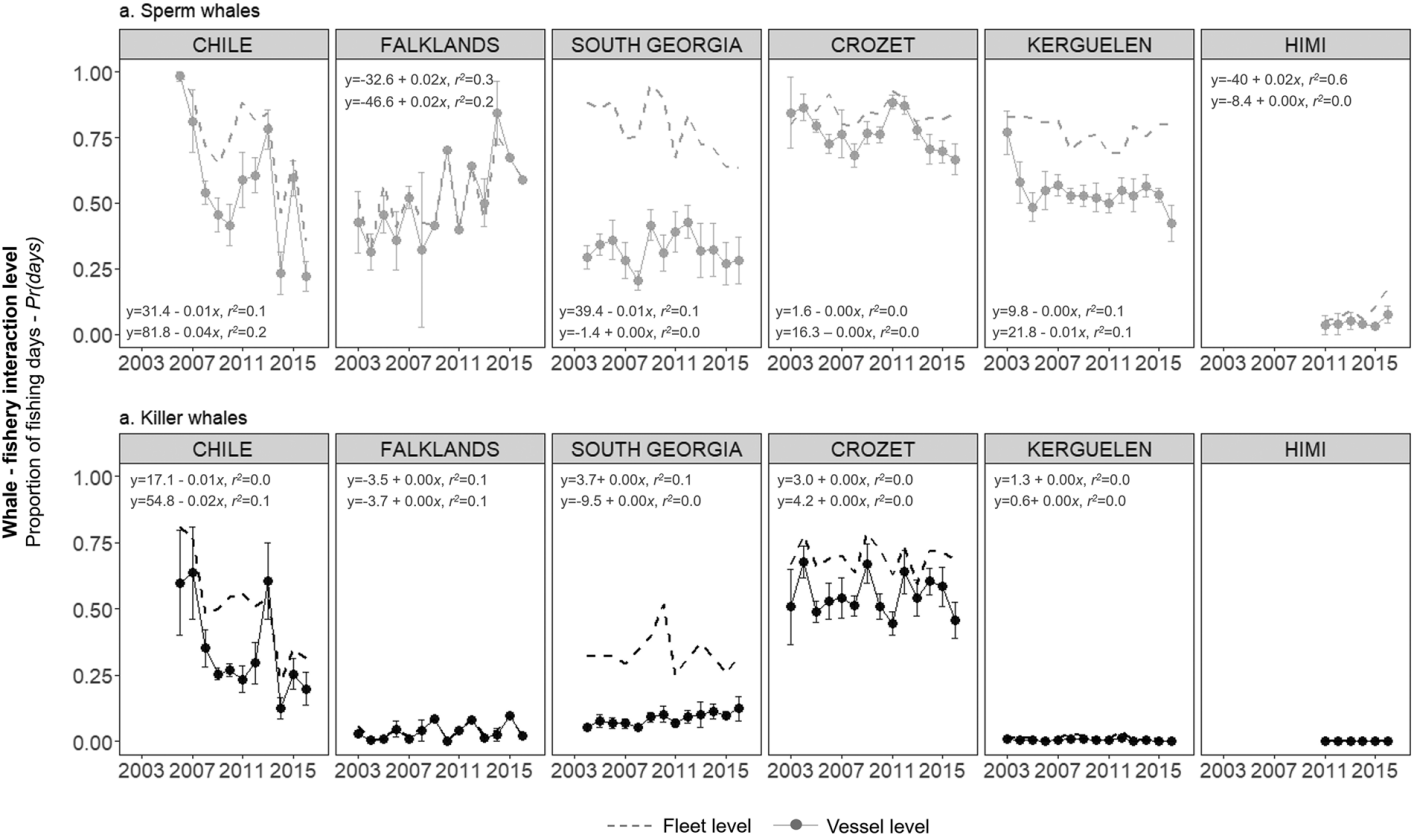
Observed annual variations of (**a**) sperm whale and (**b**) killer whale interaction levels with fisheries. Interaction levels (*Pr(days)*) were calculated as a proportion of fishing days during which at least one interaction was recorded out or all fishing days in a year, at the vessel level (mean ± SE per vessel per year, points and solid lines) and a the fleet level (dashed lines). Equations and *r*^2^ values of the linear regressions conducted at the fleet level (upper line) and at the vessel level (lower line) are also provided for each plot.

At the vessel level, significant decreases of *Pr(days)* over time were detected in Chile, Crozet and Kerguelen for sperm whales (t = −3.51, P < 0.01*;* t = −2.07, P = 0.04; t = −2.79, P < 0.01 for the three fisheries, respectively, Table S1a; Fig. 3a). However, *Pr(days)* for sperm whales significantly increased in the Falklands (t = 2.70, P = 0.01), with 0.43 ± 0.12 of the fishing days per vessel in 2003 (n = 6 vessels) to 0.59 (n = 1 vessel) in 2016 (Fig. 3a). *Pr(days)* for killer whales decreased significantly in Chile (t = −2.31, P = 0.02) but increased in South Georgia (t = 2.88, P < 0.01, Table S1b; Fig. 3b). In Chile, *Pr(days)* varied from 0.98 ± 0.02 of the fishing days per vessel with sperm whale interactions in 2006 (n = 4 vessels) to 0.22 ± 0.06 (n = 5 vessels) in 2016 (Fig. 3a), and from 0.60 ± 0.19 in 2006 (n = 4 vessels) to 0.20 ± 0.06 in 2016 (n = 5 vessels) for killer whales (Fig. 3b). At the fleet level, *Pr(days)* decreased in South Georgia (t = −3.23, P < 0.05) and increased in HIMI (t = 2.88, P = 0.04) for sperm whales (Table S1a; Fig. 3a). In HIMI, *Pr(days)* varied from 0.05 of the fishing days in 2011 to 0.17 in 2016 (Fig. 3a). No trend in *Pr(days)* was detected at the fleet level for killer whales. No general trends were detected at either the vessel or the fleet level when using data from all fisheries combined (Table S1a,b).

**Figure 4:**
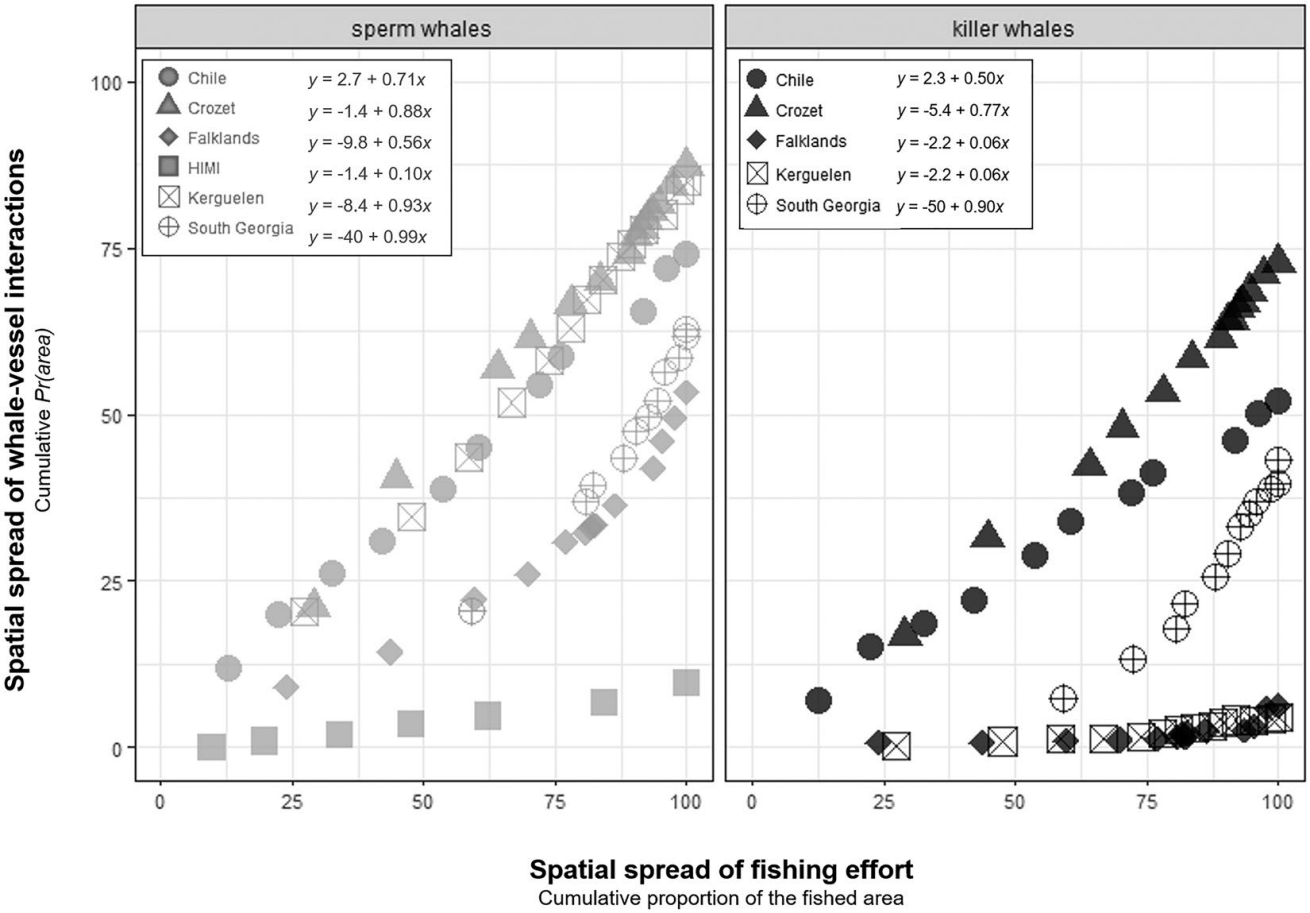
Relationship between the spatial spread of fishing effort and the spatial spread of whale-vessel interactions in fisheries (*Pr(area)*). The spatial spread of fishing effort and whale vessel interactions were calculated as the cumulative proportion of 0.1° × 0.1° cells over the full fished area in which fishing occurred and interactions were recorded, respectively, per year per fishery for sperm whales (grey) and killer whales (black). Linear regression equations are shown for each species and fishery.

Fisheries could be categorized into two groups based on the slope (${\hat{\beta }}_{1}$) of the linear correlation between the spatial spread of fishing operations and the cumulative proportion of the full fishing area where interactions occurred during the study (Fig. 4). The spatial spread of sperm whale interactions increased at a rate of ${\hat{\beta }}_{1} > 0.5$ with the spatial spread of fishing operation in all fisheries but HIMI (${\hat{\beta }}_{1}=0.1$). For killer whales, the spatial spread of interactions correlated with that of fishing operations at a rate of ${\hat{\beta }}_{1} > 0.5$ in Chile, South Georgia and Crozet and at a rate ${\hat{\beta }}_{1} < 0.5$ in the Falklands and Kerguelen (Fig. 4).

### Influence of fishing patterns on interaction probabilities

Models best fitting the level of sperm whale interactions, at both the vessel and the fleet levels, included all predictors including the interaction between fishery and mobility (Data S1, Table 1, Table S2.1 & S2.2). These models indicated that *Pr(days)* of sperm whales decreased with increasing size of the fishing area, proportion of effort in winter and depth of sets, and decreasing mobility of vessels and proportion of sets using trotlines equipped with cachalotera (Table 1). The density of vessels in fisheries decreased *Pr(days)* at the vessel level (GLM P = 0.04) but increased *Pr(days)* at the fleet level (GLM P < 0.01, Table 1).

**Table 1:** Parameter estimates for the optimal GLMs fitted to yearly sperm whale interaction levels with fishing vessels in fisheries through index *Pr(days)* at both the vessel and fleet level.

Predictors	*Vessel level*			*Fleet level*		
	Est. [95% CI]	z	P	Est. [95% CI]	z	P
**Fishery**						
*Chile*	0.59 [0.55–0.63]	3.99	<0.01	0.99 [0.98–0.99]	12.57	<0.01
*Crozet*	0.66 [0.61–0.71]	2.56	0.01	0.78 [0.61–0.89]	−7.74	<0.01
*Falklands*	0.39 [0.35–0.43]	−9.35	<0.01	0.76 [0.59–0.87]	−8.85	<0.01
*HIMI*	0.06 [0.05–0.08]	−19.77	<0.01	0.12 [0.06–0.24]	−16.30	<0.01
*Kerguelen*	0.67 [0.61–0.71]	2.75	0.01	0.82 [0.71–0.90]	−8.85	<0.01
*PEMI*	0.04 [0.03–0.07]	−13.36	<0.01	0.14 [0–0.85]	−3.54	<0.01
*South Georgia*	0.36 [0.31–0.42]	−7.79	<0.01	0.63 [0.41–0.80]	−9.11	<0.01
Total size of fishing area	0.48 [0.46–0.50]	−11.50	<0.01	0.99 [0.98–0.99]	−2.65	0.01
Density of vessels	0.57 [0.56–0.59]	−2.09	0.04	0.99 [0.99–0.99]	3.75	<0.01
Mobility of vessels	0.70 [0.67–0.72]	8.19	<0.01	1.00 [1.00–1.00]	9.85	<0.01
Depth	0.58 [0.57–0.59]	−2.13	0.03	0.99 [0.99–0.99]	−5.29	<0.01
Proportion of effort in winter	0.56 [0.54–0.57]	−5.34	<0.01	0.99[0.99–0.99]	−3.30	<0.01
Proportion of effort using trotlines and cachalotera	0.62 [0.61–0.62]	5.52	0.00	0.99 [0.99–0.99]	1.95	0.05
Fishery* Mobility of vessels						
*Crozet*	0.49 [0.45–0.53]	−5.08	<0.01	0.74 [0.55–0.81]	−8.18	<0.01
*Falklands*	0.50 [0.46–0.55]	−4.08	<0.01	0.89 [0.79–0.94]	−6.38	<0.01
*HIMI*	0.57 [0.46–0.66]	−0.48	0.63	0.82 [0.70–0.90]	−8.38	<0.01
*Kerguelen*	0.49 [0.46–0.52]	−6.10	<0.01	0.75 [0.60–0.86]	−9.57	<0.01
*PEMI*	0.30 [0.20–0.42]	−4.36	<0.01	0.77 [0.14–0.99]	−2.16	0.03
*South Georgia*	0.49 [0.45–0.53]	−5.16	<0.01	0.84 [0.71–0.92]	−7.13	<0.01

Parameter estimates are presented as probabilities relative to the Chilean fishery which was the default fishery in all models. Covariates with interaction probabilities higher than the Chilean fishery are associated with an increase in whale interactions while those with lower with interaction probabilities are associated with a reduction in whale interactions.

For killer whales at the vessel level, *Pr(days)* was best explained by the model including the fishery, the size of fishing areas, the density of vessels, their mobility, the depth of sets and an interaction between fishery and mobility (Data S2, Table 2, Table S2.3). The model without the interaction term was within 2 AIC of the optimal model (Table S2.3), however, trends in the common covariates were identical to the optimal model so it is not further discussed. At the vessel level, *Pr(days)* decreased with increasing size of fishing areas (GLM P < 0.01), increasing mobility of vessels (GLM P < 0.01) and increasing density of vessels (GLM P < 0.01, Table 2). At the fleet level for *Pr(days)* six models were within 2 AIC of the optimal model (Data S2, Table S2.4) which included the fishery, the density of vessels and the depth of sets and mobility (Data S2, Table 2, Table S2.4). All models within 2 AIC of the optimal model included fishery and the depth of sets, while the density of vessels was significant in five of the six models. Mobility of vessels was included in one model other than the optimal model, while the size of the fishing area, proportion of effort in winter and proportion of cachalotera errors were significant in one model each. For all models at the vessel level within 2 AIC of the optimal model, *Pr(days)* increased with the density and mobility of vessels and decreased with the depth of sets (Data S2, Table 2, Table S2.4).

**Table 2:** Parameter estimates for the optimal GLMs fitted to yearly killer whale interaction levels with fishing vessels in fisheries through index *Pr(days)* at both the vessel and fleet level.

Predictors	*Vessel level*			*Fleet level*		
	Est. [95% CI]	z	P	Est. [95% CI]	z	P
**Fishery**						
*Chile*	0.31 [0.28–0.35]	−9.37	<0.01	0.66 [0.61–0.70]	6.37	<0.01
*Crozet*	0.37 [0.32–0.42]	2.15	0.03	0.63 [0.56–0.70]	−0.78	0.44
*Falklands*	0.02 [0.01–0.02]	−23.25	<0.01	0.05 [0.04–0.06]	−25.23	<0.01
*HIMI*	0.00 [0.00–1.00]	−0.04	0.97	0.00 [0.00–1.00]	−0.02	0.98
*Kerguelen*	0.01 [0.01–0.02]	−16.46	<0.01	0.01 [0.01–0.02]	−24.98	<0.01
*PEMI*	0.09 [0.07–0.12]	−8.54	<0.01	0.23 [0.18–0.29]	−12.03	<0.01
*South Georgia*	0.08 [0.07–0.10]	−14.19	<0.01	0.28 [0.23–0.33]	−11.51	<0.01
Total size of fishing area	0.17 [0.15–0.19]	−11.43	<0.01	NS		
Density of vessels	0.26 [0.24–0.27]	−6.78	<0.01	0.68 [0.66–0.70]	2.51	0.01
Mobility of vessels	0.28 [0.26–0.30]	−2.70	0.01	NS		
**Depth**	0.26 [0.25–0.27]	−8.51	<0.01	0.58 [0.56–0.61]	−5.79	<0.01
**Proportion of effort in winter**						
Proportion of effort using trotlines and cachalotera	NS			NS		
Fishery * Mobility of vessels						
*Crozet*	0.33 [0.29–0.36]	0.72	0.47	NS		
*Falklands*	0.42 [0.35–0.49]	2.90	<0.01			
*HIMI*	0.36 [0.00–1.00]	0.00	1.00			
*Kerguelen*	0.40 [0.31–0.51]	1.79	0.07			
*PEMI*	0.36 [0.27–0.45]	0.94	0.35			
*South Georgia*	0.36 [0.32–0.41]	2.18	0.03			

Parameter estimates are presented as probabilities relative to the Chilean fishery which was the default fishery in all models. Covariates with interaction probabilities higher than the Chilean fishery are associated with an increase in whale interactions while those with lower with interaction probabilities are associated with a reduction in whale interactions.

## Discussion

The present study demonstrated large variations in the level of killer whale and sperm whale interactions with Patagonian toothfish fishing vessels between commercial fisheries in the Southern Ocean, and indicated that some of this variation can be explained by the fishing patterns of vessels. These findings suggest that the level of whale-fisheries interactions may primarily depend upon the extent to which fisheries provide whales with opportunities to depredate in space and time. However, the present study also demonstrated that some of the variability around whale-vessel interaction levels was attributed to as yet unknown area-specific factors that are further discussed here as potential directions for future research on odontocete depredation in the Southern Ocean.

Over the last 14 years, Patagonian toothfish fisheries operating in Chile, the Falklands, South Georgia, PEMI, Crozet, Kerguelen and HIMI all experienced sperm whale interactions, and six of them experienced killer whale interactions. In most fisheries, the level of interaction has remained stable. Interactions, which were reported as soon as demersal longlining started in the Southern Ocean in the 1980s and 1990s, are now an established behaviour for the majority of local whale populations. The exception is sperm whale interactions at HIMI which were first reported in 2010 despite longlining for Patagonian toothfish commencing in 2003^40^.

Increased fishing effort by vessels in winter coincided with decreased sperm whale interaction levels. This decrease is likely explained by seasonal shifts in the local abundance of mature male sperm whales, possibly driven by ecological and/or reproduction factors, with smaller numbers of individuals found at high latitudes in winter months^41–45^. As a result, lower densities of sperm whales in winter months may contribute to interaction levels being the lowest with vessels at HIMI and South Georgia, which are both primarily winter fisheries. As the fishing season at HIMI has extended in recent years, vessels concentrating increasing proportions of their effort in spring may also explain the emergence of sperm whale interactions in this fishery since 2010^40^.

Larger fishing areas were associated with decreased levels of both killer and sperm whales interaction with vessels. A larger fishing area is likely to decrease the probability of vessels being detected by whales^46–48^, to decrease the predictability of the fishing activity^29,49^ and to increase the effectiveness of move-on strategies which have been implemented to avoid/escape depredation^27,49,50^. However, these effects may be also driven by the density of vessels operating simultaneously in fishing areas. At the fleet level, greater densities of vessels were associated with higher interaction levels (present study). Increased number of vessels combined with a small fishing area may increase the detectability of fleets as a whole. This combination is, therefore, likely to contribute to the high interaction levels observed at Crozet, which, with 7 vessels operating in an area of 17,900 km^2^, hosts one of the largest fleets and one of the smallest fishing areas of the Southern Ocean.

Interestingly, increased density of vessels in fishing areas was associated with decreased sperm whale and killer whale interactions at the individual vessel level. This effect may result from a limited number of depredating specialist individuals which, once they have found a vessel, may keep interacting with its fishing gear until this vessel leaves and travels over distances sufficiently large to outrun the whales. Consequently, increased numbers of vessels operating simultaneously in the same region may generate a “dilution” effect decreasing the level of whale interaction per vessel^50^.

Greater vessel mobility was associated with decreased interaction levels for killer whales. Increased vessel mobility may reduce interaction levels either by limiting the detectability/predictability of vessels prior to interactions and/or by working as an effective strategy to outrun depredating whales in response to the occurrence of interaction events^50–52^. However, for sperm whales, greater mobility of vessels was associated with higher interaction levels. Firstly, this result may be due to an ineffectiveness of vessels of avoiding interactions by being mobile because of naturally large densities of sperm whales overlapping with areas of fishing operations. Varying densities of sperm whales across areas used by different fisheries may also explain the significance of the fishery-mobility interaction terms in models. For instance, vessels were more mobile at Crozet and Kerguelen than in any of the other fisheries, but these two areas were recently described as hosting densities of depredating sperm whales substantially larger than densities of killer whales^17,53^. In such areas, the probabilities of interaction with any sperm whale may be high across large proportions of fishing areas and vessels may, therefore, experience high levels of interaction regardless of their mobility. Secondly, this result may also be explained by sperm whales actively following vessels, and vessels not moving on distance great enough to outrun these whales. In a recent study, Janc *et al*.^51^ showed a drop in the probability of sperm whale interaction when vessels travelled over a range of 40 to 60 km between sets. While this distance is lower than the distance estimated for killer whale (100 km^50^), it is likely that vessels are less incline to implement costly strategies of avoidance of sperm whales given the lower impact of that species on catch rates compared to that of killer whales^15,27^.

Interestingly, the use of trotline equipped with cachalotera, a fishing system designed to prevent whales from accessing fish caught on longlines^32^, did not significantly influence the level of killer whale-vessel interactions, and was associated with higher levels of sperm whale-vessel interactions. Therefore, it is unlikely that the significant decrease in both sperm and killer whale interaction levels observed in Chile since 2006, when vessels switched from the autoline to the trotline and the cachalotera system^23,32^, may be attributed to that change in fishing system. While cachaloteras may increase the difficulty for whales to remove fish from hooks^54,55^, this study suggests that whales still gain benefit from feeding off longlines equipped with such a system. In addition, if cachaloteras are effective means to lower depredation and maintain high catch rates, vessels may be more likely to stay and keep fishing despite the presence of depredating sperm whales, further increasing interactions with this species. Further research is therefore needed to identify the causes of the decrease in killer and sperm whale interactions in the Chilean fishery. As this fishery has undergone substantial decreases in both quotas and fleet size^56^, it is possible that lower numbers of vessels paired with the implementation of fishing strategies being more effective in avoiding depredation have contributed to this decrease.

Part of the variability in interaction rates across fisheries was due to unexplained area-specific factors. The importance of such local factors was further emphasized by different levels of correlation between the spatial spread of interactions and the spatial spread of fishing operations between fisheries. Spatial variations in the natural presence and density of whales in the Southern Ocean are likely to contribute to these differences. The depth at which longlines were set on the seafloor had a negative influence on the levels of both killer and sperm whale interactions with vessels, suggesting that depredating individuals in the Southern Ocean may be generally naturally distributed on the shallowest part of the bathymetric range used by fishing vessels. However, the natural distribution of the depredating whales is likely to be influenced by a number of other habitat drivers that have characteristics which may differ between areas where fisheries operate. For instance, the distribution of mature male sperm whales at high latitudes was found to be highly correlated with oceanographic variables, such as frontal zones, bathymetric slope and primary productivity likely to drive the abundance and availability of their natural prey items^42,44,45^. These prey items may include Patagonian toothfish but also cephalopods, a resource with a distribution and abundance that is highly influenced by oceanographic processes. The variability of these processes across the Southern Ocean^57^ may, therefore, greatly influence the degree of overlap between sperm whales and fishing operations.

Among other unexplained area-specific factors, local ecological specializations may also influence the natural distribution patterns and movements and, therefore, the degree of overlap of whales with fishing operations. Such specializations have been extensively described across killer whale populations, including among those involved in interactions in the Southern Ocean^58,59^. For instance, killer whales interacting with fisheries are all fish specialists or generalist foragers whereas individuals feeding exclusively on marine mammals have never been observed undertaking this behaviour^14,60–62^.

In addition, the probability of whales to switch from natural foraging to depredation may also depend upon the level of experience to this behaviour and, therefore, on the history of the fisheries and the number of years whales have been exposed to fishing operations^63^. Depredation is assumed to be a learnt artificial behaviour and likely transmitted across individuals of populations through social pathways^63^. As such, and paired with natural individual heterogeneity in foraging behaviours, the experience of depredating whales, their propensity to find/follow vessels and to efficiently remove fish from longlines may vary between fisheries.

Finally, the influence of the fishing vessel itself on the occurrence of depredation interactions was not examined in this study and would require a dedicated investigation. From previous studies, odontocetes were found to detect fishing vessels through specific acoustic cues produced by the engine, such as cavitation noise generated during the hauling phase of longlines^46,47^. While Patagonian toothfish commercial fishing vessels operating in the Southern Ocean are similar in size and design to those in previous studies, there may be variation in the type and the level of acoustic signals vessels produce during fishing operations. As these signals may be intrinsic to the vessel itself (type of engine and propulsion, features of the hull), and/or determined by the way fishers operate the engine, further studies should examine whether variation in the acoustic detectability of vessels for whales may also contribute to differences of depredation levels reported between fishing areas of the Southern Ocean.

In summary, sperm whale and killer whale interaction with Patagonian toothfish fishing vessels is a widespread and established issue in the Southern Ocean. The drivers of these interactions include the spatio-temporal patterns of fishing operations and the extent to which these operations give opportunities for whales to feed on fish caught on fishing gear. Changing the simple operational aspects of fishing could, therefore, mitigate the issue. However, further research is needed to identify the factors driving whale habitat selection, distribution, movements and the mechanisms leading these whales to switch from natural foraging to depredation interactions. These drivers, which depend upon the ecology of local whale populations, could be used to better predict the occurrence of interactions and may, therefore, be used to implement effective strategies of avoidance in the future.

## Methods

### Data collection and standardisation

Fishing and whale interaction data from the seven study fisheries were collected by fishery observers and/or crews over periods ranging from 3 to 14 years. These fisheries are all fully controlled by local and/or international (Commission for the Conservation of Antarctic Marine Living Resources – “CCAMLR”) management authorities and all fishing operations are monitored. Data from Chile and the Falklands, regions which are not part of the CCAMLR Convention Area, were collected by fishery observers following protocols based on those used by CCAMLR observers in the other fisheries of the study. Data from all vessels legally operating in these fisheries and all fishing trips of these vessels were therefore accessed for the study. In all fisheries, the base unit was the longline set *i.e*. a mainline bearing series of hooks (autoline) or clusters of hooks (trotline) with, at each end, one anchor at the bottom connected to a buoy at the surface by a downline. For each longline set, fishery observers and/or crews collected the same data on the date and time, as well as GPS coordinates, at setting (*i.e*. deployed at sea) and at hauling (*i.e*. retrieved and landed on-board), in the same way in all seven fisheries.

The occurrence of whale depredation interactions with longline sets was recorded during hauling operations by visual cues. An interaction was confirmed when one of these two species, or the two species co-occurring with a typical depredation behaviour were sighted within a 500 m range from the vessel. During depredation, individuals made repeated dives towards the line being hauled and throughout the hauling process, they were usually surrounded by birds when surfacing after long dives, and slicks of fish oil were visible at the surface. When all these cues were observed, true depredation interaction events (recorded as 1) were monitored in a standardised way across all fisheries. However, only the Crozet, Kerguelen and South Georgia fishery observers distinguished between longline sets with confirmed non-occurrence of depredation (recorded as 0) and sets with lacking information due to insufficient or impossible monitoring effort (recorded as “N/A”) caused by poor weather (e.g. fog), sea or light conditions. As Chile, Falklands and HIMI recorded zeros for sets with either a true non-occurrence of depredation and/or a set for which the occurrence of depredation was unknown, we consider all the Crozet, Kerguelen and South Georgia sets with N/A’s as zeros for the sake of between-fisheries standardisation needed for this study. As a result, the estimates of depredation should to be considered as minimum estimates.

Differences in spatial and temporal frequencies of killer whale and sperm whale interactions were estimated using two indices, which were both calculated annually for each fishery per vessel (one value for each vessel that operated in a given fishery during a given year), and per fleet (one value for all data collected in a given fishery during a given year regardless of the vessel identity). Firstly, we calculated the proportion of fishing days (days of hauling operations only) with a minimum of one depredated longline set during the day out of all fishing days per year (*Pr(days)*). Secondly, we calculated a proportion of the fishing area for which depredation interactions occurred as the number of 0.1° latitude × 0.1° longitude cells in which a minimum of one longline set was depredated out of the total number of cells in which fishing occurred (*Pr(area)*).

### Statistical analyses

Annual trends of whale-fishery interaction levels over the study periods were examined using linear regressions. PEMI was excluded from this analysis due to the limited time series (n = 3 years of data) available for that fishery. Trends were tested on *Pr(days)* calculated per vessel (several values per year depending on the number of vessels) or per fleet (a single value per year), separately for killer whales and sperm whales, in each fishery and across all fisheries. In addition, a regression analysis was conducted to investigate the inter-annual changes in *Pr(area)* in relation to inter-annual changes in the spatial spread of the fishing effort. For this analysis *Pr(area)* was calculated annually as a cumulative number of new 0.1° × 0.1° cells in which interactions occurred every year, out of the total number of 0.1° × 0.1° cells fished during the respective study periods in the respective fisheries. The spatial spread of fishing effort was calculated annually as the cumulative number of new 0.1° × 0.1° cells in which fishing occurred every year, out of the total number of 0.1° × 0.1° cells fished during the respective study periods in the respective fisheries.

The influence of fishing operations on *Pr(days)* was investigated using Generalised Linear Models (GLMs). GLMs were developed for each species at both the vessel (using individual *Pr(days)* values per vessel per year per fishery) and the fleet (using individual *Pr(days)* values per year per fishery) levels. As fisheries differed in fleet size and study periods, the number of *Pr(days)* values per vessel per year varied between fisheries and ranged from 1.5 ± 0.3 vessels per year (n = 5 values) in PEMI to 8.4 ± 0.8 (n = 109 values at South Georgia, Table 3).

**Table 3:** Summary of terms used as fishing patterns variables and considered in GLMs fitted on the level of whale-vessels interaction levels.

Term	Unit	Chile	Falklands	South Georgia	PEMI	Crozet	Kerguelen	HIMI
	vessels per year	N_total_ = 60	29	109	5	96	102	20
		mean = 5.5 ± 0.5	2.1 ± 0.4	8.4 ± 0.8	1.5 ± 0.3	6.9 ± 0.2	7.3 ± 0.2	3.3 ± 0.6
		range = [3–8]	[1–6]	[6–16]	[1–2]	[5–8]	[7–9]	[2–6]
*Fishing effort*	n days	116 ± 9	101 ± 11	75 ± 2	83 ± 19	38 ± 2	131 ± 3	128 ± 8
*Spatial spread of effort*	n 0.1 × 0.1° cells	308 ± 12	248 ± 15	303 ± 3	127 ± 19	205 ± 4	750 ± 10	450 ± 37
*Density of vessels*	n vessels/100 cells	1.04 ± 0.05	0.59 ± 0.05	2.00 ± 0.04	1.04 ± 0.2	1.19 ± 0.05	0.06 ± 0.02	0.06 ± 0.02
*Seasonal spread of effort*	proportion of days in winter	0.03 ± 0.02	0.24 ± 0.04	0.63 ± 0.02	0.31 ± 0.09	0.16 ± 0.02	0.10 ± 0.01	0.47 ± 0.05
*Mobility of vessels*	n cells/day	0.87 ± 0.03	1.2 ± 0.05	0.85 ± 0.02	1.07 ± 0.12	1.55 ± 0.03	1.43 ± 0.03	1.02 ± 0.04
*Depth of sets*	meters	1580 ± 20	1341 ± 25	1248 ± 14	1339 ± 43	1099 ± 19	1186 ± 14	1311 ± 51
*Fishing technique*	proportion of sets with trotline & cachalotera	0.88 ± 0.04	0.48 ± 0.09	0 ± 0	0.52 ± 0.17	0 ± 0	0 ± 0	0 ± 0

Mean ± SE per vessel per year are here presented for each of the seven studied Patagonian toothfish fisheries of the Southern Ocean. N_total_ (total number of vessel per year values), the mean and the range of the number of vessels operating in fisheries per year are provided.

A series of binomial GLMs with logit link functions were fitted using the function glm in R 3.3.0^59^ to the proportion of total fishing days for each vessel, in each year (Table 3) where depredation was observed. To account for variability in the number of days each vessel/fleet fished the total number of days fished each year was used as the model weights (i.e. equivalent to using the *weights* argument in the *glm* function in R) for each vessel/fleet. The fishery was included in models as a categorical variable with seven levels for each of the studied fisheries, with Chile being the fishery compared to each one of the others. The other predictors included were all continuous and were calculated as annual values, either at the vessel or at the fleet level, as follows i) the spatial spread of fishing effort calculated as the total number of 0.1° × 0.1° spatial cells in which at least one set was hauled by vessels; ii) the mean density of vessels per fishing day, calculated as the mean number of different vessels operating during the same day in the same fishery out of the spatial spread of fishing effort previously calculated; iii) the seasonal spread of fishing effort, measured as the proportion of fishing days during winter months (from 1 June to 31 Aug) out of all fishing days during a given year; iv) the mobility of vessels, calculated as the ratio between the spatial spread of fishing effort and the total number of fishing days during a given year; v) the mean depth at which longlines were set; and vi) the fishing system, calculated as the proportion of sets using trotlines equipped with cachalotera out of all sets (Table 3). In addition to the single predictors described above, we also tested an interaction between fishery and mobility, when both were present in the optimal model. Collinearity between continuous predictors was checked using Pearson tests and predictors were retained if r < 0.8 (Table S3). All continuous predictors were centred then scaled using the *scale* function in R and variable selection was conducted using stepwise forward selection of models with the lowest Akaike Information Criterion (AIC)^64^. When multiple models were within 2 AIC of the model with the lowest AIC (i.e. the optimal model) we considered all of them. The proportion of the total variance explained was quantified for each model using the pseudo r^2^ statistic^65^. Model estimates are presented as probabilities with 95% confidence intervals by applying an inverse logit transformation.

### Guidelines and regulations

All methods were carried out in accordance with relevant ethical guidelines and regulations of Deakin University, Australia. Data used in this manuscript were collected by national and international fishery observers under the authority of CCAMLR, Instituto de Fomento Pesquero (IFOP) and the Fisheries Department of the Falkland Islands Government.

## Electronic supplementary material

Table S1

Table S2

Table S3

Data S1

Data S2

## Data Availability

The datasets generated during and/or analysed during the current study are available from the corresponding author on reasonable request.
