# Supplementary material for: Commercial fishing patterns influence odontocete whale-longline interactions in the Southern Ocean
Source: Sci Rep. 2019 Feb 13;9:1904. doi: 10.1038/s41598-018-36389-x (PMC6374415; doi:10.1038/s41598-018-36389-x)
Supplement: Supplementary file 1 — Table S1 [file 41598_2018_36389_MOESM1_ESM.pdf]

Commercial fishing patterns influence odontocete whale-longline interactions in the Southern Ocean

P. Tixier, P. Burch, G. Richard, K. Olsson, D. Welsford, M.-A. Lea, M. A. Hindell, C. Guinet, A. Janc, N. Gasco, G. Duhamel, M. C. Villanueva, L. Suberg, R. Arangio, M. Söffker, J. P. Y. Arnould

Table S1.

Temporal trends of whale-vessel interaction levels

June 6th, 2018

Table S1. Parameter estimates of the linear models testing for annual trends in a. sperm whale-, and b. killer whale – fisheries interaction levels in the study fishing areas. Interaction levels were calculated as the proportion of fishing days with interactions recorded out of all fishing days at the vessel level (*$Pr(days)_{vessel}$*) and at the fleet level (*$Pr(days)_{fleet}$*)

a. sperm whales

Area	*n* years	Response	Trend			
			Est.	***SE***	***t***	***P***
All	14	$Pr(days)_{vessel}$	-0.009	0.003	-2.697	0.073
		$Pr(days)_{fleet}$	-0.012	0.007	-1.695	0.092
Chile	11	$Pr(days)_{vessel}$	-0.040	0.012	-3.513	**0.001**
		$Pr(days)_{fleet}$	-0.015	0.008	-1.891	0.064
Falklands	14	$Pr(days)_{vessel}$	0.023	0.009	2.696	**0.012**
		$Pr(days)_{fleet}$	0.016	0.008	2.114	0.056
South Georgia	13	$Pr(days)_{vessel}$	0.001	0.005	0.174	0.862
		$Pr(days)_{fleet}$	-0.019	0.006	-3.227	**0.008**
Crozet	14	$Pr(days)_{vessel}$	-0.008	0.004	-2.067	**0.041**
		$Pr(days)_{fleet}$	0.000	0.003	-0.120	0.906
Kerguelen	14	$Pr(days)_{vessel}$	-0.011	0.004	-2.793	**0.006**
		$Pr(days)_{fleet}$	-0.005	0.003	-1.350	0.202
HIMI	6	$Pr(days)_{vessel}$	0.004	0.006	0.684	0.503
		$Pr(days)_{fleet}$	0.020	0.007	2.878	**0.045**

b. killer whales

Area	*n* years	Response	Trend			
			Est.	***SE***	***t***	***P***
All	14	$Pr(days)_{vessel}$	0.001	0.003	0.426	0.671
		$Pr(days)_{fleet}$	-0.007	0.009	-0.855	0.396
Chile	11	$Pr(days)_{vessel}$	-0.027	0.012	-2.312	**0.024**
		$Pr(days)_{fleet}$	-0.008	0.010	-0.850	0.399
Falklands	14	$Pr(days)_{vessel}$	0.002	0.001	1.274	0.213
		$Pr(days)_{fleet}$	0.002	0.002	0.854	0.410
South Georgia	13	$Pr(days)_{vessel}$	0.005	0.002	2.876	**0.005**
		$Pr(days)_{fleet}$	-0.002	0.005	-0.321	0.754
Crozet	14	$Pr(days)_{vessel}$	-0.002	0.005	-0.390	0.698
		$Pr(days)_{fleet}$	-0.001	0.004	-0.312	0.761
Kerguelen	14	$Pr(days)_{vessel}$	0.000	0.000	-1.268	0.208
		$Pr(days)_{fleet}$	-0.001	0.001	-0.801	0.439
HIMI	6	$Pr(days)_{vessel}$	0.000	0.000	-	-
		$Pr(days)_{fleet}$	0.000	0.000	-	-
