# Supplementary material for: Commercial fishing patterns influence odontocete whale-longline interactions in the Southern Ocean
Source: Sci Rep. 2019 Feb 13;9:1904. doi: 10.1038/s41598-018-36389-x (PMC6374415; doi:10.1038/s41598-018-36389-x)
Supplement: Supplementary file 2 — Table S2 [file 41598_2018_36389_MOESM2_ESM.pdf]

Commercial fishing patterns influence odontocete whale-longline interactions in the Southern Ocean

P. Tixier, P. Burch, G. Richard, K. Olsson, D. Welsford, M.-A. Lea, M. A. Hindell, C. Guinet, A. Janc, N. Gasco, G. Duhamel, M. C. Villanueva, L. Suberg,

R. Arangio, M. Söffker, J. P. Y. Arnould

Table S2

AIC selection for the GLMs fitted to the annual proportions of fishing days with whale depredation interactions

August 28th, 2018

Table S2.1. AIC selection table for GLMs fitted to *Pr(days)* of sperm whales at the vessel level. Models are ordered by increasing AIC values and coefficient estimates are provided for the intercept and for continuous covariates when included in models. (*) indicates an interaction term between two variables, (+) indicates a categorical variable included in the model and blanks indicate that the variable was not included in the model. A correlation coefficient (pseudo r^2) is also provided.

Intercept	Depth	Proportion effort cachalotera	Proportion effort winter	Total area size	Density of vessels	Fishery	Mobility of vessels	Fishery* mobility	df	logLik	AIC	ΔAIC	pseudo r^2	weight
0.37	-0.04	0.11	-0.14	-0.43	-0.07	+	0.48	+	19	-2900.23	5838.46	0.00	0.55	1.00
0.02	-0.06	0.12	-0.14	-0.45	-0.06	+	0.13		13	-2927.87	5881.75	43.29	0.55	0.00
0.05	-0.06	0.12	-0.13	-0.44		+	0.14		12	-2929.85	5883.69	45.24	0.55	0.00
-0.09		0.12	-0.14	-0.46	-0.05	+	0.12		12	-2933.31	5890.62	52.16	0.54	0.00
-0.06		0.12	-0.13	-0.46		+	0.13		11	-2934.64	5891.28	52.83	0.54	0.00
0.18	-0.05	0.10		-0.45		+	0.15		11	-2943.43	5908.86	70.40	0.54	0.00
0.08		0.10		-0.46		+	0.14		10	-2947.99	5915.99	77.53	0.54	0.00
0.07		0.10		-0.47	-0.03	+	0.14		11	-2947.50	5916.99	78.54	0.54	0.00
0.23			-0.11	-0.47		+	0.15		10	-2953.60	5927.20	88.74	0.54	0.00
0.42	-0.05			-0.45		+	0.17		10	-2958.89	5937.79	99.33	0.54	0.00
0.32				-0.47		+	0.16		9	-2963.15	5944.29	105.83	0.54	0.00
0.31				-0.47	-0.03	+	0.16		10	-2962.56	5945.12	106.67	0.54	0.00
-0.10		0.12		-0.41		+			9	-2971.78	5961.56	123.11	0.54	0.00
0.07			-0.12	-0.41		+			9	-2982.44	5982.88	144.43	0.53	0.00
0.13				-0.42	-0.08	+			9	-2990.92	5999.85	161.39	0.53	0.00
0.23	-0.04			-0.40		+			9	-2992.48	6002.95	164.49	0.53	0.00
0.16				-0.41		+			8	-2994.50	6005.00	166.54	0.53	0.00
-0.02		0.12				+			8	-3043.08	6102.16	263.70	0.52	0.00
0.36						+	0.10		8	-3052.21	6120.43	281.97	0.52	0.00
0.15			-0.12			+			8	-3053.54	6123.09	284.63	0.52	0.00
0.37	-0.07					+			8	-3056.70	6129.40	290.95	0.52	0.00
0.25						+			7	-3065.38	6144.77	306.31	0.52	0.00
0.23					-0.03	+			8	-3064.70	6145.40	306.94	0.52	0.00
-0.16			-0.52						2	-4530.49	9064.99	3226.53	0.20	0.00
-0.11							0.35		2	-5025.35	10054.70	4216.24	0.09	0.00
-0.20					-0.23				2	-5272.90	10549.79	4711.34	0.04	0.00
-0.17		0.14							2	-5362.38	10728.76	4890.30	0.02	0.00
-0.14	-0.09								2	-5435.47	10874.94	5036.49	0.01	0.00
-0.17				0.05					2	-5455.40	10914.80	5076.34	0.00	0.00
-0.15									1	-5466.33	10934.66	5096.20	0.00	0.00

Table S2.2. AIC selection table for GLMs fitted to *Pr(days)* of sperm whales at the fleet level. Models are ordered by increasing AIC values and coefficient estimates are provided for the intercept and for continuous covariates when included in models. (*) indicates an interaction term between two variables, (+) indicates a categorical variable included in the model and blanks indicate that the variable was not included in the model. A correlation coefficient (pseudo r^2) is also provided.

Intercept	Depth	Proportion effort cachalotera	Proportion effort winter	Total area size	Density of vessels	Fishery	Mobility of vessels	Fishery* mobility	df	logLik	AIC	ΔAIC	pseudo r^2	weight
4.56	-0.25	0.09	-0.18	-0.27	0.32	+	3.65	+	19	-370.59	779.19	0.00	0.90	1.00
1.91	-0.47	0.19	-0.19	-0.20	0.12	+	0.54		13	-443.69	913.38	134.20	0.87	0.00
1.95	-0.49	0.19	-0.19	-0.29		+	0.60		12	-445.72	915.43	136.24	0.86	0.00
1.76	-0.46	0.21	-0.20		0.18	+	0.41		12	-446.06	916.12	136.93	0.86	0.00
1.73	-0.49	0.24	-0.21			+	0.41		11	-452.22	926.45	147.26	0.86	0.00
2.16	-0.49	0.17		-0.32		+	0.60		11	-453.23	928.47	149.28	0.86	0.00
1.96	-0.45	0.19			0.19	+	0.39		11	-454.76	931.53	152.34	0.86	0.00
1.94	-0.48	0.22				+	0.39		10	-461.20	942.40	163.21	0.86	0.00
2.48	-0.48			-0.43		+	0.69		10	-461.99	943.98	164.79	0.86	0.00
2.27	-0.43				0.24	+	0.40		10	-467.38	954.76	175.57	0.85	0.00
2.16	-0.47		-0.16			+	0.42		10	-472.62	965.25	186.06	0.85	0.00
2.29	-0.46					+	0.41		9	-478.25	974.49	195.30	0.85	0.00
1.41	-0.40	0.24				+			9	-498.04	1014.09	234.90	0.84	0.00
1.75	-0.34				0.25	+			9	-505.25	1028.50	249.31	0.83	0.00
1.65	-0.38		-0.12			+			9	-514.95	1047.89	268.70	0.83	0.00
1.82	-0.39			0.13		+			9	-515.33	1048.66	269.47	0.83	0.00
1.77	-0.38					+			8	-518.17	1052.35	273.16	0.83	0.00
1.14					0.33	+			8	-540.19	1096.38	317.19	0.82	0.00
1.31						+	0.28		8	-542.23	1100.46	321.27	0.81	0.00
0.73		0.20				+			8	-548.70	1113.40	334.21	0.81	0.00
0.95			-0.13			+			8	-559.95	1135.90	356.71	0.81	0.00
1.07						+			7	-563.39	1140.78	361.59	0.80	0.00
1.07				0.05		+			8	-562.92	1141.84	362.65	0.80	0.00
0.79					0.40				2	-1967.33	3938.66	3159.47	0.08	0.00
0.63			-0.33						2	-1991.62	3987.24	3208.05	0.07	0.00
0.70	-0.25								2	-2010.38	4024.75	3245.56	0.06	0.00
0.66							0.25		2	-2023.78	4051.55	3272.36	0.05	0.00
0.62				0.19					2	-2063.61	4131.21	3352.03	0.03	0.00
0.67									1	-2122.42	4246.85	3467.66	0.00	0.00
0.67		-0.01							2	-2122.35	4248.70	3469.51	0.00	0.00

Table S2.3. AIC selection table for GLMs fitted to *Pr(days)* of killer whales at the vessel level. Models are ordered by increasing AIC values and coefficient estimates are provided for the intercept and for continuous covariates when included in models. (*) indicates an interaction term between two variables, (+) indicates a categorical variable included in the model and blanks indicate that the variable was not included in the model. A correlation coefficient (pseudo r^2) is also provided.

Intercept	Depth	Proportion effort cachalotera	Proportion effort winter	Total area size	Density of vessels	Fishery	Mobility of vessels	Fishery* Mobility	df	logLik	AIC	ΔAIC	pseudo r^2	weight
-0.78	-0.25			-0.82	-0.29	+	-0.16	+	17	-1294.13	2622.27	0.00	0.85	0.33
-0.70	-0.24			-0.80	-0.32	+	-0.06		11	-1300.83	2623.65	1.39	0.85	0.17
-0.75	-0.24	0.02		-0.81	-0.32	+	-0.07		12	-1300.60	2625.21	2.94	0.85	0.08
-0.69	-0.24		0.02	-0.81	-0.32	+	-0.06		12	-1300.67	2625.34	3.08	0.85	0.07
-0.63	-0.24			-0.80	-0.31	+			10	-1302.80	2625.60	3.33	0.85	0.06
-0.61	-0.24		0.03	-0.80	-0.31	+			11	-1302.55	2627.11	4.84	0.85	0.03
-0.66	-0.24	0.02		-0.80	-0.31	+			11	-1302.67	2627.35	5.08	0.85	0.03
-0.50	-0.21		0.07	-0.70		+			10	-1331.72	2683.44	61.17	0.84	0.00
-0.60	-0.20			-0.69		+	-0.05		10	-1332.29	2684.57	62.31	0.84	0.00
-0.55	-0.21			-0.69		+			9	-1333.36	2684.72	62.45	0.84	0.00
-0.64	-0.21	0.04		-0.70		+			10	-1332.52	2685.05	62.78	0.84	0.00
-1.02				-0.81	-0.26	+			9	-1339.89	2697.78	75.51	0.84	0.00
-0.97				-0.72		+	-0.06		9	-1359.10	2736.19	113.93	0.83	0.00
-0.85			0.07	-0.73		+			9	-1359.33	2736.66	114.39	0.83	0.00
-0.90				-0.72		+			8	-1361.05	2738.11	115.84	0.83	0.00
-0.98		0.04		-0.73		+			9	-1360.40	2738.80	116.53	0.83	0.00
-0.37	-0.22					+			8	-1383.94	2783.88	161.61	0.83	0.00
-0.81					-0.17	+			8	-1406.69	2829.39	207.12	0.82	0.00
-0.80						+	-0.05		8	-1414.87	2845.73	223.46	0.82	0.00
-0.74						+			7	-1416.25	2846.49	224.23	0.82	0.00
-0.72			0.03			+			8	-1415.88	2847.76	225.50	0.82	0.00
-0.71		-0.01				+			8	-1416.16	2848.32	226.06	0.82	0.00
-2.18				-1.53					2	-3534.58	7073.16	4450.89	0.40	0.00
-2.01		0.32							2	-5251.14	10506.28	7884.01	0.07	0.00
-1.96			-0.38						2	-5385.85	10775.71	8153.44	0.04	0.00
-1.89					0.22				2	-5493.02	10990.04	8367.77	0.02	0.00
-1.95	0.16								2	-5540.86	11085.72	8463.45	0.01	0.00
-1.94							-0.14		2	-5554.72	11113.44	8491.18	0.01	0.00
-1.91									1	-5587.30	11176.61	8554.34	0.00	0.00

Table S2.4. AIC selection table for GLMs fitted to *Pr(days)* of killer whales at the fleet level. Models are ordered by increasing AIC values and coefficient estimates are provided for the intercept and for continuous covariates when included in models. (*) indicates an interaction term between two variables, (+) indicates a categorical variable included in the model and blanks indicate that the variable was not included in the model. A correlation coefficient (pseudo r^2) is also provided.

Intercept	Depth	Proportion effort cachalotera	Proportion effort winter	Total area size	Density of vessels	Fishery	Mobility of vessels	df	logLik	AIC	ΔAIC	pseudo r^2	weight
0.74	-0.30				0.09	+	0.12	10	-293.67	607.33	0.00	0.95	0.15
0.64	-0.30				0.13	+		9	-294.67	607.34	0.01	0.95	0.15
0.80	-0.32					+	0.18	9	-294.78	607.57	0.23	0.95	0.14
0.60	-0.30		-0.05		0.13	+		10	-294.26	608.53	1.20	0.95	0.08
0.64	-0.30			-0.03	0.12	+		10	-294.62	609.23	1.90	0.95	0.06
0.67	-0.30	-0.02			0.13	+		10	-294.65	609.29	1.96	0.95	0.06
0.67	-0.33					+		8	-297.82	611.64	4.31	0.95	0.02
0.67	-0.33			-0.08		+		9	-297.37	612.74	5.41	0.95	0.01
0.63	-0.33		-0.04			+		9	-297.54	613.08	5.74	0.95	0.01
0.68	-0.33	-0.01				+		9	-297.82	613.64	6.30	0.95	0.01
0.11					0.19	+		8	-311.59	639.19	31.86	0.95	0.00
0.26						+	0.23	8	-314.07	644.14	36.81	0.95	0.00
0.07						+		7	-319.11	652.22	44.89	0.94	0.00
0.18		-0.06				+		8	-318.85	653.70	46.37	0.94	0.00
0.04			-0.03			+		8	-318.88	653.76	46.43	0.94	0.00
0.07				-0.01		+		8	-319.11	654.21	46.88	0.94	0.00
-1.14				-1.12				2	-2237.04	4478.08	3870.75	0.33	0.00
-0.99					0.80			2	-2577.60	5159.21	4551.87	0.22	0.00
-1.21							-0.76	2	-2638.61	5281.22	4673.88	0.20	0.00
-1.14		0.30						2	-3112.46	6228.93	5621.60	0.04	0.00
-1.15			-0.23					2	-3195.55	6395.10	5787.77	0.02	0.00
-1.12	0.15							2	-3214.44	6432.88	5825.55	0.01	0.00
-1.11								1	-3245.90	6493.79	5886.46	0.00	0.00
