# Supplementary material for: Commercial fishing patterns influence odontocete whale-longline interactions in the Southern Ocean
Source: Sci Rep. 2019 Feb 13;9:1904. doi: 10.1038/s41598-018-36389-x (PMC6374415; doi:10.1038/s41598-018-36389-x)
Supplement: Supplementary file 3 — Table S3 [file 41598_2018_36389_MOESM3_ESM.pdf]

Commercial fishing patterns influence odontocete whale-longline interactions in the Southern Ocean

P. Tixier, P. Burch, G. Richard, K. Olsson, D. Welsford, M.-A. Lea, M. A. Hindell, C. Guinet, A. Janc, N. Gasco, G. Duhamel, M. C. Villanueva, L. Suberg, R. Arangio, M. Söffker, J. P. Y. Arnould

Table S3

Collinearity tests for the GLMs fitted to the annual proportions of fishing days with whale depredation interactions

August 28th, 2018

Table S3. Results from the tests of collinearity between covariates used in the GLMs fitted to the proportion of fishing days with sperm whale and killer whale interactions recorded out of all fishing days at the vessel level (*$Pr(days)_{vessel}$*) and at the fleet level (*$Pr(days)_{fleet}$*).

Vessel level

Covariate correlation (r)	Depth	Total size of fishing area	Density of vessels	Fishing effort	Mobility of vessels	Proportion of days in winter	Proportion of effort with cachaloteras
**Depth**	1.00	-0.02	-0.08	0.26	-0.25	-0.03	0.49
**Total size of fishing area**	-0.02	1.00	-0.48	0.51	0.26	-0.26	-0.17
**Density of vessels**	-0.08	-0.48	1.00	-0.35	-0.45	0.51	-0.18
**Fishing effort**	0.26	0.51	-0.35	1.00	-0.21	0.01	0.24
**Mobility of vessels**	-0.25	0.26	-0.45	-0.21	1.00	-0.39	0.20
**Proportion of days in winter**	-0.03	-0.26	0.51	0.01	-0.39	1.00	-0.22
**Proportion of effort with cachaloteras**	0.49	-0.17	-0.18	0.24	0.20	-0.22	1.00

Fleet level

Covariate correlation (r)	Depth	Total size of fishing area	Density of vessels	Fishing effort	Mobility of vessels	Proportion of days in winter	Proportion of effort with cachaloteras
**Depth**	1.00	-0.16	-0.17	0.21	-0.33	-0.14	0.70
**Total size of fishing area**	-0.16	1.00	-0.43	0.70	0.79	-0.13	-0.33
**Density of vessels**	-0.17	-0.43	1.00	-0.69	0.09	0.47	-0.15
**Fishing effort**	0.21	0.71	-0.69	1.00	0.18	-0.37	0.06
**Mobility of vessels**	-0.33	0.79	0.09	0.18	1.00	0.27	-0.53
**Proportion of days in winter**	-0.14	-0.13	0.47	-0.37	0.27	1.00	-0.31
**Proportion of effort with cachaloteras**	0.70	-0.33	-0.15	0.06	-0.53	-0.31	1.00
