# Supplementary material for: Commercial fishing patterns influence odontocete whale-longline interactions in the Southern Ocean
Source: Sci Rep. 2019 Feb 13;9:1904. doi: 10.1038/s41598-018-36389-x (PMC6374415; doi:10.1038/s41598-018-36389-x)
Supplement: Supplementary file 4 — Data S1 [file 41598_2018_36389_MOESM4_ESM.pdf]

Commercial fishing patterns influence odontocete whale-longline interactions in the Southern Ocean

P. Tixier, P. Burch, G. Richard, K. Olsson, D. Welsford, M.-A. Lea, M. Hindell, C. Guinet, A. Janc, N. Gasco, G. Duhamel, M. C. Villanueva, L. Suberg, R. Arangio, M. Söffker, J.P.Y. Arnould

Data S1

Analysis of sperm whale - fisheries interactions using Generalised Linear Models

August 28th, 2018

Load packages

```
library(lme4)

library(lmerTest)

library(merTools)

library(MuMIn)
library(ggplot2)
library(ggpubr)

library(Rmisc)
```

Function Definitions

Define the pseudo r squared statistic of Swartzman et al (1992)

```
pseudo_r2 <- function(fit, null_fit=NULL){
  if(is.null(null_fit)){
    null_dev <- fit$null.deviance
  }else{
    null_dev <- null_fit$deviance
  }
  ## calcualte pseudo r2
  pseudo_r2 <- 1- (fit$deviance/null_dev)
  ## return the pseudor2
  pseudo_r2
}
```

A Models on *Pr(days)* at the vessel level

A.1. Data preparation

Import the data

```
setwd("C:/Users/LES_research211/Documents/003_ANALYSES/009_SOUTHERN_OCEAN_PAP
ER")
data<-read.csv("final_dataset_modeling_day_interaction.csv",h=T,sep=",")
str(data)

## 'data.frame':    5088 obs. of  24 variables:
##  $ area                 : Factor w/ 7 levels "CHILE","CROZET",..: 1 1 1 1
1 1 1 1 1 1 ...
##  $ year                 : int  2006 2006 2006 2006 2006 2006 2006 2006 20
06 2006 ...
```

```
##  $ vessel                 : Factor w/ 67 levels "A","ABA","AN",..: 55 19 18
32 32 18 19 55 19 55 ...
##  $ depredation            : int  0 0 0 0 1 1 1 1 0 0 ...
##  $ count                  : int  35 33 4 0 3 59 15 10 5 2 ...
##  $ Pr_inter               : num  0.7778 0.6875 0.0635 0 1 ...
##  $ species                : Factor w/ 2 levels "killer whales",..: 1 1 1 1
1 1 1 1 2 2 ...
##  $ response               : Factor w/ 3 levels "Pr(area)","Pr(days)",..: 1
1 1 1 1 1 1 1 1 1 ...
##  $ area_yr_vessel         : Factor w/ 424 levels "CHILE 2006 GPI",..: 4 2 1
3 3 1 2 4 2 4 ...
##  $ area_size_vessel       : int  45 48 63 3 3 63 48 45 48 45 ...
##  $ count_total            : int  40 59 64 3 3 64 59 40 59 40 ...
##  $ mean_nb_vessels_vessel: num  2.61 2.41 2.36 3.33 3.33 ...
##  $ mean_lat_vessel        : num  -55.8 -55.6 -55.6 -56.3 -56.3 ...
##  $ mean_depth_vessel      : num  1536 1568 1480 1508 1508 ...
##  $ mean_effort_day        : num  8859 6845 7842 12800 12800 ...
##  $ prop_day_winter        : num  0 0 0 0 0 0 0 0 0 0 ...
##  $ prop_day_spring        : num  0.975 0.847 0.875 1 1 ...
##  $ prop_day_summer        : num  0.025 0.153 0.125 0 0 ...
##  $ prop_day_autumn        : num  0 0 0 0 0 0 0 0 0 0 ...
##  $ prop_day_spanish       : num  0 0 0 0 0 0 0 0 0 0 ...
##  $ prop_day_trotline      : num  0 0 0 0 0 0 0 0 0 0 ...
##  $ prop_day_trotline_cach: num  0.475 2.085 0.188 0 0 ...
##  $ prop_day_autoline      : num  1.5 0 1.77 1 1 ...
##  $ prop_day_japanese      : num  0 0 0 0 0 0 0 0 0 0 ...
```

Prepare the data for modelling

```
data$area_size_vessel=as.numeric(as.character(data$area_size_vessel))
data$prop_day_trotline_cach=as.numeric(as.character(data$prop_day_trotline_ca
ch))
data <-subset(data,!is.na(mean_lat_vessel))
data <-subset(data,!is.na(mean_depth_vessel))
data$mean_lat_vessel=-data$mean_lat_vessel
data$mobility=data$area_size_vessel/data$count_total

setwd("C:/Users/LES_research211/Documents/003_ANALYSES/009_SOUTHERN_OCEAN_PAP
ER")
data_fleet=read.csv("summary_proportions_predictors_fleet_pemi_a.csv",h=T,sep
=",")
str(data_fleet)

## 'data.frame':    912 obs. of  22 variables:
##  $ area                   : Factor w/ 7 levels "CHILE","CROZET",..: 6 6 6 5
5 5 5 5 5 4 ...
##  $ year                   : int  2017 2017 2017 2013 2013 2013 2016 2016 20
16 2011 ...
##  $ depredation            : int  0 0 0 0 0 0 0 0 0 0 ...
##  $ count                  : int  366 130 133 2557 253 875 2532 248 838 493
...
```

```
##  $ count_total          : int  130 130 130 253 253 253 248 248 248 191 ..
.
##  $ Pr_inter             : num  1 1 1 1 1 1 1 1 1 1 ...
##  $ species              : Factor w/ 2 levels "killer whales",..: 2 2 2 1
1 1 1 1 1 1 ...
##  $ response             : Factor w/ 4 levels "Pr(area)","Pr(days)",..: 3
2 1 3 2 1 3 2 1 3 ...
##  $ area_yr              : Factor w/ 76 levels "CHILE 2006","CHILE 2007",.
.: 62 62 62 56 56 56 59 59 59 40 ...
##  $ total_area           : int  133 133 133 875 875 875 838 838 838 223 ..
.
##  $ length_season        : int  130 130 130 253 253 253 248 248 248 191 ..
.
##  $ mean_nb_vessels      : num  1 1 1 4.38 4.38 ...
##  $ mean_lat             : num  -45.3 -45.3 -45.3 -48.9 -48.9 ...
##  $ mean_depth           : num  1352 1352 1352 1199 1199 ...
##  $ prop_day_winter      : num  0.462 0.462 0.462 0.158 0.158 ...
##  $ prop_day_spring      : num  0 0 0 0.316 0.316 ...
##  $ prop_day_summer      : num  0.0692 0.0692 0.0692 0.2451 0.2451 ...
##  $ prop_day_autumn      : num  0.469 0.469 0.469 0.281 0.281 ...
##  $ prop_day_spanish     : num  0 0 0 0 0 0 0 0 0 0 ...
##  $ prop_day_autoline    : num  0 0 0 1 1 1 1 1 1 1 ...
##  $ prop_day_trotline_cach: num  0.485 0.485 0.485 0 0 ...
##  $ prop_day_trotline    : num  0.515 0.515 0.515 0 0 ...
```

```
data$area_yr = paste(data$area,data$year)
data_fleet$area_yr = paste(data_fleet$area,data_fleet$year)
data$total_area <- data_fleet$total_area[match(data$area_yr,data_fleet$area_y
r)]
data$density =data$mean_nb_vessels_vessel/data$total_area
```

Data selection

```
d1 <- droplevels(data[data$depredation==1 & data$species %in% "sperm whales"
& data$response %in% "Pr(days)",
        c("area","year","count","count_total", "Pr_inter", "vessel","mean_dep
th_vessel","mobility","prop_day_trotline_cach", "prop_day_winter","total_area
","density" )])
str(d1)
```

```
## 'data.frame':    410 obs. of  12 variables:
##  $ area                 : Factor w/ 7 levels "CHILE","CROZET",..: 1 1 1 1
1 1 1 1 1 1 ...
##  $ year                 : int  2006 2006 2006 2006 2007 2007 2007 2007 20
08 2008 ...
##  $ count                : int  64 3 40 55 91 60 24 24 98 92 ...
##  $ count_total          : int  64 3 40 59 91 65 28 51 164 162 ...
##  $ Pr_inter             : num  1 1 1 0.932 1 ...
##  $ vessel               : Factor w/ 67 levels "A","ABA","AN",..: 18 32 55
19 18 55 19 2 19 18 ...
##  $ mean_depth_vessel    : num  1480 1508 1536 1568 1507 ...
```

```
##  $ mobility              : num  0.984 1 1.125 0.814 0.78 ...
##  $ prop_day_trotline_cach: num  0.188 0 0.475 2.085 2.176 ...
##  $ prop_day_winter       : num  0 0 0 0 0 ...
##  $ total_area            : int  141 141 141 141 173 173 173 173 237 237 ..
.
##  $ density               : num  0.01674 0.02364 0.01849 0.01707 0.00887 ..
.
```

Data exploration

```
hist(d1$Pr_inter)
```

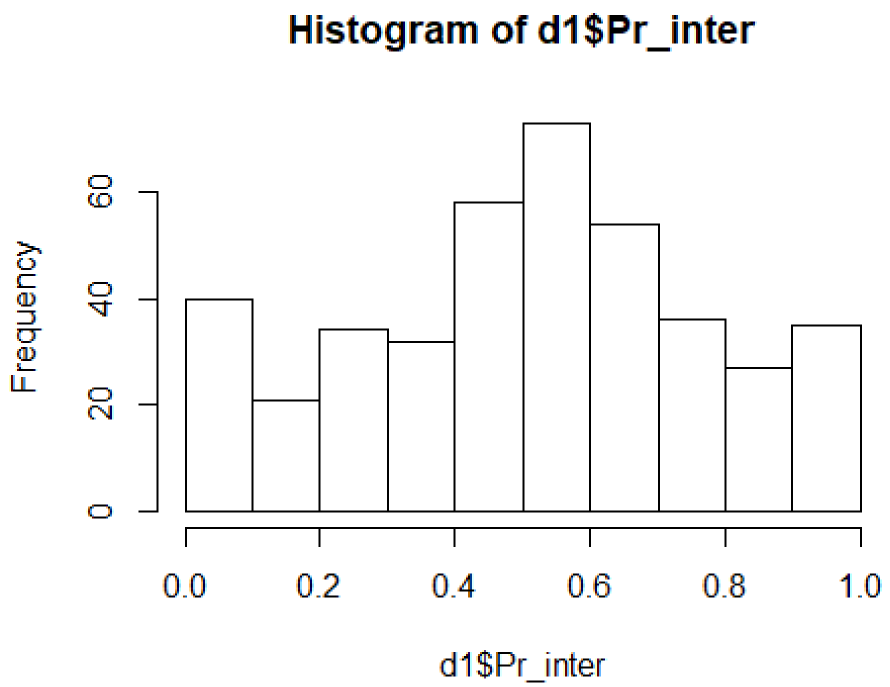


```
hist(d1$mean_depth_vessel)
```

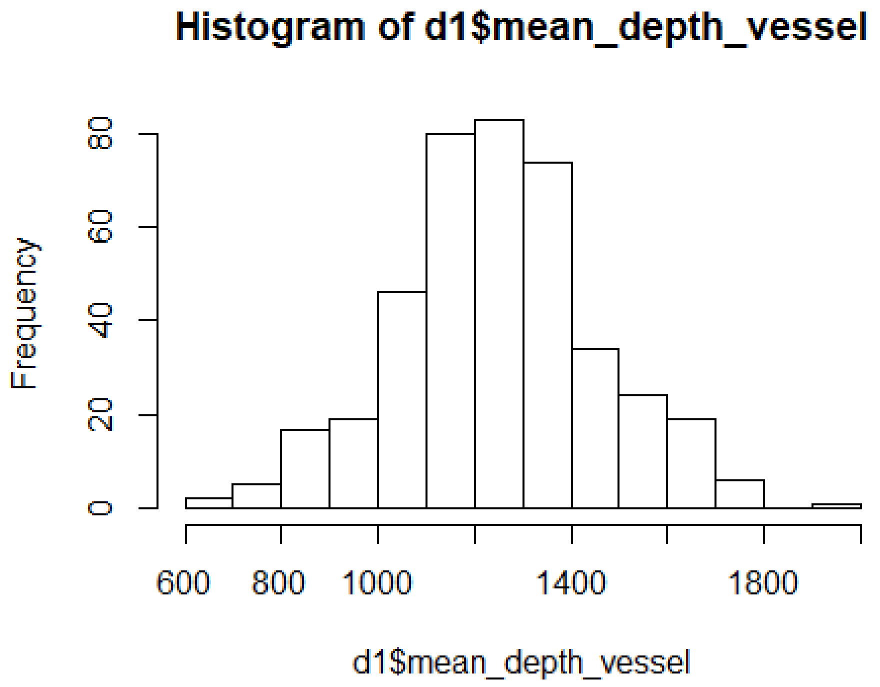


```r
hist(d1$total_area)
```

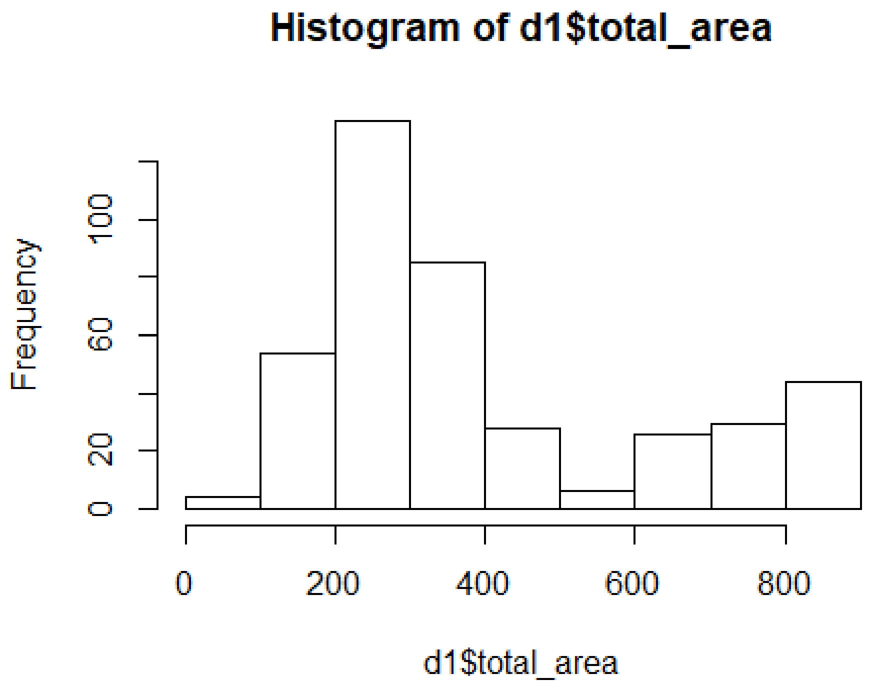


```r
hist(d1$mobility)
```

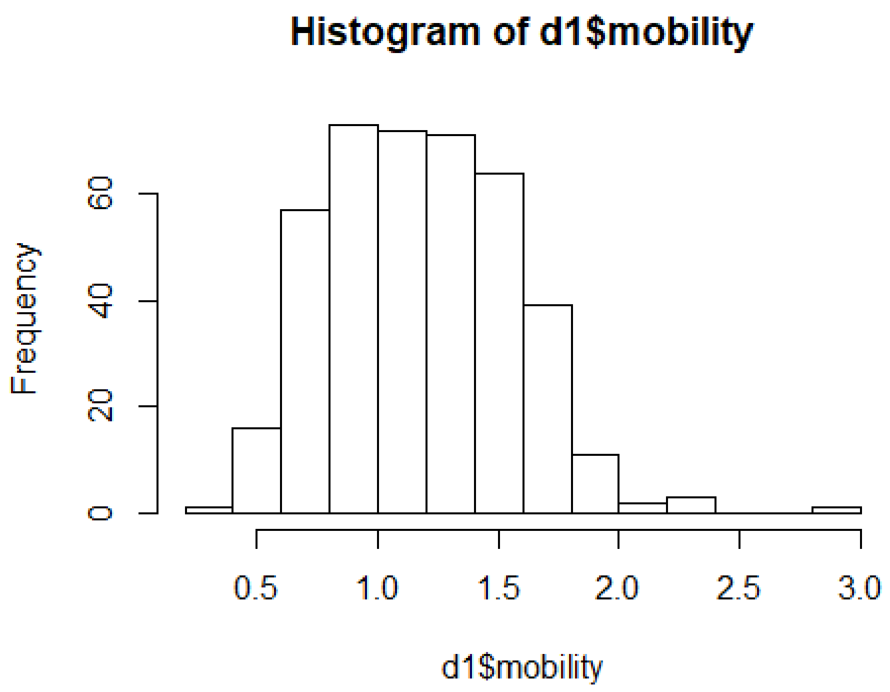


```
# exclude extreme high value
d1 = subset(d1, d1$mobility<=2.5)

hist(d1$density)
```

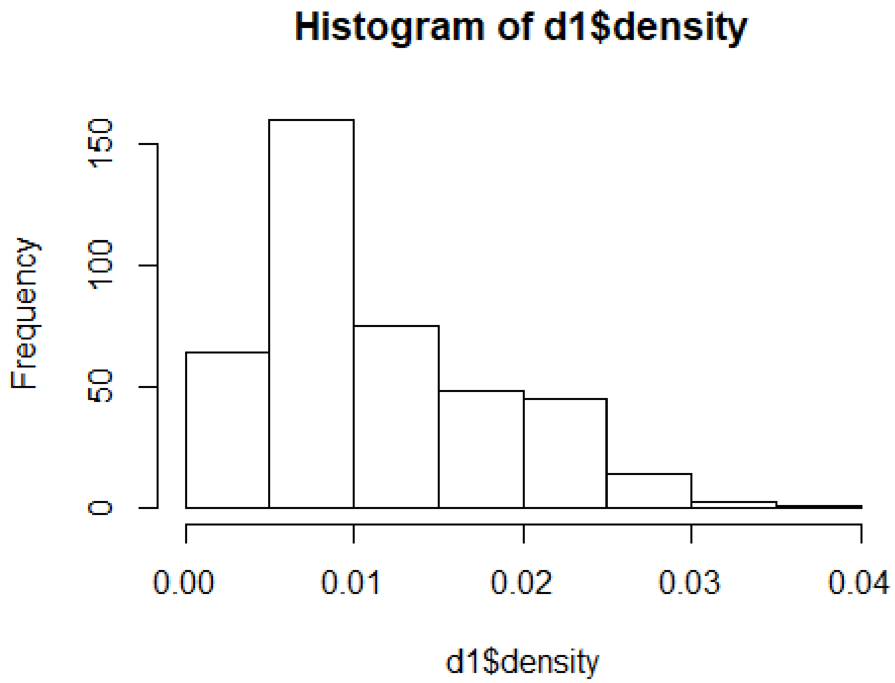


```
# exclude extreme high value
d1 = subset(d1, d1$density<=0.035)
```

Exploratory plots

```
p1 <- ggplot(d1,aes(mean_depth_vessel,Pr_inter,colour=area))+geom_point()+ggt
itle("depth")
p2 <- ggplot(d1,aes(density,Pr_inter,colour=area))+geom_point()+ggtitle("dens
ity")
p3 <- ggplot(d1,aes(prop_day_winter,Pr_inter,colour=area))+geom_point()+ggtit
le("effort in winter")
p4 <- ggplot(d1,aes(total_area,Pr_inter,colour=area))+geom_point()+ggtitle("t
otal area size")
p5 <- ggplot(d1,aes(mobility,Pr_inter,colour=area))+geom_point()+ggtitle("mob
ility")
p6 <- ggplot(d1,aes(count_total,Pr_inter,colour=area))+geom_point()+ggtitle("
nb fishing days")

ggarrange(p1, p2,p3,p4,p5,p6, common.legend=TRUE, legend = "right")
```

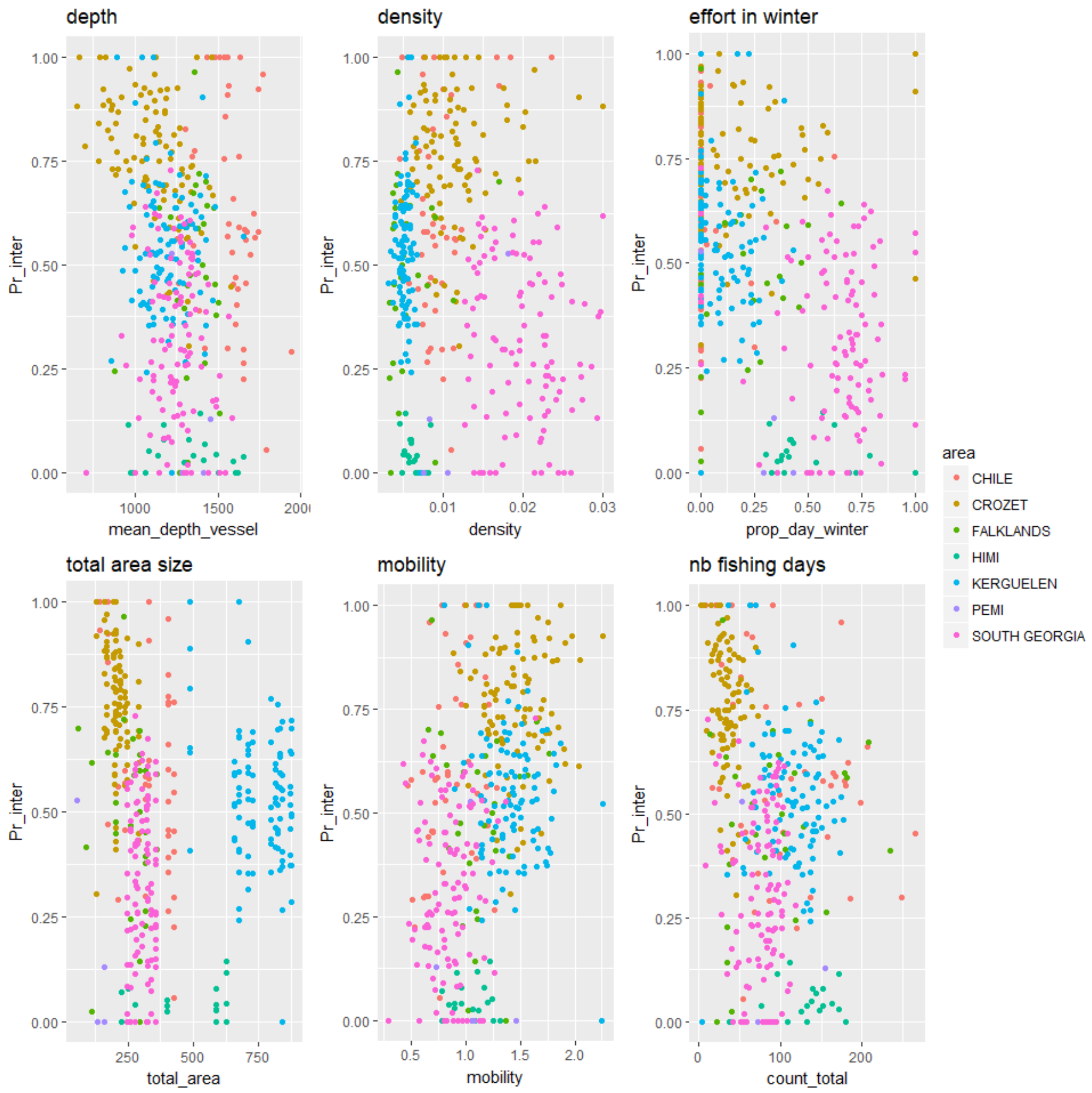

Pairwise plots

```
d1_explo <-droplevels(d1[,
        c(  "mean_depth_vessel","total_area","density", "count_total","mobili
ty","prop_day_winter","prop_day_trotline_cach")])
pairs(d1_explo)
```

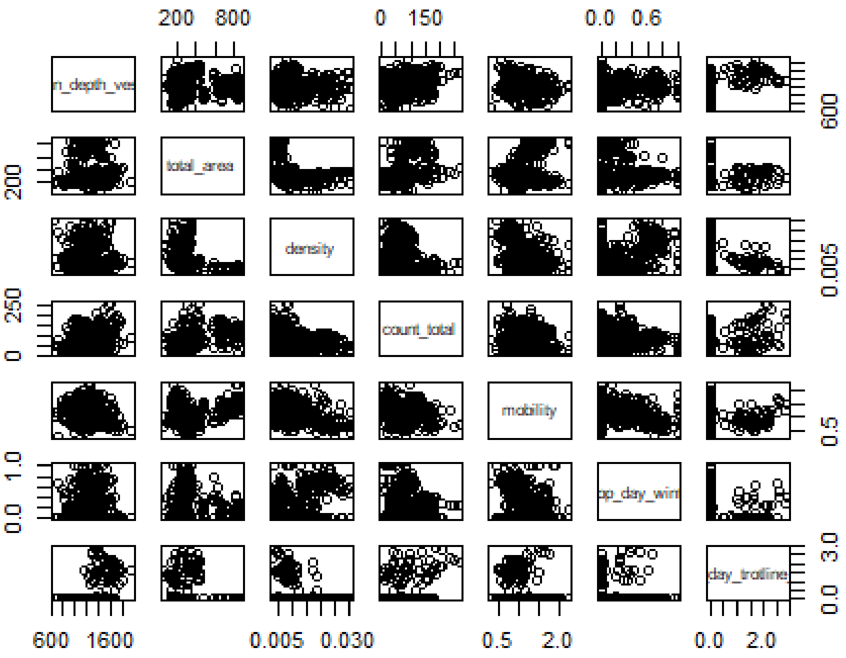

Pearson correlation

Calculate Pearson correlation coefficients for the above data

```
print(cor(d1_explo, method=c("pearson")), digits=4)

##                         mean_depth_vessel total_area  density count_total
## mean_depth_vessel                 1.00000   -0.02004 -0.08112     0.25987
## total_area                       -0.02004    1.00000 -0.47783     0.50976
## density                          -0.08112   -0.47783  1.00000    -0.35101
## count_total                       0.25987    0.50976 -0.35101     1.00000
## mobility                         -0.25118    0.26095 -0.45266    -0.20633
## prop_day_winter                  -0.02605   -0.25759  0.50612     0.01525
## prop_day_trotline_cach            0.48570   -0.17325 -0.17755     0.23666
##                         mobility prop_day_winter prop_day_trotline_cach
## mean_depth_vessel        -0.2512        -0.02605                 0.4857
## total_area                0.2610        -0.25759                -0.1732
## density                  -0.4527         0.50612                -0.1776
## count_total              -0.2063         0.01525                 0.2367
## mobility                  1.0000        -0.39491                -0.2045
## prop_day_winter          -0.3949         1.00000                -0.2176
## prop_day_trotline_cach   -0.2045        -0.21756                 1.0000
```

Re-scale continuous predictors

```
pvars <- c("mean_depth_vessel","prop_day_trotline_cach", "prop_day_winter","t
otal_area","density","mobility" )
```

```r
datsc <- d1
datsc[pvars] <- lapply(datsc[pvars],scale)
```

We fit a glm to the proportion of days with sperm whale interaction using a binomial fmaily and a logit link function. The most pacimonious model is selected using a stepwise forward AIC selection

Null model

```r
glm_null <- glm(cbind(count,count_total - count)~ 1, family = binomial(link = logit), data = datsc)
summary(glm_null)

## 
## Call:
## glm(formula = cbind(count, count_total - count) ~ 1, family = binomial(lin
k = logit), 
##     data = datsc)
## 
## Deviance Residuals: 
##     Min       1Q   Median       3Q      Max  
## -14.949   -2.383    1.284    3.346   14.407  
## 
## Coefficients:
##             Estimate Std. Error z value Pr(>|z|)    
## (Intercept) -0.15045    0.01097  -13.71   <2e-16 ***
## ---
## Signif. codes:  0 '***' 0.001 '**' 0.01 '*' 0.05 '.' 0.1 ' ' 1
## 
## (Dispersion parameter for binomial family taken to be 1)
## 
##     Null deviance: 9307.5  on 407  degrees of freedom
## Residual deviance: 9307.5  on 407  degrees of freedom
## AIC: 10935
## 
## Number of Fisher Scoring iterations: 4
```

Calculate pseudo r squared for the Null model as a check

```r
pseudo_r2(glm_null)

## [1] -4.440892e-16
```

Single term models

Model with depth

```r
glm_1.1 <- glm(cbind(count,count_total - count)~ mean_depth_vessel, family = binomial(link = logit), data = datsc,na.action(na.fail))
summary(glm_1.1)
```

```
##
## Call:
## glm(formula = cbind(count, count_total - count) ~ mean_depth_vessel,
##     family = binomial(link = logit), data = datsc, weights = na.action(na.
fail))
##
## Deviance Residuals:
##     Min       1Q   Median       3Q      Max
## -15.452   -2.347    1.180    3.538   15.640
##
## Coefficients:
##                    Estimate Std. Error z value Pr(>|z|)
## (Intercept)       -0.13770    0.01110 -12.407  < 2e-16 ***
## mean_depth_vessel -0.08690    0.01108  -7.843 4.39e-15 ***
## ---
## Signif. codes:  0 '***' 0.001 '**' 0.01 '*' 0.05 '.' 0.1 ' ' 1
##
## (Dispersion parameter for binomial family taken to be 1)
##
##     Null deviance: 9307.5  on 407  degrees of freedom
## Residual deviance: 9245.8  on 406  degrees of freedom
## AIC: 10875
##
## Number of Fisher Scoring iterations: 4
```

Calculate pseudo r squared

```
pseudo_r2(glm_1.1)

## [1] 0.006630495
```

Model with proportion of sets using trotlines-cachalotera

```
glm_1.2 <- glm(cbind(count,count_total - count)~ prop_day_trotline_cach, fami
ly = binomial(link = logit), data = datsc,na.action(na.fail))
summary(glm_1.2)

##
## Call:
## glm(formula = cbind(count, count_total - count) ~ prop_day_trotline_cach,
##     family = binomial(link = logit), data = datsc, weights = na.action(na.
fail))
##
## Deviance Residuals:
##     Min       1Q   Median       3Q      Max
## -14.539   -2.308    1.215    3.467   12.794
##
## Coefficients:
##                         Estimate Std. Error z value Pr(>|z|)
## (Intercept)            -0.169430   0.011085  -15.29   <2e-16 ***
## prop_day_trotline_cach  0.136531   0.009511   14.36   <2e-16 ***
```

```
## ---
## Signif. codes:  0 '***' 0.001 '**' 0.01 '*' 0.05 '.' 0.1 ' ' 1
##
## (Dispersion parameter for binomial family taken to be 1)
##
##     Null deviance: 9307.5  on 407  degrees of freedom
## Residual deviance: 9099.7  on 406  degrees of freedom
## AIC: 10729
##
## Number of Fisher Scoring iterations: 4
```

Calculate pseudo r squared

```
pseudo_r2(glm_1.2)

## [1] 0.02233636
```

Model with proportion of days in winter

```
glm_1.3 <- glm(cbind(count,count_total - count)~ prop_day_winter, family = bi
nomial(link = logit), data = datsc,na.action(na.fail))
summary(glm_1.3)

##
## Call:
## glm(formula = cbind(count, count_total - count) ~ prop_day_winter,
##     family = binomial(link = logit), data = datsc, weights = na.action(na.
fail))
##
## Deviance Residuals:
##     Min       1Q   Median       3Q      Max
## -13.830   -2.001    0.794    3.137   11.572
##
## Coefficients:
##                 Estimate Std. Error z value Pr(>|z|)
## (Intercept)     -0.16093    0.01131  -14.23   <2e-16 ***
## prop_day_winter -0.52428    0.01255  -41.77   <2e-16 ***
## ---
## Signif. codes:  0 '***' 0.001 '**' 0.01 '*' 0.05 '.' 0.1 ' ' 1
##
## (Dispersion parameter for binomial family taken to be 1)
##
##     Null deviance: 9307.5  on 407  degrees of freedom
## Residual deviance: 7435.9  on 406  degrees of freedom
## AIC: 9065
##
## Number of Fisher Scoring iterations: 4
```

Calculate pseudo r squared

```
pseudo_r2(glm_1.3)
```

```
## [1] 0.2010915
```

Model with total area size

```
glm_1.4 <- glm(cbind(count,count_total - count)~ total_area, family = binomia
l(link = logit), data = datsc,na.action(na.fail))
summary(glm_1.4)
```

```
##
## Call:
## glm(formula = cbind(count, count_total - count) ~ total_area,
##     family = binomial(link = logit), data = datsc, weights = na.action(na.
fail))
##
## Deviance Residuals:
##     Min       1Q   Median       3Q      Max
## -15.145   -2.456    1.222    3.385   14.483
##
## Coefficients:
##             Estimate Std. Error z value Pr(>|z|)
## (Intercept) -0.16510    0.01142 -14.456  < 2e-16 ***
## total_area   0.04943    0.01057   4.676 2.93e-06 ***
## ---
## Signif. codes:  0 '***' 0.001 '**' 0.01 '*' 0.05 '.' 0.1 ' ' 1
##
## (Dispersion parameter for binomial family taken to be 1)
##
##     Null deviance: 9307.5  on 407  degrees of freedom
## Residual deviance: 9285.7  on 406  degrees of freedom
## AIC: 10915
##
## Number of Fisher Scoring iterations: 4
```

Calculate pseudo r squared

```
pseudo_r2(glm_1.4)
```

```
## [1] 0.002348577
```

Model with density of vessels

```
glm_1.5 <- glm(cbind(count,count_total - count)~ density, family = binomial(l
ink = logit), data = datsc,na.action(na.fail))
summary(glm_1.5)
```

```
##
## Call:
## glm(formula = cbind(count, count_total - count) ~ density, family = binomi
al(link = logit),
##     data = datsc, weights = na.action(na.fail))
##
## Deviance Residuals:
```

```
##     Min       1Q   Median       3Q      Max
## -15.923   -2.024    1.276    3.465   13.927
##
## Coefficients:
##             Estimate Std. Error z value Pr(>|z|)
## (Intercept) -0.20011    0.01137  -17.59   <2e-16 ***
## density     -0.23131    0.01191  -19.43   <2e-16 ***
## ---
## Signif. codes:  0 '***' 0.001 '**' 0.01 '*' 0.05 '.' 0.1 ' ' 1
##
## (Dispersion parameter for binomial family taken to be 1)
##
##     Null deviance: 9307.5  on 407  degrees of freedom
## Residual deviance: 8920.7  on 406  degrees of freedom
## AIC: 10550
##
## Number of Fisher Scoring iterations: 4
```

Calculate pseudo r squared

```
pseudo_r2(glm_1.5)

## [1] 0.04156481
```

Model with the fishery

```
glm_1.6 <- glm(cbind(count,count_total - count)~ area, family = binomial(link
= logit), data = datsc,na.action(na.fail))
summary(glm_1.6)

##
## Call:
## glm(formula = cbind(count, count_total - count) ~ area, family = binomial(
link = logit),
##     data = datsc, weights = na.action(na.fail))
##
## Deviance Residuals:
##     Min       1Q   Median       3Q      Max
## -8.4528  -1.9363   0.0895   2.1460  12.0650
##
## Coefficients:
##                     Estimate Std. Error z value Pr(>|z|)
## (Intercept)          0.24757    0.02904   8.524  < 2e-16 ***
## areaCROZET           0.84068    0.04933  17.043  < 2e-16 ***
## areaFALKLANDS       -0.23612    0.04868  -4.851 1.23e-06 ***
## areaHIMI            -3.25983    0.09791 -33.294  < 2e-16 ***
## areaKERGUELEN       -0.09474    0.03456  -2.742  0.00611 **
## areaPEMI            -2.28445    0.15618 -14.627  < 2e-16 ***
## areaSOUTH GEORGIA -1.01897    0.03754 -27.146  < 2e-16 ***
## ---
## Signif. codes:  0 '***' 0.001 '**' 0.01 '*' 0.05 '.' 0.1 ' ' 1
```

```
##
## (Dispersion parameter for binomial family taken to be 1)
##
##     Null deviance: 9307.5  on 407  degrees of freedom
## Residual deviance: 4505.7  on 401  degrees of freedom
## AIC: 6144.8
##
## Number of Fisher Scoring iterations: 5
```

Calculate pseudo r squared

```
pseudo_r2(glm_1.6)

## [1] 0.5159135
```

Model with mobility

```
glm_1.7 <- glm(cbind(count,count_total - count)~ mobility, family = binomial(
link = logit), data = datsc,na.action(na.fail))
summary(glm_1.7)

##
## Call:
## glm(formula = cbind(count, count_total - count) ~ mobility, family = binom
ial(link = logit),
##     data = datsc, weights = na.action(na.fail))
##
## Deviance Residuals:
##      Min        1Q    Median        3Q       Max
## -14.6842   -2.3425    0.6848    3.3608   17.0995
##
## Coefficients:
##             Estimate Std. Error z value Pr(>|z|)
## (Intercept) -0.11249    0.01119  -10.06   <2e-16 ***
## mobility     0.34770    0.01189   29.24   <2e-16 ***
## ---
## Signif. codes:  0 '***' 0.001 '**' 0.01 '*' 0.05 '.' 0.1 ' ' 1
##
## (Dispersion parameter for binomial family taken to be 1)
##
##     Null deviance: 9307.5  on 407  degrees of freedom
## Residual deviance: 8425.6  on 406  degrees of freedom
## AIC: 10055
##
## Number of Fisher Scoring iterations: 4
```

Calculate pseudo r squared

```
pseudo_r2(glm_1.7)

## [1] 0.0947574
```

AIC summary

```
model.sel(list(glm_null, glm_1.1,glm_1.2,glm_1.3,glm_1.4,glm_1.5,glm_1.6,glm_
1.7))
```

```
## Model selection table
##     (Int) men_dpt_vss prp_day_trt_cch prp_day_wnt ttl_are     dns are
## 7  0.2476                                                           +
## 4 -0.1609                                -0.5243
## 8 -0.1125
## 6 -0.2001                                                -0.2313
## 3 -0.1694                      0.1365
## 2 -0.1377     -0.0869
## 5 -0.1651                                        0.04943
## 1 -0.1505
##      mbl  weights df    logLik    AICc   delta weight
## 7        n.a(n.f)  7 -3065.383  6145.0    0.00      1
## 4        n.a(n.f)  2 -4530.494  9065.0 2919.97      0
## 8 0.3477 n.a(n.f)  2 -5025.349 10054.7 3909.68      0
## 6        n.a(n.f)  2 -5272.895 10549.8 4404.77      0
## 3        n.a(n.f)  2 -5362.380 10728.8 4583.74      0
## 2        n.a(n.f)  2 -5435.472 10875.0 4729.93      0
## 5        n.a(n.f)  2 -5455.399 10914.8 4769.78      0
## 1                  1 -5466.329 10934.7 4789.62      0
## Abbreviations:
## weights: n.a(n.f) = 'na.action(na.fail)'
## Models ranked by AICc(x)
```

Model with the lowest AIC

```
glm_2.0 <- glm(cbind(count,count_total - count)~ area, family = binomial(link
= logit), data = datsc)
summary(glm_2.0)
```

```
##
## Call:
## glm(formula = cbind(count, count_total - count) ~ area, family = binomial(
link = logit),
##     data = datsc)
##
## Deviance Residuals:
##     Min       1Q   Median       3Q      Max
## -8.4528  -1.9363   0.0895   2.1460  12.0650
##
## Coefficients:
##                     Estimate Std. Error z value Pr(>|z|)
## (Intercept)          0.24757    0.02904   8.524  < 2e-16 ***
## areaCROZET           0.84068    0.04933  17.043  < 2e-16 ***
## areaFALKLANDS       -0.23612    0.04868  -4.851 1.23e-06 ***
## areaHIMI            -3.25983    0.09791 -33.294  < 2e-16 ***
## areaKERGUELEN       -0.09474    0.03456  -2.742  0.00611 **
```

```
## areaPEMI          -2.28445    0.15618 -14.627  < 2e-16 ***
## areaSOUTH GEORGIA -1.01897    0.03754 -27.146  < 2e-16 ***
## ---
## Signif. codes:  0 '***' 0.001 '**' 0.01 '*' 0.05 '.' 0.1 ' ' 1
##
## (Dispersion parameter for binomial family taken to be 1)
##
##     Null deviance: 9307.5  on 407  degrees of freedom
## Residual deviance: 4505.7  on 401  degrees of freedom
## AIC: 6144.8
##
## Number of Fisher Scoring iterations: 5
```

Calculate pseudo r squared

```
pseudo_r2(glm_2.0)

## [1] 0.5159135
```

Two terms models

Model with depth

```
glm_2.1 <- glm(cbind(count,count_total - count)~ area+ mean_depth_vessel, fam
ily = binomial(link = logit), data = datsc)
summary(glm_2.1)

##
## Call:
## glm(formula = cbind(count, count_total - count) ~ area + mean_depth_vessel
,
##     family = binomial(link = logit), data = datsc)
##
## Deviance Residuals:
##     Min       1Q   Median       3Q      Max
## -8.5742  -2.0007   0.0543   2.1265  12.4004
##
## Coefficients:
##                   Estimate Std. Error z value Pr(>|z|)
## (Intercept)        0.37090    0.04149   8.938  < 2e-16 ***
## areaCROZET         0.67198    0.06369  10.550  < 2e-16 ***
## areaFALKLANDS     -0.32045    0.05274  -6.076 1.23e-09 ***
## areaHIMI          -3.35779    0.10085 -33.295  < 2e-16 ***
## areaKERGUELEN     -0.24003    0.04911  -4.888 1.02e-06 ***
## areaPEMI          -2.36615    0.15743 -15.030  < 2e-16 ***
## areaSOUTH GEORGIA -1.14400    0.04814 -23.764  < 2e-16 ***
## mean_depth_vessel -0.07287    0.01749  -4.166 3.10e-05 ***
## ---
## Signif. codes:  0 '***' 0.001 '**' 0.01 '*' 0.05 '.' 0.1 ' ' 1
##
## (Dispersion parameter for binomial family taken to be 1)
```

```
##
##     Null deviance: 9307.5  on 407  degrees of freedom
## Residual deviance: 4488.3  on 400  degrees of freedom
## AIC: 6129.4
##
## Number of Fisher Scoring iterations: 5
```

pseudo_r2(glm_2.1)

Model with proportion of sets using trotlines-cachalotera

```
glm_2.2 <- glm(cbind(count,count_total - count)~ area+ prop_day_trotline_cach
, family = binomial(link = logit), data = datsc)
summary(glm_2.2)
```

```
##
## Call:
## glm(formula = cbind(count, count_total - count) ~ area + prop_day_trotline
_cach,
##     family = binomial(link = logit), data = datsc)
##
## Deviance Residuals:
##     Min       1Q   Median       3Q      Max
## -9.1627  -1.9473   0.1014   2.1460  12.0900
##
## Coefficients:
##                       Estimate Std. Error z value Pr(>|z|)
## (Intercept)           -0.01548    0.04901  -0.316 0.752132
## areaCROZET             1.15267    0.06801  16.948  < 2e-16 ***
## areaFALKLANDS         -0.14892    0.05063  -2.941 0.003269 **
## areaHIMI              -2.94785    0.10853 -27.161  < 2e-16 ***
## areaKERGUELEN          0.21724    0.05820   3.733 0.000189 ***
## areaPEMI              -2.19896    0.15672 -14.031  < 2e-16 ***
## areaSOUTH GEORGIA     -0.70699    0.06001 -11.781  < 2e-16 ***
## prop_day_trotline_cach  0.12135    0.01823   6.657  2.8e-11 ***
## ---
## Signif. codes:  0 '***' 0.001 '**' 0.01 '*' 0.05 '.' 0.1 ' ' 1
##
## (Dispersion parameter for binomial family taken to be 1)
##
##     Null deviance: 9307.5  on 407  degrees of freedom
## Residual deviance: 4461.1  on 400  degrees of freedom
## AIC: 6102.2
##
## Number of Fisher Scoring iterations: 5
```

pseudo_r2(glm_2.2)

Model with proportion of days in winter

```
glm_2.3 <- glm(cbind(count,count_total - count)~ area+ prop_day_winter, famil
y = binomial(link = logit), data = datsc)
summary(glm_2.3)
```

```
##
## Call:
## glm(formula = cbind(count, count_total - count) ~ area + prop_day_winter,
##     family = binomial(link = logit), data = datsc)
##
## Deviance Residuals:
##     Min       1Q   Median       3Q      Max
## -8.6071  -2.0255   0.1493   2.1201  11.9449
##
## Coefficients:
##                     Estimate Std. Error z value Pr(>|z|)
## (Intercept)         0.15370    0.03484   4.412 1.02e-05 ***
## areaCROZET          0.88689    0.05031  17.627  < 2e-16 ***
## areaFALKLANDS      -0.14526    0.05214  -2.786  0.00533 **
## areaHIMI           -3.09557    0.10341 -29.936  < 2e-16 ***
## areaKERGUELEN      -0.07195    0.03488  -2.063  0.03914 *
## areaPEMI           -2.17234    0.15785 -13.762  < 2e-16 ***
## areaSOUTH GEORGIA -0.76892    0.06341 -12.126  < 2e-16 ***
## prop_day_winter    -0.12164    0.02493  -4.878 1.07e-06 ***
## ---
## Signif. codes:  0 '***' 0.001 '**' 0.01 '*' 0.05 '.' 0.1 ' ' 1
##
## (Dispersion parameter for binomial family taken to be 1)
##
##     Null deviance: 9307.5  on 407  degrees of freedom
## Residual deviance: 4482.0  on 400  degrees of freedom
## AIC: 6123.1
##
## Number of Fisher Scoring iterations: 5
```

pseudo_r2(glm_2.3)

Model with total area size

```
glm_2.4 <- glm(cbind(count,count_total - count)~ area+ total_area, family = b
inomial(link = logit), data = datsc)
summary(glm_2.4)
```

```
##
## Call:
## glm(formula = cbind(count, count_total - count) ~ area + total_area,
##     family = binomial(link = logit), data = datsc)
##
## Deviance Residuals:
##     Min       1Q   Median       3Q      Max
## -8.8521  -1.9078   0.1274   2.0896  12.6429
##
```

```
## Coefficients:
##                    Estimate Std. Error z value Pr(>|z|)    
## (Intercept)         0.16474    0.02992   5.507 3.65e-08 ***
## areaCROZET          0.59423    0.05363  11.081  < 2e-16 ***
## areaFALKLANDS      -0.39923    0.05075  -7.867 3.63e-15 ***
## areaHIMI           -3.09854    0.09864 -31.412  < 2e-16 ***
## areaKERGUELEN       0.63698    0.07090   8.984  < 2e-16 ***
## areaPEMI           -2.66782    0.15961 -16.714  < 2e-16 ***
## areaSOUTH GEORGIA -1.10324    0.03832 -28.794  < 2e-16 ***
## total_area         -0.40831    0.03452 -11.829  < 2e-16 ***
## ---
## Signif. codes:  0 '***' 0.001 '**' 0.01 '*' 0.05 '.' 0.1 ' ' 1
## 
## (Dispersion parameter for binomial family taken to be 1)
## 
##     Null deviance: 9307.5  on 407  degrees of freedom
## Residual deviance: 4363.9  on 400  degrees of freedom
## AIC: 6005
## 
## Number of Fisher Scoring iterations: 5
```

pseudo_r2(glm_2.4)

Model with density of vessels

```
glm_2.5 <- glm(cbind(count,count_total - count)~ area+ density, family = bino
mial(link = logit), data = datsc)
summary(glm_2.5)
```

```
## 
## Call:
## glm(formula = cbind(count, count_total - count) ~ area + density, 
##     family = binomial(link = logit), data = datsc)
## 
## Deviance Residuals: 
##     Min       1Q   Median       3Q      Max  
## -8.5518  -1.9615   0.0987   2.1885  12.0298  
## 
## Coefficients:
##                    Estimate Std. Error z value Pr(>|z|)    
## (Intercept)         0.23484    0.03101   7.572 3.68e-14 ***
## areaCROZET          0.85346    0.05054  16.887  < 2e-16 ***
## areaFALKLANDS      -0.25288    0.05075  -4.983 6.25e-07 ***
## areaHIMI           -3.27335    0.09859 -33.201  < 2e-16 ***
## areaKERGUELEN      -0.11259    0.03778  -2.980  0.00288 ** 
## areaPEMI           -2.27940    0.15624 -14.589  < 2e-16 ***
## areaSOUTH GEORGIA -0.96570    0.05896 -16.380  < 2e-16 ***
## density            -0.03363    0.02874  -1.170  0.24203    
## ---
## Signif. codes:  0 '***' 0.001 '**' 0.01 '*' 0.05 '.' 0.1 ' ' 1
## 
```

```
## (Dispersion parameter for binomial family taken to be 1)
##
##     Null deviance: 9307.5  on 407  degrees of freedom
## Residual deviance: 4504.3  on 400  degrees of freedom
## AIC: 6145.4
##
## Number of Fisher Scoring iterations: 5
```

pseudo_r2(glm_2.5)

Model with mobility

```
glm_2.6 <- glm(cbind(count,count_total - count)~ area+ mobility, family = bin
omial(link = logit), data = datsc)
summary(glm_2.6)
```

```
##
## Call:
## glm(formula = cbind(count, count_total - count) ~ area + mobility,
##     family = binomial(link = logit), data = datsc)
##
## Deviance Residuals:
##     Min       1Q   Median       3Q      Max
## -8.5824  -1.9982   0.0294   2.1118  12.2436
##
## Coefficients:
##                    Estimate Std. Error z value Pr(>|z|)
## (Intercept)         0.35586    0.03594   9.900  < 2e-16 ***
## areaCROZET          0.65997    0.06052  10.905  < 2e-16 ***
## areaFALKLANDS      -0.34810    0.05339  -6.520 7.05e-11 ***
## areaHIMI           -3.32976    0.09889 -33.670  < 2e-16 ***
## areaKERGUELEN      -0.26644    0.04814  -5.535 3.12e-08 ***
## areaPEMI           -2.34474    0.15669 -14.964  < 2e-16 ***
## areaSOUTH GEORGIA -1.03671    0.03772 -27.483  < 2e-16 ***
## mobility            0.10089    0.01968   5.126 2.96e-07 ***
## ---
## Signif. codes:  0 '***' 0.001 '**' 0.01 '*' 0.05 '.' 0.1 ' ' 1
##
## (Dispersion parameter for binomial family taken to be 1)
##
##     Null deviance: 9307.5  on 407  degrees of freedom
## Residual deviance: 4479.3  on 400  degrees of freedom
## AIC: 6120.4
##
## Number of Fisher Scoring iterations: 5
```

pseudo_r2(glm_2.6)

AIC summary

```
model.sel(list(glm_2.0,glm_2.1,glm_2.2,glm_2.3,glm_2.4,glm_2.5,glm_2.6))
```

```
## Model selection table
##      (Int) are men_dpt_vss prp_day_trt_cch prp_day_wnt ttl_are      dns
## 5  0.16470   +                                         -0.4083
## 3 -0.01548   +                      0.1213
## 7  0.35590   +
## 4  0.15370   +                                -0.1216
## 2  0.37090   +    -0.07287
## 1  0.24760   +
## 6  0.23480   +                                                 -0.03363
##      mbl df    logLik   AICc  delta weight
## 5         8 -2994.500 6005.4   0.00      1
## 3         8 -3043.079 6102.5  97.16      0
## 7 0.1009  8 -3052.214 6120.8 115.43      0
## 4         8 -3053.545 6123.5 118.09      0
## 2         8 -3056.701 6129.8 124.40      0
## 1         7 -3065.383 6145.0 139.69      0
## 6         8 -3064.699 6145.8 140.40      0
## Models ranked by AICc(x)
```

Model with the lowest AIC

```
glm_3.0 <- glm(cbind(count,count_total - count)~ area+ total_area, family = b
inomial(link = logit), data = datsc)
summary(glm_3.0)
```

```
##
## Call:
## glm(formula = cbind(count, count_total - count) ~ area + total_area,
##     family = binomial(link = logit), data = datsc)
##
## Deviance Residuals:
##     Min       1Q   Median       3Q      Max
## -8.8521  -1.9078   0.1274   2.0896  12.6429
##
## Coefficients:
##                    Estimate Std. Error z value Pr(>|z|)
## (Intercept)         0.16474    0.02992   5.507 3.65e-08 ***
## areaCROZET          0.59423    0.05363  11.081  < 2e-16 ***
## areaFALKLANDS      -0.39923    0.05075  -7.867 3.63e-15 ***
## areaHIMI           -3.09854    0.09864 -31.412  < 2e-16 ***
## areaKERGUELEN       0.63698    0.07090   8.984  < 2e-16 ***
## areaPEMI           -2.66782    0.15961 -16.714  < 2e-16 ***
## areaSOUTH GEORGIA -1.10324    0.03832 -28.794  < 2e-16 ***
## total_area         -0.40831    0.03452 -11.829  < 2e-16 ***
## ---
## Signif. codes:  0 '***' 0.001 '**' 0.01 '*' 0.05 '.' 0.1 ' ' 1
##
## (Dispersion parameter for binomial family taken to be 1)
##
##     Null deviance: 9307.5  on 407  degrees of freedom
```

```
## Residual deviance: 4363.9  on 400  degrees of freedom
## AIC: 6005
##
## Number of Fisher Scoring iterations: 5
```

pseudo_r2(glm_3.0)

Three terms models

Model with depth

```
glm_3.1 <- glm(cbind(count,count_total - count)~ area+ total_area+ mean_depth
_vessel, family = binomial(link = logit), data = datsc)
summary(glm_3.1)
```

```
##
## Call:
## glm(formula = cbind(count, count_total - count) ~ area + total_area +
##     mean_depth_vessel, family = binomial(link = logit), data = datsc)
##
## Deviance Residuals:
##     Min       1Q   Median       3Q      Max
## -8.8996  -1.9184   0.0888   2.0816  12.7921
##
## Coefficients:
##                     Estimate Std. Error z value Pr(>|z|)
## (Intercept)          0.22803    0.04341   5.253 1.50e-07 ***
## areaCROZET           0.51882    0.06537   7.937 2.07e-15 ***
## areaFALKLANDS       -0.43562    0.05388  -8.085 6.22e-16 ***
## areaHIMI            -3.14907    0.10189 -30.908  < 2e-16 ***
## areaKERGUELEN        0.54263    0.08499   6.384 1.72e-10 ***
## areaPEMI            -2.69633    0.16026 -16.824  < 2e-16 ***
## areaSOUTH GEORGIA -1.16192    0.04819 -24.114  < 2e-16 ***
## total_area          -0.39552    0.03510 -11.268  < 2e-16 ***
## mean_depth_vessel -0.03590    0.01784  -2.013   0.0441 *
## ---
## Signif. codes:  0 '***' 0.001 '**' 0.01 '*' 0.05 '.' 0.1 ' ' 1
##
## (Dispersion parameter for binomial family taken to be 1)
##
##     Null deviance: 9307.5  on 407  degrees of freedom
## Residual deviance: 4359.8  on 399  degrees of freedom
## AIC: 6003
##
## Number of Fisher Scoring iterations: 5
```

pseudo_r2(glm_3.1)

Model with proportion of sets using trotlines-cachalotera

```
glm_3.2 <- glm(cbind(count,count_total - count)~ area+ total_area+ prop_day_t
rotline_cach, family = binomial(link = logit), data = datsc)
summary(glm_3.2)
```

```
##
## Call:
## glm(formula = cbind(count, count_total - count) ~ area + total_area +
##     prop_day_trotline_cach, family = binomial(link = logit),
##     data = datsc)
##
## Deviance Residuals:
##     Min       1Q   Median       3Q      Max
## -9.6537  -1.8949   0.1323   2.0816  12.6726
##
## Coefficients:
##                        Estimate Std. Error z value Pr(>|z|)
## (Intercept)            -0.10226    0.04968  -2.058   0.0396 *
## areaCROZET              0.90982    0.07116  12.785  < 2e-16 ***
## areaFALKLANDS          -0.31122    0.05261  -5.916 3.30e-09 ***
## areaHIMI               -2.78188    0.10930 -25.453  < 2e-16 ***
## areaKERGUELEN           0.95544    0.08536  11.193  < 2e-16 ***
## areaPEMI               -2.58284    0.16013 -16.130  < 2e-16 ***
## areaSOUTH GEORGIA      -0.78718    0.06057 -12.997  < 2e-16 ***
## total_area             -0.40951    0.03451 -11.865  < 2e-16 ***
## prop_day_trotline_cach  0.12287    0.01828   6.720 1.81e-11 ***
## ---
## Signif. codes:  0 '***' 0.001 '**' 0.01 '*' 0.05 '.' 0.1 ' ' 1
##
## (Dispersion parameter for binomial family taken to be 1)
##
##     Null deviance: 9307.5  on 407  degrees of freedom
## Residual deviance: 4318.5  on 399  degrees of freedom
## AIC: 5961.6
##
## Number of Fisher Scoring iterations: 5
```

pseudo_r2(glm_3.2)

Model with mobility

```
glm_3.3 <- glm(cbind(count,count_total - count)~ area+ total_area+mobility, f
amily = binomial(link = logit), data = datsc)
summary(glm_3.3)
```

```
##
## Call:
## glm(formula = cbind(count, count_total - count) ~ area + total_area +
##     mobility, family = binomial(link = logit), data = datsc)
##
## Deviance Residuals:
##     Min       1Q   Median       3Q      Max
```

```
## -8.9195  -1.8202   0.0851   2.0043  13.0141
##
## Coefficients:
##                     Estimate Std. Error z value Pr(>|z|)
## (Intercept)          0.32495    0.03620   8.976  < 2e-16 ***
## areaCROZET           0.27085    0.06740   4.019 5.86e-05 ***
## areaFALKLANDS       -0.60256    0.05703 -10.566  < 2e-16 ***
## areaHIMI            -3.18993    0.09936 -32.105  < 2e-16 ***
## areaKERGUELEN        0.47401    0.07388   6.416 1.40e-10 ***
## areaPEMI            -2.82594    0.16117 -17.534  < 2e-16 ***
## areaSOUTH GEORGIA -1.14616    0.03883 -29.516  < 2e-16 ***
## total_area          -0.47007    0.03552 -13.233  < 2e-16 ***
## mobility             0.16015    0.02027   7.900 2.79e-15 ***
## ---
## Signif. codes:  0 '***' 0.001 '**' 0.01 '*' 0.05 '.' 0.1 ' ' 1
##
## (Dispersion parameter for binomial family taken to be 1)
##
##     Null deviance: 9307.5  on 407  degrees of freedom
## Residual deviance: 4301.2  on 399  degrees of freedom
## AIC: 5944.3
##
## Number of Fisher Scoring iterations: 5
```

pseudo_r2(glm_3.3)

Model with density of vessels

```
glm_3.4 <- glm(cbind(count,count_total - count)~ area+ total_area+ density, f
amily = binomial(link = logit), data = datsc)
summary(glm_3.4)
```

```
##
## Call:
## glm(formula = cbind(count, count_total - count) ~ area + total_area +
##     density, family = binomial(link = logit), data = datsc)
##
## Deviance Residuals:
##     Min       1Q   Median       3Q      Max
## -8.8941  -1.8670   0.1151   2.0543  12.5781
##
## Coefficients:
##                     Estimate Std. Error z value Pr(>|z|)
## (Intercept)          0.13262    0.03223   4.115 3.88e-05 ***
## areaCROZET           0.61721    0.05433  11.361  < 2e-16 ***
## areaFALKLANDS       -0.44259    0.05325  -8.311  < 2e-16 ***
## areaHIMI            -3.12463    0.09912 -31.523  < 2e-16 ***
## areaKERGUELEN        0.61662    0.07132   8.646  < 2e-16 ***
## areaPEMI            -2.66553    0.15956 -16.705  < 2e-16 ***
## areaSOUTH GEORGIA -0.98277    0.05896 -16.667  < 2e-16 ***
## total_area          -0.41986    0.03480 -12.067  < 2e-16 ***
```

```
## density           -0.07793    0.02912  -2.676  0.00745 **
## ---
## Signif. codes:  0 '***' 0.001 '**' 0.01 '*' 0.05 '.' 0.1 ' ' 1
##
## (Dispersion parameter for binomial family taken to be 1)
##
##     Null deviance: 9307.5  on 407  degrees of freedom
## Residual deviance: 4356.7  on 399  degrees of freedom
## AIC: 5999.8
##
## Number of Fisher Scoring iterations: 5
```

pseudo_r2(glm_3.4)

Model with proportion of days in winter

```
glm_3.5 <- glm(cbind(count,count_total - count)~ area+ total_area+ prop_day_w
inter, family = binomial(link = logit), data = datsc)
summary(glm_3.5)
```

```
##
## Call:
## glm(formula = cbind(count, count_total - count) ~ area + total_area +
##     prop_day_winter, family = binomial(link = logit), data = datsc)
##
## Deviance Residuals:
##    Min      1Q  Median      3Q     Max
## -8.711  -1.964   0.174   2.142  12.520
##
## Coefficients:
##                    Estimate Std. Error z value Pr(>|z|)
## (Intercept)         0.07006    0.03557   1.969   0.0489 *
## areaCROZET          0.64051    0.05453  11.747  < 2e-16 ***
## areaFALKLANDS      -0.30781    0.05406  -5.694 1.24e-08 ***
## areaHIMI           -2.92428    0.10469 -27.932  < 2e-16 ***
## areaKERGUELEN       0.66042    0.07098   9.304  < 2e-16 ***
## areaPEMI           -2.55722    0.16126 -15.858  < 2e-16 ***
## areaSOUTH GEORGIA -0.85132    0.06382 -13.340  < 2e-16 ***
## total_area         -0.40874    0.03449 -11.852  < 2e-16 ***
## prop_day_winter    -0.12279    0.02494  -4.923 8.52e-07 ***
## ---
## Signif. codes:  0 '***' 0.001 '**' 0.01 '*' 0.05 '.' 0.1 ' ' 1
##
## (Dispersion parameter for binomial family taken to be 1)
##
##     Null deviance: 9307.5  on 407  degrees of freedom
## Residual deviance: 4339.8  on 399  degrees of freedom
## AIC: 5982.9
##
## Number of Fisher Scoring iterations: 5
```

pseudo_r2(glm_3.5)

AIC summary

```
model.sel(list(glm_3.0, glm_3.1,glm_3.2,glm_3.3,glm_3.4,glm_3.5))

## Model selection table
##      (Int) are ttl_are men_dpt_vss prp_day_trt_cch    mbl      dns
## 4  0.32490   + -0.4701                                0.1601
## 3 -0.10230   + -0.4095                         0.1229
## 6  0.07006   + -0.4087
## 5  0.13260   + -0.4199                                       -0.07793
## 2  0.22800   + -0.3955     -0.0359
## 1  0.16470   + -0.4083
##   prp_day_wnt df    logLik   AICc delta weight
## 4              9 -2963.145 5944.7  0.00      1
## 3              9 -2971.781 5962.0 17.27      0
## 6     -0.1228  9 -2982.440 5983.3 38.59      0
## 5              9 -2990.923 6000.3 55.56      0
## 2              9 -2992.475 6003.4 58.66      0
## 1              8 -2994.500 6005.4 60.62      0
## Models ranked by AICc(x)
```

Model with the lowest AIC

```
glm_4.0 <- glm(cbind(count,count_total - count)~ area+ total_area+mobility, f
amily = binomial(link = logit), data = datsc)
summary(glm_4.0)

##
## Call:
## glm(formula = cbind(count, count_total - count) ~ area + total_area +
##     mobility, family = binomial(link = logit), data = datsc)
##
## Deviance Residuals:
##     Min       1Q   Median       3Q      Max
## -8.9195  -1.8202   0.0851   2.0043  13.0141
##
## Coefficients:
##                     Estimate Std. Error z value Pr(>|z|)
## (Intercept)          0.32495    0.03620   8.976  < 2e-16 ***
## areaCROZET           0.27085    0.06740   4.019 5.86e-05 ***
## areaFALKLANDS       -0.60256    0.05703 -10.566  < 2e-16 ***
## areaHIMI            -3.18993    0.09936 -32.105  < 2e-16 ***
## areaKERGUELEN        0.47401    0.07388   6.416 1.40e-10 ***
## areaPEMI            -2.82594    0.16117 -17.534  < 2e-16 ***
## areaSOUTH GEORGIA -1.14616    0.03883 -29.516  < 2e-16 ***
## total_area          -0.47007    0.03552 -13.233  < 2e-16 ***
## mobility             0.16015    0.02027   7.900 2.79e-15 ***
## ---
## Signif. codes:  0 '***' 0.001 '**' 0.01 '*' 0.05 '.' 0.1 ' ' 1
```

```
##
## (Dispersion parameter for binomial family taken to be 1)
##
##     Null deviance: 9307.5  on 407  degrees of freedom
## Residual deviance: 4301.2  on 399  degrees of freedom
## AIC: 5944.3
##
## Number of Fisher Scoring iterations: 5
```

pseudo_r2(glm_4.0)

Four terms models

Model with depth

```
glm_4.1 <- glm(cbind(count,count_total - count)~ area+ total_area+mobility+me
an_depth_vessel, family = binomial(link = logit), data = datsc)
summary(glm_4.1)
```

```
##
## Call:
## glm(formula = cbind(count, count_total - count) ~ area + total_area +
##     mobility + mean_depth_vessel, family = binomial(link = logit),
##     data = datsc)
##
## Deviance Residuals:
##     Min       1Q   Median       3Q      Max
## -8.8451  -1.8491   0.1063   2.0524  13.2487
##
## Coefficients:
##                   Estimate Std. Error z value Pr(>|z|)
## (Intercept)        0.42420    0.04973   8.529  < 2e-16 ***
## areaCROZET         0.14683    0.07968   1.843 0.065370 .
## areaFALKLANDS     -0.66429    0.06087 -10.913  < 2e-16 ***
## areaHIMI          -3.26719    0.10297 -31.729  < 2e-16 ***
## areaKERGUELEN      0.32915    0.08906   3.696 0.000219 ***
## areaPEMI          -2.87587    0.16219 -17.731  < 2e-16 ***
## areaSOUTH GEORGIA -1.23350    0.04910 -25.120  < 2e-16 ***
## total_area        -0.45390    0.03596 -12.621  < 2e-16 ***
## mobility           0.16677    0.02040   8.175 2.96e-16 ***
## mean_depth_vessel -0.05248    0.01800  -2.916 0.003544 **
## ---
## Signif. codes:  0 '***' 0.001 '**' 0.01 '*' 0.05 '.' 0.1 ' ' 1
##
## (Dispersion parameter for binomial family taken to be 1)
##
##     Null deviance: 9307.5  on 407  degrees of freedom
## Residual deviance: 4292.7  on 398  degrees of freedom
## AIC: 5937.8
##
## Number of Fisher Scoring iterations: 5
```

pseudo_r2(glm_4.1)

Model with proportion of sets using trotlines-cachalotera

```
glm_4.2 <- glm(cbind(count,count_total - count)~ area+ total_area+mobility+pr
op_day_trotline_cach, family = binomial(link = logit), data = datsc)
summary(glm_4.2)
```

```
##
## Call:
## glm(formula = cbind(count, count_total - count) ~ area + total_area +
##     mobility + prop_day_trotline_cach, family = binomial(link = logit),
##     data = datsc)
##
## Deviance Residuals:
##     Min       1Q   Median       3Q      Max
## -9.2185  -1.8556   0.1335   1.9677  12.9967
##
## Coefficients:
##                        Estimate Std. Error z value Pr(>|z|)
## (Intercept)             0.08397    0.05673   1.480    0.139
## areaCROZET              0.57137    0.08655   6.601 4.07e-11 ***
## areaFALKLANDS          -0.50460    0.05979  -8.439  < 2e-16 ***
## areaHIMI               -2.91546    0.11114 -26.231  < 2e-16 ***
## areaKERGUELEN           0.75723    0.09013   8.401  < 2e-16 ***
## areaPEMI               -2.73599    0.16193 -16.896  < 2e-16 ***
## areaSOUTH GEORGIA      -0.87773    0.06226 -14.098  < 2e-16 ***
## total_area             -0.46333    0.03549 -13.055  < 2e-16 ***
## mobility                0.14144    0.02055   6.884 5.83e-12 ***
## prop_day_trotline_cach  0.10218    0.01860   5.494 3.94e-08 ***
## ---
## Signif. codes:  0 '***' 0.001 '**' 0.01 '*' 0.05 '.' 0.1 ' ' 1
##
## (Dispersion parameter for binomial family taken to be 1)
##
##     Null deviance: 9307.5  on 407  degrees of freedom
## Residual deviance: 4270.9  on 398  degrees of freedom
## AIC: 5916
##
## Number of Fisher Scoring iterations: 5
```

pseudo_r2(glm_4.2)

Model with density of vessels

```
glm_4.3 <- glm(cbind(count,count_total - count)~ area+ total_area+mobility+de
nsity, family = binomial(link = logit), data = datsc)
summary(glm_4.3)
```

```
##
## Call:
```

```
## glm(formula = cbind(count, count_total - count) ~ area + total_area +
##     mobility + density, family = binomial(link = logit), data = datsc)
##
## Deviance Residuals:
##     Min       1Q   Median       3Q      Max
## -8.9843  -1.8292   0.1039   1.9767  12.9767
##
## Coefficients:
##                    Estimate Std. Error z value Pr(>|z|)
## (Intercept)         0.30697    0.03982   7.709 1.27e-14 ***
## areaCROZET          0.28936    0.06955   4.160 3.18e-05 ***
## areaFALKLANDS      -0.61456    0.05808 -10.581  < 2e-16 ***
## areaHIMI           -3.19808    0.09964 -32.096  < 2e-16 ***
## areaKERGUELEN       0.47023    0.07397   6.357 2.06e-10 ***
## areaPEMI           -2.81997    0.16122 -17.492  < 2e-16 ***
## areaSOUTH GEORGIA -1.09511    0.06105 -17.938  < 2e-16 ***
## total_area         -0.47305    0.03563 -13.278  < 2e-16 ***
## mobility            0.15556    0.02071   7.513 5.79e-14 ***
## density            -0.03231    0.02986  -1.082    0.279
## ---
## Signif. codes:  0 '***' 0.001 '**' 0.01 '*' 0.05 '.' 0.1 ' ' 1
##
## (Dispersion parameter for binomial family taken to be 1)
##
##     Null deviance: 9307.5  on 407  degrees of freedom
## Residual deviance: 4300.0  on 398  degrees of freedom
## AIC: 5945.1
##
## Number of Fisher Scoring iterations: 5
```

pseudo_r2(glm_4.3)

Model with proportion of days in winter

```
glm_4.4 <- glm(cbind(count,count_total - count)~ area+ total_area+mobility+pr
op_day_winter, family = binomial(link = logit), data = datsc)
summary(glm_4.4)
```

```
##
## Call:
## glm(formula = cbind(count, count_total - count) ~ area + total_area +
##     mobility + prop_day_winter, family = binomial(link = logit),
##     data = datsc)
##
## Deviance Residuals:
##     Min       1Q   Median       3Q      Max
## -9.1222  -1.8787   0.1287   2.0688  12.8882
##
## Coefficients:
##                    Estimate Std. Error z value Pr(>|z|)
## (Intercept)         0.23418    0.04171   5.615 1.96e-08 ***
```

```
## areaCROZET         0.32437    0.06857   4.731 2.24e-06 ***
## areaFALKLANDS     -0.51316    0.06056  -8.474  < 2e-16 ***
## areaHIMI          -3.02882    0.10580 -28.629  < 2e-16 ***
## areaKERGUELEN      0.50106    0.07407   6.764 1.34e-11 ***
## areaPEMI          -2.71982    0.16298 -16.688  < 2e-16 ***
## areaSOUTH GEORGIA -0.91962    0.06457 -14.242  < 2e-16 ***
## total_area        -0.46801    0.03549 -13.188  < 2e-16 ***
## mobility           0.15400    0.02032   7.578 3.51e-14 ***
## prop_day_winter   -0.10984    0.02507  -4.381 1.18e-05 ***
## ---
## Signif. codes:  0 '***' 0.001 '**' 0.01 '*' 0.05 '.' 0.1 ' ' 1
##
## (Dispersion parameter for binomial family taken to be 1)
##
##     Null deviance: 9307.5  on 407  degrees of freedom
## Residual deviance: 4282.1  on 398  degrees of freedom
## AIC: 5927.2
##
## Number of Fisher Scoring iterations: 5
```

pseudo_r2(glm_4.4)

AIC summary

```
model.sel(list(glm_4.0,glm_4.1,glm_4.2,glm_4.3,glm_4.4))

## Model selection table
##     (Int) are    mbl ttl_are men_dpt_vss prp_day_trt_cch      dns
## 3 0.08397   + 0.1414 -0.4633                      0.1022
## 5 0.23420   + 0.1540 -0.4680
## 2 0.42420   + 0.1668 -0.4539    -0.05248
## 1 0.32490   + 0.1601 -0.4701
## 4 0.30700   + 0.1556 -0.4730                                -0.03231
##   prp_day_wnt df    logLik   AICc delta weight
## 3             10 -2947.994 5916.5  0.00  0.996
## 5     -0.1098 10 -2953.598 5927.8 11.21  0.004
## 2             10 -2958.893 5938.3 21.80  0.000
## 1              9 -2963.145 5944.7 28.20  0.000
## 4             10 -2962.560 5945.7 29.13  0.000
## Models ranked by AICc(x)
```

Model with the lowest AIC

```
glm_5.0 <- glm(cbind(count,count_total - count)~ area+ total_area+mobility+pr
op_day_trotline_cach, family = binomial(link = logit), data = datsc)
summary(glm_5.0)

##
## Call:
## glm(formula = cbind(count, count_total - count) ~ area + total_area +
##     mobility + prop_day_trotline_cach, family = binomial(link = logit),
```

```
##     data = datsc)
##
## Deviance Residuals:
##     Min       1Q   Median       3Q      Max
## -9.2185  -1.8556   0.1335   1.9677  12.9967
##
## Coefficients:
##                          Estimate Std. Error z value Pr(>|z|)
## (Intercept)               0.08397    0.05673   1.480    0.139
## areaCROZET                0.57137    0.08655   6.601 4.07e-11 ***
## areaFALKLANDS            -0.50460    0.05979  -8.439  < 2e-16 ***
## areaHIMI                 -2.91546    0.11114 -26.231  < 2e-16 ***
## areaKERGUELEN             0.75723    0.09013   8.401  < 2e-16 ***
## areaPEMI                 -2.73599    0.16193 -16.896  < 2e-16 ***
## areaSOUTH GEORGIA        -0.87773    0.06226 -14.098  < 2e-16 ***
## total_area               -0.46333    0.03549 -13.055  < 2e-16 ***
## mobility                  0.14144    0.02055   6.884 5.83e-12 ***
## prop_day_trotline_cach  0.10218    0.01860   5.494 3.94e-08 ***
## ---
## Signif. codes:  0 '***' 0.001 '**' 0.01 '*' 0.05 '.' 0.1 ' ' 1
##
## (Dispersion parameter for binomial family taken to be 1)
##
##     Null deviance: 9307.5  on 407  degrees of freedom
## Residual deviance: 4270.9  on 398  degrees of freedom
## AIC: 5916
##
## Number of Fisher Scoring iterations: 5
```

pseudo_r2(glm_5.0)

Five terms models

Model with depth

```
glm_5.1 <- glm(cbind(count,count_total - count)~ area+ total_area+mobility+pr
op_day_trotline_cach+mean_depth_vessel, family = binomial(link = logit), data
= datsc)
summary(glm_5.1)
```

```
##
## Call:
## glm(formula = cbind(count, count_total - count) ~ area + total_area +
##     mobility + prop_day_trotline_cach + mean_depth_vessel, family = binomi
al(link = logit),
##     data = datsc)
##
## Deviance Residuals:
##     Min       1Q   Median       3Q      Max
## -9.2845  -1.8740   0.1241   2.0427  13.2396
##
```

```
## Coefficients:
##                         Estimate Std. Error z value Pr(>|z|)
## (Intercept)              0.18429    0.06576   2.802  0.00507 **
## areaCROZET               0.44606    0.09596   4.648 3.34e-06 ***
## areaFALKLANDS           -0.56763    0.06336  -8.958  < 2e-16 ***
## areaHIMI                -2.99275    0.11417 -26.212  < 2e-16 ***
## areaKERGUELEN            0.61006    0.10248   5.953 2.63e-09 ***
## areaPEMI                -2.78661    0.16291 -17.105  < 2e-16 ***
## areaSOUTH GEORGIA       -0.96540    0.06873 -14.046  < 2e-16 ***
## total_area              -0.44649    0.03594 -12.425  < 2e-16 ***
## mobility                 0.14815    0.02067   7.169 7.58e-13 ***
## prop_day_trotline_cach   0.10328    0.01861   5.550 2.86e-08 ***
## mean_depth_vessel       -0.05438    0.01800  -3.022  0.00251 **
## ---
## Signif. codes:  0 '***' 0.001 '**' 0.01 '*' 0.05 '.' 0.1 ' ' 1
##
## (Dispersion parameter for binomial family taken to be 1)
##
##     Null deviance: 9307.5  on 407  degrees of freedom
## Residual deviance: 4261.7  on 397  degrees of freedom
## AIC: 5908.9
##
## Number of Fisher Scoring iterations: 5
```

pseudo_r2(glm_5.1)

Model with density of vessels

```
glm_5.2 <- glm(cbind(count,count_total - count)~ area+ total_area+mobility+pr
op_day_trotline_cach+density, family = binomial(link = logit), data = datsc)
summary(glm_5.2)
```

```
##
## Call:
## glm(formula = cbind(count, count_total - count) ~ area + total_area +
##     mobility + prop_day_trotline_cach + density, family = binomial(link =
logit),
##     data = datsc)
##
## Deviance Residuals:
##     Min       1Q   Median       3Q      Max
## -9.2411  -1.8435   0.1512   1.9711  12.9621
##
## Coefficients:
##                         Estimate Std. Error z value Pr(>|z|)
## (Intercept)              0.06825    0.05885   1.160    0.246
## areaCROZET               0.58736    0.08801   6.673 2.50e-11 ***
## areaFALKLANDS           -0.51607    0.06087  -8.479  < 2e-16 ***
## areaHIMI                -2.92391    0.11145 -26.235  < 2e-16 ***
## areaKERGUELEN            0.75284    0.09023   8.344  < 2e-16 ***
## areaPEMI                -2.73089    0.16197 -16.861  < 2e-16 ***
```

```
## areaSOUTH GEORGIA      -0.83159    0.07749 -10.731  < 2e-16 ***
## total_area             -0.46615    0.03560 -13.093  < 2e-16 ***
## mobility                0.13729    0.02096   6.551 5.71e-11 ***
## prop_day_trotline_cach  0.10185    0.01859   5.477 4.31e-08 ***
## density                -0.02977    0.02983  -0.998    0.318
## ---
## Signif. codes:  0 '***' 0.001 '**' 0.01 '*' 0.05 '.' 0.1 ' ' 1
##
## (Dispersion parameter for binomial family taken to be 1)
##
##     Null deviance: 9307.5  on 407  degrees of freedom
## Residual deviance: 4269.9  on 397  degrees of freedom
## AIC: 5917
##
## Number of Fisher Scoring iterations: 5
```

pseudo_r2(glm_5.2)

Model with proportion of days in winter

```
glm_5.3 <- glm(cbind(count,count_total - count)~ area+ total_area+mobility+pr
op_day_trotline_cach+prop_day_winter, family = binomial(link = logit), data =
datsc)
summary(glm_5.3)
```

```
##
## Call:
## glm(formula = cbind(count, count_total - count) ~ area + total_area +
##     mobility + prop_day_trotline_cach + prop_day_winter, family = binomial
(link = logit),
##     data = datsc)
##
## Deviance Residuals:
##     Min       1Q   Median       3Q      Max
## -9.1009  -1.9718   0.1882   2.0733  12.8436
##
## Coefficients:
##                        Estimate Std. Error z value Pr(>|z|)
## (Intercept)            -0.05553    0.06282  -0.884    0.377
## areaCROZET              0.67462    0.08894   7.585 3.33e-14 ***
## areaFALKLANDS          -0.38546    0.06404  -6.019 1.75e-09 ***
## areaHIMI               -2.68845    0.11928 -22.538  < 2e-16 ***
## areaKERGUELEN           0.82607    0.09108   9.070  < 2e-16 ***
## areaPEMI               -2.59805    0.16411 -15.831  < 2e-16 ***
## areaSOUTH GEORGIA      -0.57277    0.08561  -6.690 2.23e-11 ***
## total_area             -0.45992    0.03544 -12.976  < 2e-16 ***
## mobility                0.13149    0.02064   6.371 1.88e-10 ***
## prop_day_trotline_cach  0.11529    0.01877   6.144 8.05e-10 ***
## prop_day_winter        -0.13116    0.02531  -5.182 2.20e-07 ***
## ---
## Signif. codes:  0 '***' 0.001 '**' 0.01 '*' 0.05 '.' 0.1 ' ' 1
```

```
##
## (Dispersion parameter for binomial family taken to be 1)
##
##     Null deviance: 9307.5  on 407  degrees of freedom
## Residual deviance: 4244.2  on 397  degrees of freedom
## AIC: 5891.3
##
## Number of Fisher Scoring iterations: 5
```

pseudo_r2(glm_5.3)

AIC summary

```
model.sel(list(glm_5.0,glm_5.1,glm_5.2,glm_5.3))

## Model selection table
##      (Int) are    mbl prp_day_trt_cch ttl_are men_dpt_vss      dns
## 4 -0.05553   + 0.1315          0.1153 -0.4599
## 2  0.18430   + 0.1482          0.1033 -0.4465    -0.05438
## 1  0.08397   + 0.1414          0.1022 -0.4633
## 3  0.06825   + 0.1373          0.1019 -0.4661             -0.02977
##   prp_day_wnt df    logLik   AICc delta weight
## 4     -0.1312 11 -2934.642 5892.0  0.00      1
## 2             11 -2943.428 5909.5 17.57      0
## 1             10 -2947.994 5916.5 24.59      0
## 3             11 -2947.496 5917.7 25.71      0
## Models ranked by AICc(x)
```

Model with the lowest AIC

```
glm_6.0 <- glm(cbind(count,count_total - count)~ area+ total_area+mobility+pr
op_day_trotline_cach+prop_day_winter, family = binomial(link = logit), data =
datsc)
summary(glm_6.0)

##
## Call:
## glm(formula = cbind(count, count_total - count) ~ area + total_area +
##     mobility + prop_day_trotline_cach + prop_day_winter, family = binomial
(link = logit),
##     data = datsc)
##
## Deviance Residuals:
##     Min       1Q   Median       3Q      Max
## -9.1009  -1.9718   0.1882   2.0733  12.8436
##
## Coefficients:
##                        Estimate Std. Error z value Pr(>|z|)
## (Intercept)            -0.05553    0.06282  -0.884    0.377
## areaCROZET              0.67462    0.08894   7.585 3.33e-14 ***
## areaFALKLANDS          -0.38546    0.06404  -6.019 1.75e-09 ***
```

```
## areaHIMI              -2.68845    0.11928 -22.538  < 2e-16 ***
## areaKERGUELEN          0.82607    0.09108   9.070  < 2e-16 ***
## areaPEMI              -2.59805    0.16411 -15.831  < 2e-16 ***
## areaSOUTH GEORGIA     -0.57277    0.08561  -6.690 2.23e-11 ***
## total_area            -0.45992    0.03544 -12.976  < 2e-16 ***
## mobility               0.13149    0.02064   6.371 1.88e-10 ***
## prop_day_trotline_cach  0.11529    0.01877   6.144 8.05e-10 ***
## prop_day_winter       -0.13116    0.02531  -5.182 2.20e-07 ***
## ---
## Signif. codes:  0 '***' 0.001 '**' 0.01 '*' 0.05 '.' 0.1 ' ' 1
##
## (Dispersion parameter for binomial family taken to be 1)
##
##     Null deviance: 9307.5  on 407  degrees of freedom
## Residual deviance: 4244.2  on 397  degrees of freedom
## AIC: 5891.3
##
## Number of Fisher Scoring iterations: 5
```

pseudo_r2(glm_6.0)

Six terms models

Model with depth

```
glm_6.1 <- glm(cbind(count,count_total - count)~ area+ total_area+mobility+pr
op_day_trotline_cach+prop_day_winter+mean_depth_vessel, family = binomial(lin
k = logit), data = datsc)
summary(glm_6.1)

##
## Call:
## glm(formula = cbind(count, count_total - count) ~ area + total_area +
##     mobility + prop_day_trotline_cach + prop_day_winter + mean_depth_vesse
l,
##     family = binomial(link = logit), data = datsc)
##
## Deviance Residuals:
##     Min       1Q   Median       3Q      Max
## -9.0221  -1.9293   0.1905   1.9752  13.0909
##
## Coefficients:
##                       Estimate Std. Error z value Pr(>|z|)
## (Intercept)            0.04630    0.07091   0.653  0.51379
## areaCROZET             0.54710    0.09800   5.583 2.37e-08 ***
## areaFALKLANDS         -0.44909    0.06728  -6.675 2.47e-11 ***
## areaHIMI              -2.76616    0.12198 -22.677  < 2e-16 ***
## areaKERGUELEN          0.67546    0.10326   6.541 6.10e-11 ***
## areaPEMI              -2.64914    0.16504 -16.051  < 2e-16 ***
## areaSOUTH GEORGIA     -0.66027    0.09015  -7.324 2.40e-13 ***
## total_area            -0.44254    0.03590 -12.328  < 2e-16 ***
```

```
## mobility                0.13826    0.02075   6.662 2.70e-11 ***
## prop_day_trotline_cach  0.11654    0.01878   6.207 5.41e-10 ***
## prop_day_winter        -0.13222    0.02530  -5.226 1.73e-07 ***
## mean_depth_vessel      -0.05584    0.01803  -3.097  0.00195 **
## ---
## Signif. codes:  0 '***' 0.001 '**' 0.01 '*' 0.05 '.' 0.1 ' ' 1
##
## (Dispersion parameter for binomial family taken to be 1)
##
##     Null deviance: 9307.5  on 407  degrees of freedom
## Residual deviance: 4234.6  on 396  degrees of freedom
## AIC: 5883.7
##
## Number of Fisher Scoring iterations: 5
```

pseudo_r2(glm_6.1)

Model with density of vessels

```
glm_6.2 <- glm(cbind(count,count_total - count)~ area+ total_area+mobility+pr
op_day_trotline_cach+prop_day_winter+density, family = binomial(link = logit)
, data = datsc)
summary(glm_6.2)
```

```
##
## Call:
## glm(formula = cbind(count, count_total - count) ~ area + total_area +
##     mobility + prop_day_trotline_cach + prop_day_winter + density,
##     family = binomial(link = logit), data = datsc)
##
## Deviance Residuals:
##     Min       1Q   Median       3Q      Max
## -9.2100  -1.8828   0.2019   2.0480  12.7808
##
## Coefficients:
##                        Estimate Std. Error z value Pr(>|z|)
## (Intercept)            -0.08687    0.06565  -1.323    0.186
## areaCROZET              0.70500    0.09086   7.759 8.57e-15 ***
## areaFALKLANDS          -0.39970    0.06460  -6.187 6.12e-10 ***
## areaHIMI               -2.69381    0.11929 -22.582  < 2e-16 ***
## areaKERGUELEN           0.82147    0.09110   9.017  < 2e-16 ***
## areaPEMI               -2.58325    0.16429 -15.724  < 2e-16 ***
## areaSOUTH GEORGIA      -0.48499    0.10102  -4.801 1.58e-06 ***
## total_area             -0.46439    0.03555 -13.064  < 2e-16 ***
## mobility                0.12426    0.02110   5.890 3.87e-09 ***
## prop_day_trotline_cach  0.11528    0.01875   6.147 7.91e-10 ***
## prop_day_winter        -0.13625    0.02551  -5.341 9.27e-08 ***
## density                -0.04904    0.03003  -1.633    0.103
## ---
## Signif. codes:  0 '***' 0.001 '**' 0.01 '*' 0.05 '.' 0.1 ' ' 1
##
```

```
## (Dispersion parameter for binomial family taken to be 1)
##
##     Null deviance: 9307.5  on 407  degrees of freedom
## Residual deviance: 4241.5  on 396  degrees of freedom
## AIC: 5890.6
##
## Number of Fisher Scoring iterations: 5
```

pseudo_r2(glm_6.2)

AIC summary

```
model.sel(list(glm_6.0,glm_6.1,glm_6.2))
```

```
## Model selection table
##      (Int) are    mbl prp_day_trt_cch prp_day_wnt ttl_are men_dpt_vss
## 2  0.04630   + 0.1383          0.1165     -0.1322 -0.4425    -0.05584
## 3 -0.08687   + 0.1243          0.1153     -0.1363 -0.4644
## 1 -0.05553   + 0.1315          0.1153     -0.1312 -0.4599
##        dns df    logLik   AICc delta weight
## 2          12 -2929.846 5884.5  0.00  0.948
## 3 -0.04904 12 -2933.310 5891.4  6.93  0.030
## 1          11 -2934.642 5892.0  7.47  0.023
## Models ranked by AICc(x)
```

Model with the lowest AIC

```
glm_7.0 <- glm(cbind(count,count_total - count)~ area+ total_area+mobility+pr
op_day_trotline_cach+prop_day_winter+mean_depth_vessel, family = binomial(lin
k = logit), data = datsc)
summary(glm_7.0)
```

```
##
## Call:
## glm(formula = cbind(count, count_total - count) ~ area + total_area +
##     mobility + prop_day_trotline_cach + prop_day_winter + mean_depth_vesse
l,
##     family = binomial(link = logit), data = datsc)
##
## Deviance Residuals:
##     Min       1Q   Median       3Q      Max
## -9.0221  -1.9293   0.1905   1.9752  13.0909
##
## Coefficients:
##                         Estimate Std. Error z value Pr(>|z|)
## (Intercept)              0.04630    0.07091   0.653  0.51379
## areaCROZET               0.54710    0.09800   5.583 2.37e-08 ***
## areaFALKLANDS           -0.44909    0.06728  -6.675 2.47e-11 ***
## areaHIMI                -2.76616    0.12198 -22.677  < 2e-16 ***
## areaKERGUELEN            0.67546    0.10326   6.541 6.10e-11 ***
## areaPEMI                -2.64914    0.16504 -16.051  < 2e-16 ***
```

```
## areaSOUTH GEORGIA      -0.66027    0.09015  -7.324 2.40e-13 ***
## total_area             -0.44254    0.03590 -12.328  < 2e-16 ***
## mobility                0.13826    0.02075   6.662 2.70e-11 ***
## prop_day_trotline_cach  0.11654    0.01878   6.207 5.41e-10 ***
## prop_day_winter        -0.13222    0.02530  -5.226 1.73e-07 ***
## mean_depth_vessel      -0.05584    0.01803  -3.097  0.00195 **
## ---
## Signif. codes:  0 '***' 0.001 '**' 0.01 '*' 0.05 '.' 0.1 ' ' 1
##
## (Dispersion parameter for binomial family taken to be 1)
##
##     Null deviance: 9307.5  on 407  degrees of freedom
## Residual deviance: 4234.6  on 396  degrees of freedom
## AIC: 5883.7
##
## Number of Fisher Scoring iterations: 5
```

pseudo_r2(glm_7.0)

Seven terms models

Model with density of vessels

```
glm_7.1 <- glm(cbind(count,count_total - count)~ area+ total_area+mobility+pr
op_day_trotline_cach+prop_day_winter+mean_depth_vessel+density, family = bino
mial(link = logit), data = datsc)
summary(glm_7.1)
```

```
##
## Call:
## glm(formula = cbind(count, count_total - count) ~ area + total_area +
##     mobility + prop_day_trotline_cach + prop_day_winter + mean_depth_vesse
l +
##     density, family = binomial(link = logit), data = datsc)
##
## Deviance Residuals:
##     Min       1Q   Median       3Q      Max
## -9.1496  -1.9158   0.1761   1.9737  13.0314
##
## Coefficients:
##                         Estimate Std. Error z value Pr(>|z|)
## (Intercept)             0.01515    0.07258   0.209 0.834604
## areaCROZET              0.57526    0.09901   5.810 6.24e-09 ***
## areaFALKLANDS          -0.47111    0.06816  -6.912 4.79e-12 ***
## areaHIMI               -2.77844    0.12211 -22.753  < 2e-16 ***
## areaKERGUELEN           0.65903    0.10358   6.362 1.99e-10 ***
## areaPEMI               -2.63429    0.16512 -15.954  < 2e-16 ***
## areaSOUTH GEORGIA      -0.55882    0.10349  -5.400 6.68e-08 ***
## total_area             -0.44677    0.03595 -12.426  < 2e-16 ***
## mobility                0.12983    0.02117   6.133 8.65e-10 ***
## prop_day_trotline_cach  0.11662    0.01876   6.215 5.13e-10 ***
```

```
## prop_day_winter        -0.13853    0.02551  -5.431 5.61e-08 ***
## mean_depth_vessel      -0.05989    0.01816  -3.297 0.000976 ***
## density                -0.06020    0.03029  -1.988 0.046860 *
## ---
## Signif. codes:  0 '***' 0.001 '**' 0.01 '*' 0.05 '.' 0.1 ' ' 1
##
## (Dispersion parameter for binomial family taken to be 1)
##
##     Null deviance: 9307.5  on 407  degrees of freedom
## Residual deviance: 4230.6  on 395  degrees of freedom
## AIC: 5881.7
##
## Number of Fisher Scoring iterations: 5
```

pseudo_r2(glm_7.1)

AIC summary

```
model.sel(list(glm_7.0,glm_7.1))

## Model selection table
##     (Int) are men_dpt_vss    mbl prp_day_trt_cch prp_day_wnt ttl_are
## 2 0.01515   +    -0.05989 0.1298          0.1166     -0.1385 -0.4468
## 1 0.04630   +    -0.05584 0.1383          0.1165     -0.1322 -0.4425
##       dns df    logLik   AICc delta weight
## 2 -0.0602 13 -2927.873 5882.7  0.00  0.712
## 1         12 -2929.846 5884.5  1.81  0.288
## Models ranked by AICc(x)
```

FINAL MODEL without interactions

```
glm_7.0 <- glm(cbind(count,count_total - count)~ area+ total_area+mobility+pr
op_day_trotline_cach+prop_day_winter+mean_depth_vessel+density, family = bino
mial(link = logit), data = datsc)
summary(glm_7.0)

##
## Call:
## glm(formula = cbind(count, count_total - count) ~ area + total_area +
##     mobility + prop_day_trotline_cach + prop_day_winter + mean_depth_vesse
l +
##     density, family = binomial(link = logit), data = datsc)
##
## Deviance Residuals:
##     Min       1Q   Median       3Q      Max
## -9.1496  -1.9158   0.1761   1.9737  13.0314
##
## Coefficients:
##                        Estimate Std. Error z value Pr(>|z|)
## (Intercept)             0.01515    0.07258   0.209 0.834604
## areaCROZET              0.57526    0.09901   5.810 6.24e-09 ***
## areaFALKLANDS          -0.47111    0.06816  -6.912 4.79e-12 ***
```

```
## areaHIMI               -2.77844    0.12211 -22.753  < 2e-16 ***
## areaKERGUELEN           0.65903    0.10358   6.362 1.99e-10 ***
## areaPEMI               -2.63429    0.16512 -15.954  < 2e-16 ***
## areaSOUTH GEORGIA      -0.55882    0.10349  -5.400 6.68e-08 ***
## total_area             -0.44677    0.03595 -12.426  < 2e-16 ***
## mobility                0.12983    0.02117   6.133 8.65e-10 ***
## prop_day_trotline_cach  0.11662    0.01876   6.215 5.13e-10 ***
## prop_day_winter        -0.13853    0.02551  -5.431 5.61e-08 ***
## mean_depth_vessel      -0.05989    0.01816  -3.297 0.000976 ***
## density                -0.06020    0.03029  -1.988 0.046860 *
## ---
## Signif. codes:  0 '***' 0.001 '**' 0.01 '*' 0.05 '.' 0.1 ' ' 1
##
## (Dispersion parameter for binomial family taken to be 1)
##
##     Null deviance: 9307.5  on 407  degrees of freedom
## Residual deviance: 4230.6  on 395  degrees of freedom
## AIC: 5881.7
##
## Number of Fisher Scoring iterations: 5
```

Model validation

plots of residuals versus fitted values for check of linearity and homoscedasticity of variance

```
plot(fitted(glm_7.0), resid(glm_7.0), main="Residual Plot for linearity and h
omoscedasticity of variance",xlab="Fitted value",ylab="Residuals")
```

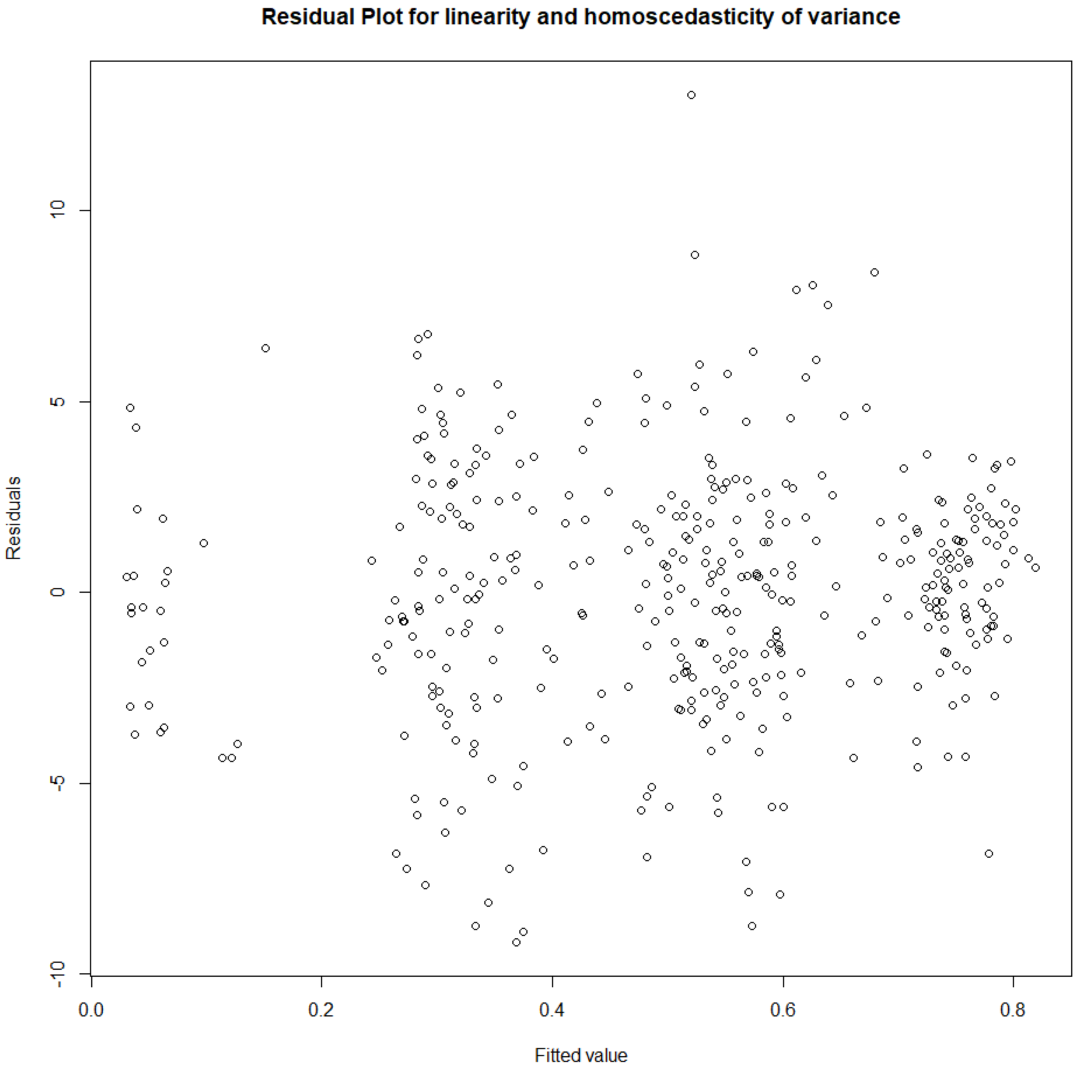

test for normality of the residuals

```
hist(resid(glm_7.0),xlab="Residuals",main="Normality of the Residuals")
```

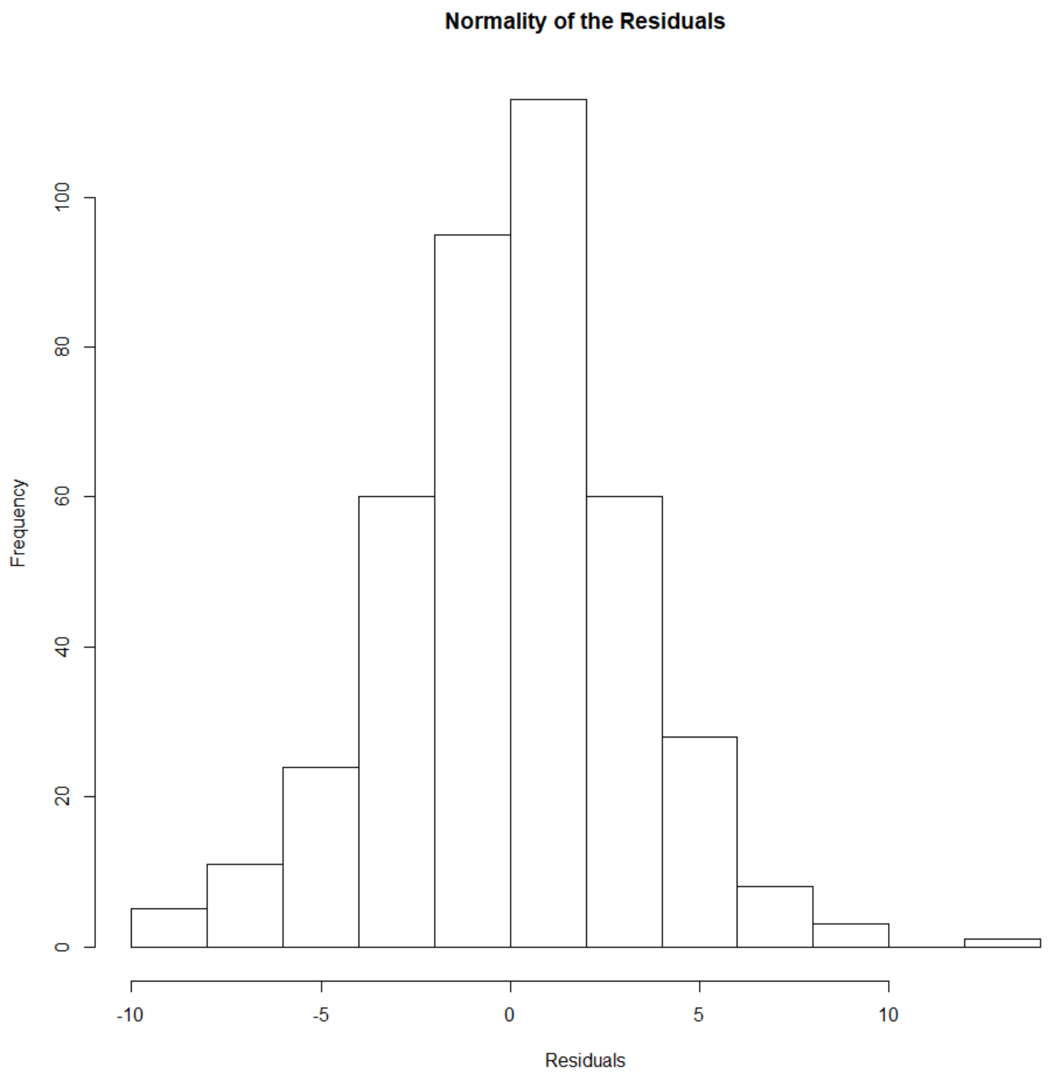

Interaction between Area and mobility

A reviewer requested we investigate including an interaction between fishery and mobility

```
glm_8.0 <- glm(cbind(count,count_total - count)~ area+ total_area+mobility+
                 prop_day_trotline_cach+prop_day_winter+mean_depth_vessel+den
sity+
                 area:mobility, family = binomial(link = logit), data = datsc
)
summary(glm_8.0)
```

```
##
## Call:
## glm(formula = cbind(count, count_total - count) ~ area + total_area +
##     mobility + prop_day_trotline_cach + prop_day_winter + mean_depth_vesse
l +
##     density + area:mobility, family = binomial(link = logit),
```

```
##     data = datsc)
##
## Deviance Residuals:
##     Min       1Q   Median       3Q      Max
## -9.0597  -1.9177   0.1581   2.0102  13.5065
##
## Coefficients:
##                            Estimate Std. Error z value Pr(>|z|)
## (Intercept)                 0.36637    0.09173   3.994 6.49e-05 ***
## areaCROZET                  0.29162    0.11384   2.562  0.01042 *
## areaFALKLANDS              -0.82595    0.08835  -9.349  < 2e-16 ***
## areaHIMI                   -3.05541    0.15455 -19.769  < 2e-16 ***
## areaKERGUELEN               0.32107    0.11670   2.751  0.00594 **
## areaPEMI                   -3.47488    0.26016 -13.357  < 2e-16 ***
## areaSOUTH GEORGIA          -0.93973    0.12060  -7.792 6.58e-15 ***
## total_area                 -0.43440    0.03776 -11.503  < 2e-16 ***
## mobility                    0.47760    0.05831   8.190 2.61e-16 ***
## prop_day_trotline_cach      0.11154    0.02019   5.524 3.32e-08 ***
## prop_day_winter            -0.14220    0.02663  -5.339 9.34e-08 ***
## mean_depth_vessel          -0.03965    0.01862  -2.130  0.03320 *
## density                    -0.06882    0.03298  -2.087  0.03690 *
## areaCROZET:mobility        -0.41612    0.08187  -5.083 3.72e-07 ***
## areaFALKLANDS:mobility     -0.34864    0.08541  -4.082 4.47e-05 ***
## areaHIMI:mobility          -0.10264    0.21409  -0.479  0.63162
## areaKERGUELEN:mobility     -0.40951    0.06710  -6.103 1.04e-09 ***
## areaPEMI:mobility          -1.21644    0.27923  -4.356 1.32e-05 ***
## areaSOUTH GEORGIA:mobility -0.40204    0.07798  -5.156 2.52e-07 ***
## ---
## Signif. codes:  0 '***' 0.001 '**' 0.01 '*' 0.05 '.' 0.1 ' ' 1
##
## (Dispersion parameter for binomial family taken to be 1)
##
##     Null deviance: 9307.5  on 407  degrees of freedom
## Residual deviance: 4175.3  on 389  degrees of freedom
## AIC: 5838.5
##
## Number of Fisher Scoring iterations: 6
```

Calculate the pseudo r squared

```
pseudo_r2(glm_8.0)

## [1] 0.551402
```

Check the plots of residuals versus fitted values for check of linearity and homoscedasticity of variance

```
plot(fitted(glm_8.0), resid(glm_8.0), main="Residual Plot for linearity and h
omoscedasticity of variance",xlab="Fitted value",ylab="Residuals")
```

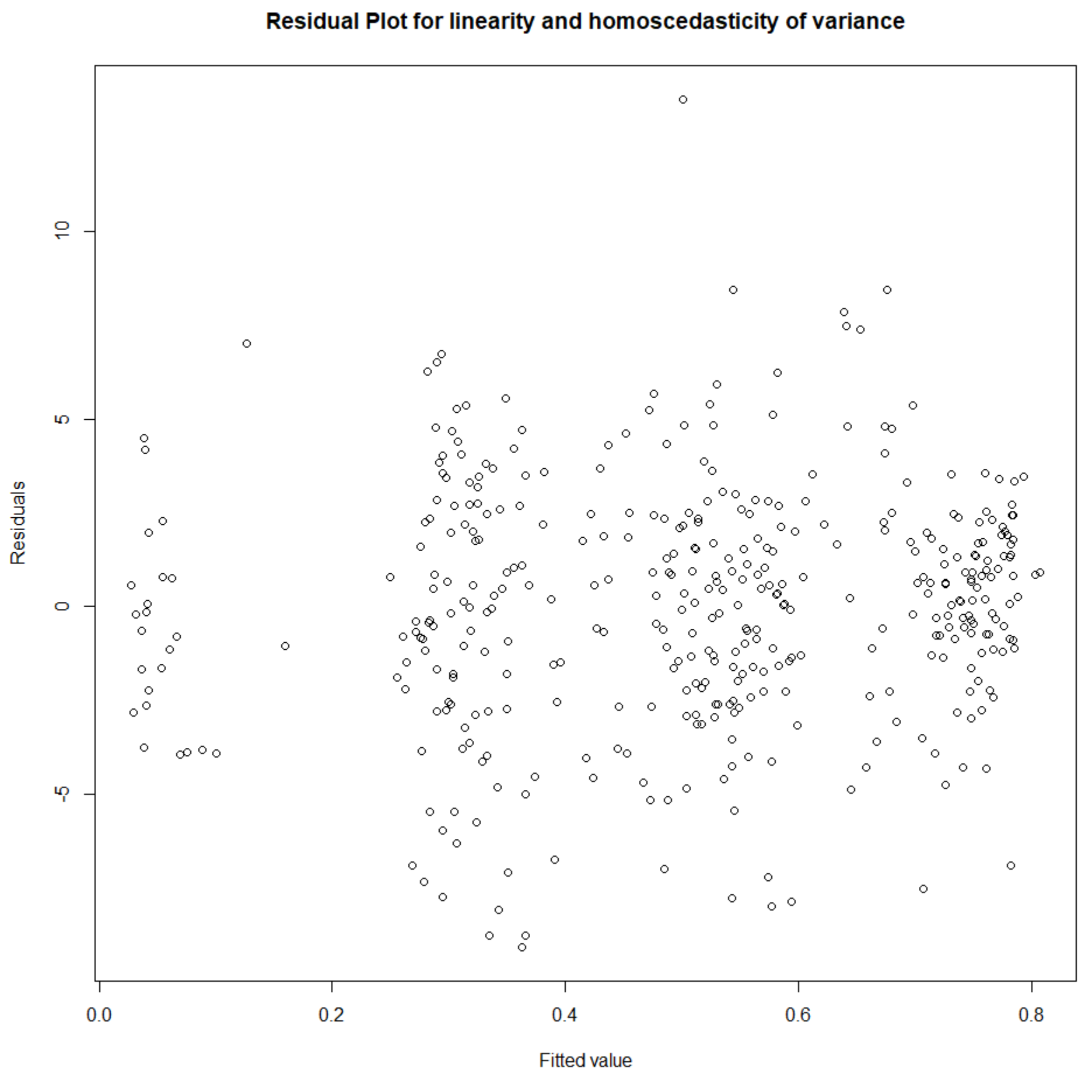

test for normality of the residuals

```
hist(resid(glm_8.0),xlab="Residuals",main="Normality of the Residuals")
```

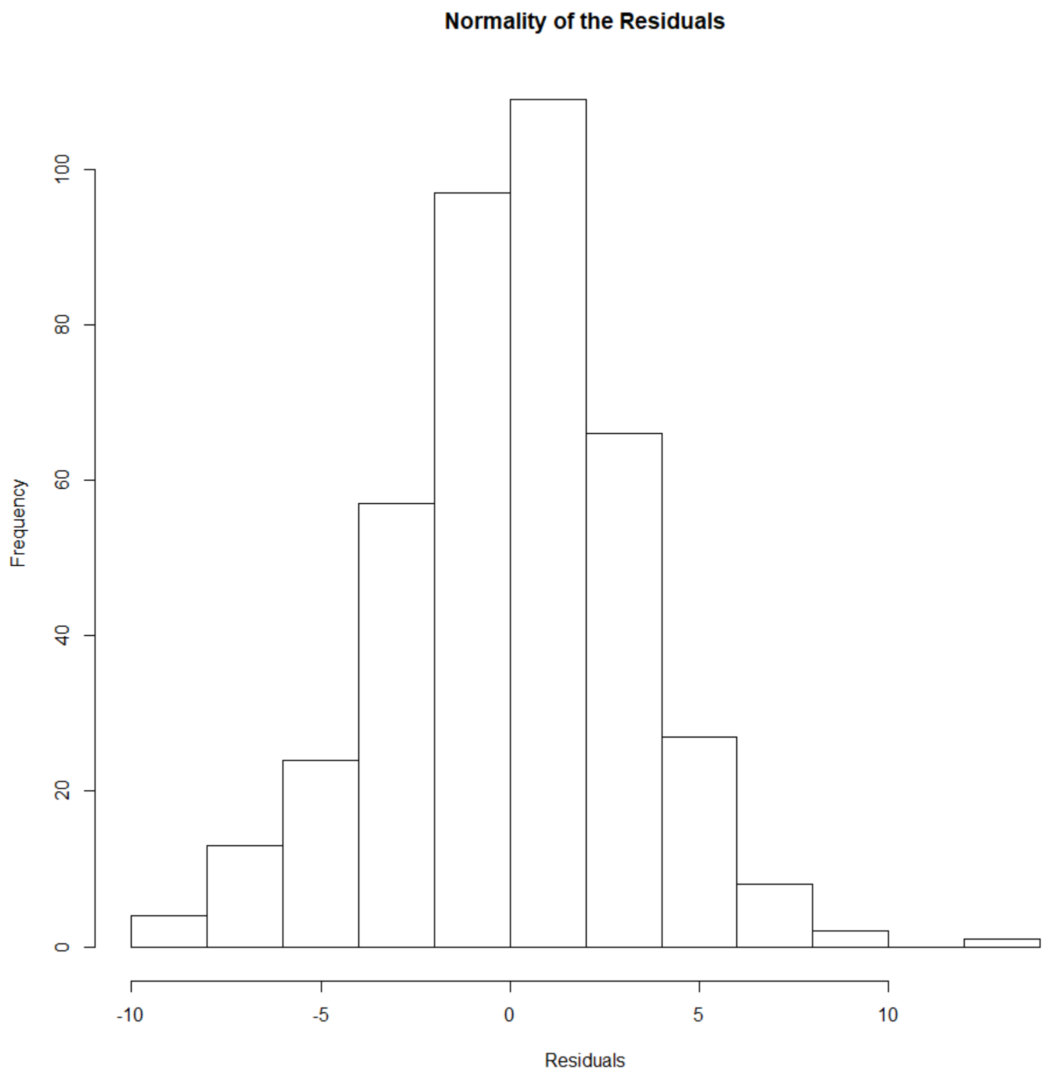

Create the AIC selection table including `glm_8.0`, we also calculate all of the pseudo r squared statistics using the `extra` argument of `model.sel`.

```
(vessel_summary <- model.sel(list(glm_null, glm_1.1,glm_1.2,glm_1.3,glm_1.4,g
lm_1.5,glm_1.6,glm_1.7,
               glm_2.0,glm_2.1,glm_2.2,glm_2.3,glm_2.4,glm_2.5,glm_2.6,
               glm_3.0,glm_3.1,glm_3.2,glm_3.3,glm_3.4,glm_3.5,
               glm_4.0,glm_4.1,glm_4.2,glm_4.3,glm_4.4,
               glm_5.0,glm_5.1,glm_5.2,glm_5.3,
               glm_6.0,glm_6.1,glm_6.2,
               glm_7.0,glm_7.1,
               glm_8.0), rank=AIC, extra=pseudo_r2))

## Model selection table
##       (Int) men_dpt_vss prp_day_trt_cch prp_day_wnt  ttl_are      dns are
## 36  0.36640    -0.03965          0.1115     -0.1422 -0.43440 -0.06882   +
## 34  0.01515    -0.05989          0.1166     -0.1385 -0.44680 -0.06020   +
```

```
## 35  0.01515    -0.05989          0.1166     -0.1385 -0.44680 -0.06020   +
## 32  0.04630    -0.05584          0.1165     -0.1322 -0.44250            +
## 33 -0.08687                      0.1153     -0.1363 -0.46440 -0.04904   +
## 30 -0.05553                      0.1153     -0.1312 -0.45990            +
## 31 -0.05553                      0.1153     -0.1312 -0.45990            +
## 28  0.18430    -0.05438          0.1033             -0.44650            +
## 24  0.08397                      0.1022             -0.46330            +
## 27  0.08397                      0.1022             -0.46330            +
## 29  0.06825                      0.1019             -0.46610 -0.02977   +
## 26  0.23420                                 -0.1098 -0.46800            +
## 23  0.42420    -0.05248                             -0.45390            +
## 19  0.32490                                         -0.47010            +
## 22  0.32490                                         -0.47010            +
## 25  0.30700                                         -0.47300 -0.03231   +
## 18 -0.10230                      0.1229             -0.40950            +
## 21  0.07006                                 -0.1228 -0.40870            +
## 20  0.13260                                         -0.41990 -0.07793   +
## 17  0.22800    -0.03590                             -0.39550            +
## 13  0.16470                                         -0.40830            +
## 16  0.16470                                         -0.40830            +
## 11 -0.01548                      0.1213                                 +
## 15  0.35590                                                             +
## 12  0.15370                                 -0.1216                     +
## 10  0.37090    -0.07287                                                 +
## 7   0.24760                                                             +
## 9   0.24760                                                             +
## 14  0.23480                                                  -0.03363   +
## 4  -0.16090                                 -0.5243
## 8  -0.11250
## 6  -0.20010                                                  -0.23130
## 3  -0.16940                      0.1365
## 2  -0.13770    -0.08690
## 5  -0.16510                                           0.04943
## 1  -0.15050
##       mbl are:mbl  pseudo_r2          weights df    logLik     AIC
## 36 0.4776       +  5.514e-01                  19 -2900.228  5838.5
## 34 0.1298          5.455e-01                  13 -2927.873  5881.7
## 35 0.1298          5.455e-01                  13 -2927.873  5881.7
## 32 0.1383          5.450e-01                  12 -2929.846  5883.7
## 33 0.1243          5.443e-01                  12 -2933.310  5890.6
## 30 0.1315          5.440e-01                  11 -2934.642  5891.3
## 31 0.1315          5.440e-01                  11 -2934.642  5891.3
## 28 0.1482          5.421e-01                  11 -2943.428  5908.9
## 24 0.1414          5.411e-01                  10 -2947.994  5916.0
## 27 0.1414          5.411e-01                  10 -2947.994  5916.0
## 29 0.1373          5.412e-01                  11 -2947.496  5917.0
## 26 0.1540          5.399e-01                  10 -2953.598  5927.2
## 23 0.1668          5.388e-01                  10 -2958.893  5937.8
## 19 0.1601          5.379e-01                   9 -2963.145  5944.3
## 22 0.1601          5.379e-01                   9 -2963.145  5944.3
```

```
## 25 0.1556          5.380e-01                     10 -2962.560  5945.1
## 18                 5.360e-01                      9 -2971.781  5961.6
## 21                 5.337e-01                      9 -2982.440  5982.9
## 20                 5.319e-01                      9 -2990.923  5999.8
## 17                 5.316e-01                      9 -2992.475  6003.0
## 13                 5.311e-01                      8 -2994.500  6005.0
## 16                 5.311e-01                      8 -2994.500  6005.0
## 11                 5.207e-01                      8 -3043.079  6102.2
## 15 0.1009          5.187e-01                      8 -3052.214  6120.4
## 12                 5.185e-01                      8 -3053.545  6123.1
## 10                 5.178e-01                      8 -3056.701  6129.4
## 7                  5.159e-01 na.action(na.fail)  7 -3065.383  6144.8
## 9                  5.159e-01                      7 -3065.383  6144.8
## 14                 5.161e-01                      8 -3064.699  6145.4
## 4                  2.011e-01 na.action(na.fail)  2 -4530.494  9065.0
## 8  0.3477          9.476e-02 na.action(na.fail)  2 -5025.349 10054.7
## 6                  4.156e-02 na.action(na.fail)  2 -5272.895 10549.8
## 3                  2.234e-02 na.action(na.fail)  2 -5362.380 10728.8
## 2                  6.630e-03 na.action(na.fail)  2 -5435.472 10874.9
## 5                  2.349e-03 na.action(na.fail)  2 -5455.399 10914.8
## 1                 -4.441e-16                      1 -5466.329 10934.7
##      delta weight
## 36    0.00      1
## 34   43.29      0
## 35   43.29      0
## 32   45.24      0
## 33   52.16      0
## 30   52.83      0
## 31   52.83      0
## 28   70.40      0
## 24   77.53      0
## 27   77.53      0
## 29   78.54      0
## 26   88.74      0
## 23   99.33      0
## 19  105.83      0
## 22  105.83      0
## 25  106.67      0
## 18  123.11      0
## 21  144.43      0
## 20  161.39      0
## 17  164.49      0
## 13  166.54      0
## 16  166.54      0
## 11  263.70      0
## 15  281.97      0
## 12  284.63      0
## 10  290.95      0
## 7   306.31      0
## 9   306.31      0
```

```
## 14  306.94      0
## 4  3226.53      0
## 8  4216.24      0
## 6  4711.34      0
## 3  4890.30      0
## 2  5036.49      0
## 5  5076.34      0
## 1  5096.20      0
## Models ranked by AIC(x)
```

Save a copy to file

```
write.csv(vessel_summary, "Sperm_whale_vessel_data_AIC_summary.csv")
```

Coefficients of final model

Extract the coefficients of the final model and convert from logit space to natural numbers (i.e. probabilities) and the upper and lower confidence intervals

```
vsl_est <- plogis(summary(glm_8.0)$coefficients[,1]+summary(glm_8.0)$coeffici
ents[1,1])
vsl_lwr <- plogis((summary(glm_8.0)$coefficients[,1]+summary(glm_8.0)$coeffic
ients[1,1]-1.96*summary(glm_8.0)$coefficients[,2]))
vsl_upr <- plogis((summary(glm_8.0)$coefficients[,1]+summary(glm_8.0)$coeffic
ients[1,1]+1.96*summary(glm_8.0)$coefficients[,2]))
vsl_z_stat <- summary(glm_8.0)$coefficients[,3]
vsl_p_value <- summary(glm_8.0)$coefficients[,4]
```

Bind them together

```
(res_vessel <- cbind(vsl_est, vsl_lwr, vsl_upr, vsl_z_stat, vsl_p_value))

##                                vsl_est    vsl_lwr    vsl_upr  vsl_z_stat
## (Intercept)                 0.67540750 0.63482138 0.71351847   3.9940843
## areaCROZET                  0.65881034 0.60703409 0.70705681   2.5616038
## areaFALKLANDS               0.38708728 0.34689037 0.42888335  -9.3486757
## areaHIMI                    0.06362329 0.04779017 0.08423795 -19.7691201
## areaKERGUELEN               0.66539736 0.61270995 0.71425978   2.7513333
## areaPEMI                    0.04275772 0.02612443 0.06922851 -13.3569075
## areaSOUTH GEORGIA           0.36046342 0.30794974 0.41654211  -7.7923341
## total_area                  0.48300077 0.46454923 0.50149878 -11.5027835
## mobility                    0.69930085 0.67473391 0.72277464   8.1900717
## prop_day_trotline_cach      0.61725543 0.60786243 0.62656166   5.5236764
## prop_day_winter             0.55581063 0.54288837 0.56865783  -5.3391073
## mean_depth_vessel           0.58096081 0.57205118 0.58981796  -2.1297117
## density                     0.57384422 0.55796705 0.58957061  -2.0868184
## areaCROZET:mobility         0.48756631 0.44763995 0.52765198  -5.0826441
## areaFALKLANDS:mobility      0.50443289 0.46265096 0.54615299  -4.0818847
## areaHIMI:mobility           0.56555254 0.46110625 0.66448515  -0.4794443
## areaKERGUELEN:mobility      0.48921625 0.45644779 0.52207765  -6.1033384
## areaPEMI:mobility           0.29941825 0.19823616 0.42487700  -4.3564604
```

```
## areaSOUTH GEORGIA:mobility 0.49108330 0.45301214 0.52925816  -5.1558501
##                              vsl_p_value
## (Intercept)                 6.494474e-05
## areaCROZET                  1.041901e-02
## areaFALKLANDS               8.875200e-21
## areaHIMI                    5.492554e-87
## areaKERGUELEN               5.935321e-03
## areaPEMI                    1.079568e-40
## areaSOUTH GEORGIA           6.578251e-15
## total_area                  1.277286e-30
## mobility                    2.610706e-16
## prop_day_trotline_cach      3.319787e-08
## prop_day_winter             9.340534e-08
## mean_depth_vessel           3.319542e-02
## density                     3.690456e-02
## areaCROZET:mobility         3.722167e-07
## areaFALKLANDS:mobility      4.467195e-05
## areaHIMI:mobility           6.316226e-01
## areaKERGUELEN:mobility      1.038756e-09
## areaPEMI:mobility           1.321826e-05
## areaSOUTH GEORGIA:mobility 2.524828e-07
```

Create the estimates for Chile (which is zero in binomial regression model)

```
(areaChile <- c(plogis(0+summary(glm_8.0)$coefficients[1,1]),
               plogis(0+summary(glm_8.0)$coefficients[1,1]-1.96*summary(glm_8
.0)$coefficients[1,2]),
               plogis(0+summary(glm_8.0)$coefficients[1,1]+1.96*summary(glm_8
.0)$coefficients[1,2]),
               summary(glm_8.0)$coefficients[1,3], summary(glm_8.0)$coefficie
nts[1,4]))

## [1] 5.905822e-01 5.465112e-01 6.332446e-01 3.994084e+00 6.494474e-05
```

Replace the intercept with Chilean values

```
res_vessel[1,] <- areaChile
```

Save as a csv

```
write.csv(res_vessel, "Sperm_whale_final_model_vessel_est.csv")
```

B Models on *Pr(days)* at the fleet level

B.1. Data preparation

Import the data

```
setwd("C:/Users/LES_research211/Documents/003_ANALYSES/009_SOUTHERN_OCEAN_PAP
ER")
```

```
data=read.csv("summary_proportions_predictors_fleet_pemi_a.csv",h=T,sep=",")
str(data)
```

```
## 'data.frame':    912 obs. of  22 variables:
##  $ area                 : Factor w/ 7 levels "CHILE","CROZET",..: 6 6 6 5
5 5 5 5 5 4 ...
##  $ year                 : int  2017 2017 2017 2013 2013 2013 2016 2016 20
16 2011 ...
##  $ depredation          : int  0 0 0 0 0 0 0 0 0 0 ...
##  $ count                : int  366 130 133 2557 253 875 2532 248 838 493
...
##  $ count_total          : int  130 130 130 253 253 253 248 248 248 191 ..
.
##  $ Pr_inter             : num  1 1 1 1 1 1 1 1 1 1 ...
##  $ species              : Factor w/ 2 levels "killer whales",..: 2 2 2 1
1 1 1 1 1 1 ...
##  $ response             : Factor w/ 4 levels "Pr(area)","Pr(days)",..: 3
2 1 3 2 1 3 2 1 3 ...
##  $ area_yr              : Factor w/ 76 levels "CHILE 2006","CHILE 2007",.
.: 62 62 62 56 56 56 59 59 59 40 ...
##  $ total_area           : int  133 133 133 875 875 875 838 838 838 223 ..
.
##  $ length_season        : int  130 130 130 253 253 253 248 248 248 191 ..
.
##  $ mean_nb_vessels      : num  1 1 1 4.38 4.38 ...
##  $ mean_lat             : num  -45.3 -45.3 -45.3 -48.9 -48.9 ...
##  $ mean_depth           : num  1352 1352 1352 1199 1199 ...
##  $ prop_day_winter      : num  0.462 0.462 0.462 0.158 0.158 ...
##  $ prop_day_spring      : num  0 0 0 0.316 0.316 ...
##  $ prop_day_summer      : num  0.0692 0.0692 0.0692 0.2451 0.2451 ...
##  $ prop_day_autumn      : num  0.469 0.469 0.469 0.281 0.281 ...
##  $ prop_day_spanish     : num  0 0 0 0 0 0 0 0 0 0 ...
##  $ prop_day_autoline    : num  0 0 0 1 1 1 1 1 1 1 ...
##  $ prop_day_trotline_cach: num  0.485 0.485 0.485 0 0 ...
##  $ prop_day_trotline    : num  0.515 0.515 0.515 0 0 ...
```

Prepare the data for modelling

```
data$length_season = as.numeric(as.character(data$length_season))
data$total_area=as.numeric(as.character(data$total_area))
data$mobility=data$total_area/data$length_season
data$prop_day_trotline_cach=as.numeric(as.character(data$prop_day_trotline_ca
ch))
data <-subset(data,!is.na(mean_lat))
data <-subset(data,!is.na(mean_depth))
data$mean_lat=-data$mean_lat
data$density = data$mean_nb_vessels/data$total_area
```

Data selection

```
d1 <- droplevels(data[data$depredation==1 & data$species %in% "sperm whales"
& data$response %in% "Pr(days)",
        c("area","year","count","count_total","Pr_inter", "total_area","mean_
nb_vessels", "mean_lat", "mean_depth","mobility","prop_day_trotline_cach", "p
rop_day_winter","density" )])
str(d1)
```

```
## 'data.frame':    74 obs. of  13 variables:
##  $ area                  : Factor w/ 7 levels "CHILE","CROZET",..: 1 7 2 2
7 1 2 7 7 1 ...
##  $ year                  : int  2006 2009 2011 2006 2010 2007 2012 2006 20
04 2011 ...
##  $ count                 : int  79 117 80 145 115 150 122 109 99 250 ...
##  $ count_total           : int  79 123 86 158 128 167 136 123 112 283 ...
##  $ Pr_inter              : num  1 0.951 0.93 0.918 0.898 ...
##  $ total_area            : num  141 304 166 204 325 173 192 327 356 405 ..
.
##  $ mean_nb_vessels       : num  2.45 6.69 3.49 2.38 6.44 ...
##  $ mean_lat              : num  55.7 53.9 46.3 46.2 54 ...
##  $ mean_depth            : num  1526 1266 986 949 1238 ...
##  $ mobility              : num  1.78 2.47 1.93 1.29 2.54 ...
##  $ prop_day_trotline_cach: num  0.848 0 0 0 0 ...
##  $ prop_day_winter       : num  0 0.7562 0.0465 0.1772 0.6745 ...
##  $ density               : num  0.0173 0.022 0.021 0.0117 0.0198 ...
```

Data exploration

```
hist(d1$mean_depth)
```

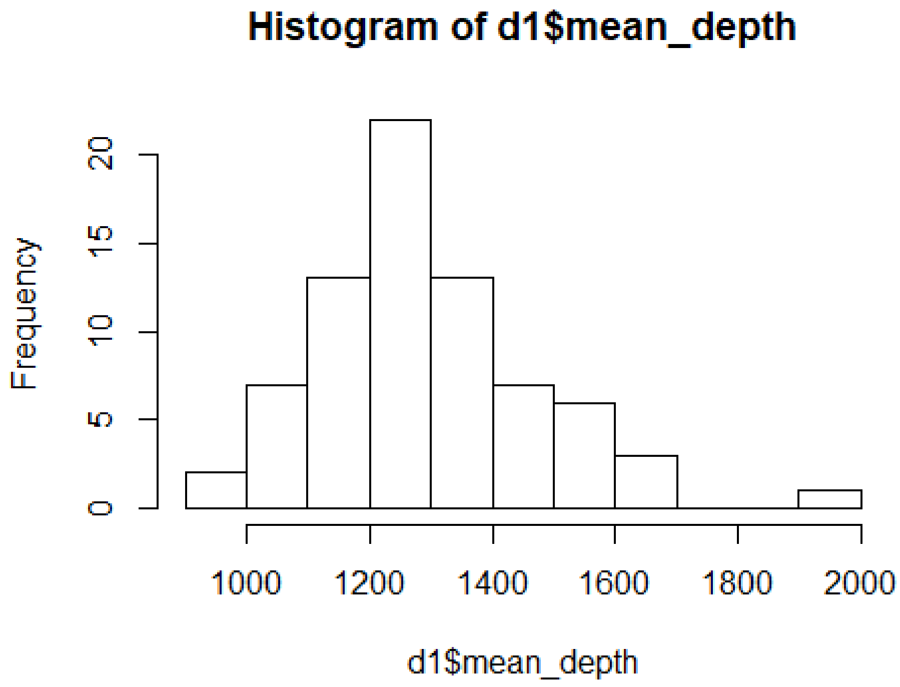


```
hist(d1$total_area)
```

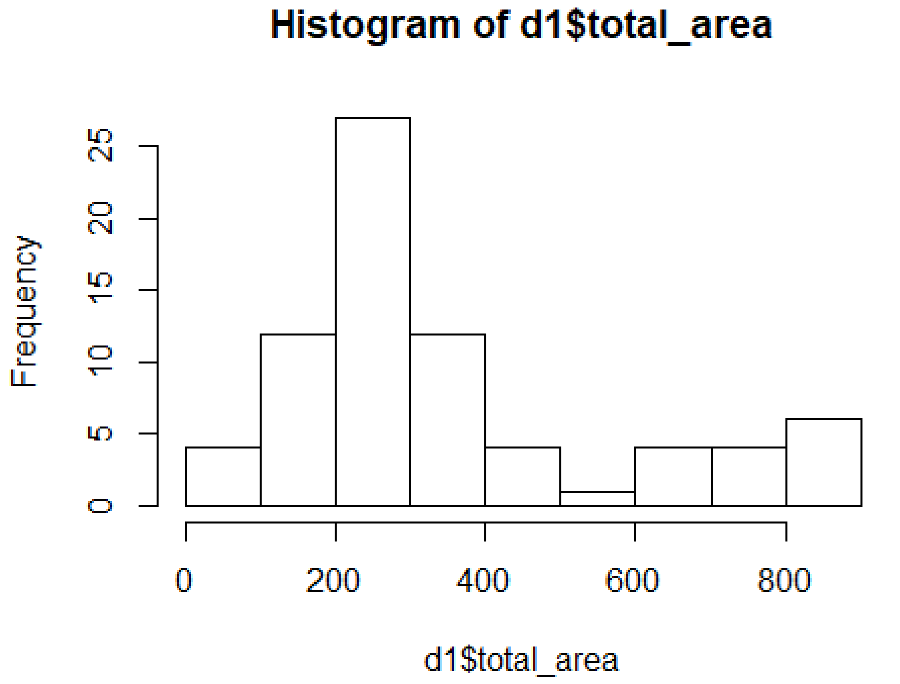


```
hist(d1$mobility)
```

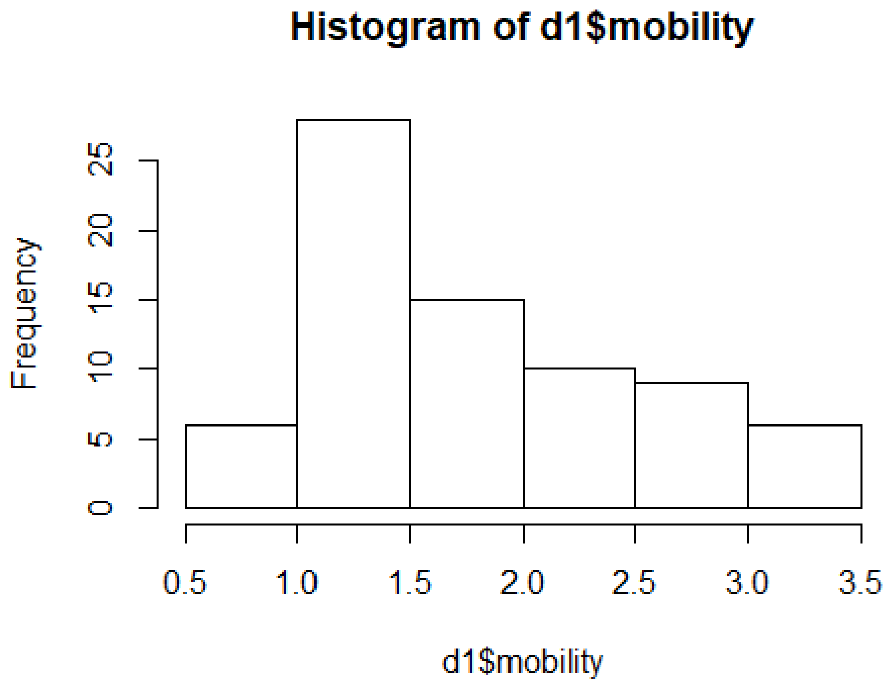


```
# exclude extreme high value
#d1 = subset(d1, d1$density<=0.035)

hist(d1$density)
```

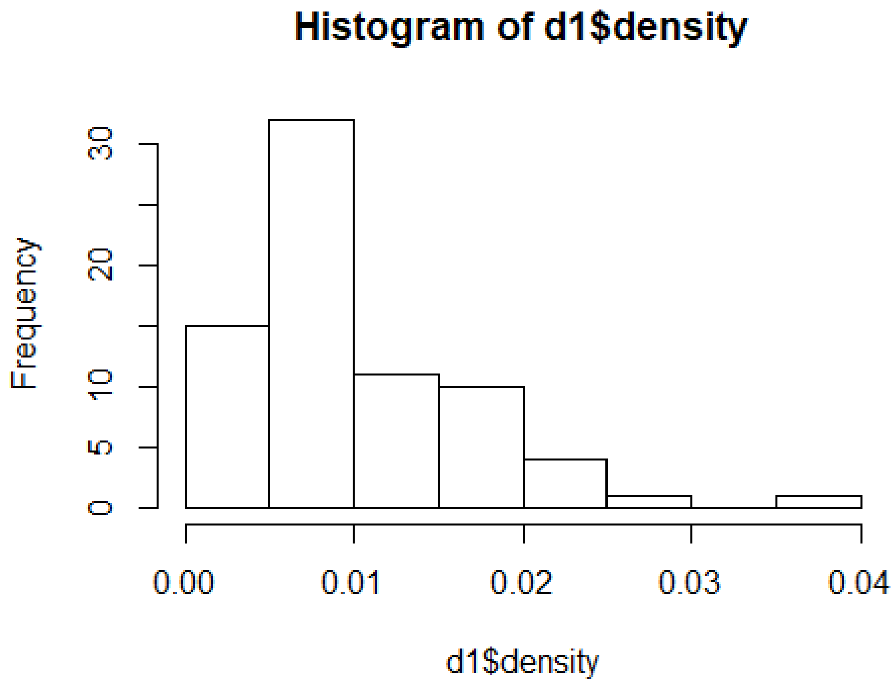


```
# exclude extreme high value
d1 = subset(d1, d1$density<=0.03)
```

Exploratory plots

```
p1 <- ggplot(d1,aes(total_area,Pr_inter,colour=area))+geom_point()+ggtitle("s
ize fishing area")
p2 <- ggplot(d1,aes(mean_depth,Pr_inter,colour=area))+geom_point()+ggtitle("d
epth")
p3 <- ggplot(d1,aes(density,Pr_inter,colour=area))+geom_point()+ggtitle("dens
ity")
p4 <- ggplot(d1,aes(prop_day_winter,Pr_inter,colour=area))+geom_point()+ggtit
le("effort in winter")
p5 <- ggplot(d1,aes(mobility,Pr_inter,colour=area))+geom_point()+ggtitle("mob
ility")
p6 <- ggplot(d1,aes(count_total,Pr_inter,colour=area))+geom_point()+ggtitle("
effort")


ggarrange(p1, p2,p3,p4,p5, common.legend=TRUE, legend = "right")
```

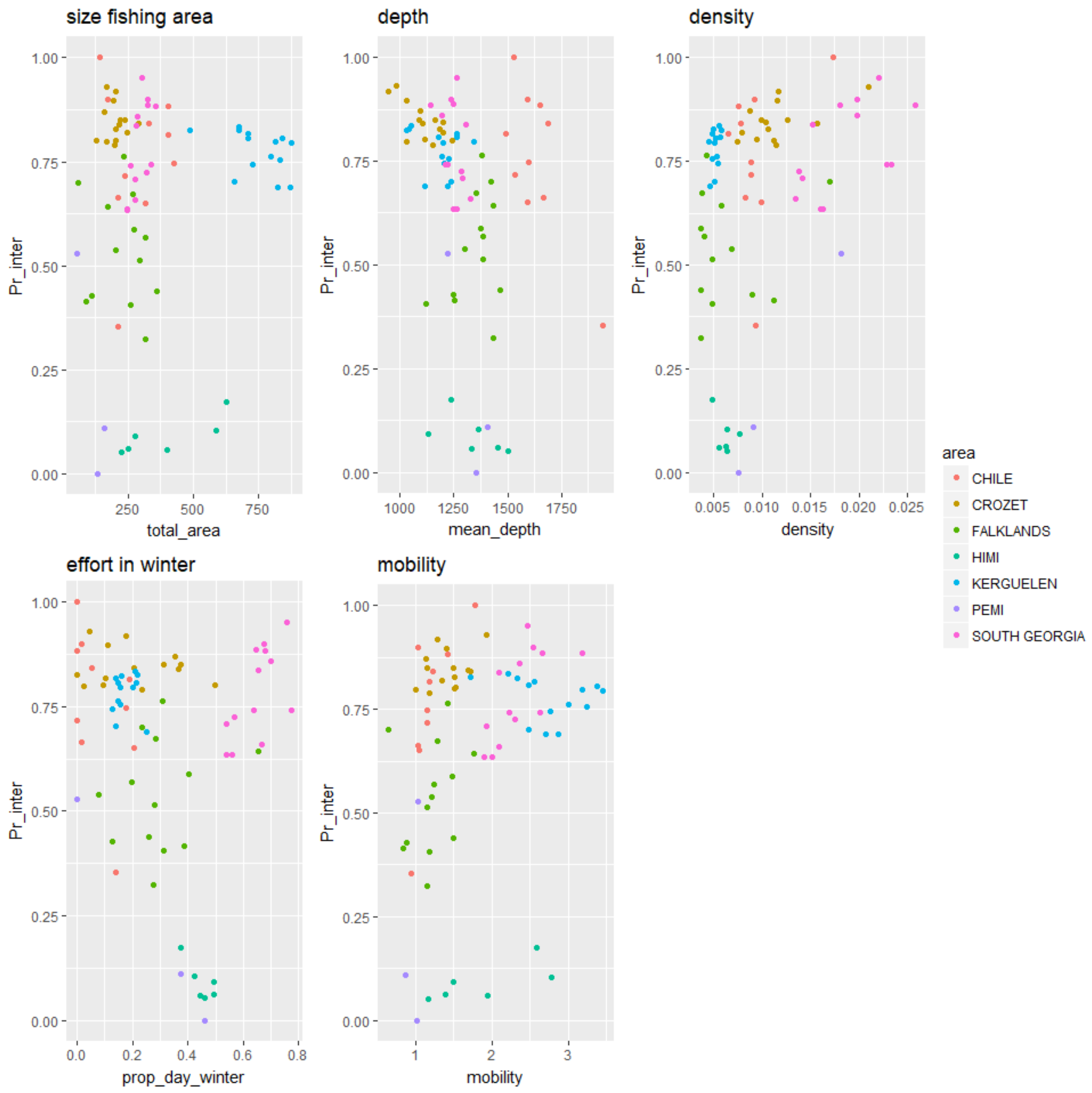

Pairwise plots

```
d1_explo <-droplevels(d1[,
        c(  "mean_depth","total_area","density", "count_total","mobility","pr
op_day_winter","prop_day_trotline_cach")])
pairs(d1_explo)
```

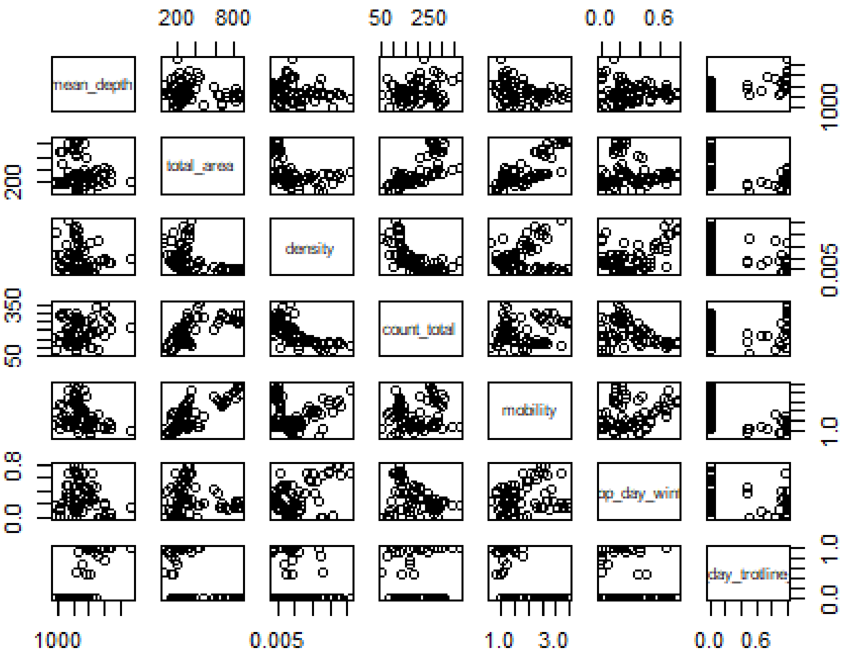

Pearson correlation

Calculate Pearson correlation coefficients for the above data

```
print(cor(d1_explo, method=c("pearson")), digits=4)
```

```
##                        mean_depth total_area  density count_total mobility
## mean_depth                 1.0000    -0.1568 -0.16876     0.20580 -0.32776
## total_area                -0.1568     1.0000 -0.43097     0.70497  0.78957
## density                   -0.1688    -0.4310  1.00000    -0.69281  0.09026
## count_total                0.2058     0.7050 -0.69281     1.00000  0.18197
## mobility                  -0.3278     0.7896  0.09026     0.18197  1.00000
## prop_day_winter           -0.1407    -0.1302  0.46705    -0.37471  0.27328
## prop_day_trotline_cach     0.7037    -0.3335 -0.14706     0.05914 -0.53103
##                        prop_day_winter prop_day_trotline_cach
## mean_depth                     -0.1407                0.70365
## total_area                     -0.1302               -0.33346
## density                         0.4671               -0.14706
## count_total                    -0.3747                0.05914
## mobility                        0.2733               -0.53103
## prop_day_winter                 1.0000               -0.31019
## prop_day_trotline_cach         -0.3102                1.00000
```

Re-scale continuous predictors

```
pvars <- c("total_area", "mean_depth","mobility","prop_day_trotline_cach", "p
rop_day_winter","density" )
```

```
datsc <- d1
datsc[pvars] <- lapply(datsc[pvars],scale)
```

We fit a glm to the proportion of days with sperm whale interaction using a binomial family and a logit link function. The most parcimonious model is selected using a stepwise forward AIC selection

Null model

```
glm_null <- glm(cbind(count,count_total - count)~ 1, family = binomial(link =
logit), data = datsc)
summary(glm_null)

##
## Call:
## glm(formula = cbind(count, count_total - count) ~ 1, family = binomial(lin
k = logit),
##     data = datsc)
##
## Deviance Residuals:
##     Min       1Q   Median       3Q      Max
## -18.319   -1.985    2.883    5.457    8.650
##
## Coefficients:
##             Estimate Std. Error z value Pr(>|z|)
## (Intercept)  0.66572    0.01789   37.22   <2e-16 ***
## ---
## Signif. codes:  0 '***' 0.001 '**' 0.01 '*' 0.05 '.' 0.1 ' ' 1
##
## (Dispersion parameter for binomial family taken to be 1)
##
##     Null deviance: 3878.9  on 72  degrees of freedom
## Residual deviance: 3878.9  on 72  degrees of freedom
## AIC: 4246.8
##
## Number of Fisher Scoring iterations: 4
```

Calculate pseudo r squared for the Null model as a check

```
pseudo_r2(glm_null)

## [1] 0
```

Single term models

Model with total area size

```
glm_1.1 <- glm(cbind(count,count_total - count)~ total_area, family = binomia
l(link = logit), data = datsc)
summary(glm_1.1)
```

```
##
## Call:
## glm(formula = cbind(count, count_total - count) ~ total_area,
##     family = binomial(link = logit), data = datsc)
##
## Deviance Residuals:
##     Min       1Q   Median       3Q      Max
## -18.302   -1.829    2.285    5.455    8.846
##
## Coefficients:
##             Estimate Std. Error z value Pr(>|z|)
## (Intercept)  0.62310    0.01827    34.1   <2e-16 ***
## total_area   0.18756    0.01752    10.7   <2e-16 ***
## ---
## Signif. codes:  0 '***' 0.001 '**' 0.01 '*' 0.05 '.' 0.1 ' ' 1
##
## (Dispersion parameter for binomial family taken to be 1)
##
##     Null deviance: 3878.9  on 72  degrees of freedom
## Residual deviance: 3761.3  on 71  degrees of freedom
## AIC: 4131.2
##
## Number of Fisher Scoring iterations: 4
```

Calculate pseudo r squared

```
pseudo_r2(glm_1.1)

## [1] 0.0303264
```

Model with depth

```
glm_1.2 <- glm(cbind(count,count_total - count)~ mean_depth, family = binomia
l(link = logit), data = datsc)
summary(glm_1.2)

##
## Call:
## glm(formula = cbind(count, count_total - count) ~ mean_depth,
##     family = binomial(link = logit), data = datsc)
##
## Deviance Residuals:
##     Min       1Q   Median       3Q      Max
## -18.114   -1.464    2.429    4.248   12.122
##
## Coefficients:
##             Estimate Std. Error z value Pr(>|z|)
## (Intercept)  0.69542    0.01822   38.16   <2e-16 ***
## mean_depth  -0.25299    0.01697  -14.91   <2e-16 ***
## ---
## Signif. codes:  0 '***' 0.001 '**' 0.01 '*' 0.05 '.' 0.1 ' ' 1
```

```
##
## (Dispersion parameter for binomial family taken to be 1)
##
##     Null deviance: 3878.9  on 72  degrees of freedom
## Residual deviance: 3654.8  on 71  degrees of freedom
## AIC: 4024.8
##
## Number of Fisher Scoring iterations: 4
```

Calculate pseudo r squared

```
pseudo_r2(glm_1.2)

## [1] 0.05777331
```

Model with mobility

```
glm_1.3 <- glm(cbind(count,count_total - count)~ mobility, family = binomial(
link = logit), data = datsc)
summary(glm_1.3)

##
## Call:
## glm(formula = cbind(count, count_total - count) ~ mobility, family = binom
ial(link = logit),
##     data = datsc)
##
## Deviance Residuals:
##     Min       1Q   Median       3Q      Max
## -19.204   -1.424    1.621    5.089    9.605
##
## Coefficients:
##             Estimate Std. Error z value Pr(>|z|)
## (Intercept)  0.66003    0.01802   36.63   <2e-16 ***
## mobility     0.24774    0.01792   13.82   <2e-16 ***
## ---
## Signif. codes:  0 '***' 0.001 '**' 0.01 '*' 0.05 '.' 0.1 ' ' 1
##
## (Dispersion parameter for binomial family taken to be 1)
##
##     Null deviance: 3878.9  on 72  degrees of freedom
## Residual deviance: 3681.6  on 71  degrees of freedom
## AIC: 4051.6
##
## Number of Fisher Scoring iterations: 4
```

Calculate pseudo r squared

```
pseudo_r2(glm_1.3)

## [1] 0.05086386
```

Model with proportion of sets using trotlines-cachalotera

```
glm_1.4 <- glm(cbind(count,count_total - count)~ prop_day_trotline_cach, fami
ly = binomial(link = logit), data = datsc)
summary(glm_1.4)

##
## Call:
## glm(formula = cbind(count, count_total - count) ~ prop_day_trotline_cach,
##     family = binomial(link = logit), data = datsc)
##
## Deviance Residuals:
##     Min       1Q   Median       3Q      Max
## -18.349   -1.964    2.898    5.437    8.735
##
## Coefficients:
##                         Estimate Std. Error z value Pr(>|z|)
## (Intercept)             0.665874   0.017894  37.213   <2e-16 ***
## prop_day_trotline_cach -0.006707   0.017471  -0.384    0.701
## ---
## Signif. codes:  0 '***' 0.001 '**' 0.01 '*' 0.05 '.' 0.1 ' ' 1
##
## (Dispersion parameter for binomial family taken to be 1)
##
##     Null deviance: 3878.9  on 72  degrees of freedom
## Residual deviance: 3878.7  on 71  degrees of freedom
## AIC: 4248.7
##
## Number of Fisher Scoring iterations: 4
```

Calculate pseudo r squared

```
pseudo_r2(glm_1.4)

## [1] 3.79707e-05
```

Model with proportion of days in winter

```
glm_1.5 <- glm(cbind(count,count_total - count)~ prop_day_winter, family = bi
nomial(link = logit), data = datsc)
summary(glm_1.5)

##
## Call:
## glm(formula = cbind(count, count_total - count) ~ prop_day_winter,
##     family = binomial(link = logit), data = datsc)
##
## Deviance Residuals:
##     Min       1Q   Median       3Q      Max
## -16.622   -2.568    2.383    5.173   11.384
##
## Coefficients:
```

```
##                 Estimate Std. Error z value Pr(>|z|)
## (Intercept)      0.63184    0.01812   34.87   <2e-16 ***
## prop_day_winter -0.32774    0.02031  -16.14   <2e-16 ***
## ---
## Signif. codes:  0 '***' 0.001 '**' 0.01 '*' 0.05 '.' 0.1 ' ' 1
##
## (Dispersion parameter for binomial family taken to be 1)
##
##     Null deviance: 3878.9  on 72  degrees of freedom
## Residual deviance: 3617.3  on 71  degrees of freedom
## AIC: 3987.2
##
## Number of Fisher Scoring iterations: 4
```

Calculate pseudo r squared

```
pseudo_r2(glm_1.5)

## [1] 0.06744407
```

Model with density of vessels

```
glm_1.6 <- glm(cbind(count,count_total - count)~ density, family = binomial(l
ink = logit), data = datsc)
summary(glm_1.6)

##
## Call:
## glm(formula = cbind(count, count_total - count) ~ density, family = binomi
al(link = logit),
##     data = datsc)
##
## Deviance Residuals:
##     Min       1Q   Median       3Q      Max
## -17.096   -3.074    2.259    5.014    8.969
##
## Coefficients:
##             Estimate Std. Error z value Pr(>|z|)
## (Intercept)  0.79038    0.02013   39.26   <2e-16 ***
## density      0.40035    0.02408   16.63   <2e-16 ***
## ---
## Signif. codes:  0 '***' 0.001 '**' 0.01 '*' 0.05 '.' 0.1 ' ' 1
##
## (Dispersion parameter for binomial family taken to be 1)
##
##     Null deviance: 3878.9  on 72  degrees of freedom
## Residual deviance: 3568.7  on 71  degrees of freedom
## AIC: 3938.7
##
## Number of Fisher Scoring iterations: 4
```

Calculate pseudo r squared

```
pseudo_r2(glm_1.6)

## [1] 0.07996836
```

Model with the fishery

```
glm_1.7 <- glm(cbind(count,count_total - count)~ area, family = binomial(link
= logit), data = datsc)
summary(glm_1.7)

##
## Call:
## glm(formula = cbind(count, count_total - count) ~ area, family = binomial(
link = logit),
##     data = datsc)
##
## Deviance Residuals:
##      Min        1Q    Median        3Q       Max
## -12.1989   -1.8360   -0.0052    2.2468    6.8717
##
## Coefficients:
##                     Estimate Std. Error z value Pr(>|z|)
## (Intercept)          1.06659    0.04641  22.981  < 2e-16 ***
## areaCROZET           0.61539    0.07663   8.031 9.69e-16 ***
## areaFALKLANDS       -0.97600    0.06192 -15.762  < 2e-16 ***
## areaHIMI            -3.33255    0.10879 -30.632  < 2e-16 ***
## areaKERGUELEN        0.16642    0.06033   2.758  0.00581 **
## areaPEMI            -2.95113    0.16172 -18.249  < 2e-16 ***
## areaSOUTH GEORGIA  0.19358    0.07531   2.571  0.01015 *
## ---
## Signif. codes:  0 '***' 0.001 '**' 0.01 '*' 0.05 '.' 0.1 ' ' 1
##
## (Dispersion parameter for binomial family taken to be 1)
##
##     Null deviance: 3878.89  on 72  degrees of freedom
## Residual deviance:  760.81  on 66  degrees of freedom
## AIC: 1140.8
##
## Number of Fisher Scoring iterations: 5
```

Calculate pseudo r squared

```
pseudo_r2(glm_1.7)

## [1] 0.8038574
```

AIC summary

```
model.sel(list(glm_null, glm_1.1,glm_1.2,glm_1.3,glm_1.4,glm_1.5,glm_1.6,glm_
1.7))
```

```
## Model selection table
##    (Int) ttl_are men_dpt    mbl prp_day_trt_cch prp_day_wnt    dns are df
## 8 1.0670                                                             +  7
## 7 0.7904                                                    0.4004      2
## 6 0.6318                                            -0.3277             2
## 3 0.6954          -0.253                                                2
## 4 0.6600                 0.2477                                         2
## 2 0.6231  0.1876                                                        2
## 1 0.6657                                                                1
## 5 0.6659                              -0.006707                         2
##      logLik   AICc   delta weight
## 8  -563.388 1142.5    0.00      1
## 7 -1967.329 3938.8 2796.33      0
## 6 -1991.620 3987.4 2844.91      0
## 3 -2010.375 4024.9 2882.42      0
## 4 -2023.776 4051.7 2909.22      0
## 2 -2063.607 4131.4 2988.89      0
## 1 -2122.423 4246.9 3104.40      0
## 5 -2122.350 4248.9 3106.37      0
## Models ranked by AICc(x)
```

Model with the lowest AIC

```
glm_2.0 <- glm(cbind(count,count_total - count)~ area, family = binomial(link
= logit), data = datsc)
summary(glm_2.0)
```

```
##
## Call:
## glm(formula = cbind(count, count_total - count) ~ area, family = binomial(
link = logit),
##     data = datsc)
##
## Deviance Residuals:
##      Min        1Q    Median        3Q       Max
## -12.1989   -1.8360   -0.0052    2.2468    6.8717
##
## Coefficients:
##                   Estimate Std. Error z value Pr(>|z|)
## (Intercept)        1.06659    0.04641  22.981  < 2e-16 ***
## areaCROZET         0.61539    0.07663   8.031 9.69e-16 ***
## areaFALKLANDS     -0.97600    0.06192 -15.762  < 2e-16 ***
## areaHIMI          -3.33255    0.10879 -30.632  < 2e-16 ***
## areaKERGUELEN      0.16642    0.06033   2.758  0.00581 **
## areaPEMI          -2.95113    0.16172 -18.249  < 2e-16 ***
## areaSOUTH GEORGIA  0.19358    0.07531   2.571  0.01015 *
## ---
## Signif. codes:  0 '***' 0.001 '**' 0.01 '*' 0.05 '.' 0.1 ' ' 1
##
## (Dispersion parameter for binomial family taken to be 1)
```

```
##
##     Null deviance: 3878.89  on 72  degrees of freedom
## Residual deviance:  760.81  on 66  degrees of freedom
## AIC: 1140.8
##
## Number of Fisher Scoring iterations: 5
```

Calculate pseudo r squared

```
pseudo_r2(glm_2.0)

## [1] 0.8038574
```

Two terms models

Model with total area size

```
glm_2.1 <- glm(cbind(count,count_total - count)~ area+ total_area, family = b
inomial(link = logit), data = datsc)
summary(glm_2.1)

##
## Call:
## glm(formula = cbind(count, count_total - count) ~ area + total_area,
##     family = binomial(link = logit), data = datsc)
##
## Deviance Residuals:
##      Min        1Q    Median        3Q       Max
## -12.0316   -1.8242   -0.0901    2.4788    6.9533
##
## Coefficients:
##                    Estimate Std. Error z value Pr(>|z|)
## (Intercept)         1.07457    0.04716  22.787  < 2e-16 ***
## areaCROZET          0.63971    0.08060   7.937 2.07e-15 ***
## areaFALKLANDS      -0.96235    0.06348 -15.161  < 2e-16 ***
## areaHIMI           -3.35376    0.11111 -30.185  < 2e-16 ***
## areaKERGUELEN       0.06979    0.11636   0.600  0.54864
## areaPEMI           -2.91028    0.16707 -17.420  < 2e-16 ***
## areaSOUTH GEORGIA  0.19834    0.07546   2.628  0.00858 **
## total_area          0.04977    0.05127   0.971  0.33165
## ---
## Signif. codes:  0 '***' 0.001 '**' 0.01 '*' 0.05 '.' 0.1 ' ' 1
##
## (Dispersion parameter for binomial family taken to be 1)
##
##     Null deviance: 3878.89  on 72  degrees of freedom
## Residual deviance:  759.87  on 65  degrees of freedom
## AIC: 1141.8
##
## Number of Fisher Scoring iterations: 5
```

Calculate pseudo r squared

```
pseudo_r2(glm_2.1)

## [1] 0.8040999
```

Model with depth

```
glm_2.2 <- glm(cbind(count,count_total - count)~ area+ mean_depth, family = b
inomial(link = logit), data = datsc)
summary(glm_2.2)

##
## Call:
## glm(formula = cbind(count, count_total - count) ~ area + mean_depth,
##     family = binomial(link = logit), data = datsc)
##
## Deviance Residuals:
##     Min       1Q   Median       3Q      Max
## -7.5690  -1.5824  -0.0121   1.9968   6.6938
##
## Coefficients:
##                    Estimate Std. Error z value Pr(>|z|)
## (Intercept)         1.76970    0.08862  19.969  < 2e-16 ***
## areaCROZET         -0.44797    0.13608  -3.292 0.000995 ***
## areaFALKLANDS      -1.54425    0.08707 -17.735  < 2e-16 ***
## areaHIMI           -3.97214    0.13038 -30.465  < 2e-16 ***
## areaKERGUELEN      -0.75392    0.11457  -6.580 4.69e-11 ***
## areaPEMI           -3.51506    0.17318 -20.297  < 2e-16 ***
## areaSOUTH GEORGIA -0.58641    0.11232  -5.221 1.78e-07 ***
## mean_depth         -0.37874    0.03989  -9.495  < 2e-16 ***
## ---
## Signif. codes:  0 '***' 0.001 '**' 0.01 '*' 0.05 '.' 0.1 ' ' 1
##
## (Dispersion parameter for binomial family taken to be 1)
##
##     Null deviance: 3878.89  on 72  degrees of freedom
## Residual deviance:  670.39  on 65  degrees of freedom
## AIC: 1052.3
##
## Number of Fisher Scoring iterations: 5
```

Calculate pseudo r squared

```
pseudo_r2(glm_2.2)

## [1] 0.8271707
```

Model with mobility

```
glm_2.3 <- glm(cbind(count,count_total - count)~ area+ mobility, family = bin
omial(link = logit), data = datsc)
summary(glm_2.3)
```

```
##
## Call:
## glm(formula = cbind(count, count_total - count) ~ area + mobility,
##     family = binomial(link = logit), data = datsc)
##
## Deviance Residuals:
##      Min        1Q    Median        3Q       Max
## -11.6053   -1.4594   -0.5619    2.1234    6.7684
##
## Coefficients:
##                    Estimate Std. Error z value Pr(>|z|)
## (Intercept)         1.31015    0.05982  21.901  < 2e-16 ***
## areaCROZET          0.53185    0.07770   6.845 7.63e-12 ***
## areaFALKLANDS      -1.00305    0.06211 -16.149  < 2e-16 ***
## areaHIMI           -3.65926    0.12172 -30.064  < 2e-16 ***
## areaKERGUELEN      -0.43097    0.10914  -3.949 7.85e-05 ***
## areaPEMI           -2.86635    0.16224 -17.668  < 2e-16 ***
## areaSOUTH GEORGIA -0.25247    0.10165  -2.484    0.013 *
## mobility            0.28212    0.04349   6.487 8.77e-11 ***
## ---
## Signif. codes:  0 '***' 0.001 '**' 0.01 '*' 0.05 '.' 0.1 ' ' 1
##
## (Dispersion parameter for binomial family taken to be 1)
##
##     Null deviance: 3878.9  on 72  degrees of freedom
## Residual deviance:  718.5  on 65  degrees of freedom
## AIC: 1100.5
##
## Number of Fisher Scoring iterations: 5
```

Calculate pseudo r squared

```
pseudo_r2(glm_2.3)
```

```
## [1] 0.8147673
```

Model with proportion of sets using trotlines-cachalotera

```
glm_2.4 <- glm(cbind(count,count_total - count)~ area+ prop_day_trotline_cach
, family = binomial(link = logit), data = datsc)
summary(glm_2.4)
```

```
##
## Call:
## glm(formula = cbind(count, count_total - count) ~ area + prop_day_trotline
_cach,
##     family = binomial(link = logit), data = datsc)
```

```
##
## Deviance Residuals:
##      Min        1Q    Median        3Q       Max
## -12.2896   -1.6052    0.0111    2.2468    7.0150
##
## Coefficients:
##                          Estimate Std. Error z value Pr(>|z|)
## (Intercept)               0.72779    0.07796   9.335  < 2e-16 ***
## areaCROZET                1.08098    0.11526   9.378  < 2e-16 ***
## areaFALKLANDS            -0.76476    0.07341 -10.418  < 2e-16 ***
## areaHIMI                 -2.86695    0.13874 -20.664  < 2e-16 ***
## areaKERGUELEN             0.63201    0.10514   6.011 1.84e-09 ***
## areaPEMI                 -2.71687    0.16741 -16.229  < 2e-16 ***
## areaSOUTH GEORGIA         0.65917    0.11439   5.763 8.28e-09 ***
## prop_day_trotline_cach  0.19993    0.03699   5.405 6.49e-08 ***
## ---
## Signif. codes:  0 '***' 0.001 '**' 0.01 '*' 0.05 '.' 0.1 ' ' 1
##
## (Dispersion parameter for binomial family taken to be 1)
##
##     Null deviance: 3878.89  on 72  degrees of freedom
## Residual deviance:  731.44  on 65  degrees of freedom
## AIC: 1113.4
##
## Number of Fisher Scoring iterations: 5
```

Calculate pseudo r squared

```
pseudo_r2(glm_2.4)

## [1] 0.8114316
```

Model with proportion of days in winter

```
glm_2.5 <- glm(cbind(count,count_total - count)~ area+ prop_day_winter, famil
y = binomial(link = logit), data = datsc)
summary(glm_2.5)

##
## Call:
## glm(formula = cbind(count, count_total - count) ~ area + prop_day_winter,
##     family = binomial(link = logit), data = datsc)
##
## Deviance Residuals:
##      Min        1Q    Median        3Q       Max
## -12.0372   -1.8557    0.0144    2.3004    6.6633
##
## Coefficients:
##                    Estimate Std. Error z value Pr(>|z|)
## (Intercept)         0.95041    0.06398  14.854  < 2e-16 ***
## areaCROZET          0.68233    0.08101   8.423  < 2e-16 ***
```

```
## areaFALKLANDS     -0.87241    0.07333 -11.897  < 2e-16 ***
## areaHIMI          -3.13320    0.13250 -23.648  < 2e-16 ***
## areaKERGUELEN      0.21343    0.06290   3.393 0.000691 ***
## areaPEMI          -2.80798    0.17031 -16.488  < 2e-16 ***
## areaSOUTH GEORGIA  0.51053    0.14228   3.588 0.000333 ***
## prop_day_winter   -0.12546    0.04779  -2.625 0.008665 **
## ---
## Signif. codes:  0 '***' 0.001 '**' 0.01 '*' 0.05 '.' 0.1 ' ' 1
##
## (Dispersion parameter for binomial family taken to be 1)
##
##     Null deviance: 3878.89  on 72  degrees of freedom
## Residual deviance:  753.94  on 65  degrees of freedom
## AIC: 1135.9
##
## Number of Fisher Scoring iterations: 5
```

Calculate pseudo r squared

```
pseudo_r2(glm_2.5)

## [1] 0.8056309
```

Model with density of vessels

```
glm_2.6 <- glm(cbind(count,count_total - count)~ area+ density, family = bino
mial(link = logit), data = datsc)
summary(glm_2.6)

##
## Call:
## glm(formula = cbind(count, count_total - count) ~ area + density,
##     family = binomial(link = logit), data = datsc)
##
## Deviance Residuals:
##     Min       1Q   Median       3Q      Max
## -12.496   -1.756   -0.030    2.106    6.786
##
## Coefficients:
##                   Estimate Std. Error z value Pr(>|z|)
## (Intercept)        1.13988    0.04785  23.823  < 2e-16 ***
## areaCROZET         0.48701    0.07889   6.174 6.68e-10 ***
## areaFALKLANDS     -0.78613    0.06823 -11.521  < 2e-16 ***
## areaHIMI          -3.18564    0.11090 -28.725  < 2e-16 ***
## areaKERGUELEN      0.37361    0.06768   5.520 3.38e-08 ***
## areaPEMI          -3.04210    0.16305 -18.657  < 2e-16 ***
## areaSOUTH GEORGIA -0.36223    0.11114  -3.259  0.00112 **
## density            0.33084    0.04954   6.678 2.42e-11 ***
## ---
## Signif. codes:  0 '***' 0.001 '**' 0.01 '*' 0.05 '.' 0.1 ' ' 1
##
```

```
## (Dispersion parameter for binomial family taken to be 1)
##
##     Null deviance: 3878.89  on 72  degrees of freedom
## Residual deviance:  714.42  on 65  degrees of freedom
## AIC: 1096.4
##
## Number of Fisher Scoring iterations: 5
```

Calculate pseudo r squared

```
pseudo_r2(glm_2.6)

## [1] 0.8158182
```

AIC summary

```
model.sel(list(glm_2.0,glm_2.1,glm_2.2,glm_2.3,glm_2.4,glm_2.5,glm_2.6))

## Model selection table
##    (Int) are ttl_are men_dpt    mbl prp_day_trt_cch prp_day_wnt    dns df
## 3 1.7700   +         -0.3787                                            8
## 7 1.1400   +                                                   0.3308  8
## 4 1.3100   +                 0.2821                                     8
## 5 0.7278   +                                0.1999                      8
## 6 0.9504   +                                           -0.1255          8
## 1 1.0670   +                                                            7
## 2 1.0750   + 0.04977                                                    8
##     logLik   AICc delta weight
## 3 -518.173 1054.6  0.00      1
## 7 -540.191 1098.6 44.04      0
## 4 -542.229 1102.7 48.11      0
## 5 -548.698 1115.6 61.05      0
## 6 -559.948 1138.1 83.55      0
## 1 -563.388 1142.5 87.90      0
## 2 -562.918 1144.1 89.49      0
## Models ranked by AICc(x)
```

Model with the lowest AIC

```
glm_3.0 <- glm(cbind(count,count_total - count)~ area+ mean_depth, family = b
inomial(link = logit), data = datsc)
summary(glm_3.0)

##
## Call:
## glm(formula = cbind(count, count_total - count) ~ area + mean_depth,
##     family = binomial(link = logit), data = datsc)
##
## Deviance Residuals:
##     Min       1Q   Median       3Q      Max
## -7.5690  -1.5824  -0.0121   1.9968   6.6938
##
```

```
## Coefficients:
##                    Estimate Std. Error z value Pr(>|z|)
## (Intercept)         1.76970    0.08862  19.969  < 2e-16 ***
## areaCROZET         -0.44797    0.13608  -3.292 0.000995 ***
## areaFALKLANDS      -1.54425    0.08707 -17.735  < 2e-16 ***
## areaHIMI           -3.97214    0.13038 -30.465  < 2e-16 ***
## areaKERGUELEN      -0.75392    0.11457  -6.580 4.69e-11 ***
## areaPEMI           -3.51506    0.17318 -20.297  < 2e-16 ***
## areaSOUTH GEORGIA -0.58641    0.11232  -5.221 1.78e-07 ***
## mean_depth         -0.37874    0.03989  -9.495  < 2e-16 ***
## ---
## Signif. codes:  0 '***' 0.001 '**' 0.01 '*' 0.05 '.' 0.1 ' ' 1
##
## (Dispersion parameter for binomial family taken to be 1)
##
##     Null deviance: 3878.89  on 72  degrees of freedom
## Residual deviance:  670.39  on 65  degrees of freedom
## AIC: 1052.3
##
## Number of Fisher Scoring iterations: 5
```

Calculate pseudo r squared

```
pseudo_r2(glm_3.0)

## [1] 0.8271707
```

Three terms models

Model with total area size

```
glm_3.1 <- glm(cbind(count,count_total - count)~ area+ mean_depth+ total_area
, family = binomial(link = logit), data = datsc)
summary(glm_3.1)

##
## Call:
## glm(formula = cbind(count, count_total - count) ~ area + mean_depth +
##     total_area, family = binomial(link = logit), data = datsc)
##
## Deviance Residuals:
##     Min       1Q   Median       3Q      Max
## -7.1547  -1.5214  -0.0057   1.7929   6.7807
##
## Coefficients:
##                    Estimate Std. Error z value Pr(>|z|)
## (Intercept)         1.81777    0.09121  19.929  < 2e-16 ***
## areaCROZET         -0.42794    0.13643  -3.137  0.00171 **
## areaFALKLANDS      -1.53302    0.08732 -17.555  < 2e-16 ***
## areaHIMI           -4.05992    0.13659 -29.722  < 2e-16 ***
## areaKERGUELEN      -1.03586    0.16515  -6.272 3.56e-10 ***
```

```
## areaPEMI          -3.43339    0.17639 -19.465  < 2e-16 ***
## areaSOUTH GEORGIA -0.60449    0.11272  -5.363 8.20e-08 ***
## mean_depth        -0.39231    0.04028  -9.738  < 2e-16 ***
## total_area         0.12547    0.05255   2.388  0.01696 *
## ---
## Signif. codes:  0 '***' 0.001 '**' 0.01 '*' 0.05 '.' 0.1 ' ' 1
##
## (Dispersion parameter for binomial family taken to be 1)
##
##     Null deviance: 3878.9  on 72  degrees of freedom
## Residual deviance:  664.7  on 64  degrees of freedom
## AIC: 1048.7
##
## Number of Fisher Scoring iterations: 5
```

Calculate pseudo r squared

```
pseudo_r2(glm_3.1)

## [1] 0.8286359
```

Model with density of vessels

```
glm_3.2 <- glm(cbind(count,count_total - count)~ area+ mean_depth+ density, f
amily = binomial(link = logit), data = datsc)
summary(glm_3.2)

##
## Call:
## glm(formula = cbind(count, count_total - count) ~ area + mean_depth +
##     density, family = binomial(link = logit), data = datsc)
##
## Deviance Residuals:
##    Min      1Q  Median      3Q     Max
## -8.278  -1.652   0.060   1.871   6.973
##
## Coefficients:
##                   Estimate Std. Error z value Pr(>|z|)
## (Intercept)        1.75041    0.08842  19.796  < 2e-16 ***
## areaCROZET        -0.43277    0.13579  -3.187  0.00144 **
## areaFALKLANDS     -1.33878    0.09581 -13.973  < 2e-16 ***
## areaHIMI          -3.79015    0.13466 -28.146  < 2e-16 ***
## areaKERGUELEN     -0.49809    0.12477  -3.992 6.55e-05 ***
## areaPEMI          -3.53188    0.17424 -20.270  < 2e-16 ***
## areaSOUTH GEORGIA -0.92306    0.13004  -7.098 1.26e-12 ***
## mean_depth        -0.33935    0.04059  -8.360  < 2e-16 ***
## density            0.25244    0.05026   5.022 5.10e-07 ***
## ---
## Signif. codes:  0 '***' 0.001 '**' 0.01 '*' 0.05 '.' 0.1 ' ' 1
##
## (Dispersion parameter for binomial family taken to be 1)
```

```
##
##     Null deviance: 3878.89  on 72  degrees of freedom
## Residual deviance:  644.54  on 64  degrees of freedom
## AIC: 1028.5
##
## Number of Fisher Scoring iterations: 5
```

Calculate pseudo r squared

```
pseudo_r2(glm_3.2)

## [1] 0.8338347
```

Model with proportion of days in winter

```
glm_3.3 <- glm(cbind(count,count_total - count)~ area+ mean_depth+ prop_day_w
inter, family = binomial(link = logit), data = datsc)
summary(glm_3.3)

##
## Call:
## glm(formula = cbind(count, count_total - count) ~ area + mean_depth +
##     prop_day_winter, family = binomial(link = logit), data = datsc)
##
## Deviance Residuals:
##     Min       1Q   Median       3Q      Max
## -7.4142  -1.5819  -0.1231   2.0415   6.8002
##
## Coefficients:
##                     Estimate Std. Error z value Pr(>|z|)
## (Intercept)          1.65417    0.09935  16.650  < 2e-16 ***
## areaCROZET          -0.37872    0.13874  -2.730  0.00634 **
## areaFALKLANDS       -1.44138    0.09584 -15.040  < 2e-16 ***
## areaHIMI            -3.77678    0.15111 -24.994  < 2e-16 ***
## areaKERGUELEN       -0.70545    0.11603  -6.080  1.2e-09 ***
## areaPEMI            -3.38032    0.18115 -18.660  < 2e-16 ***
## areaSOUTH GEORGIA -0.27652    0.16565  -1.669  0.09505 .
## mean_depth          -0.37765    0.03987  -9.472  < 2e-16 ***
## prop_day_winter     -0.12182    0.04793  -2.541  0.01104 *
## ---
## Signif. codes:  0 '***' 0.001 '**' 0.01 '*' 0.05 '.' 0.1 ' ' 1
##
## (Dispersion parameter for binomial family taken to be 1)
##
##     Null deviance: 3878.89  on 72  degrees of freedom
## Residual deviance:  663.93  on 64  degrees of freedom
## AIC: 1047.9
##
## Number of Fisher Scoring iterations: 5
```

Calculate pseudo r squared

```r
pseudo_r2(glm_3.3)

## [1] 0.8288352
```

Model with proportion of sets using trotlines-cachalotera

```r
glm_3.4 <- glm(cbind(count,count_total - count)~ area+ mean_depth+ prop_day_t
rotline_cach, family = binomial(link = logit), data = datsc)
summary(glm_3.4)

##
## Call:
## glm(formula = cbind(count, count_total - count) ~ area + mean_depth +
##     prop_day_trotline_cach, family = binomial(link = logit),
##     data = datsc)
##
## Deviance Residuals:
##     Min       1Q   Median       3Q      Max
## -7.3879  -1.7410  -0.0316   2.0178   6.3678
##
## Coefficients:
##                         Estimate Std. Error z value Pr(>|z|)
## (Intercept)             1.41297    0.10473  13.491  < 2e-16 ***
## areaCROZET              0.03684    0.15565   0.237   0.8129
## areaFALKLANDS          -1.33142    0.09350 -14.240  < 2e-16 ***
## areaHIMI               -3.46324    0.15312 -22.618  < 2e-16 ***
## areaKERGUELEN          -0.26021    0.13813  -1.884   0.0596 .
## areaPEMI               -3.27434    0.17730 -18.468  < 2e-16 ***
## areaSOUTH GEORGIA      -0.08440    0.13716  -0.615   0.5383
## mean_depth             -0.40231    0.03999 -10.060  < 2e-16 ***
## prop_day_trotline_cach  0.23635    0.03739   6.321 2.61e-10 ***
## ---
## Signif. codes:  0 '***' 0.001 '**' 0.01 '*' 0.05 '.' 0.1 ' ' 1
##
## (Dispersion parameter for binomial family taken to be 1)
##
##     Null deviance: 3878.89  on 72  degrees of freedom
## Residual deviance:  630.13  on 64  degrees of freedom
## AIC: 1014.1
##
## Number of Fisher Scoring iterations: 5
```

Calculate pseudo r squared

```r
pseudo_r2(glm_3.4)

## [1] 0.8375497
```

Model with mobility

```
glm_3.5 <- glm(cbind(count,count_total - count)~ area+ mean_depth+mobility, f
amily = binomial(link = logit), data = datsc)
summary(glm_3.5)
```

```
##
## Call:
## glm(formula = cbind(count, count_total - count) ~ area + mean_depth +
##     mobility, family = binomial(link = logit), data = datsc)
##
## Deviance Residuals:
##     Min       1Q   Median       3Q      Max
## -7.6135  -1.5965  -0.0514   1.8296   6.1313
##
## Coefficients:
##                    Estimate Std. Error z value Pr(>|z|)
## (Intercept)         2.29032    0.10822  21.163  < 2e-16 ***
## areaCROZET         -0.81870    0.14386  -5.691 1.26e-08 ***
## areaFALKLANDS      -1.72065    0.09043 -19.028  < 2e-16 ***
## areaHIMI           -4.64029    0.15649 -29.653  < 2e-16 ***
## areaKERGUELEN      -1.85161    0.17095 -10.831  < 2e-16 ***
## areaPEMI           -3.53416    0.17402 -20.309  < 2e-16 ***
## areaSOUTH GEORGIA -1.40526    0.14629  -9.606  < 2e-16 ***
## mean_depth         -0.46488    0.04135 -11.243  < 2e-16 ***
## mobility            0.40895    0.04616   8.859  < 2e-16 ***
## ---
## Signif. codes:  0 '***' 0.001 '**' 0.01 '*' 0.05 '.' 0.1 ' ' 1
##
## (Dispersion parameter for binomial family taken to be 1)
##
##     Null deviance: 3878.89  on 72  degrees of freedom
## Residual deviance:  590.53  on 64  degrees of freedom
## AIC: 974.49
##
## Number of Fisher Scoring iterations: 5
```

Calculate pseudo r squared

```
pseudo_r2(glm_3.5)
```

```
## [1] 0.8477577
```

AIC summary

```
model.sel(list(glm_3.0, glm_3.1,glm_3.2,glm_3.3,glm_3.4,glm_3.5))
```

```
## Model selection table
##   (Int) are men_dpt ttl_are    dns prp_day_wnt prp_day_trt_cch    mbl df
## 6 2.290   + -0.4649                                            0.4089  9
## 5 1.413   + -0.4023                                     0.2363          9
## 3 1.750   + -0.3394          0.2524                                     9
## 4 1.654   + -0.3776                      -0.1218                        9
```

```
## 2 1.818   + -0.3923  0.1255                                           9
## 1 1.770   + -0.3787                                                   8
##     logLik   AICc delta weight
## 6 -478.246  977.3  0.00      1
## 5 -498.044 1016.9 39.60      0
## 3 -505.249 1031.4 54.01      0
## 4 -514.945 1050.7 73.40      0
## 2 -515.332 1051.5 74.17      0
## 1 -518.173 1054.6 77.25      0
## Models ranked by AICc(x)
```

Model with the lowest AIC

```
glm_4.0 <- glm(cbind(count,count_total - count)~ area+ mean_depth+mobility, f
amily = binomial(link = logit), data = datsc)
summary(glm_4.0)
```

```
##
## Call:
## glm(formula = cbind(count, count_total - count) ~ area + mean_depth +
##     mobility, family = binomial(link = logit), data = datsc)
##
## Deviance Residuals:
##     Min       1Q   Median       3Q      Max
## -7.6135  -1.5965  -0.0514   1.8296   6.1313
##
## Coefficients:
##                    Estimate Std. Error z value Pr(>|z|)
## (Intercept)         2.29032    0.10822  21.163  < 2e-16 ***
## areaCROZET         -0.81870    0.14386  -5.691 1.26e-08 ***
## areaFALKLANDS      -1.72065    0.09043 -19.028  < 2e-16 ***
## areaHIMI           -4.64029    0.15649 -29.653  < 2e-16 ***
## areaKERGUELEN      -1.85161    0.17095 -10.831  < 2e-16 ***
## areaPEMI           -3.53416    0.17402 -20.309  < 2e-16 ***
## areaSOUTH GEORGIA -1.40526    0.14629  -9.606  < 2e-16 ***
## mean_depth         -0.46488    0.04135 -11.243  < 2e-16 ***
## mobility            0.40895    0.04616   8.859  < 2e-16 ***
## ---
## Signif. codes:  0 '***' 0.001 '**' 0.01 '*' 0.05 '.' 0.1 ' ' 1
##
## (Dispersion parameter for binomial family taken to be 1)
##
##     Null deviance: 3878.89  on 72  degrees of freedom
## Residual deviance:  590.53  on 64  degrees of freedom
## AIC: 974.49
##
## Number of Fisher Scoring iterations: 5
```

Calculate pseudo r squared

```
pseudo_r2(glm_4.0)
```

```
## [1] 0.8477577
```

Four terms models

Model with proportion of days in winter

```
glm_4.1 <- glm(cbind(count,count_total - count)~ area+ mean_depth+mobility+ p
rop_day_winter, family = binomial(link = logit), data = datsc)
summary(glm_4.1)
```

```
##
## Call:
## glm(formula = cbind(count, count_total - count) ~ area + mean_depth +
##     mobility + prop_day_winter, family = binomial(link = logit),
##     data = datsc)
##
## Deviance Residuals:
##     Min       1Q   Median       3Q      Max
## -7.4549  -1.5342  -0.0126   1.7470   6.2529
##
## Coefficients:
##                   Estimate Std. Error z value Pr(>|z|)
## (Intercept)        2.15776    0.11516  18.737  < 2e-16 ***
## areaCROZET        -0.74245    0.14577  -5.093 3.52e-07 ***
## areaFALKLANDS     -1.59269    0.09800 -16.253  < 2e-16 ***
## areaHIMI          -4.41669    0.17065 -25.881  < 2e-16 ***
## areaKERGUELEN     -1.82745    0.17137 -10.664  < 2e-16 ***
## areaPEMI          -3.36308    0.18164 -18.515  < 2e-16 ***
## areaSOUTH GEORGIA -1.02883    0.18449  -5.576 2.45e-08 ***
## mean_depth        -0.46714    0.04138 -11.289  < 2e-16 ***
## mobility           0.42309    0.04643   9.113  < 2e-16 ***
## prop_day_winter   -0.16155    0.04814  -3.356 0.000792 ***
## ---
## Signif. codes:  0 '***' 0.001 '**' 0.01 '*' 0.05 '.' 0.1 ' ' 1
##
## (Dispersion parameter for binomial family taken to be 1)
##
##     Null deviance: 3878.89  on 72  degrees of freedom
## Residual deviance:  579.29  on 63  degrees of freedom
## AIC: 965.25
##
## Number of Fisher Scoring iterations: 5
```

Calculate pseudo r squared

```
pseudo_r2(glm_4.1)
```

```
## [1] 0.850656
```

Model with density of vessels

```
glm_4.2 <- glm(cbind(count,count_total - count)~ area+ mean_depth+mobility+de
nsity, family = binomial(link = logit), data = datsc)
summary(glm_4.2)

##
## Call:
## glm(formula = cbind(count, count_total - count) ~ area + mean_depth +
##     mobility + density, family = binomial(link = logit), data = datsc)
##
## Deviance Residuals:
##     Min       1Q   Median       3Q      Max
## -7.0998  -1.4121   0.0559   1.7984   6.4095
##
## Coefficients:
##                    Estimate Std. Error z value Pr(>|z|)
## (Intercept)         2.27010    0.10865  20.894  < 2e-16 ***
## areaCROZET         -0.80129    0.14372  -5.575 2.47e-08 ***
## areaFALKLANDS      -1.53133    0.09873 -15.511  < 2e-16 ***
## areaHIMI           -4.45632    0.16067 -27.736  < 2e-16 ***
## areaKERGUELEN      -1.60097    0.17947  -8.920  < 2e-16 ***
## areaPEMI           -3.55872    0.17527 -20.305  < 2e-16 ***
## areaSOUTH GEORGIA -1.70178    0.15980 -10.650  < 2e-16 ***
## mean_depth         -0.42996    0.04199 -10.239  < 2e-16 ***
## mobility            0.40178    0.04651   8.639  < 2e-16 ***
## density             0.23674    0.05115   4.628 3.69e-06 ***
## ---
## Signif. codes:  0 '***' 0.001 '**' 0.01 '*' 0.05 '.' 0.1 ' ' 1
##
## (Dispersion parameter for binomial family taken to be 1)
##
##     Null deviance: 3878.9  on 72  degrees of freedom
## Residual deviance:  568.8  on 63  degrees of freedom
## AIC: 954.76
##
## Number of Fisher Scoring iterations: 5
```

Calculate pseudo r squared

```
pseudo_r2(glm_4.2)

## [1] 0.8533608
```

Model with proportion of sets using trotlines-cachalotera

```
glm_4.3 <- glm(cbind(count,count_total - count)~ area+ mean_depth+mobility+pr
op_day_trotline_cach, family = binomial(link = logit), data = datsc)
summary(glm_4.3)

##
## Call:
## glm(formula = cbind(count, count_total - count) ~ area + mean_depth +
```

```
##     mobility + prop_day_trotline_cach, family = binomial(link = logit),
##     data = datsc)
##
## Deviance Residuals:
##     Min       1Q   Median       3Q      Max
## -6.2057  -1.5646  -0.0441   2.0020   5.5930
##
## Coefficients:
##                        Estimate Std. Error z value Pr(>|z|)
## (Intercept)             1.93892    0.12330  15.725  < 2e-16 ***
## areaCROZET             -0.35352    0.16376  -2.159   0.0309 *
## areaFALKLANDS          -1.51614    0.09707 -15.618  < 2e-16 ***
## areaHIMI               -4.14230    0.17768 -23.313  < 2e-16 ***
## areaKERGUELEN          -1.35277    0.19039  -7.105 1.20e-12 ***
## areaPEMI               -3.30960    0.17817 -18.575  < 2e-16 ***
## areaSOUTH GEORGIA      -0.90825    0.16868  -5.384 7.27e-08 ***
## mean_depth             -0.48218    0.04132 -11.669  < 2e-16 ***
## mobility                0.39373    0.04622   8.519  < 2e-16 ***
## prop_day_trotline_cach  0.21840    0.03754   5.818 5.95e-09 ***
## ---
## Signif. codes:  0 '***' 0.001 '**' 0.01 '*' 0.05 '.' 0.1 ' ' 1
##
## (Dispersion parameter for binomial family taken to be 1)
##
##     Null deviance: 3878.89  on 72  degrees of freedom
## Residual deviance:  556.44  on 63  degrees of freedom
## AIC: 942.4
##
## Number of Fisher Scoring iterations: 5
```

Calculate pseudo r squared

```
pseudo_r2(glm_4.3)

## [1] 0.8565475
```

Model with total area size

```
glm_4.4 <- glm(cbind(count,count_total - count)~ area+ mean_depth+mobility+ t
otal_area, family = binomial(link = logit), data = datsc)
summary(glm_4.4)

##
## Call:
## glm(formula = cbind(count, count_total - count) ~ area + mean_depth +
##     mobility + total_area, family = binomial(link = logit), data = datsc)
##
## Deviance Residuals:
##     Min       1Q   Median       3Q      Max
## -7.5092  -1.4741   0.1645   1.6031   5.5045
##
```

```
## Coefficients:
##                    Estimate Std. Error z value Pr(>|z|)
## (Intercept)         2.48462    0.11503  21.600  < 2e-16 ***
## areaCROZET         -1.14058    0.15573  -7.324 2.41e-13 ***
## areaFALKLANDS      -1.88682    0.09590 -19.676  < 2e-16 ***
## areaHIMI           -4.75699    0.15753 -30.198  < 2e-16 ***
## areaKERGUELEN      -1.63368    0.17613  -9.275  < 2e-16 ***
## areaPEMI           -3.84567    0.18412 -20.887  < 2e-16 ***
## areaSOUTH GEORGIA -1.90299    0.17272 -11.018  < 2e-16 ***
## mean_depth         -0.47584    0.04156 -11.450  < 2e-16 ***
## mobility            0.68593    0.06831  10.042  < 2e-16 ***
## total_area         -0.43448    0.07685  -5.654 1.57e-08 ***
## ---
## Signif. codes:  0 '***' 0.001 '**' 0.01 '*' 0.05 '.' 0.1 ' ' 1
##
## (Dispersion parameter for binomial family taken to be 1)
##
##     Null deviance: 3878.89  on 72  degrees of freedom
## Residual deviance:  558.02  on 63  degrees of freedom
## AIC: 943.98
##
## Number of Fisher Scoring iterations: 5
```

Calculate pseudo r squared

```
pseudo_r2(glm_4.4)

## [1] 0.8561401
```

AIC summary

```
model.sel(list(glm_4.0,glm_4.1,glm_4.2,glm_4.3,glm_4.4))

## Model selection table
##   (Int) are men_dpt    mbl prp_day_wnt    dns prp_day_trt_cch ttl_are df
## 4 1.939   + -0.4822 0.3937                             0.2184         10
## 5 2.485   + -0.4758 0.6859                                    -0.4345 10
## 3 2.270   + -0.4300 0.4018             0.2367                         10
## 2 2.158   + -0.4671 0.4231     -0.1616                                10
## 1 2.290   + -0.4649 0.4089                                             9
##     logLik  AICc delta weight
## 4 -461.199 945.9  0.00  0.687
## 5 -461.989 947.5  1.58  0.312
## 3 -467.379 958.3 12.36  0.001
## 2 -472.625 968.8 22.85  0.000
## 1 -478.246 977.3 31.40  0.000
## Models ranked by AICc(x)
```

Model with the lowest AIC

```
glm_5.0 <- glm(cbind(count,count_total - count)~ area+ mean_depth+mobility+pr
op_day_trotline_cach, family = binomial(link = logit), data = datsc)
summary(glm_5.0)
```

```
##
## Call:
## glm(formula = cbind(count, count_total - count) ~ area + mean_depth +
##     mobility + prop_day_trotline_cach, family = binomial(link = logit),
##     data = datsc)
##
## Deviance Residuals:
##     Min       1Q   Median       3Q      Max
## -6.2057  -1.5646  -0.0441   2.0020   5.5930
##
## Coefficients:
##                        Estimate Std. Error z value Pr(>|z|)
## (Intercept)             1.93892    0.12330  15.725  < 2e-16 ***
## areaCROZET             -0.35352    0.16376  -2.159   0.0309 *
## areaFALKLANDS          -1.51614    0.09707 -15.618  < 2e-16 ***
## areaHIMI               -4.14230    0.17768 -23.313  < 2e-16 ***
## areaKERGUELEN          -1.35277    0.19039  -7.105 1.20e-12 ***
## areaPEMI               -3.30960    0.17817 -18.575  < 2e-16 ***
## areaSOUTH GEORGIA      -0.90825    0.16868  -5.384 7.27e-08 ***
## mean_depth             -0.48218    0.04132 -11.669  < 2e-16 ***
## mobility                0.39373    0.04622   8.519  < 2e-16 ***
## prop_day_trotline_cach  0.21840    0.03754   5.818 5.95e-09 ***
## ---
## Signif. codes:  0 '***' 0.001 '**' 0.01 '*' 0.05 '.' 0.1 ' ' 1
##
## (Dispersion parameter for binomial family taken to be 1)
##
##     Null deviance: 3878.89  on 72  degrees of freedom
## Residual deviance:  556.44  on 63  degrees of freedom
## AIC: 942.4
##
## Number of Fisher Scoring iterations: 5
```

Calculate pseudo r squared

```
pseudo_r2(glm_5.0)

## [1] 0.8565475
```

Five terms models

Model with proportion of days in winter

```
glm_5.1 <- glm(cbind(count,count_total - count)~ area+ mean_depth+mobility+pr
op_day_trotline_cach+ prop_day_winter, family = binomial(link = logit), data
= datsc)
summary(glm_5.1)
```

```
##
## Call:
## glm(formula = cbind(count, count_total - count) ~ area + mean_depth +
##     mobility + prop_day_trotline_cach + prop_day_winter, family = binomial
(link = logit),
##     data = datsc)
##
## Deviance Residuals:
##     Min       1Q   Median       3Q      Max
## -5.8430  -1.7591   0.0269   2.1005   5.5162
##
## Coefficients:
##                         Estimate Std. Error z value Pr(>|z|)
## (Intercept)              1.73270    0.13249  13.078  < 2e-16 ***
## areaCROZET              -0.20640    0.16761  -1.231   0.2181
## areaFALKLANDS           -1.33101    0.10632 -12.519  < 2e-16 ***
## areaHIMI                -3.80393    0.19544 -19.463  < 2e-16 ***
## areaKERGUELEN           -1.26985    0.19167  -6.625 3.47e-11 ***
## areaPEMI                -3.07064    0.18728 -16.396  < 2e-16 ***
## areaSOUTH GEORGIA       -0.37433    0.21072  -1.776   0.0757 .
## mean_depth              -0.48729    0.04139 -11.774  < 2e-16 ***
## mobility                 0.41008    0.04647   8.825  < 2e-16 ***
## prop_day_trotline_cach  0.24136    0.03793   6.363 1.98e-10 ***
## prop_day_winter         -0.20669    0.04872  -4.242 2.22e-05 ***
## ---
## Signif. codes:  0 '***' 0.001 '**' 0.01 '*' 0.05 '.' 0.1 ' ' 1
##
## (Dispersion parameter for binomial family taken to be 1)
##
##     Null deviance: 3878.89  on 72  degrees of freedom
## Residual deviance:  538.48  on 62  degrees of freedom
## AIC: 926.45
##
## Number of Fisher Scoring iterations: 5
```

Calculate pseudo r squared

```
pseudo_r2(glm_5.1)

## [1] 0.8611754
```

Model with density of vessels

```
glm_5.2 <- glm(cbind(count,count_total - count)~ area+ mean_depth+mobility+pr
op_day_trotline_cach+density, family = binomial(link = logit), data = datsc)
summary(glm_5.2)

##
## Call:
## glm(formula = cbind(count, count_total - count) ~ area + mean_depth +
##     mobility + prop_day_trotline_cach + density, family = binomial(link =
```

```
logit),
##     data = datsc)
##
## Deviance Residuals:
##     Min       1Q   Median       3Q      Max
## -6.1036  -1.4936   0.0744   1.9811   5.2886
##
## Coefficients:
##                          Estimate Std. Error z value Pr(>|z|)
## (Intercept)               1.96447    0.12403  15.838  < 2e-16 ***
## areaCROZET               -0.39637    0.16426  -2.413 0.015817 *
## areaFALKLANDS            -1.39212    0.10286 -13.534  < 2e-16 ***
## areaHIMI                 -4.05735    0.17908 -22.657  < 2e-16 ***
## areaKERGUELEN            -1.21506    0.19489  -6.234 4.53e-10 ***
## areaPEMI                 -3.35497    0.17955 -18.686  < 2e-16 ***
## areaSOUTH GEORGIA        -1.20006    0.18774  -6.392 1.63e-10 ***
## mean_depth               -0.45253    0.04215 -10.737  < 2e-16 ***
## mobility                  0.38916    0.04652   8.365  < 2e-16 ***
## prop_day_trotline_cach    0.19156    0.03825   5.008 5.51e-07 ***
## density                   0.18521    0.05198   3.563 0.000367 ***
## ---
## Signif. codes:  0 '***' 0.001 '**' 0.01 '*' 0.05 '.' 0.1 ' ' 1
##
## (Dispersion parameter for binomial family taken to be 1)
##
##     Null deviance: 3878.89  on 72  degrees of freedom
## Residual deviance:  543.57  on 62  degrees of freedom
## AIC: 931.53
##
## Number of Fisher Scoring iterations: 5
```

Calculate pseudo r squared

```
pseudo_r2(glm_5.2)

## [1] 0.8598655
```

Model with total area size

```
glm_5.3 <- glm(cbind(count,count_total - count)~ area+ mean_depth+mobility+pr
op_day_trotline_cach+total_area, family = binomial(link = logit), data = dats
c)
summary(glm_5.3)

##
## Call:
## glm(formula = cbind(count, count_total - count) ~ area + mean_depth +
##     mobility + prop_day_trotline_cach + total_area, family = binomial(link
= logit),
##     data = datsc)
##
```

```
## Deviance Residuals:
##     Min       1Q   Median       3Q      Max
## -6.2348  -1.5626  -0.0534   1.8307   5.3668
##
## Coefficients:
##                        Estimate Std. Error z value Pr(>|z|)
## (Intercept)             2.16034    0.13718  15.748  < 2e-16 ***
## areaCROZET             -0.69648    0.18677  -3.729 0.000192 ***
## areaFALKLANDS          -1.68320    0.10682 -15.758  < 2e-16 ***
## areaHIMI               -4.34035    0.18538 -23.413  < 2e-16 ***
## areaKERGUELEN          -1.30341    0.19228  -6.779 1.21e-11 ***
## areaPEMI               -3.58863    0.19301 -18.593  < 2e-16 ***
## areaSOUTH GEORGIA      -1.38778    0.20987  -6.613 3.77e-11 ***
## mean_depth             -0.48521    0.04145 -11.705  < 2e-16 ***
## mobility                0.60090    0.07057   8.514  < 2e-16 ***
## prop_day_trotline_cach  0.16667    0.03987   4.180 2.91e-05 ***
## total_area             -0.32077    0.08095  -3.963 7.41e-05 ***
## ---
## Signif. codes:  0 '***' 0.001 '**' 0.01 '*' 0.05 '.' 0.1 ' ' 1
##
## (Dispersion parameter for binomial family taken to be 1)
##
##     Null deviance: 3878.89  on 72  degrees of freedom
## Residual deviance:  540.51  on 62  degrees of freedom
## AIC: 928.47
##
## Number of Fisher Scoring iterations: 5
```

Calculate pseudo r squared

```
pseudo_r2(glm_5.3)

## [1] 0.860654
```

AIC summary

```
model.sel(list(glm_5.0,glm_5.1,glm_5.2,glm_5.3))

## Model selection table
##   (Int) are men_dpt    mbl prp_day_trt_cch prp_day_wnt    dns ttl_are df
## 2 1.733   + -0.4873 0.4101          0.2414     -0.2067                11
## 4 2.160   + -0.4852 0.6009          0.1667                    -0.3208 11
## 3 1.964   + -0.4525 0.3892          0.1916             0.1852         11
## 1 1.939   + -0.4822 0.3937          0.2184                            10
##     logLik  AICc delta weight
## 2 -452.223 930.8  0.00  0.693
## 4 -453.234 932.8  2.02  0.252
## 3 -454.764 935.9  5.08  0.055
## 1 -461.199 945.9 15.17  0.000
## Models ranked by AICc(x)
```

Model with the lowest AIC

```
glm_6.0 <- glm(cbind(count,count_total - count)~ area+ mean_depth+mobility+pr
op_day_trotline_cach+prop_day_winter, family = binomial(link = logit), data =
datsc)
summary(glm_6.0)
```

```
##
## Call:
## glm(formula = cbind(count, count_total - count) ~ area + mean_depth +
##     mobility + prop_day_trotline_cach + prop_day_winter, family = binomial
(link = logit),
##     data = datsc)
##
## Deviance Residuals:
##     Min       1Q   Median       3Q      Max
## -5.8430  -1.7591   0.0269   2.1005   5.5162
##
## Coefficients:
##                        Estimate Std. Error z value Pr(>|z|)
## (Intercept)             1.73270    0.13249  13.078  < 2e-16 ***
## areaCROZET             -0.20640    0.16761  -1.231   0.2181
## areaFALKLANDS          -1.33101    0.10632 -12.519  < 2e-16 ***
## areaHIMI               -3.80393    0.19544 -19.463  < 2e-16 ***
## areaKERGUELEN          -1.26985    0.19167  -6.625 3.47e-11 ***
## areaPEMI               -3.07064    0.18728 -16.396  < 2e-16 ***
## areaSOUTH GEORGIA      -0.37433    0.21072  -1.776   0.0757 .
## mean_depth             -0.48729    0.04139 -11.774  < 2e-16 ***
## mobility                0.41008    0.04647   8.825  < 2e-16 ***
## prop_day_trotline_cach  0.24136    0.03793   6.363 1.98e-10 ***
## prop_day_winter        -0.20669    0.04872  -4.242 2.22e-05 ***
## ---
## Signif. codes:  0 '***' 0.001 '**' 0.01 '*' 0.05 '.' 0.1 ' ' 1
##
## (Dispersion parameter for binomial family taken to be 1)
##
##     Null deviance: 3878.89  on 72  degrees of freedom
## Residual deviance:  538.48  on 62  degrees of freedom
## AIC: 926.45
##
## Number of Fisher Scoring iterations: 5
```

Calculate pseudo r squared

```
pseudo_r2(glm_6.0)
```

```
## [1] 0.8611754
```

Six terms models

Model with density of vessels

```
glm_6.1 <- glm(cbind(count,count_total - count)~ area+ mean_depth+mobility+pr
op_day_trotline_cach+prop_day_winter+density, family = binomial(link = logit)
, data = datsc)
summary(glm_6.1)
```

```
##
## Call:
## glm(formula = cbind(count, count_total - count) ~ area + mean_depth +
##     mobility + prop_day_trotline_cach + prop_day_winter + density,
##     family = binomial(link = logit), data = datsc)
##
## Deviance Residuals:
##     Min       1Q   Median       3Q      Max
## -5.7321  -1.9529  -0.2182   2.0904   5.2293
##
## Coefficients:
##                         Estimate Std. Error z value Pr(>|z|)
## (Intercept)              1.76263    0.13308  13.245  < 2e-16 ***
## areaCROZET              -0.25337    0.16806  -1.508 0.131643
## areaFALKLANDS           -1.21363    0.11123 -10.911  < 2e-16 ***
## areaHIMI                -3.72676    0.19645 -18.970  < 2e-16 ***
## areaKERGUELEN           -1.13848    0.19592  -5.811 6.21e-09 ***
## areaPEMI                -3.13235    0.18863 -16.606  < 2e-16 ***
## areaSOUTH GEORGIA       -0.67317    0.22686  -2.967 0.003004 **
## mean_depth              -0.45864    0.04219 -10.870  < 2e-16 ***
## mobility                 0.40566    0.04676   8.676  < 2e-16 ***
## prop_day_trotline_cach  0.21467    0.03867   5.552 2.82e-08 ***
## prop_day_winter         -0.20329    0.04868  -4.177 2.96e-05 ***
## density                  0.18122    0.05196   3.488 0.000487 ***
## ---
## Signif. codes:  0 '***' 0.001 '**' 0.01 '*' 0.05 '.' 0.1 ' ' 1
##
## (Dispersion parameter for binomial family taken to be 1)
##
##     Null deviance: 3878.89  on 72  degrees of freedom
## Residual deviance:  526.15  on 61  degrees of freedom
## AIC: 916.12
##
## Number of Fisher Scoring iterations: 5
```

Calculate pseudo r squared

```
pseudo_r2(glm_6.1)

## [1] 0.8643543
```

Model with total area size

```r
glm_6.2 <- glm(cbind(count,count_total - count)~ area+ mean_depth+mobility+pr
op_day_trotline_cach+prop_day_winter+total_area, family = binomial(link = log
it), data = datsc)
summary(glm_6.2)
```

```
##
## Call:
## glm(formula = cbind(count, count_total - count) ~ area + mean_depth +
##     mobility + prop_day_trotline_cach + prop_day_winter + total_area,
##     family = binomial(link = logit), data = datsc)
##
## Deviance Residuals:
##     Min       1Q   Median       3Q      Max
## -5.8840  -1.7430   0.0782   1.9044   5.3477
##
## Coefficients:
##                         Estimate Std. Error z value Pr(>|z|)
## (Intercept)              1.95495    0.14733  13.269  < 2e-16 ***
## areaCROZET              -0.53558    0.19207  -2.788 0.005295 **
## areaFALKLANDS           -1.50171    0.11688 -12.849  < 2e-16 ***
## areaHIMI                -4.01270    0.20429 -19.642  < 2e-16 ***
## areaKERGUELEN           -1.23792    0.19303  -6.413 1.42e-10 ***
## areaPEMI                -3.35072    0.20372 -16.448  < 2e-16 ***
## areaSOUTH GEORGIA       -0.85778    0.25097  -3.418 0.000631 ***
## mean_depth              -0.49066    0.04151 -11.821  < 2e-16 ***
## mobility                 0.59733    0.07058   8.463  < 2e-16 ***
## prop_day_trotline_cach  0.19223    0.04042   4.756 1.98e-06 ***
## prop_day_winter         -0.19083    0.04910  -3.886 0.000102 ***
## total_area              -0.29110    0.08119  -3.586 0.000336 ***
## ---
## Signif. codes:  0 '***' 0.001 '**' 0.01 '*' 0.05 '.' 0.1 ' ' 1
##
## (Dispersion parameter for binomial family taken to be 1)
##
##     Null deviance: 3878.89  on 72  degrees of freedom
## Residual deviance:  525.47  on 61  degrees of freedom
## AIC: 915.43
##
## Number of Fisher Scoring iterations: 5
```

Calculate pseudo r squared

```r
pseudo_r2(glm_6.2)
```

```
## [1] 0.8645308
```

AIC summary

```r
model.sel(list(glm_6.0,glm_6.1,glm_6.2))
```

```
## Model selection table
##   (Int) are men_dpt    mbl prp_day_trt_cch prp_day_wnt    dns ttl_are df
## 3 1.955   + -0.4907 0.5973          0.1922     -0.1908        -0.2911 12
## 2 1.763   + -0.4586 0.4057          0.2147     -0.2033 0.1812         12
## 1 1.733   + -0.4873 0.4101          0.2414     -0.2067                11
##     logLik  AICc delta weight
## 3 -445.716 920.6  0.00  0.583
## 2 -446.058 921.3  0.68  0.414
## 1 -452.223 930.8 10.14  0.004
## Models ranked by AICc(x)
```

Model with the lowest AIC

```
glm_7.0 <- glm(cbind(count,count_total - count)~ area+ mean_depth+mobility+pr
op_day_trotline_cach+prop_day_winter+total_area, family = binomial(link = log
it), data = datsc)
summary(glm_7.0)

##
## Call:
## glm(formula = cbind(count, count_total - count) ~ area + mean_depth +
##     mobility + prop_day_trotline_cach + prop_day_winter + total_area,
##     family = binomial(link = logit), data = datsc)
##
## Deviance Residuals:
##     Min       1Q   Median       3Q      Max
## -5.8840  -1.7430   0.0782   1.9044   5.3477
##
## Coefficients:
##                         Estimate Std. Error z value Pr(>|z|)
## (Intercept)              1.95495    0.14733  13.269  < 2e-16 ***
## areaCROZET              -0.53558    0.19207  -2.788 0.005295 **
## areaFALKLANDS           -1.50171    0.11688 -12.849  < 2e-16 ***
## areaHIMI                -4.01270    0.20429 -19.642  < 2e-16 ***
## areaKERGUELEN           -1.23792    0.19303  -6.413 1.42e-10 ***
## areaPEMI                -3.35072    0.20372 -16.448  < 2e-16 ***
## areaSOUTH GEORGIA       -0.85778    0.25097  -3.418 0.000631 ***
## mean_depth              -0.49066    0.04151 -11.821  < 2e-16 ***
## mobility                 0.59733    0.07058   8.463  < 2e-16 ***
## prop_day_trotline_cach   0.19223    0.04042   4.756 1.98e-06 ***
## prop_day_winter         -0.19083    0.04910  -3.886 0.000102 ***
## total_area              -0.29110    0.08119  -3.586 0.000336 ***
## ---
## Signif. codes:  0 '***' 0.001 '**' 0.01 '*' 0.05 '.' 0.1 ' ' 1
##
## (Dispersion parameter for binomial family taken to be 1)
##
##     Null deviance: 3878.89  on 72  degrees of freedom
## Residual deviance:  525.47  on 61  degrees of freedom
## AIC: 915.43
```

```
##
## Number of Fisher Scoring iterations: 5
```

Calculate pseudo r squared

```
pseudo_r2(glm_7.0)

## [1] 0.8645308
```

Seven terms models

Model with density of vessels

```
glm_7.1 <- glm(cbind(count,count_total - count)~ area+ mean_depth+mobility+pr
op_day_trotline_cach+prop_day_winter+total_area+density, family = binomial(li
nk = logit), data = datsc)
summary(glm_7.1)

##
## Call:
## glm(formula = cbind(count, count_total - count) ~ area + mean_depth +
##     mobility + prop_day_trotline_cach + prop_day_winter + total_area +
##     density, family = binomial(link = logit), data = datsc)
##
## Deviance Residuals:
##    Min      1Q  Median      3Q     Max
## -5.957  -1.793   0.117   1.925   5.039
##
## Coefficients:
##                        Estimate Std. Error z value Pr(>|z|)
## (Intercept)             1.90748    0.14939  12.769  < 2e-16 ***
## areaCROZET             -0.46614    0.19513  -2.389 0.016902 *
## areaFALKLANDS          -1.37260    0.13325 -10.301  < 2e-16 ***
## areaHIMI               -3.89944    0.21197 -18.396  < 2e-16 ***
## areaKERGUELEN          -1.16437    0.19656  -5.924 3.15e-09 ***
## areaPEMI               -3.30528    0.20518 -16.109  < 2e-16 ***
## areaSOUTH GEORGIA      -0.90619    0.25196  -3.597 0.000322 ***
## mean_depth             -0.47121    0.04262 -11.057  < 2e-16 ***
## mobility                0.53752    0.07685   6.995 2.66e-12 ***
## prop_day_trotline_cach  0.18981    0.04041   4.697 2.64e-06 ***
## prop_day_winter        -0.19387    0.04901  -3.956 7.63e-05 ***
## total_area             -0.20141    0.09274  -2.172 0.029873 *
## density                 0.11908    0.05931   2.008 0.044678 *
## ---
## Signif. codes:  0 '***' 0.001 '**' 0.01 '*' 0.05 '.' 0.1 ' ' 1
##
## (Dispersion parameter for binomial family taken to be 1)
##
##     Null deviance: 3878.89  on 72  degrees of freedom
## Residual deviance:  521.42  on 60  degrees of freedom
## AIC: 913.38
```

```
##
## Number of Fisher Scoring iterations: 5
```

Calculate pseudo r squared

```
pseudo_r2(glm_7.1)

## [1] 0.8655742
```

AIC summary

```
model.sel(list(glm_7.0,glm_7.1))

## Model selection table
##   (Int) are men_dpt    mbl prp_day_trt_cch prp_day_wnt ttl_are    dns df
## 2 1.907   + -0.4712 0.5375          0.1898     -0.1939 -0.2014 0.1191 13
## 1 1.955   + -0.4907 0.5973          0.1922     -0.1908 -0.2911        12
##     logLik  AICc delta weight
## 2 -443.692 919.6  0.00  0.632
## 1 -445.716 920.6  1.08  0.368
## Models ranked by AICc(x)
```

FINAL AIC SELECTION

```
model.sel(list(glm_null, glm_1.1,glm_1.2,glm_1.3,glm_1.4,glm_1.5,glm_1.6,glm_
1.7,glm_2.0,glm_2.1,glm_2.2,glm_2.3,glm_2.4,glm_2.5,glm_2.6,glm_3.0,glm_3.1,g
lm_3.2,glm_3.3,glm_3.4,glm_3.5,glm_4.0,glm_4.1,glm_4.2,glm_4.3,glm_4.4,glm_5.
0,glm_5.1,glm_5.2,glm_5.3,glm_6.0,glm_6.1,glm_6.2,
               glm_7.0,glm_7.1))

## Model selection table
##     (Int)  ttl_are men_dpt    mbl prp_day_trt_cch prp_day_wnt    dns are
## 35 1.9070 -0.20140 -0.4712 0.5375        0.189800     -0.1939 0.1191   +
## 33 1.9550 -0.29110 -0.4907 0.5973        0.192200     -0.1908          +
## 34 1.9550 -0.29110 -0.4907 0.5973        0.192200     -0.1908          +
## 32 1.7630          -0.4586 0.4057        0.214700     -0.2033 0.1812   +
## 28 1.7330          -0.4873 0.4101        0.241400     -0.2067          +
## 31 1.7330          -0.4873 0.4101        0.241400     -0.2067          +
## 30 2.1600 -0.32080 -0.4852 0.6009        0.166700                     +
## 29 1.9640          -0.4525 0.3892        0.191600             0.1852   +
## 25 1.9390          -0.4822 0.3937        0.218400                     +
## 27 1.9390          -0.4822 0.3937        0.218400                     +
## 26 2.4850 -0.43450 -0.4758 0.6859                                     +
## 24 2.2700          -0.4300 0.4018                             0.2367   +
## 23 2.1580          -0.4671 0.4231                     -0.1616          +
## 21 2.2900          -0.4649 0.4089                                     +
## 22 2.2900          -0.4649 0.4089                                     +
## 20 1.4130          -0.4023               0.236300                     +
## 18 1.7500          -0.3394                                    0.2524   +
## 19 1.6540          -0.3776                            -0.1218          +
## 17 1.8180  0.12550 -0.3923                                            +
```

```
## 11 1.7700          -0.3787                                                    +
## 16 1.7700          -0.3787                                                    +
## 15 1.1400                                                            0.3308   +
## 12 1.3100                  0.2821                                            +
## 13 0.7278                                 0.199900                           +
## 14 0.9504                                              -0.1255               +
## 8  1.0670                                                                    +
## 9  1.0670                                                                    +
## 10 1.0750  0.04977                                                           +
## 7  0.7904                                                            0.4004
## 6  0.6318                                              -0.3277
## 3  0.6954          -0.2530
## 4  0.6600                  0.2477
## 2  0.6231  0.18760
## 1  0.6657
## 5  0.6659                                -0.006707
##    df    logLik   AICc   delta weight
## 35 13  -443.692  919.6    0.00  0.386
## 33 12  -445.716  920.6    1.08  0.225
## 34 12  -445.716  920.6    1.08  0.225
## 32 12  -446.058  921.3    1.76  0.160
## 28 11  -452.223  930.8   11.22  0.001
## 31 11  -452.223  930.8   11.22  0.001
## 30 11  -453.234  932.8   13.24  0.001
## 29 11  -454.764  935.9   16.30  0.000
## 25 10  -461.199  945.9   26.39  0.000
## 27 10  -461.199  945.9   26.39  0.000
## 26 10  -461.989  947.5   27.97  0.000
## 24 10  -467.379  958.3   38.75  0.000
## 23 10  -472.625  968.8   49.25  0.000
## 21  9  -478.246  977.3   57.80  0.000
## 22  9  -478.246  977.3   57.80  0.000
## 20  9  -498.044 1016.9   97.39  0.000
## 18  9  -505.249 1031.4  111.80  0.000
## 19  9  -514.945 1050.7  131.19  0.000
## 17  9  -515.332 1051.5  131.97  0.000
## 11  8  -518.173 1054.6  135.04  0.000
## 16  8  -518.173 1054.6  135.04  0.000
## 15  8  -540.191 1098.6  179.08  0.000
## 12  8  -542.229 1102.7  183.15  0.000
## 13  8  -548.698 1115.6  196.09  0.000
## 14  8  -559.948 1138.1  218.59  0.000
## 8   7  -563.388 1142.5  222.95  0.000
## 9   7  -563.388 1142.5  222.95  0.000
## 10  8  -562.918 1144.1  224.53  0.000
## 7   2 -1967.329 3938.8 3019.28  0.000
## 6   2 -1991.620 3987.4 3067.86  0.000
## 3   2 -2010.375 4024.9 3105.37  0.000
## 4   2 -2023.776 4051.7 3132.17  0.000
## 2   2 -2063.607 4131.4 3211.83  0.000
```

```
## 1   1 -2122.423 4246.9 3327.35  0.000
## 5   2 -2122.350 4248.9 3329.32  0.000
## Models ranked by AICc(x)
```

FINAL MODEL without interactions

```
glm_7.1 <- glm(cbind(count,count_total - count)~ area+ mean_depth+mobility+pr
op_day_trotline_cach+prop_day_winter+total_area+density, family = binomial(li
nk = logit), data = datsc)
summary(glm_7.1)
```

```
##
## Call:
## glm(formula = cbind(count, count_total - count) ~ area + mean_depth +
##     mobility + prop_day_trotline_cach + prop_day_winter + total_area +
##     density, family = binomial(link = logit), data = datsc)
##
## Deviance Residuals:
##    Min      1Q  Median      3Q     Max
## -5.957  -1.793   0.117   1.925   5.039
##
## Coefficients:
##                          Estimate Std. Error z value Pr(>|z|)
## (Intercept)               1.90748    0.14939  12.769  < 2e-16 ***
## areaCROZET               -0.46614    0.19513  -2.389 0.016902 *
## areaFALKLANDS            -1.37260    0.13325 -10.301  < 2e-16 ***
## areaHIMI                 -3.89944    0.21197 -18.396  < 2e-16 ***
## areaKERGUELEN            -1.16437    0.19656  -5.924 3.15e-09 ***
## areaPEMI                 -3.30528    0.20518 -16.109  < 2e-16 ***
## areaSOUTH GEORGIA        -0.90619    0.25196  -3.597 0.000322 ***
## mean_depth               -0.47121    0.04262 -11.057  < 2e-16 ***
## mobility                  0.53752    0.07685   6.995 2.66e-12 ***
## prop_day_trotline_cach    0.18981    0.04041   4.697 2.64e-06 ***
## prop_day_winter          -0.19387    0.04901  -3.956 7.63e-05 ***
## total_area               -0.20141    0.09274  -2.172 0.029873 *
## density                   0.11908    0.05931   2.008 0.044678 *
## ---
## Signif. codes:  0 '***' 0.001 '**' 0.01 '*' 0.05 '.' 0.1 ' ' 1
##
## (Dispersion parameter for binomial family taken to be 1)
##
##     Null deviance: 3878.89  on 72  degrees of freedom
## Residual deviance:  521.42  on 60  degrees of freedom
## AIC: 913.38
##
## Number of Fisher Scoring iterations: 5
```

Model validation

plots of residuals versus fitted values for check of linearity and homoscedasticity of variance

```
plot(fitted(glm_7.1), resid(glm_7.1), main="Residual Plot for linearity and h
omoscedasticity of variance",xlab="Fitted value",ylab="Residuals")
```

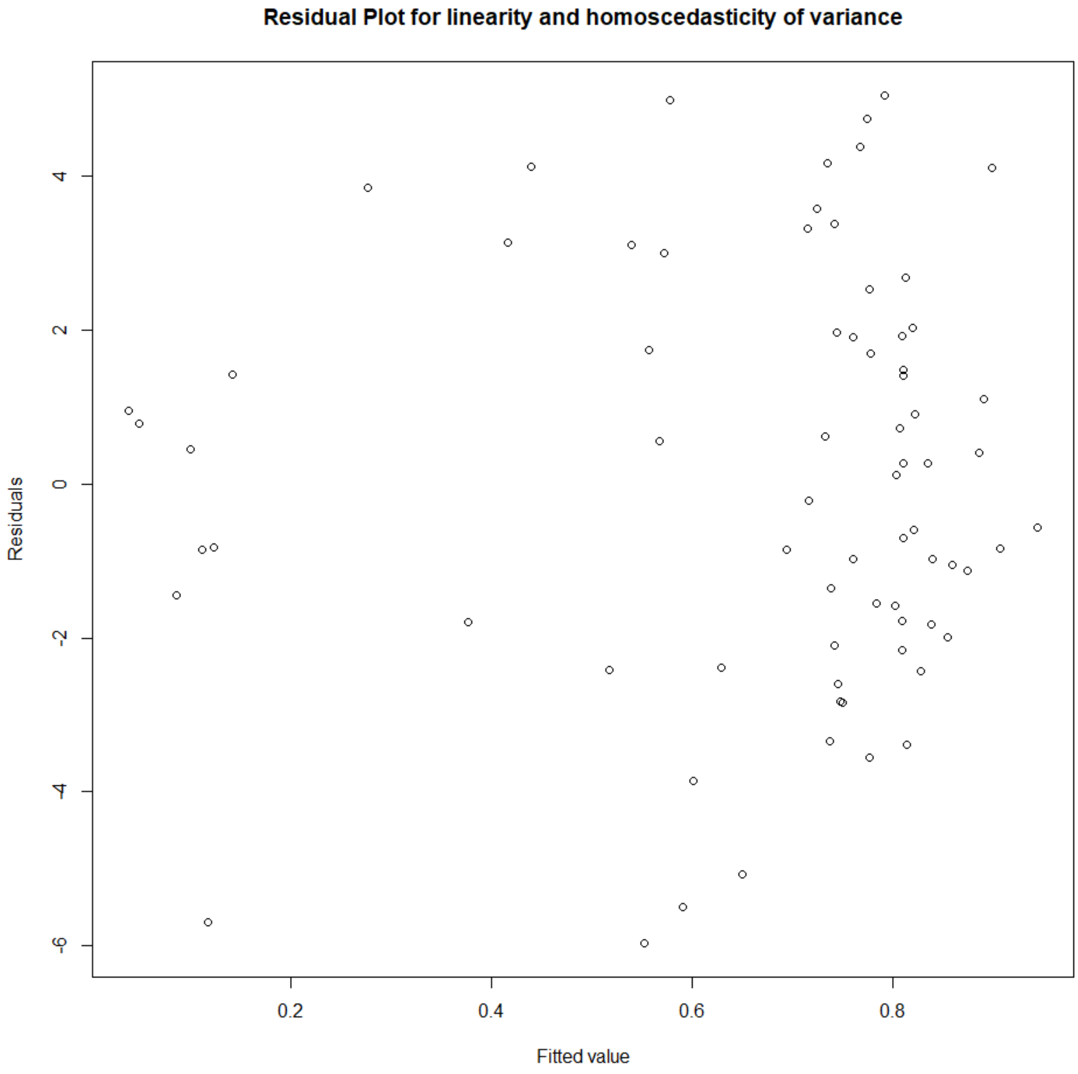

test for normality of the residuals

```
hist(resid(glm_7.1),xlab="Residuals",main="Normality of the Residuals")
```

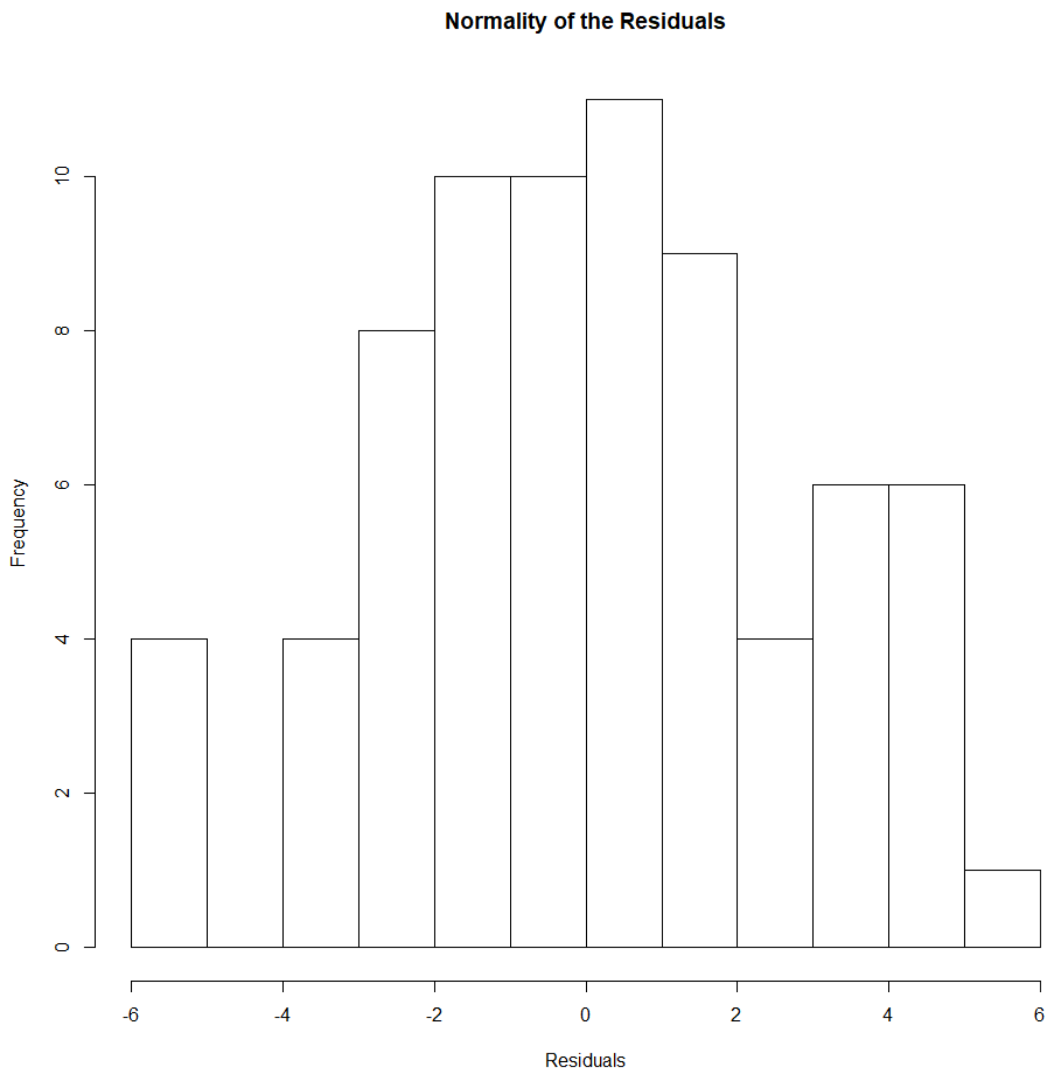

Interaction between Fishery and mobility

A reviewer requested we investigate including an interaction between Fishery and mobility

```
glm_8.0 <- glm(cbind(count,count_total - count)~ area+ mean_depth+mobility+pr
op_day_trotline_cach+
                 prop_day_winter+total_area+density + area:mobility,
               family = binomial(link = logit), data = datsc)
summary(glm_8.0)

##
## Call:
## glm(formula = cbind(count, count_total - count) ~ area + mean_depth +
##     mobility + prop_day_trotline_cach + prop_day_winter + total_area +
##     density + area:mobility, family = binomial(link = logit),
##     data = datsc)
```

```
##
## Deviance Residuals:
##     Min       1Q   Median       3Q      Max
## -5.3701  -1.6825   0.0592   1.5548   6.1950
##
## Coefficients:
##                            Estimate Std. Error z value Pr(>|z|)
## (Intercept)                 4.55992    0.36289  12.565  < 2e-16 ***
## areaCROZET                 -3.28605    0.42474  -7.737 1.02e-14 ***
## areaFALKLANDS              -3.42453    0.38677  -8.854  < 2e-16 ***
## areaHIMI                   -6.52290    0.40024 -16.297  < 2e-16 ***
## areaKERGUELEN              -3.01300    0.34050  -8.849  < 2e-16 ***
## areaPEMI                   -6.34750    1.79097  -3.544 0.000394 ***
## areaSOUTH GEORGIA          -4.04348    0.44406  -9.106  < 2e-16 ***
## mean_depth                 -0.25036    0.04735  -5.288 1.24e-07 ***
## mobility                    3.65355    0.37084   9.852  < 2e-16 ***
## prop_day_trotline_cach      0.08568    0.04404   1.946 0.051708 .
## prop_day_winter            -0.17932    0.05435  -3.299 0.000969 ***
## total_area                 -0.27491    0.10388  -2.646 0.008133 **
## density                     0.31571    0.08421   3.749 0.000178 ***
## areaCROZET:mobility        -3.52705    0.43116  -8.180 2.83e-16 ***
## areaFALKLANDS:mobility     -2.48207    0.38896  -6.381 1.76e-10 ***
## areaHIMI:mobility          -3.01223    0.35934  -8.383  < 2e-16 ***
## areaKERGUELEN:mobility     -3.45532    0.36092  -9.574  < 2e-16 ***
## areaPEMI:mobility          -3.34919    1.55125  -2.159 0.030849 *
## areaSOUTH GEORGIA:mobility -2.88980    0.40538  -7.129 1.01e-12 ***
## ---
## Signif. codes:  0 '***' 0.001 '**' 0.01 '*' 0.05 '.' 0.1 ' ' 1
##
## (Dispersion parameter for binomial family taken to be 1)
##
##     Null deviance: 3878.89  on 72  degrees of freedom
## Residual deviance:  375.23  on 54  degrees of freedom
## AIC: 779.19
##
## Number of Fisher Scoring iterations: 5
```

Calculate pseudo r squared

```
pseudo_r2(glm_8.0)
```

```
## [1] 0.9032642
```

Check the plots of residuals versus fitted values for check of linearity and homoscedasticity of variance

```
plot(fitted(glm_8.0), resid(glm_8.0), main="Residual Plot for linearity and h
omoscedasticity of variance",xlab="Fitted value",ylab="Residuals")
```

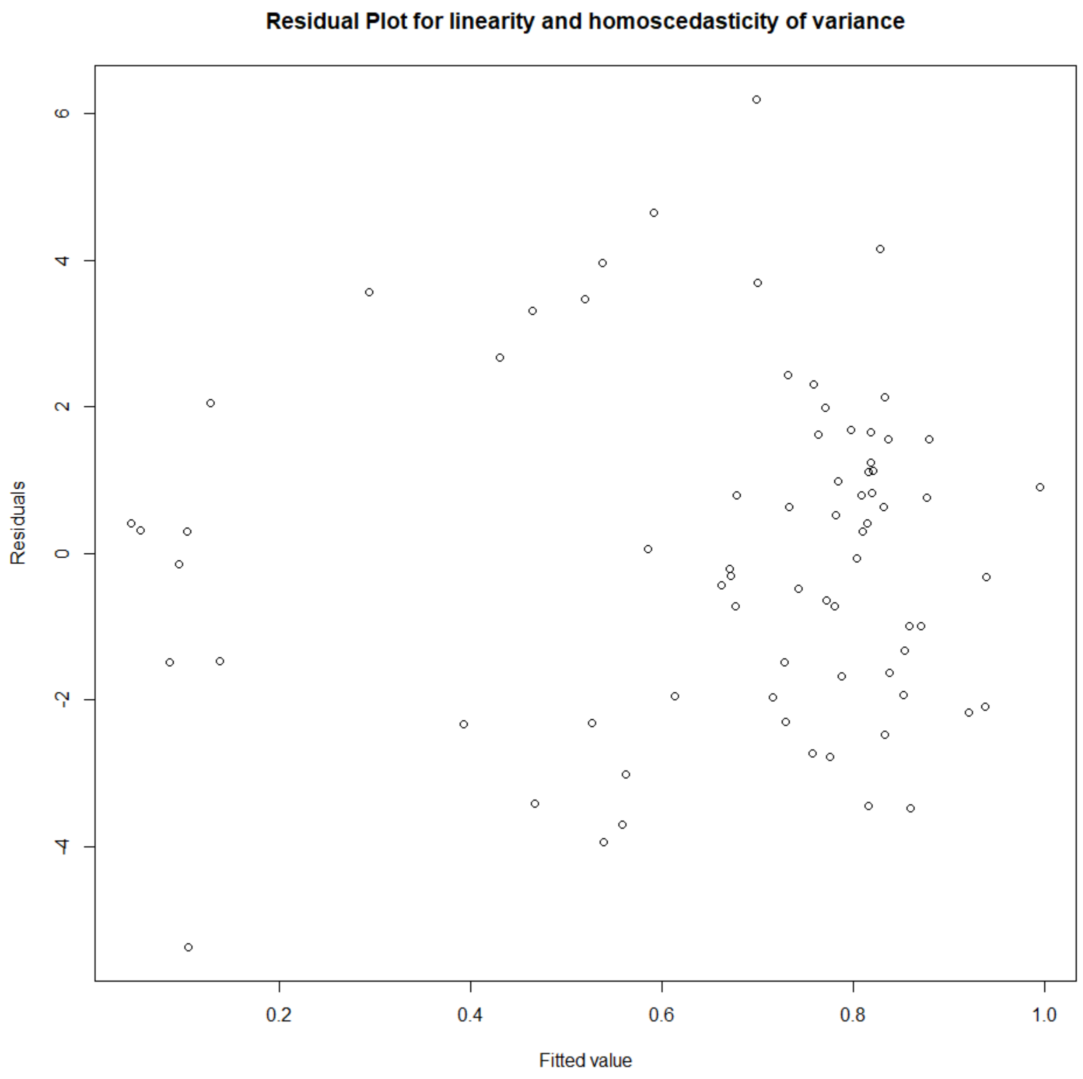

test for normality of the residuals

```
hist(resid(glm_8.0),xlab="Residuals",main="Normality of the Residuals")
```

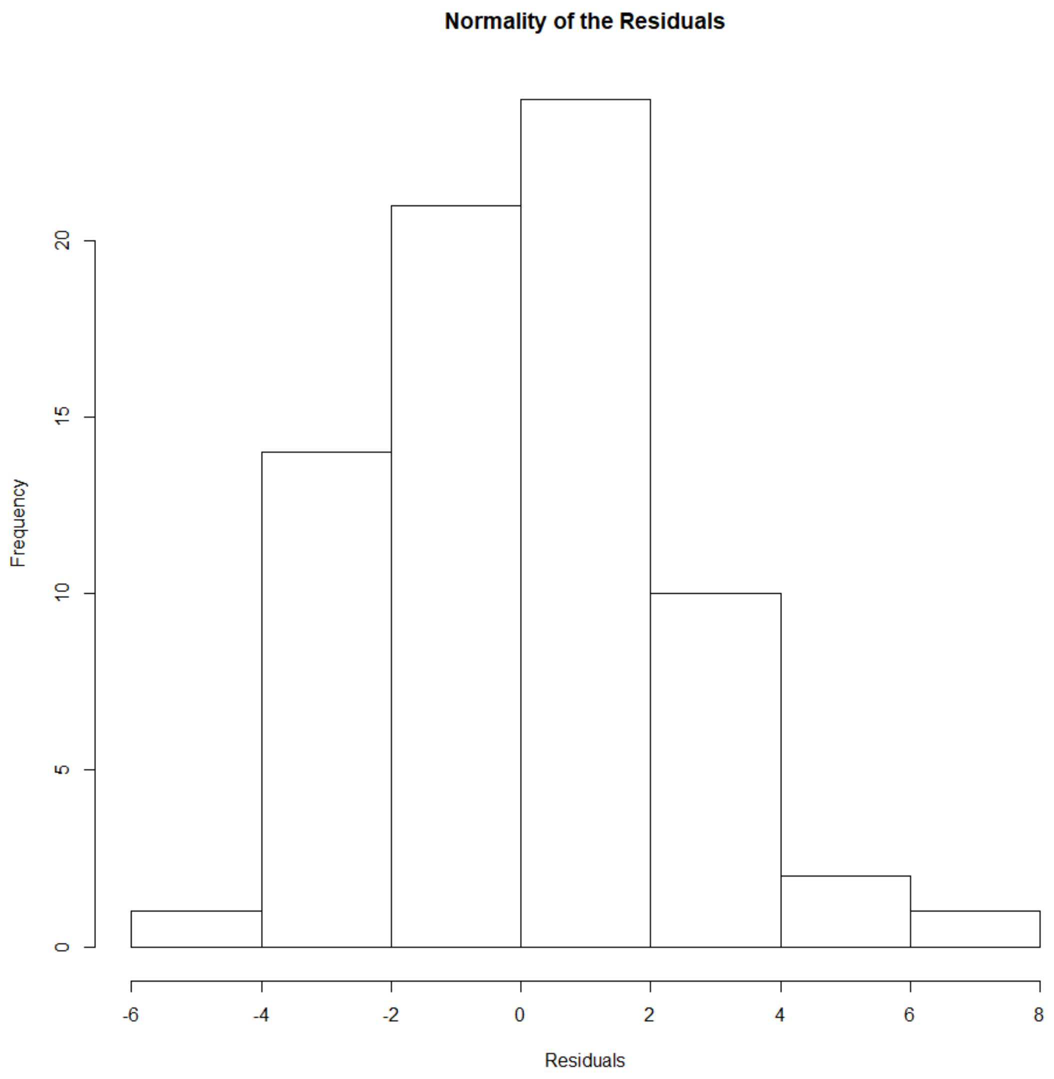

Create the AIC selection table including `glm_8.0`, we also calculate all of the pseudo r squared statistics using the `extra` argument of `model.sel`.

```
(fleet_summary <- model.sel(list(glm_null, glm_1.1,glm_1.2,glm_1.3,glm_1.4,gl
m_1.5,glm_1.6,glm_1.7,
               glm_2.0,glm_2.1,glm_2.2,glm_2.3,glm_2.4,glm_2.5,glm_2.6,
               glm_3.0,glm_3.1,glm_3.2,glm_3.3,glm_3.4,glm_3.5,
               glm_4.0,glm_4.1,glm_4.2,glm_4.3,glm_4.4,
               glm_5.0,glm_5.1,glm_5.2,glm_5.3,
               glm_6.0,glm_6.1,glm_6.2,
               glm_7.0,glm_7.1,
               glm_8.0), rank=AIC, extra=pseudo_r2))

## Model selection table
##     (Int)  ttl_are men_dpt    mbl prp_day_trt_cch prp_day_wnt    dns are
## 36 4.5600 -0.27490 -0.2504 3.6540        0.085680     -0.1793 0.3157   +
## 35 1.9070 -0.20140 -0.4712 0.5375        0.189800     -0.1939 0.1191   +
```

```
## 33 1.9550 -0.29110 -0.4907 0.5973        0.192200     -0.1908          +
## 34 1.9550 -0.29110 -0.4907 0.5973        0.192200     -0.1908          +
## 32 1.7630          -0.4586 0.4057        0.214700     -0.2033 0.1812   +
## 28 1.7330          -0.4873 0.4101        0.241400     -0.2067          +
## 31 1.7330          -0.4873 0.4101        0.241400     -0.2067          +
## 30 2.1600 -0.32080 -0.4852 0.6009        0.166700                      +
## 29 1.9640          -0.4525 0.3892        0.191600             0.1852   +
## 25 1.9390          -0.4822 0.3937        0.218400                      +
## 27 1.9390          -0.4822 0.3937        0.218400                      +
## 26 2.4850 -0.43450 -0.4758 0.6859                                      +
## 24 2.2700          -0.4300 0.4018                             0.2367   +
## 23 2.1580          -0.4671 0.4231                     -0.1616          +
## 21 2.2900          -0.4649 0.4089                                      +
## 22 2.2900          -0.4649 0.4089                                      +
## 20 1.4130          -0.4023               0.236300                      +
## 18 1.7500          -0.3394                                    0.2524   +
## 19 1.6540          -0.3776                            -0.1218          +
## 17 1.8180  0.12550 -0.3923                                             +
## 11 1.7700          -0.3787                                             +
## 16 1.7700          -0.3787                                             +
## 15 1.1400                                                     0.3308   +
## 12 1.3100                  0.2821                                      +
## 13 0.7278                                0.199900                      +
## 14 0.9504                                             -0.1255          +
## 8  1.0670                                                              +
## 9  1.0670                                                              +
## 10 1.0750  0.04977                                                     +
## 7  0.7904                                                     0.4004
## 6  0.6318                                             -0.3277
## 3  0.6954          -0.2530
## 4  0.6600                  0.2477
## 2  0.6231  0.18760
## 1  0.6657
## 5  0.6659                              -0.006707
##    are:mbl pseudo_r2 df    logLik    AIC   delta weight
## 36       + 9.033e-01 19  -370.594  779.2    0.00      1
## 35         8.656e-01 13  -443.692  913.4  134.20      0
## 33         8.645e-01 12  -445.716  915.4  136.24      0
## 34         8.645e-01 12  -445.716  915.4  136.24      0
## 32         8.644e-01 12  -446.058  916.1  136.93      0
## 28         8.612e-01 11  -452.223  926.4  147.26      0
## 31         8.612e-01 11  -452.223  926.4  147.26      0
## 30         8.607e-01 11  -453.234  928.5  149.28      0
## 29         8.599e-01 11  -454.764  931.5  152.34      0
## 25         8.565e-01 10  -461.199  942.4  163.21      0
## 27         8.565e-01 10  -461.199  942.4  163.21      0
## 26         8.561e-01 10  -461.989  944.0  164.79      0
## 24         8.534e-01 10  -467.379  954.8  175.57      0
## 23         8.507e-01 10  -472.625  965.2  186.06      0
## 21         8.478e-01  9  -478.246  974.5  195.30      0
```

```
## 22          8.478e-01  9  -478.246  974.5  195.30      0
## 20          8.375e-01  9  -498.044 1014.1  234.90      0
## 18          8.338e-01  9  -505.249 1028.5  249.31      0
## 19          8.288e-01  9  -514.945 1047.9  268.70      0
## 17          8.286e-01  9  -515.332 1048.7  269.47      0
## 11          8.272e-01  8  -518.173 1052.3  273.16      0
## 16          8.272e-01  8  -518.173 1052.3  273.16      0
## 15          8.158e-01  8  -540.191 1096.4  317.19      0
## 12          8.148e-01  8  -542.229 1100.5  321.27      0
## 13          8.114e-01  8  -548.698 1113.4  334.21      0
## 14          8.056e-01  8  -559.948 1135.9  356.71      0
## 8           8.039e-01  7  -563.388 1140.8  361.59      0
## 9           8.039e-01  7  -563.388 1140.8  361.59      0
## 10          8.041e-01  8  -562.918 1141.8  362.65      0
## 7           7.997e-02  2 -1967.329 3938.7 3159.47      0
## 6           6.744e-02  2 -1991.620 3987.2 3208.05      0
## 3           5.777e-02  2 -2010.375 4024.8 3245.56      0
## 4           5.086e-02  2 -2023.776 4051.6 3272.36      0
## 2           3.033e-02  2 -2063.607 4131.2 3352.03      0
## 1           0.000e+00  1 -2122.423 4246.8 3467.66      0
## 5           3.797e-05  2 -2122.350 4248.7 3469.51      0
## Models ranked by AIC(x)
```

Save a copy to file

```
write.csv(fleet_summary, "Sperm_whale_fleet_data_AIC_summary.csv")
```

Coefficients of final model

Extract the coefficients of the final model and convert from logit space to natural numbers (i.e. probabilities) and the upper and lower confidence intervals

```
flt_est <- plogis(summary(glm_8.0)$coefficients[,1]+summary(glm_8.0)$coeffici
ents[1,1])
flt_lwr <- plogis((summary(glm_8.0)$coefficients[,1]+summary(glm_8.0)$coeffic
ients[1,1]-1.96*summary(glm_8.0)$coefficients[,2]))
flt_upr <- plogis((summary(glm_8.0)$coefficients[,1]+summary(glm_8.0)$coeffic
ients[1,1]+1.96*summary(glm_8.0)$coefficients[,2]))
flt_z_stat <- summary(glm_8.0)$coefficients[,3]
flt_p_value <- summary(glm_8.0)$coefficients[,4]
```

Bind them together

```
(res_fleet <- cbind(flt_est, flt_lwr, flt_upr, flt_z_stat, flt_p_value))

##                                flt_est     flt_lwr   flt_upr flt_z_stat
## (Intercept)                  0.9998905 0.999777103 0.9999463  12.565483
## areaCROZET                   0.7814054 0.608587797 0.8915208  -7.736565
## areaFALKLANDS                0.7568337 0.593227445 0.8691509  -8.854107
## areaHIMI                     0.1231451 0.060230340 0.2353222 -16.297279
## areaKERGUELEN                0.8244685 0.706725059 0.9015272  -8.848642
```

```
## areaPEMI                   0.1433704 0.004977316 0.8484797  -3.544168
## areaSOUTH GEORGIA          0.6263161 0.412431649 0.8000828  -9.105605
## mean_depth                 0.9867389 0.985468057 0.9878999  -5.287652
## mobility                   0.9997291 0.999439790 0.9998690   9.852207
## prop_day_trotline_cach     0.9904876 0.989638960 0.9912674   1.945556
## prop_day_winter            0.9876369 0.986266280 0.9888723  -3.299375
## total_area                 0.9864137 0.983396670 0.9888886  -2.646497
## density                    0.9924275 0.991080719 0.9935723   3.749012
## areaCROZET:mobility        0.7374723 0.546814423 0.8673738  -8.180471
## areaFALKLANDS:mobility     0.8887316 0.788431501 0.9448101  -6.381374
## areaHIMI:mobility          0.8245805 0.699176812 0.9048236  -8.382588
## areaKERGUELEN:mobility     0.7511226 0.598017771 0.8596040  -9.573730
## areaPEMI:mobility          0.7704295 0.138271769 0.9859529  -2.159019
## areaSOUTH GEORGIA:mobility 0.8415929 0.705906005 0.9216279  -7.128605
##                             flt_p_value
## (Intercept)                3.268736e-36
## areaCROZET                 1.021389e-14
## areaFALKLANDS              8.435662e-19
## areaHIMI                   1.031842e-59
## areaKERGUELEN              8.859096e-19
## areaPEMI                   3.938542e-04
## areaSOUTH GEORGIA          8.578691e-20
## mean_depth                 1.238963e-07
## mobility                   6.705482e-23
## prop_day_trotline_cach     5.170810e-02
## prop_day_winter            9.690022e-04
## total_area                 8.133018e-03
## density                    1.775327e-04
## areaCROZET:mobility        2.827354e-16
## areaFALKLANDS:mobility     1.755062e-10
## areaHIMI:mobility          5.177666e-17
## areaKERGUELEN:mobility     1.031166e-21
## areaPEMI:mobility          3.084867e-02
## areaSOUTH GEORGIA:mobility 1.013915e-12
```

Create the estimates for Chile (which is zero in binomial regression model)

```
(areaChile <- c(plogis(0+summary(glm_8.0)$coefficients[1,1]),
               plogis(0+summary(glm_8.0)$coefficients[1,1]-1.96*summary(glm_8
.0)$coefficients[1,2]),
               plogis(0+summary(glm_8.0)$coefficients[1,1]+1.96*summary(glm_8
.0)$coefficients[1,2]),
               summary(glm_8.0)$coefficients[1,3], summary(glm_8.0)$coefficie
nts[1,4]))
```

```
## [1] 9.896455e-01 9.791362e-01 9.948888e-01 1.256548e+01 3.268736e-36
```

Replace the intercept with Chilean values

```
res_fleet[1,] <- areaChile
```

Save as a csv

```
write.csv(res_fleet, "Sperm_whale_final_model_fleet_est.csv")
```

Session information

Print the session information

```
sessionInfo()

## R version 3.4.1 (2017-06-30)
## Platform: x86_64-w64-mingw32/x64 (64-bit)
## Running under: Windows 10 x64 (build 17134)
##
## Matrix products: default
##
## locale:
## [1] LC_COLLATE=English_Australia.1252  LC_CTYPE=English_Australia.1252
## [3] LC_MONETARY=English_Australia.1252 LC_NUMERIC=C
## [5] LC_TIME=English_Australia.1252
##
## attached base packages:
## [1] stats     graphics  grDevices utils     datasets  methods   base
##
## other attached packages:
##  [1] Rmisc_1.5       plyr_1.8.4      lattice_0.20-35 ggpubr_0.1.6
##  [5] magrittr_1.5    ggplot2_2.2.1   MuMIn_1.15.6    merTools_0.3.0
##  [9] dplyr_0.7.2     arm_1.9-3       MASS_7.3-47     lmerTest_2.0-33
## [13] lme4_1.1-15     Matrix_1.2-10
##
## loaded via a namespace (and not attached):
##  [1] Rcpp_0.12.12        mvtnorm_1.0-6       tidyr_0.7.0
##  [4] assertthat_0.2.0    rprojroot_1.2       digest_0.6.12
##  [7] psych_1.7.5         mime_0.5            R6_2.2.2
## [10] backports_1.1.0     acepack_1.4.1       stats4_3.4.1
## [13] evaluate_0.10.1     coda_0.19-1         rlang_0.1.2
## [16] lazyeval_0.2.0      minqa_1.2.4         data.table_1.10.4
## [19] nloptr_1.0.4        rpart_4.1-11        DT_0.2
## [22] checkmate_1.8.3     rmarkdown_1.6       labeling_0.3
## [25] splines_3.4.1       stringr_1.2.0       foreign_0.8-69
## [28] htmlwidgets_0.9     munsell_0.4.3       shiny_1.0.5
## [31] broom_0.4.2         compiler_3.4.1      httpuv_1.3.5
## [34] pkgconfig_2.0.1     base64enc_0.1-3     mnormt_1.5-5
## [37] htmltools_0.3.6     nnet_7.3-12         tibble_1.3.4
## [40] gridExtra_2.2.1     htmlTable_1.9       Hmisc_4.0-3
## [43] grid_3.4.1          xtable_1.8-2        nlme_3.1-131
## [46] gtable_0.2.0        scales_0.5.0        stringi_1.1.5
## [49] reshape2_1.4.3      bindrcpp_0.2        latticeExtra_0.6-28
## [52] cowplot_0.8.0       Formula_1.2-2       blme_1.0-4
## [55] RColorBrewer_1.1-2  tools_3.4.1         glue_1.1.1
## [58] purrr_0.2.3         abind_1.4-5         parallel_3.4.1
```

```
## [61] survival_2.41-3     yaml_2.1.14         colorspace_1.3-2
## [64] cluster_2.0.6       knitr_1.17          bindr_0.1
```
