# Supplementary material for: Commercial fishing patterns influence odontocete whale-longline interactions in the Southern Ocean
Source: Sci Rep. 2019 Feb 13;9:1904. doi: 10.1038/s41598-018-36389-x (PMC6374415; doi:10.1038/s41598-018-36389-x)
Supplement: Supplementary file 5 — Data S2 [file 41598_2018_36389_MOESM5_ESM.pdf]

Commercial fishing patterns influence odontocete whale-longline interactions in the Southern Ocean

P. Tixier, P. Burch, G. Richard, K. Olsson, D. Welsford, M.-A. Lea, M. A. Hindell, C. Guinet, A. Janc, N. Gasco, G. Duhamel, M. C. Villanueva, L. Suberg, R. Arangio, M. Söffker, J. P. Y. Arnould

Data S2

Analysis of killer whale - fisheries interactions using Generalised Linear Models

August 28th, 2018

Load packages

```
library(lme4)

library(lmerTest)

library(merTools)

library(MuMIn)
library(ggplot2)
library(ggpubr)

library(Rmisc)
```

Function Definitions

Define the pseudo r squared statistic of Swartzman et al (1992)

```
pseudo_r2 <- function(fit, null_fit=NULL){
  if(is.null(null_fit)){
    null_dev <- fit$null.deviance
  }else{
    null_dev <- null_fit$deviance
  }
  ## calcualte pseudo r2
  pseudo_r2 <- 1- (fit$deviance/null_dev)
  ## return the pseudor2
  pseudo_r2
}
```

A Models on *Pr(days)* at the vessel level

A.1. Data preparation

Import the data

```
setwd("C:/Users/LES_research211/Documents/003_ANALYSES/009_SOUTHERN_OCEAN_PAP
ER")
data=read.csv("final_dataset_modeling_day_interaction.csv",h=T,sep=",")
str(data)

## 'data.frame':    5088 obs. of  24 variables:
##  $ area                : Factor w/ 7 levels "CHILE","CROZET",..: 1 1 1 1
1 1 1 1 1 1 ...
##  $ year                : int  2006 2006 2006 2006 2006 2006 2006 2006 20
06 2006 ...
```

```
##  $ vessel                 : Factor w/ 67 levels "A","ABA","AN",..: 55 19 18
32 32 18 19 55 19 55 ...
##  $ depredation            : int  0 0 0 0 1 1 1 1 0 0 ...
##  $ count                  : int  35 33 4 0 3 59 15 10 5 2 ...
##  $ Pr_inter               : num  0.7778 0.6875 0.0635 0 1 ...
##  $ species                : Factor w/ 2 levels "killer whales",..: 1 1 1 1
1 1 1 1 2 2 ...
##  $ response               : Factor w/ 3 levels "Pr(area)","Pr(days)",..: 1
1 1 1 1 1 1 1 1 1 ...
##  $ area_yr_vessel         : Factor w/ 424 levels "CHILE 2006 GPI",..: 4 2 1
3 3 1 2 4 2 4 ...
##  $ area_size_vessel       : int  45 48 63 3 3 63 48 45 48 45 ...
##  $ count_total            : int  40 59 64 3 3 64 59 40 59 40 ...
##  $ mean_nb_vessels_vessel: num  2.61 2.41 2.36 3.33 3.33 ...
##  $ mean_lat_vessel        : num  -55.8 -55.6 -55.6 -56.3 -56.3 ...
##  $ mean_depth_vessel      : num  1536 1568 1480 1508 1508 ...
##  $ mean_effort_day        : num  8859 6845 7842 12800 12800 ...
##  $ prop_day_winter        : num  0 0 0 0 0 0 0 0 0 0 ...
##  $ prop_day_spring        : num  0.975 0.847 0.875 1 1 ...
##  $ prop_day_summer        : num  0.025 0.153 0.125 0 0 ...
##  $ prop_day_autumn        : num  0 0 0 0 0 0 0 0 0 0 ...
##  $ prop_day_spanish       : num  0 0 0 0 0 0 0 0 0 0 ...
##  $ prop_day_trotline      : num  0 0 0 0 0 0 0 0 0 0 ...
##  $ prop_day_trotline_cach: num  0.475 2.085 0.188 0 0 ...
##  $ prop_day_autoline      : num  1.5 0 1.77 1 1 ...
##  $ prop_day_japanese      : num  0 0 0 0 0 0 0 0 0 0 ...
```

Prepare the data for modelling

```
data$count_total=as.numeric(as.character(data$count_total))
data$area_size_vessel=as.numeric(as.character(data$area_size_vessel))
data$prop_day_trotline_cach=as.numeric(as.character(data$prop_day_trotline_ca
ch))
data <-subset(data,!is.na(mean_lat_vessel))
data <-subset(data,!is.na(mean_depth_vessel))
data$mean_lat_vessel=-data$mean_lat_vessel
data$mobility =data$area_size_vessel/data$count_total

setwd("C:/Users/LES_research211/Documents/003_ANALYSES/009_SOUTHERN_OCEAN_PAP
ER")
data_fleet=read.csv("summary_proportions_predictors_fleet_pemi_a.csv",h=T,sep
=",")
str(data_fleet)
```

```
## 'data.frame':    912 obs. of  22 variables:
##  $ area                   : Factor w/ 7 levels "CHILE","CROZET",..: 6 6 6 5
5 5 5 5 5 4 ...
##  $ year                   : int  2017 2017 2017 2013 2013 2013 2016 2016 20
16 2011 ...
##  $ depredation            : int  0 0 0 0 0 0 0 0 0 0 ...
##  $ count                  : int  366 130 133 2557 253 875 2532 248 838 493
```

```
...
##  $ count_total           : int  130 130 130 253 253 253 248 248 248 191 ..
.
##  $ Pr_inter              : num  1 1 1 1 1 1 1 1 1 1 ...
##  $ species               : Factor w/ 2 levels "killer whales",..: 2 2 2 1
1 1 1 1 1 1 ...
##  $ response              : Factor w/ 4 levels "Pr(area)","Pr(days)",..: 3
2 1 3 2 1 3 2 1 3 ...
##  $ area_yr               : Factor w/ 76 levels "CHILE 2006","CHILE 2007",.
.: 62 62 62 56 56 56 59 59 59 40 ...
##  $ total_area            : int  133 133 133 875 875 875 838 838 838 223 ..
.
##  $ length_season         : int  130 130 130 253 253 253 248 248 248 191 ..
.
##  $ mean_nb_vessels       : num  1 1 1 4.38 4.38 ...
##  $ mean_lat              : num  -45.3 -45.3 -45.3 -48.9 -48.9 ...
##  $ mean_depth            : num  1352 1352 1352 1199 1199 ...
##  $ prop_day_winter       : num  0.462 0.462 0.462 0.158 0.158 ...
##  $ prop_day_spring       : num  0 0 0 0.316 0.316 ...
##  $ prop_day_summer       : num  0.0692 0.0692 0.0692 0.2451 0.2451 ...
##  $ prop_day_autumn       : num  0.469 0.469 0.469 0.281 0.281 ...
##  $ prop_day_spanish      : num  0 0 0 0 0 0 0 0 0 0 ...
##  $ prop_day_autoline     : num  0 0 0 1 1 1 1 1 1 1 ...
##  $ prop_day_trotline_cach: num  0.485 0.485 0.485 0 0 ...
##  $ prop_day_trotline     : num  0.515 0.515 0.515 0 0 ...
```

```
data$area_yr = paste(data$area,data$year)
data_fleet$area_yr = paste(data_fleet$area,data_fleet$year)
data$total_area <- data_fleet$total_area[match(data$area_yr,data_fleet$area_y
r)]
data$density =data$mean_nb_vessels_vessel/data$total_area
```

Data selection

```
d1 <- droplevels(data[data$depredation==1 & data$species %in% "killer whales"
& data$response %in% "Pr(days)",
        c("area","year","count","count_total", "Pr_inter", "vessel","mean_dep
th_vessel","mobility","prop_day_trotline_cach", "prop_day_winter","total_area
","density" )])
str(d1)
```

```
## 'data.frame':    410 obs. of  12 variables:
##  $ area                  : Factor w/ 7 levels "CHILE","CROZET",..: 1 1 1 1
1 1 1 1 1 1 ...
##  $ year                  : int  2006 2006 2006 2006 2007 2007 2007 2007 20
08 2008 ...
##  $ count                 : int  3 56 12 13 91 24 28 13 26 62 ...
##  $ count_total           : num  3 64 40 59 91 28 65 51 57 164 ...
##  $ Pr_inter              : num  1 0.875 0.3 0.22 1 ...
##  $ vessel                : Factor w/ 67 levels "A","ABA","AN",..: 32 18 55
19 18 19 55 2 10 19 ...
```

```
##  $ mean_depth_vessel     : num  1508 1480 1536 1568 1507 ...
##  $ mobility              : num  1 0.984 1.125 0.814 0.78 ...
##  $ prop_day_trotline_cach: num  0 0.188 0.475 2.085 2.176 ...
##  $ prop_day_winter       : num  0 0 0 0 0 ...
##  $ total_area            : int  141 141 141 141 173 173 173 173 237 237 ..
.
##  $ density               : num  0.02364 0.01674 0.01849 0.01707 0.00887 ..
.
```

Data exploration

```
hist(d1$mean_depth_vessel)
```

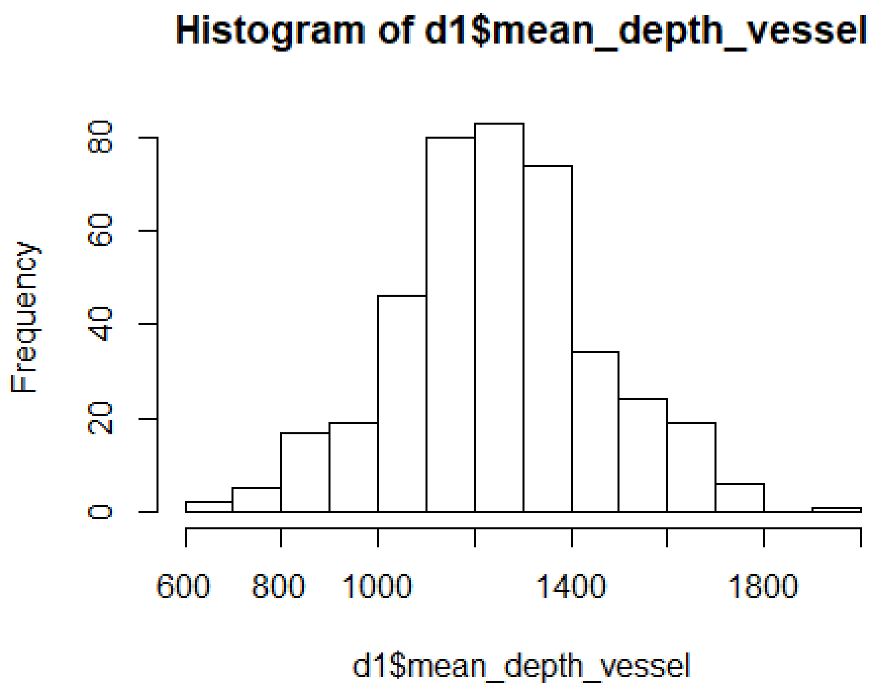


```
hist(d1$total_area)
```

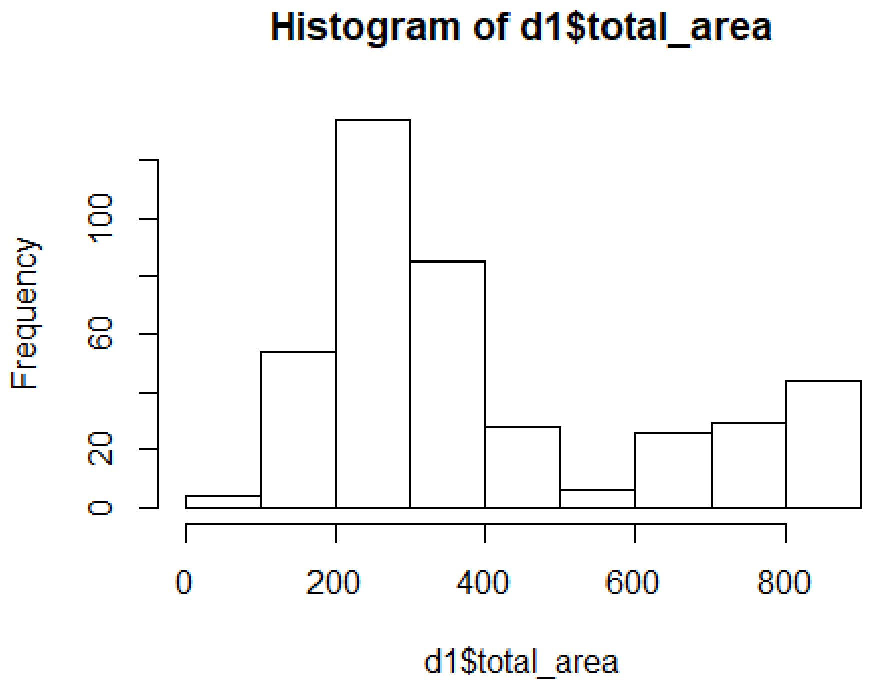


```
hist(d1$mobility)
```

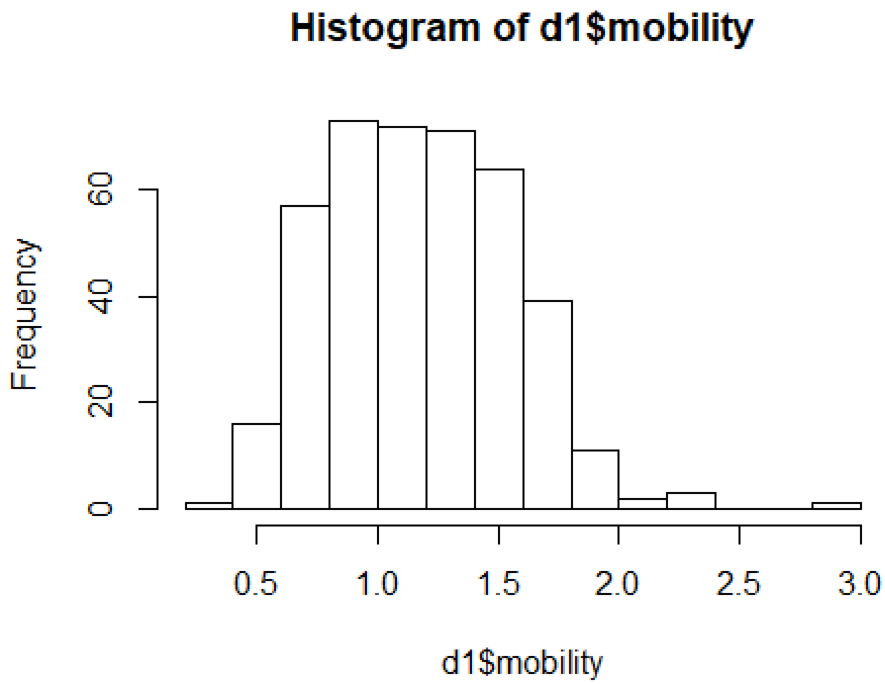

```
# exclude extreme high value
d1 = subset(d1, d1$mobility<=2.5)

hist(d1$density)
```

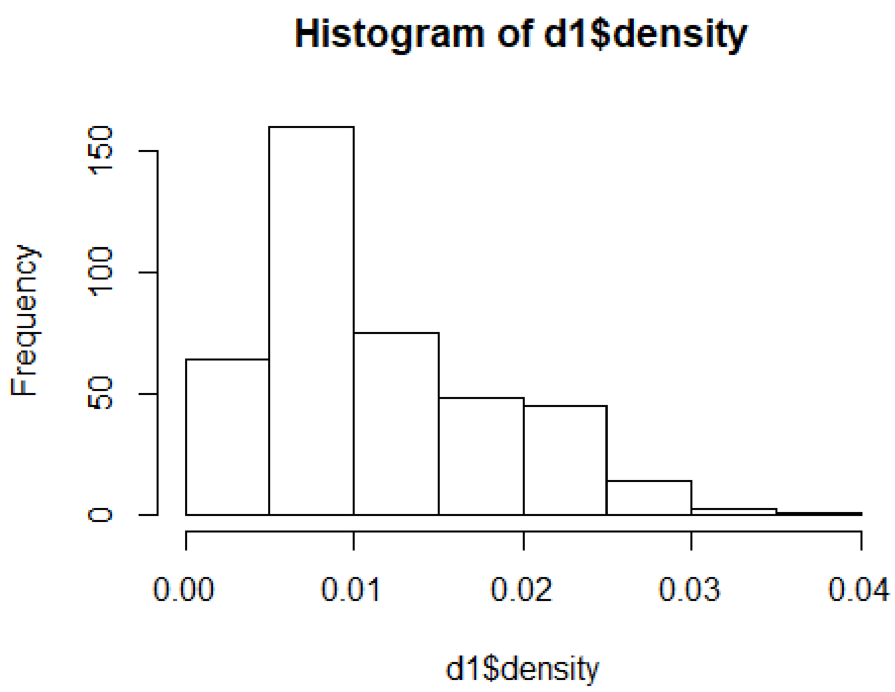


```
# exclude extreme high value
d1 = subset(d1, d1$density<=0.035)
```

Exploratory plots

```
p1 <- ggplot(d1,aes(mean_depth_vessel,Pr_inter,colour=area))+geom_point()+ggt
itle("depth")
p2 <- ggplot(d1,aes(density,Pr_inter,colour=area))+geom_point()+ggtitle("dens
ity")
p3 <- ggplot(d1,aes(prop_day_winter,Pr_inter,colour=area))+geom_point()+ggtit
le("effort in winter")
p4 <- ggplot(d1,aes(total_area,Pr_inter,colour=area))+geom_point()+ggtitle("t
otal area size")
p5 <- ggplot(d1,aes(mobility,Pr_inter,colour=area))+geom_point()+ggtitle("mob
ility")
p6 <- ggplot(d1,aes(count_total,Pr_inter,colour=area))+geom_point()+ggtitle("
nb fishing days")

ggarrange(p1, p2,p3,p4,p5,p6, common.legend=TRUE, legend = "right")
```

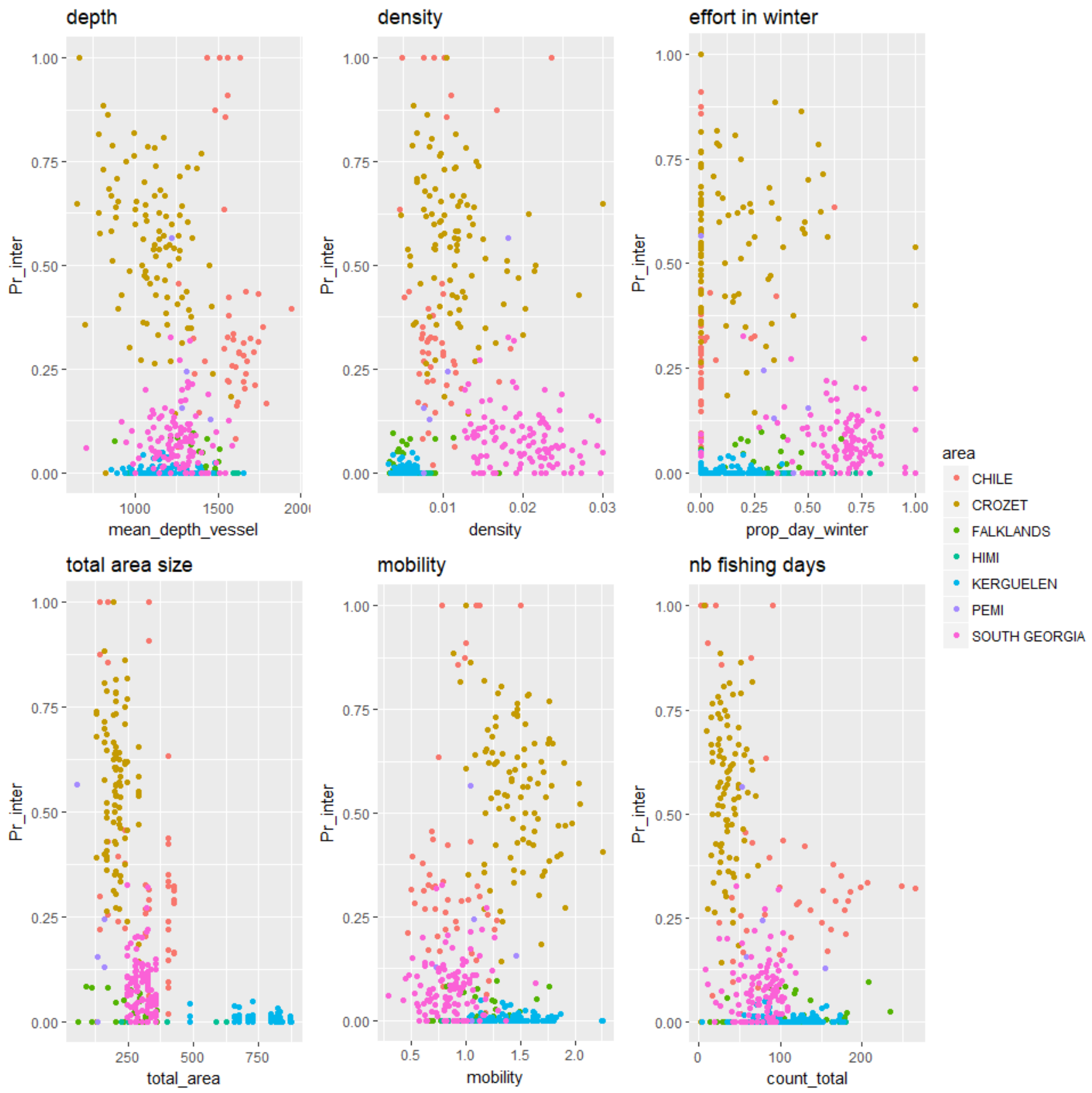

Pairwise plots

```
d1_explo <-droplevels(d1[,
        c(  "mean_depth_vessel","total_area","density", "count_total","mobili
ty","prop_day_winter","prop_day_trotline_cach")])
pairs(d1_explo)
```

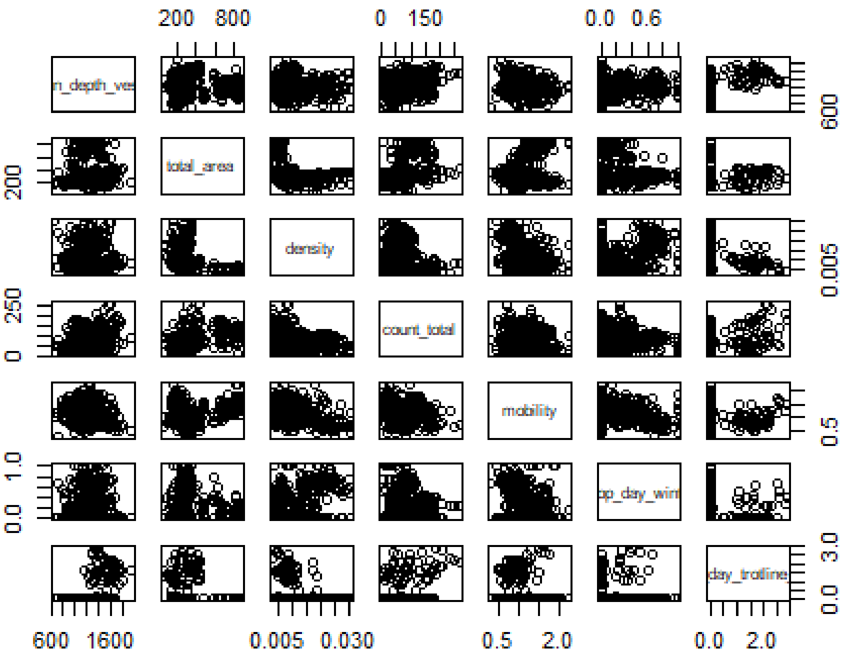

Pearson correlation

Calculate Pearson correlation coefficients for the above data

```
print(cor(d1_explo, method=c("pearson")), digits=4)

##                         mean_depth_vessel total_area  density count_total
## mean_depth_vessel                 1.00000   -0.02004 -0.08112     0.25987
## total_area                       -0.02004    1.00000 -0.47783     0.50976
## density                          -0.08112   -0.47783  1.00000    -0.35101
## count_total                       0.25987    0.50976 -0.35101     1.00000
## mobility                         -0.25118    0.26095 -0.45266    -0.20633
## prop_day_winter                  -0.02605   -0.25759  0.50612     0.01525
## prop_day_trotline_cach            0.48570   -0.17325 -0.17755     0.23666
##                         mobility prop_day_winter prop_day_trotline_cach
## mean_depth_vessel        -0.2512        -0.02605                 0.4857
## total_area                0.2610        -0.25759                -0.1732
## density                  -0.4527         0.50612                -0.1776
## count_total              -0.2063         0.01525                 0.2367
## mobility                  1.0000        -0.39491                -0.2045
## prop_day_winter          -0.3949         1.00000                -0.2176
## prop_day_trotline_cach   -0.2045        -0.21756                 1.0000
```

Re-scale continuous predictors

```
pvars <- c("mean_depth_vessel","prop_day_trotline_cach", "prop_day_winter","t
otal_area","density","mobility" )
```

```
datsc <- d1
datsc[pvars] <- lapply(datsc[pvars],scale)
```

A.2. Model fitting

We fit a glm to the proportion of days with killer whale interaction using a binomial fmaily and a logit link function. The most parcimonious model is selected using a stepwise forward AIC selection

Null model

```
glm_null <- glm(cbind(count,count_total - count)~ 1, family = binomial(link =
logit), data = datsc)
summary(glm_null)

##
## Call:
## glm(formula = cbind(count, count_total - count) ~ 1, family = binomial(lin
k = logit),
##     data = datsc)
##
## Deviance Residuals:
##    Min      1Q  Median      3Q     Max
## -7.037  -4.588  -1.462   3.971  19.323
##
## Coefficients:
##             Estimate Std. Error z value Pr(>|z|)
## (Intercept) -1.91406    0.01635  -117.1   <2e-16 ***
## ---
## Signif. codes:  0 '***' 0.001 '**' 0.01 '*' 0.05 '.' 0.1 ' ' 1
##
## (Dispersion parameter for binomial family taken to be 1)
##
##     Null deviance: 10139  on 407  degrees of freedom
## Residual deviance: 10139  on 407  degrees of freedom
## AIC: 11177
##
## Number of Fisher Scoring iterations: 5
```

Calculate pseudo r squared for the Null model as a check

```
pseudo_r2(glm_null)

## [1] 0
```

Single term models

Model with depth

```
glm_1.1 <- glm(cbind(count,count_total - count)~ mean_depth_vessel, family =
binomial(link = logit), data = datsc)
summary(glm_1.1)
```

```
##
## Call:
## glm(formula = cbind(count, count_total - count) ~ mean_depth_vessel,
##     family = binomial(link = logit), data = datsc)
##
## Deviance Residuals:
##    Min      1Q  Median      3Q     Max
## -7.181  -4.517  -1.427   3.957  18.658
##
## Coefficients:
##                    Estimate Std. Error  z value Pr(>|z|)
## (Intercept)       -1.94681    0.01694 -114.955   <2e-16 ***
## mean_depth_vessel  0.15742    0.01629    9.661   <2e-16 ***
## ---
## Signif. codes:  0 '***' 0.001 '**' 0.01 '*' 0.05 '.' 0.1 ' ' 1
##
## (Dispersion parameter for binomial family taken to be 1)
##
##     Null deviance: 10139  on 407  degrees of freedom
## Residual deviance: 10046  on 406  degrees of freedom
## AIC: 11086
##
## Number of Fisher Scoring iterations: 5
```

Calculate pseudo r squared

```
pseudo_r2(glm_1.1)

## [1] 0.009162282
```

Model with proportion of sets using trotlines-cachalotera

```
glm_1.2 <- glm(cbind(count,count_total - count)~ prop_day_trotline_cach, fami
ly = binomial(link = logit), data = datsc)
summary(glm_1.2)

##
## Call:
## glm(formula = cbind(count, count_total - count) ~ prop_day_trotline_cach,
##     family = binomial(link = logit), data = datsc)
##
## Deviance Residuals:
##     Min       1Q   Median       3Q      Max
## -10.781   -4.262   -1.282    3.748   15.625
##
## Coefficients:
##                         Estimate Std. Error z value Pr(>|z|)
## (Intercept)            -2.01173    0.01749 -115.04   <2e-16 ***
## prop_day_trotline_cach  0.31792    0.01170   27.18   <2e-16 ***
## ---
## Signif. codes:  0 '***' 0.001 '**' 0.01 '*' 0.05 '.' 0.1 ' ' 1
```

```
##
## (Dispersion parameter for binomial family taken to be 1)
##
##     Null deviance: 10138.7  on 407  degrees of freedom
## Residual deviance:  9466.4  on 406  degrees of freedom
## AIC: 10506
##
## Number of Fisher Scoring iterations: 5
```

Calculate pseudo r squared

```
pseudo_r2(glm_1.2)

## [1] 0.0663132
```

Model with proportion of days in winter

```
glm_1.3 <- glm(cbind(count,count_total - count)~ prop_day_winter, family = bi
nomial(link = logit), data = datsc)
summary(glm_1.3)

##
## Call:
## glm(formula = cbind(count, count_total - count) ~ prop_day_winter,
##     family = binomial(link = logit), data = datsc)
##
## Deviance Residuals:
##     Min       1Q   Median       3Q      Max
## -7.4664  -4.5317  -0.7248   3.7373  18.0227
##
## Coefficients:
##                 Estimate Std. Error z value Pr(>|z|)
## (Intercept)     -1.95509    0.01700 -114.99   <2e-16 ***
## prop_day_winter -0.37515    0.01957  -19.17   <2e-16 ***
## ---
## Signif. codes:  0 '***' 0.001 '**' 0.01 '*' 0.05 '.' 0.1 ' ' 1
##
## (Dispersion parameter for binomial family taken to be 1)
##
##     Null deviance: 10138.7  on 407  degrees of freedom
## Residual deviance:  9735.8  on 406  degrees of freedom
## AIC: 10776
##
## Number of Fisher Scoring iterations: 5
```

Calculate pseudo r squared

```
pseudo_r2(glm_1.3)

## [1] 0.03973892
```

Model with total area size

```
glm_1.4 <- glm(cbind(count,count_total - count)~ total_area, family = binomia
l(link = logit), data = datsc)
summary(glm_1.4)
```

```
##
## Call:
## glm(formula = cbind(count, count_total - count) ~ total_area,
##     family = binomial(link = logit), data = datsc)
##
## Deviance Residuals:
##     Min       1Q   Median       3Q      Max
## -11.538   -2.701   -1.084    2.196   14.076
##
## Coefficients:
##             Estimate Std. Error z value Pr(>|z|)
## (Intercept) -2.17886    0.02339  -93.16   <2e-16 ***
## total_area  -1.53132    0.03328  -46.01   <2e-16 ***
## ---
## Signif. codes:  0 '***' 0.001 '**' 0.01 '*' 0.05 '.' 0.1 ' ' 1
##
## (Dispersion parameter for binomial family taken to be 1)
##
##     Null deviance: 10138.7  on 407  degrees of freedom
## Residual deviance:  6033.3  on 406  degrees of freedom
## AIC: 7073.2
##
## Number of Fisher Scoring iterations: 5
```

Calculate pseudo r squared

```
pseudo_r2(glm_1.4)
```

```
## [1] 0.4049282
```

Model with density of vessels

```
glm_1.5 <- glm(cbind(count,count_total - count)~ density, family = binomial(l
ink = logit), data = datsc)
summary(glm_1.5)
```

```
##
## Call:
## glm(formula = cbind(count, count_total - count) ~ density, family = binomi
al(link = logit),
##     data = datsc)
##
## Deviance Residuals:
##    Min      1Q  Median      3Q     Max
## -6.457  -4.560  -1.954   3.850  19.536
##
## Coefficients:
```

```
##              Estimate Std. Error z value Pr(>|z|)
## (Intercept) -1.88585    0.01642 -114.88   <2e-16 ***
## density      0.22477    0.01602   14.03   <2e-16 ***
## ---
## Signif. codes:  0 '***' 0.001 '**' 0.01 '*' 0.05 '.' 0.1 ' ' 1
##
## (Dispersion parameter for binomial family taken to be 1)
##
##     Null deviance: 10138.7  on 407  degrees of freedom
## Residual deviance:  9950.1  on 406  degrees of freedom
## AIC: 10990
##
## Number of Fisher Scoring iterations: 6
```

Calculate pseudo r squared

```
pseudo_r2(glm_1.5)

## [1] 0.01859899
```

Model with the fishery

```
glm_1.6 <- glm(cbind(count,count_total - count)~ area, family = binomial(link
= logit), data = datsc)
summary(glm_1.6)

##
## Call:
## glm(formula = cbind(count, count_total - count) ~ area, family = binomial(
link = logit),
##     data = datsc)
##
## Deviance Residuals:
##     Min       1Q   Median       3Q      Max
## -5.6682  -1.2632  -0.3206   0.8205  14.3466
##
## Coefficients:
##                      Estimate Std. Error z value Pr(>|z|)
## (Intercept)          -0.74120    0.03082 -24.046  < 2e-16 ***
## areaCROZET            0.93836    0.04648  20.190  < 2e-16 ***
## areaFALKLANDS        -2.53931    0.10896 -23.305  < 2e-16 ***
## areaHIMI            -19.76403  339.92907  -0.058    0.954
## areaKERGUELEN        -4.47400    0.13102 -34.147  < 2e-16 ***
## areaPEMI             -0.72514    0.12934  -5.606 2.07e-08 ***
## areaSOUTH GEORGIA    -1.67375    0.05073 -32.993  < 2e-16 ***
## ---
## Signif. codes:  0 '***' 0.001 '**' 0.01 '*' 0.05 '.' 0.1 ' ' 1
##
## (Dispersion parameter for binomial family taken to be 1)
##
##     Null deviance: 10138.7  on 407  degrees of freedom
```

```
## Residual deviance:  1796.6  on 401  degrees of freedom
## AIC: 2846.5
##
## Number of Fisher Scoring iterations: 15
```

Calculate pseudo r squared

```
pseudo_r2(glm_1.6)

## [1] 0.8227982
```

Model with mobility

```
glm_1.7 <- glm(cbind(count,count_total - count)~ mobility, family = binomial(
link = logit), data = datsc)
summary(glm_1.7)

##
## Call:
## glm(formula = cbind(count, count_total - count) ~ mobility, family = binom
ial(link = logit),
##     data = datsc)
##
## Deviance Residuals:
##    Min      1Q  Median      3Q     Max
## -7.150  -4.470  -1.735   4.218  18.801
##
## Coefficients:
##             Estimate Std. Error  z value Pr(>|z|)
## (Intercept) -1.93728    0.01678 -115.465  < 2e-16 ***
## mobility    -0.13990    0.01742   -8.031 9.67e-16 ***
## ---
## Signif. codes:  0 '***' 0.001 '**' 0.01 '*' 0.05 '.' 0.1 ' ' 1
##
## (Dispersion parameter for binomial family taken to be 1)
##
##     Null deviance: 10139  on 407  degrees of freedom
## Residual deviance: 10074  on 406  degrees of freedom
## AIC: 11113
##
## Number of Fisher Scoring iterations: 6
```

Calculate pseudo r squared

```
pseudo_r2(glm_1.7)

## [1] 0.006427514
```

AIC summary

```
model.sel(list(glm_null, glm_1.1,glm_1.2,glm_1.3,glm_1.4,glm_1.5,glm_1.6,glm_
1.7))
```

```
## Model selection table
##     (Int) men_dpt_vss prp_day_trt_cch prp_day_wnt ttl_are    dns are
## 7 -0.7412                                                          +
## 5 -2.1790                                          -1.531
## 3 -2.0120                      0.3179
## 4 -1.9550                                 -0.3752
## 6 -1.8860                                                 0.2248
## 2 -1.9470      0.1574
## 8 -1.9370
## 1 -1.9140
##       mbl df    logLik    AICc   delta weight
## 7          7 -1416.247  2846.8    0.00      1
## 5          2 -3534.579  7073.2 4226.41      0
## 3          2 -5251.139 10506.3 7659.54      0
## 4          2 -5385.854 10775.7 7928.96      0
## 6          2 -5493.020 10990.1 8143.30      0
## 2          2 -5540.858 11085.7 8238.97      0
## 8 -0.1399  2 -5554.721 11113.5 8266.70      0
## 1          1 -5587.305 11176.6 8329.85      0
## Models ranked by AICc(x)
```

Model with lowest AIC

```
glm_2.0 <- glm(cbind(count,count_total - count)~ area, family = binomial(link
= logit), data = datsc)
summary(glm_2.0)

##
## Call:
## glm(formula = cbind(count, count_total - count) ~ area, family = binomial(
link = logit),
##     data = datsc)
##
## Deviance Residuals:
##     Min       1Q   Median       3Q      Max
## -5.6682  -1.2632  -0.3206   0.8205  14.3466
##
## Coefficients:
##                      Estimate Std. Error z value Pr(>|z|)
## (Intercept)          -0.74120    0.03082 -24.046  < 2e-16 ***
## areaCROZET            0.93836    0.04648  20.190  < 2e-16 ***
## areaFALKLANDS        -2.53931    0.10896 -23.305  < 2e-16 ***
## areaHIMI            -19.76403  339.92907  -0.058    0.954
## areaKERGUELEN        -4.47400    0.13102 -34.147  < 2e-16 ***
## areaPEMI             -0.72514    0.12934  -5.606 2.07e-08 ***
## areaSOUTH GEORGIA    -1.67375    0.05073 -32.993  < 2e-16 ***
## ---
## Signif. codes:  0 '***' 0.001 '**' 0.01 '*' 0.05 '.' 0.1 ' ' 1
##
## (Dispersion parameter for binomial family taken to be 1)
```

```
##
##     Null deviance: 10138.7  on 407  degrees of freedom
## Residual deviance:  1796.6  on 401  degrees of freedom
## AIC: 2846.5
##
## Number of Fisher Scoring iterations: 15
```

Calculate pseudo r squared

```
pseudo_r2(glm_2.0)

## [1] 0.8227982
```

Two terms models

Model with depth

```
glm_2.1 <- glm(cbind(count,count_total - count)~ area+ mean_depth_vessel, fam
ily = binomial(link = logit), data = datsc)
summary(glm_2.1)

##
## Call:
## glm(formula = cbind(count, count_total - count) ~ area + mean_depth_vessel
,
##     family = binomial(link = logit), data = datsc)
##
## Deviance Residuals:
##     Min       1Q   Median       3Q      Max
## -6.5691  -1.2541  -0.3407   0.8285  13.9252
##
## Coefficients:
##                    Estimate Std. Error z value Pr(>|z|)
## (Intercept)        -0.37216    0.05500  -6.766 1.32e-11 ***
## areaCROZET          0.43120    0.07793   5.533 3.14e-08 ***
## areaFALKLANDS      -2.80014    0.11401 -24.561  < 2e-16 ***
## areaHIMI          -20.07191  339.69826  -0.059    0.953
## areaKERGUELEN      -4.92025    0.14267 -34.487  < 2e-16 ***
## areaPEMI           -0.97006    0.13296  -7.296 2.97e-13 ***
## areaSOUTH GEORGIA  -2.05514    0.06996 -29.376  < 2e-16 ***
## mean_depth_vessel  -0.21997    0.02734  -8.047 8.49e-16 ***
## ---
## Signif. codes:  0 '***' 0.001 '**' 0.01 '*' 0.05 '.' 0.1 ' ' 1
##
## (Dispersion parameter for binomial family taken to be 1)
##
##     Null deviance: 10139  on 407  degrees of freedom
## Residual deviance:  1732  on 400  degrees of freedom
## AIC: 2783.9
##
## Number of Fisher Scoring iterations: 15
```

Calculate pseudo r squared

```
pseudo_r2(glm_2.1)

## [1] 0.8291712
```

Model with proportion of sets using trotlines-cachalotera

```
glm_2.2 <- glm(cbind(count,count_total - count)~ area+ prop_day_trotline_cach
, family = binomial(link = logit), data = datsc)
summary(glm_2.2)

##
## Call:
## glm(formula = cbind(count, count_total - count) ~ area + prop_day_trotline
_cach,
##     family = binomial(link = logit), data = datsc)
##
## Deviance Residuals:
##     Min       1Q   Median       3Q      Max
## -5.6181  -1.2632  -0.3380   0.8205  14.3967
##
## Coefficients:
##                         Estimate Std. Error z value Pr(>|z|)
## (Intercept)             -0.71280    0.07550  -9.441  < 2e-16 ***
## areaCROZET               0.90468    0.09402   9.622  < 2e-16 ***
## areaFALKLANDS           -2.54899    0.11160 -22.841  < 2e-16 ***
## areaHIMI               -19.79772  339.92908  -0.058    0.954
## areaKERGUELEN           -4.50768    0.15442 -29.190  < 2e-16 ***
## areaPEMI                -0.73448    0.13131  -5.593 2.23e-08 ***
## areaSOUTH GEORGIA       -1.70744    0.09620 -17.749  < 2e-16 ***
## prop_day_trotline_cach  -0.01309    0.03178  -0.412    0.680
## ---
## Signif. codes:  0 '***' 0.001 '**' 0.01 '*' 0.05 '.' 0.1 ' ' 1
##
## (Dispersion parameter for binomial family taken to be 1)
##
##     Null deviance: 10138.7  on 407  degrees of freedom
## Residual deviance:  1796.4  on 400  degrees of freedom
## AIC: 2848.3
##
## Number of Fisher Scoring iterations: 15
```

Calculate pseudo r squared

```
pseudo_r2(glm_2.2)

## [1] 0.8228149
```

Model with proportion of days in winter

```
glm_2.3 <- glm(cbind(count,count_total - count)~ area+ prop_day_winter, famil
y = binomial(link = logit), data = datsc)
summary(glm_2.3)
```

```
##
## Call:
## glm(formula = cbind(count, count_total - count) ~ area + prop_day_winter,
##     family = binomial(link = logit), data = datsc)
##
## Deviance Residuals:
##     Min       1Q   Median       3Q      Max
## -5.6533  -1.2702  -0.3162   0.8479  14.3695
##
## Coefficients:
##                      Estimate Std. Error z value Pr(>|z|)
## (Intercept)        -0.71726    0.04164 -17.224  < 2e-16 ***
## areaCROZET          0.92702    0.04832  19.184  < 2e-16 ***
## areaFALKLANDS      -2.56264    0.11237 -22.806  < 2e-16 ***
## areaHIMI          -19.80841  340.22988  -0.058    0.954
## areaKERGUELEN      -4.47986    0.13120 -34.144  < 2e-16 ***
## areaPEMI           -0.75414    0.13374  -5.639 1.71e-08 ***
## areaSOUTH GEORGIA  -1.73782    0.09062 -19.177  < 2e-16 ***
## prop_day_winter     0.03102    0.03632   0.854    0.393
## ---
## Signif. codes:  0 '***' 0.001 '**' 0.01 '*' 0.05 '.' 0.1 ' ' 1
##
## (Dispersion parameter for binomial family taken to be 1)
##
##     Null deviance: 10138.7  on 407  degrees of freedom
## Residual deviance:  1795.9  on 400  degrees of freedom
## AIC: 2847.8
##
## Number of Fisher Scoring iterations: 15
```

Calculate pseudo r squared

```
pseudo_r2(glm_2.3)

## [1] 0.8228702
```

Model with total area size

```
glm_2.4 <- glm(cbind(count,count_total - count)~ area+ total_area, family = b
inomial(link = logit), data = datsc)
summary(glm_2.4)

##
## Call:
## glm(formula = cbind(count, count_total - count) ~ area + total_area,
##     family = binomial(link = logit), data = datsc)
##
```

```
## Deviance Residuals:
##     Min       1Q   Median       3Q      Max
## -5.4913  -1.3167  -0.3327   0.8386  12.0413
##
## Coefficients:
##                      Estimate Std. Error z value Pr(>|z|)
## (Intercept)          -0.90119    0.03500 -25.748   <2e-16 ***
## areaCROZET            0.51776    0.06069   8.531   <2e-16 ***
## areaFALKLANDS        -2.84294    0.11344 -25.061   <2e-16 ***
## areaHIMI            -19.48490  332.50888  -0.059    0.953
## areaKERGUELEN        -3.22443    0.17384 -18.548   <2e-16 ***
## areaPEMI             -1.38618    0.14355  -9.656   <2e-16 ***
## areaSOUTH GEORGIA    -1.81143    0.05242 -34.554   <2e-16 ***
## total_area           -0.71820    0.06794 -10.570   <2e-16 ***
## ---
## Signif. codes:  0 '***' 0.001 '**' 0.01 '*' 0.05 '.' 0.1 ' ' 1
##
## (Dispersion parameter for binomial family taken to be 1)
##
##     Null deviance: 10138.7  on 407  degrees of freedom
## Residual deviance:  1686.2  on 400  degrees of freedom
## AIC: 2738.1
##
## Number of Fisher Scoring iterations: 15
```

Calculate pseudo r squared

```
pseudo_r2(glm_2.4)

## [1] 0.8336857
```

Model with density of vessels

```
glm_2.5 <- glm(cbind(count,count_total - count)~ area+ density, family = bino
mial(link = logit), data = datsc)
summary(glm_2.5)

##
## Call:
## glm(formula = cbind(count, count_total - count) ~ area + density,
##     family = binomial(link = logit), data = datsc)
##
## Deviance Residuals:
##     Min       1Q   Median       3Q      Max
## -5.6726  -1.2403  -0.2713   0.7784  14.3652
##
## Coefficients:
##                      Estimate Std. Error z value Pr(>|z|)
## (Intercept)          -0.80582    0.03428 -23.505  < 2e-16 ***
## areaCROZET            1.00309    0.04890  20.515  < 2e-16 ***
## areaFALKLANDS        -2.62522    0.11081 -23.691  < 2e-16 ***
```

```
## areaHIMI          -19.82612  339.00824  -0.058    0.953
## areaKERGUELEN      -4.56321    0.13260 -34.413  < 2e-16 ***
## areaPEMI           -0.70078    0.12951  -5.411 6.27e-08 ***
## areaSOUTH GEORGIA  -1.40967    0.07840 -17.981  < 2e-16 ***
## density            -0.16898    0.03883  -4.352 1.35e-05 ***
## ---
## Signif. codes:  0 '***' 0.001 '**' 0.01 '*' 0.05 '.' 0.1 ' ' 1
##
## (Dispersion parameter for binomial family taken to be 1)
##
##     Null deviance: 10138.7  on 407  degrees of freedom
## Residual deviance:  1777.5  on 400  degrees of freedom
## AIC: 2829.4
##
## Number of Fisher Scoring iterations: 15
```

Calculate pseudo r squared

```
pseudo_r2(glm_2.5)

## [1] 0.8246826
```

Model with mobility of vessels

```
glm_2.6 <- glm(cbind(count,count_total - count)~ area+ mobility, family = bin
omial(link = logit), data = datsc)
summary(glm_2.6)

##
## Call:
## glm(formula = cbind(count, count_total - count) ~ area + mobility,
##     family = binomial(link = logit), data = datsc)
##
## Deviance Residuals:
##     Min       1Q   Median       3Q      Max
## -5.5108  -1.2467  -0.3553   0.8037  14.3471
##
## Coefficients:
##                    Estimate Std. Error z value Pr(>|z|)
## (Intercept)        -0.79820    0.04618 -17.283  < 2e-16 ***
## areaCROZET          1.03420    0.07418  13.942  < 2e-16 ***
## areaFALKLANDS      -2.48103    0.11438 -21.692  < 2e-16 ***
## areaHIMI          -19.72904  340.11133  -0.058   0.9537
## areaKERGUELEN      -4.38410    0.14167 -30.946  < 2e-16 ***
## areaPEMI           -0.69468    0.13063  -5.318 1.05e-07 ***
## areaSOUTH GEORGIA  -1.66499    0.05100 -32.650  < 2e-16 ***
## mobility           -0.05306    0.03194  -1.661   0.0966 .
## ---
## Signif. codes:  0 '***' 0.001 '**' 0.01 '*' 0.05 '.' 0.1 ' ' 1
##
## (Dispersion parameter for binomial family taken to be 1)
```

```
##
##     Null deviance: 10138.7  on 407  degrees of freedom
## Residual deviance:  1793.8  on 400  degrees of freedom
## AIC: 2845.7
##
## Number of Fisher Scoring iterations: 15
```

Calculate pseudo r squared

```
pseudo_r2(glm_2.6)

## [1] 0.8230707
```

AIC summary

```
model.sel(list(glm_2.0,glm_2.1,glm_2.2,glm_2.3,glm_2.4,glm_2.5,glm_2.6))

## Model selection table
##     (Int) are men_dpt_vss prp_day_trt_cch prp_day_wnt ttl_are    dns
## 5 -0.9012   +                                         -0.7182
## 2 -0.3722   +       -0.22
## 6 -0.8058   +                                                 -0.169
## 7 -0.7982   +
## 1 -0.7412   +
## 4 -0.7173   +                                0.03102
## 3 -0.7128   +                  -0.01309
##        mbl df    logLik   AICc  delta weight
## 5           8 -1361.054 2738.5   0.00      1
## 2           8 -1383.940 2784.2  45.77      0
## 6           8 -1406.694 2829.7  91.28      0
## 7 -0.05306  8 -1414.865 2846.1 107.62      0
## 1           7 -1416.247 2846.8 108.31      0
## 4           8 -1415.881 2848.1 109.66      0
## 3           8 -1416.162 2848.7 110.22      0
## Models ranked by AICc(x)
```

Model with lowest AIC

```
glm_3.0 <- glm(cbind(count,count_total - count)~ area+ total_area, family = b
inomial(link = logit), data = datsc)
summary(glm_3.0)

##
## Call:
## glm(formula = cbind(count, count_total - count) ~ area + total_area,
##     family = binomial(link = logit), data = datsc)
##
## Deviance Residuals:
##     Min       1Q   Median       3Q      Max
## -5.4913  -1.3167  -0.3327   0.8386  12.0413
##
## Coefficients:
```

```
##                      Estimate Std. Error z value Pr(>|z|)    
## (Intercept)          -0.90119    0.03500 -25.748   <2e-16 ***
## areaCROZET            0.51776    0.06069   8.531   <2e-16 ***
## areaFALKLANDS        -2.84294    0.11344 -25.061   <2e-16 ***
## areaHIMI            -19.48490  332.50888  -0.059    0.953    
## areaKERGUELEN        -3.22443    0.17384 -18.548   <2e-16 ***
## areaPEMI             -1.38618    0.14355  -9.656   <2e-16 ***
## areaSOUTH GEORGIA    -1.81143    0.05242 -34.554   <2e-16 ***
## total_area           -0.71820    0.06794 -10.570   <2e-16 ***
## ---
## Signif. codes:  0 '***' 0.001 '**' 0.01 '*' 0.05 '.' 0.1 ' ' 1
## 
## (Dispersion parameter for binomial family taken to be 1)
## 
##     Null deviance: 10138.7  on 407  degrees of freedom
## Residual deviance:  1686.2  on 400  degrees of freedom
## AIC: 2738.1
## 
## Number of Fisher Scoring iterations: 15
```

Calculate pseudo r squared

```
pseudo_r2(glm_3.0)

## [1] 0.8336857
```

Three terms models

Model with depth

```
glm_3.1 <- glm(cbind(count,count_total - count)~ area+total_area+ mean_depth_
vessel, family = binomial(link = logit), data = datsc)
summary(glm_3.1)

## 
## Call:
## glm(formula = cbind(count, count_total - count) ~ area + total_area + 
##     mean_depth_vessel, family = binomial(link = logit), data = datsc)
## 
## Deviance Residuals: 
##     Min       1Q   Median       3Q      Max  
## -5.9803  -1.2770  -0.3276   0.8100  11.7525  
## 
## Coefficients:
##                      Estimate Std. Error z value Pr(>|z|)    
## (Intercept)          -0.54914    0.05844  -9.397  < 2e-16 ***
## areaCROZET            0.06096    0.08622   0.707    0.480    
## areaFALKLANDS        -3.08062    0.11834 -26.032  < 2e-16 ***
## areaHIMI            -19.77679  334.04480  -0.059    0.953    
## areaKERGUELEN        -3.70721    0.18466 -20.076  < 2e-16 ***
## areaPEMI             -1.59159    0.14664 -10.854  < 2e-16 ***
```

```
## areaSOUTH GEORGIA  -2.15974    0.07074 -30.529  < 2e-16 ***
## total_area         -0.68831    0.06805 -10.115  < 2e-16 ***
## mean_depth_vessel  -0.20543    0.02757  -7.451 9.29e-14 ***
## ---
## Signif. codes:  0 '***' 0.001 '**' 0.01 '*' 0.05 '.' 0.1 ' ' 1
##
## (Dispersion parameter for binomial family taken to be 1)
##
##     Null deviance: 10138.7  on 407  degrees of freedom
## Residual deviance:  1630.8  on 399  degrees of freedom
## AIC: 2684.7
##
## Number of Fisher Scoring iterations: 15
```

Calculate pseudo r squared

```
pseudo_r2(glm_3.1)

## [1] 0.8391491
```

Model with proportion of sets using trotlines-cachalotera

```
glm_3.2 <- glm(cbind(count,count_total - count)~ area+total_area+ prop_day_tr
otline_cach, family = binomial(link = logit), data = datsc)
summary(glm_3.2)

##
## Call:
## glm(formula = cbind(count, count_total - count) ~ area + total_area +
##     prop_day_trotline_cach, family = binomial(link = logit),
##     data = datsc)
##
## Deviance Residuals:
##     Min       1Q   Median       3Q      Max
## -5.5573  -1.3077  -0.3327   0.8430  11.8674
##
## Coefficients:
##                         Estimate Std. Error z value Pr(>|z|)
## (Intercept)             -0.98369    0.08023 -12.261  < 2e-16 ***
## areaCROZET               0.60564    0.09788   6.187 6.12e-10 ***
## areaFALKLANDS           -2.82761    0.11417 -24.766  < 2e-16 ***
## areaHIMI               -19.38663  332.21262  -0.058    0.953
## areaKERGUELEN           -3.11009    0.20052 -15.510  < 2e-16 ***
## areaPEMI                -1.37138    0.14416  -9.513  < 2e-16 ***
## areaSOUTH GEORGIA       -1.71901    0.09632 -17.846  < 2e-16 ***
## total_area              -0.72996    0.06872 -10.622  < 2e-16 ***
## prop_day_trotline_cach   0.03690    0.03223   1.145    0.252
## ---
## Signif. codes:  0 '***' 0.001 '**' 0.01 '*' 0.05 '.' 0.1 ' ' 1
##
## (Dispersion parameter for binomial family taken to be 1)
```

```
##
##     Null deviance: 10138.7  on 407  degrees of freedom
## Residual deviance:  1684.9  on 399  degrees of freedom
## AIC: 2738.8
##
## Number of Fisher Scoring iterations: 15
```

Calculate pseudo r squared

```
pseudo_r2(glm_3.2)

## [1] 0.8338151
```

Model with mobility of vessels

```
glm_3.3 <- glm(cbind(count,count_total - count)~ area+total_area+mobility, fa
mily = binomial(link = logit), data = datsc)
summary(glm_3.3)

##
## Call:
## glm(formula = cbind(count, count_total - count) ~ area + total_area +
##     mobility, family = binomial(link = logit), data = datsc)
##
## Deviance Residuals:
##     Min       1Q   Median       3Q      Max
## -5.4655  -1.3071  -0.3099   0.8674  12.0301
##
## Coefficients:
##                      Estimate Std. Error z value Pr(>|z|)
## (Intercept)          -0.96989    0.04939 -19.638  < 2e-16 ***
## areaCROZET            0.62947    0.08299   7.585 3.32e-14 ***
## areaFALKLANDS        -2.78013    0.11784 -23.593  < 2e-16 ***
## areaHIMI            -19.44333  332.41102  -0.058    0.953
## areaKERGUELEN        -3.11611    0.18185 -17.136  < 2e-16 ***
## areaPEMI             -1.35272    0.14446  -9.364  < 2e-16 ***
## areaSOUTH GEORGIA  -1.80104    0.05265 -34.209  < 2e-16 ***
## total_area           -0.72207    0.06794 -10.629  < 2e-16 ***
## mobility             -0.06308    0.03190  -1.977    0.048 *
## ---
## Signif. codes:  0 '***' 0.001 '**' 0.01 '*' 0.05 '.' 0.1 ' ' 1
##
## (Dispersion parameter for binomial family taken to be 1)
##
##     Null deviance: 10138.7  on 407  degrees of freedom
## Residual deviance:  1682.3  on 399  degrees of freedom
## AIC: 2736.2
##
## Number of Fisher Scoring iterations: 15
```

Calculate pseudo r squared

```r
pseudo_r2(glm_3.3)

## [1] 0.8340719
```

Model with density of vessels

```r
glm_3.4 <- glm(cbind(count,count_total - count)~ area+total_area+ density, fa
mily = binomial(link = logit), data = datsc)
summary(glm_3.4)

##
## Call:
## glm(formula = cbind(count, count_total - count) ~ area + total_area +
##     density, family = binomial(link = logit), data = datsc)
##
## Deviance Residuals:
##     Min       1Q   Median       3Q      Max
## -5.7125  -1.2832  -0.1658   0.8292  11.7680
##
## Coefficients:
##                     Estimate Std. Error z value Pr(>|z|)
## (Intercept)         -1.01761    0.03944 -25.800  < 2e-16 ***
## areaCROZET           0.55937    0.06124   9.134  < 2e-16 ***
## areaFALKLANDS       -2.99444    0.11548 -25.929  < 2e-16 ***
## areaHIMI           -19.55078  329.84070  -0.059    0.953
## areaKERGUELEN       -3.20519    0.17344 -18.480  < 2e-16 ***
## areaPEMI            -1.43163    0.14361  -9.969  < 2e-16 ***
## areaSOUTH GEORGIA  -1.44424    0.07628 -18.932  < 2e-16 ***
## total_area          -0.81136    0.06984 -11.618  < 2e-16 ***
## density             -0.25567    0.03955  -6.465 1.01e-10 ***
## ---
## Signif. codes:  0 '***' 0.001 '**' 0.01 '*' 0.05 '.' 0.1 ' ' 1
##
## (Dispersion parameter for binomial family taken to be 1)
##
##     Null deviance: 10138.7  on 407  degrees of freedom
## Residual deviance:  1643.9  on 399  degrees of freedom
## AIC: 2697.8
##
## Number of Fisher Scoring iterations: 15
```

Calculate pseudo r squared

```r
pseudo_r2(glm_3.4)

## [1] 0.8378608
```

Model with proportion of days in winter

```r
glm_3.5 <- glm(cbind(count,count_total - count)~ area+total_area+ prop_day_wi
nter, family = binomial(link = logit), data = datsc)
summary(glm_3.5)
```

```
##
## Call:
## glm(formula = cbind(count, count_total - count) ~ area + total_area +
##     prop_day_winter, family = binomial(link = logit), data = datsc)
##
## Deviance Residuals:
##     Min       1Q   Median       3Q      Max
## -5.5537  -1.3070  -0.3439   0.8605  12.0515
##
## Coefficients:
##                      Estimate Std. Error z value Pr(>|z|)
## (Intercept)         -0.85131    0.04410 -19.305  < 2e-16 ***
## areaCROZET           0.48575    0.06308   7.701 1.35e-14 ***
## areaFALKLANDS       -2.90033    0.11772 -24.638  < 2e-16 ***
## areaHIMI           -19.58247  331.83768  -0.059   0.9529
## areaKERGUELEN       -3.21856    0.17363 -18.536  < 2e-16 ***
## areaPEMI            -1.45975    0.14888  -9.805  < 2e-16 ***
## areaSOUTH GEORGIA  -1.95405    0.09326 -20.953  < 2e-16 ***
## total_area          -0.73032    0.06825 -10.700  < 2e-16 ***
## prop_day_winter      0.06802    0.03669   1.854   0.0638 .
## ---
## Signif. codes:  0 '***' 0.001 '**' 0.01 '*' 0.05 '.' 0.1 ' ' 1
##
## (Dispersion parameter for binomial family taken to be 1)
##
##     Null deviance: 10138.7  on 407  degrees of freedom
## Residual deviance:  1682.8  on 399  degrees of freedom
## AIC: 2736.7
##
## Number of Fisher Scoring iterations: 15
```

Calculate pseudo r squared

```
pseudo_r2(glm_3.5)

## [1] 0.8340257
```

AIC summary

```
model.sel(list(glm_3.0, glm_3.1,glm_3.2,glm_3.3,glm_3.4,glm_3.5))

## Model selection table
##     (Int) are ttl_are men_dpt_vss prp_day_trt_cch      mbl     dns
## 2 -0.5491   + -0.6883     -0.2054
## 5 -1.0180   + -0.8114                                      -0.2557
## 4 -0.9699   + -0.7221                             -0.06308
## 6 -0.8513   + -0.7303
## 1 -0.9012   + -0.7182
## 3 -0.9837   + -0.7300                      0.0369
##   prp_day_wnt df    logLik   AICc delta weight
## 2              9 -1333.358 2685.2  0.00  0.999
```

```
## 5              9 -1339.889 2698.2 13.06  0.001
## 4              9 -1359.096 2736.6 51.48  0.000
## 6     0.06802  9 -1359.330 2737.1 51.94  0.000
## 1              8 -1361.054 2738.5 53.30  0.000
## 3              9 -1360.398 2739.2 54.08  0.000
## Models ranked by AICc(x)
```

Model with lowest AIC

```
glm_4.0 <- glm(cbind(count,count_total - count)~ area+total_area+mean_depth_v
essel, family = binomial(link = logit), data = datsc)
summary(glm_4.0)
```

```
##
## Call:
## glm(formula = cbind(count, count_total - count) ~ area + total_area +
##     mean_depth_vessel, family = binomial(link = logit), data = datsc)
##
## Deviance Residuals:
##     Min       1Q   Median       3Q      Max
## -5.9803  -1.2770  -0.3276   0.8100  11.7525
##
## Coefficients:
##                     Estimate Std. Error z value Pr(>|z|)
## (Intercept)        -0.54914    0.05844  -9.397  < 2e-16 ***
## areaCROZET          0.06096    0.08622   0.707    0.480
## areaFALKLANDS      -3.08062    0.11834 -26.032  < 2e-16 ***
## areaHIMI          -19.77679  334.04480  -0.059    0.953
## areaKERGUELEN      -3.70721    0.18466 -20.076  < 2e-16 ***
## areaPEMI           -1.59159    0.14664 -10.854  < 2e-16 ***
## areaSOUTH GEORGIA  -2.15974    0.07074 -30.529  < 2e-16 ***
## total_area         -0.68831    0.06805 -10.115  < 2e-16 ***
## mean_depth_vessel  -0.20543    0.02757  -7.451 9.29e-14 ***
## ---
## Signif. codes:  0 '***' 0.001 '**' 0.01 '*' 0.05 '.' 0.1 ' ' 1
##
## (Dispersion parameter for binomial family taken to be 1)
##
##     Null deviance: 10138.7  on 407  degrees of freedom
## Residual deviance:  1630.8  on 399  degrees of freedom
## AIC: 2684.7
##
## Number of Fisher Scoring iterations: 15
```

Calculate pseudo r squared

```
pseudo_r2(glm_4.0)
```

```
## [1] 0.8391491
```

Four terms models

Model with density of vessels

```
glm_4.1 <- glm(cbind(count,count_total - count)~ area+total_area+mean_depth_v
essel+density, family = binomial(link = logit), data = datsc)
summary(glm_4.1)
```

```
##
## Call:
## glm(formula = cbind(count, count_total - count) ~ area + total_area +
##     mean_depth_vessel + density, family = binomial(link = logit),
##     data = datsc)
##
## Deviance Residuals:
##     Min       1Q   Median       3Q      Max
## -6.0685  -1.2501  -0.2580   0.8158  11.3664
##
## Coefficients:
##                      Estimate Std. Error z value Pr(>|z|)
## (Intercept)          -0.62706    0.05955 -10.530  < 2e-16 ***
## areaCROZET            0.02777    0.08689   0.320    0.749
## areaFALKLANDS        -3.31360    0.12208 -27.143  < 2e-16 ***
## areaHIMI            -19.91098  331.03104  -0.060    0.952
## areaKERGUELEN        -3.77624    0.18454 -20.463  < 2e-16 ***
## areaPEMI             -1.68194    0.14689 -11.450  < 2e-16 ***
## areaSOUTH GEORGIA    -1.77071    0.08546 -20.721  < 2e-16 ***
## total_area           -0.79791    0.06993 -11.409  < 2e-16 ***
## mean_depth_vessel    -0.24383    0.02835  -8.602  < 2e-16 ***
## density              -0.31430    0.04049  -7.762 8.33e-15 ***
## ---
## Signif. codes:  0 '***' 0.001 '**' 0.01 '*' 0.05 '.' 0.1 ' ' 1
##
## (Dispersion parameter for binomial family taken to be 1)
##
##     Null deviance: 10138.7  on 407  degrees of freedom
## Residual deviance:  1569.7  on 398  degrees of freedom
## AIC: 2625.6
##
## Number of Fisher Scoring iterations: 15
```

Calculate pseudo r squared

```
pseudo_r2(glm_4.1)
```

```
## [1] 0.8451773
```

Model with proportion of sets using trotlines-cachalotera

```
glm_4.2 <- glm(cbind(count,count_total - count)~ area+total_area+mean_depth_v
essel+prop_day_trotline_cach, family = binomial(link = logit), data = datsc)
summary(glm_4.2)
```

```
##
## Call:
## glm(formula = cbind(count, count_total - count) ~ area + total_area +
##     mean_depth_vessel + prop_day_trotline_cach, family = binomial(link = l
ogit),
##     data = datsc)
##
## Deviance Residuals:
##     Min       1Q   Median       3Q      Max
## -6.1383  -1.2769  -0.3133   0.8012  11.5564
##
## Coefficients:
##                         Estimate Std. Error z value Pr(>|z|)
## (Intercept)             -0.64071    0.09205  -6.960 3.39e-12 ***
## areaCROZET               0.15790    0.11441   1.380    0.168
## areaFALKLANDS           -3.06303    0.11899 -25.742  < 2e-16 ***
## areaHIMI               -19.66689  333.71352  -0.059    0.953
## areaKERGUELEN           -3.58017    0.20930 -17.106  < 2e-16 ***
## areaPEMI                -1.57611    0.14717 -10.709  < 2e-16 ***
## areaSOUTH GEORGIA       -2.05709    0.10654 -19.309  < 2e-16 ***
## total_area              -0.70175    0.06884 -10.193  < 2e-16 ***
## mean_depth_vessel       -0.20630    0.02761  -7.473 7.84e-14 ***
## prop_day_trotline_cach   0.04156    0.03221   1.290    0.197
## ---
## Signif. codes:  0 '***' 0.001 '**' 0.01 '*' 0.05 '.' 0.1 ' ' 1
##
## (Dispersion parameter for binomial family taken to be 1)
##
##     Null deviance: 10138.7  on 407  degrees of freedom
## Residual deviance:  1629.2  on 398  degrees of freedom
## AIC: 2685
##
## Number of Fisher Scoring iterations: 15
```

Calculate pseudo r squared

```
pseudo_r2(glm_4.2)
```

```
## [1] 0.8393135
```

Model with mobility of vessels

```
glm_4.3 <- glm(cbind(count,count_total - count)~ area+total_area+mean_depth_v
essel+mobility, family = binomial(link = logit), data = datsc)
summary(glm_4.3)
```

```
##
## Call:
## glm(formula = cbind(count, count_total - count) ~ area + total_area +
##     mean_depth_vessel + mobility, family = binomial(link = logit),
##     data = datsc)
##
## Deviance Residuals:
##     Min       1Q   Median       3Q      Max
## -5.8253  -1.2723  -0.3707   0.8529  11.7470
##
## Coefficients:
##                    Estimate Std. Error z value Pr(>|z|)
## (Intercept)        -0.60450    0.06968  -8.675  < 2e-16 ***
## areaCROZET          0.14998    0.10557   1.421    0.155
## areaFALKLANDS      -3.03146    0.12307 -24.632  < 2e-16 ***
## areaHIMI          -19.74253  333.93343  -0.059    0.953
## areaKERGUELEN      -3.62156    0.19347 -18.719  < 2e-16 ***
## areaPEMI           -1.56290    0.14781 -10.574  < 2e-16 ***
## areaSOUTH GEORGIA  -2.14838    0.07125 -30.152  < 2e-16 ***
## total_area         -0.69140    0.06805 -10.160  < 2e-16 ***
## mean_depth_vessel  -0.20251    0.02763  -7.329 2.31e-13 ***
## mobility           -0.04675    0.03195  -1.463    0.143
## ---
## Signif. codes:  0 '***' 0.001 '**' 0.01 '*' 0.05 '.' 0.1 ' ' 1
##
## (Dispersion parameter for binomial family taken to be 1)
##
##     Null deviance: 10138.7  on 407  degrees of freedom
## Residual deviance:  1628.7  on 398  degrees of freedom
## AIC: 2684.6
##
## Number of Fisher Scoring iterations: 15
```

Calculate pseudo r squared

```
pseudo_r2(glm_4.3)

## [1] 0.8393604
```

Model with proportion of days in winter

```
glm_4.4 <- glm(cbind(count,count_total - count)~ area+total_area+mean_depth_v
essel+prop_day_winter, family = binomial(link = logit), data = datsc)
summary(glm_4.4)

##
## Call:
## glm(formula = cbind(count, count_total - count) ~ area + total_area +
##     mean_depth_vessel + prop_day_winter, family = binomial(link = logit),
##     data = datsc)
##
```

```
## Deviance Residuals:
##     Min       1Q   Median       3Q      Max
## -5.9375  -1.2853  -0.3893   0.8274  11.7627
##
## Coefficients:
##                     Estimate Std. Error z value Pr(>|z|)
## (Intercept)         -0.50133    0.06409  -7.822 5.18e-15 ***
## areaCROZET           0.03034    0.08787   0.345   0.7299
## areaFALKLANDS       -3.13558    0.12227 -25.645  < 2e-16 ***
## areaHIMI           -19.87134  333.30974  -0.060   0.9525
## areaKERGUELEN       -3.70087    0.18446 -20.063  < 2e-16 ***
## areaPEMI            -1.66220    0.15165 -10.961  < 2e-16 ***
## areaSOUTH GEORGIA   -2.29800    0.10432 -22.029  < 2e-16 ***
## total_area          -0.70035    0.06836 -10.244  < 2e-16 ***
## mean_depth_vessel   -0.20514    0.02757  -7.440 1.01e-13 ***
## prop_day_winter      0.06613    0.03657   1.808   0.0706 .
## ---
## Signif. codes:  0 '***' 0.001 '**' 0.01 '*' 0.05 '.' 0.1 ' ' 1
##
## (Dispersion parameter for binomial family taken to be 1)
##
##     Null deviance: 10138.7  on 407  degrees of freedom
## Residual deviance:  1627.5  on 398  degrees of freedom
## AIC: 2683.4
##
## Number of Fisher Scoring iterations: 15
```

Calculate pseudo r squared

```
pseudo_r2(glm_4.4)

## [1] 0.8394724
```

AIC summary

```
model.sel(list(glm_4.0,glm_4.1,glm_4.2,glm_4.3,glm_4.4))

## Model selection table
##     (Int) are men_dpt_vss ttl_are     dns prp_day_trt_cch      mbl
## 2 -0.6271   +     -0.2438 -0.7979 -0.3143
## 5 -0.5013   +     -0.2051 -0.7003
## 4 -0.6045   +     -0.2025 -0.6914                         -0.04675
## 1 -0.5491   +     -0.2054 -0.6883
## 3 -0.6407   +     -0.2063 -0.7018                 0.04156
##   prp_day_wnt df    logLik   AICc delta weight
## 2             10 -1302.799 2626.2  0.00      1
## 5     0.06613 10 -1331.719 2684.0 57.84      0
## 4             10 -1332.287 2685.1 58.98      0
## 1              9 -1333.358 2685.2 59.02      0
## 3             10 -1332.525 2685.6 59.45      0
## Models ranked by AICc(x)
```

Model with lowest AIC

```
glm_5.0 <- glm(cbind(count,count_total - count)~ area+total_area+mean_depth_v
essel+density, family = binomial(link = logit), data = datsc)
summary(glm_5.0)
```

```
##
## Call:
## glm(formula = cbind(count, count_total - count) ~ area + total_area +
##     mean_depth_vessel + density, family = binomial(link = logit),
##     data = datsc)
##
## Deviance Residuals:
##     Min       1Q   Median       3Q      Max
## -6.0685  -1.2501  -0.2580   0.8158  11.3664
##
## Coefficients:
##                      Estimate Std. Error z value Pr(>|z|)
## (Intercept)          -0.62706    0.05955 -10.530  < 2e-16 ***
## areaCROZET            0.02777    0.08689   0.320    0.749
## areaFALKLANDS        -3.31360    0.12208 -27.143  < 2e-16 ***
## areaHIMI            -19.91098  331.03104  -0.060    0.952
## areaKERGUELEN        -3.77624    0.18454 -20.463  < 2e-16 ***
## areaPEMI             -1.68194    0.14689 -11.450  < 2e-16 ***
## areaSOUTH GEORGIA    -1.77071    0.08546 -20.721  < 2e-16 ***
## total_area           -0.79791    0.06993 -11.409  < 2e-16 ***
## mean_depth_vessel    -0.24383    0.02835  -8.602  < 2e-16 ***
## density              -0.31430    0.04049  -7.762 8.33e-15 ***
## ---
## Signif. codes:  0 '***' 0.001 '**' 0.01 '*' 0.05 '.' 0.1 ' ' 1
##
## (Dispersion parameter for binomial family taken to be 1)
##
##     Null deviance: 10138.7  on 407  degrees of freedom
## Residual deviance:  1569.7  on 398  degrees of freedom
## AIC: 2625.6
##
## Number of Fisher Scoring iterations: 15
```

Calculate pseudo r squared

```
pseudo_r2(glm_5.0)
```

```
## [1] 0.8451773
```

Five terms models

Model with mobility

```r
glm_5.1 <- glm(cbind(count,count_total - count)~ area+total_area+mean_depth_v
essel+density+mobility, family = binomial(link = logit), data = datsc)
summary(glm_5.1)
```

```
##
## Call:
## glm(formula = cbind(count, count_total - count) ~ area + total_area +
##     mean_depth_vessel + density + mobility, family = binomial(link = logit
),
##     data = datsc)
##
## Deviance Residuals:
##     Min       1Q   Median       3Q      Max
## -5.8582  -1.2519  -0.2447   0.8501  11.3511
##
## Coefficients:
##                    Estimate Std. Error z value Pr(>|z|)
## (Intercept)        -0.70357    0.07103  -9.905  < 2e-16 ***
## areaCROZET          0.14849    0.10614   1.399   0.1618
## areaFALKLANDS      -3.24792    0.12645 -25.685  < 2e-16 ***
## areaHIMI          -19.86714  330.82816  -0.060   0.9521
## areaKERGUELEN      -3.66179    0.19301 -18.972  < 2e-16 ***
## areaPEMI           -1.64475    0.14791 -11.120  < 2e-16 ***
## areaSOUTH GEORGIA  -1.74703    0.08649 -20.199  < 2e-16 ***
## total_area         -0.80416    0.07000 -11.489  < 2e-16 ***
## mean_depth_vessel  -0.24073    0.02839  -8.479  < 2e-16 ***
## density            -0.31958    0.04059  -7.874 3.43e-15 ***
## mobility           -0.06387    0.03219  -1.984   0.0472 *
## ---
## Signif. codes:  0 '***' 0.001 '**' 0.01 '*' 0.05 '.' 0.1 ' ' 1
##
## (Dispersion parameter for binomial family taken to be 1)
##
##     Null deviance: 10138.7  on 407  degrees of freedom
## Residual deviance:  1565.8  on 397  degrees of freedom
## AIC: 2623.7
##
## Number of Fisher Scoring iterations: 15
```

Calculate pseudo r squared

```r
pseudo_r2(glm_5.1)
```

```
## [1] 0.8455663
```

Model with proportion of days in winter

```r
glm_5.2 <- glm(cbind(count,count_total - count)~ area+total_area+mean_depth_v
essel+density+prop_day_winter, family = binomial(link = logit), data = datsc)
summary(glm_5.2)
```

```
##
## Call:
## glm(formula = cbind(count, count_total - count) ~ area + total_area +
##     mean_depth_vessel + density + prop_day_winter, family = binomial(link
= logit),
##     data = datsc)
##
## Deviance Residuals:
##     Min       1Q   Median       3Q      Max
## -6.0501  -1.2593  -0.2609   0.8461  11.3754
##
## Coefficients:
##                    Estimate Std. Error z value Pr(>|z|)
## (Intercept)        -0.60755    0.06575  -9.240  < 2e-16 ***
## areaCROZET          0.01636    0.08840   0.185    0.853
## areaFALKLANDS      -3.33177    0.12484 -26.689  < 2e-16 ***
## areaHIMI          -19.94585  330.75236  -0.060    0.952
## areaKERGUELEN      -3.77300    0.18450 -20.450  < 2e-16 ***
## areaPEMI           -1.70909    0.15194 -11.249  < 2e-16 ***
## areaSOUTH GEORGIA  -1.82908    0.11958 -15.296  < 2e-16 ***
## total_area         -0.80124    0.07009 -11.432  < 2e-16 ***
## mean_depth_vessel  -0.24319    0.02836  -8.576  < 2e-16 ***
## density            -0.31027    0.04090  -7.587 3.28e-14 ***
## prop_day_winter     0.02581    0.03693   0.699    0.485
## ---
## Signif. codes:  0 '***' 0.001 '**' 0.01 '*' 0.05 '.' 0.1 ' ' 1
##
## (Dispersion parameter for binomial family taken to be 1)
##
##     Null deviance: 10138.7  on 407  degrees of freedom
## Residual deviance:  1569.2  on 397  degrees of freedom
## AIC: 2627.1
##
## Number of Fisher Scoring iterations: 15
```

Calculate pseudo r squared

```
pseudo_r2(glm_5.2)

## [1] 0.8452255
```

Model with proportion of sets using trotlines-cachalotera

```
glm_5.3 <- glm(cbind(count,count_total - count)~ area+total_area+mean_depth_v
essel+density+prop_day_trotline_cach, family = binomial(link = logit), data =
datsc)
summary(glm_5.3)

##
## Call:
## glm(formula = cbind(count, count_total - count) ~ area + total_area +
```

```
##     mean_depth_vessel + density + prop_day_trotline_cach, family = binomia
l(link = logit),
##     data = datsc)
##
## Deviance Residuals:
##     Min       1Q   Median       3Q      Max
## -6.1295  -1.2506  -0.2598   0.8144  11.2935
##
## Coefficients:
##                         Estimate Std. Error z value Pr(>|z|)
## (Intercept)             -0.66212    0.09194  -7.201 5.96e-13 ***
## areaCROZET               0.06556    0.11510   0.570    0.569
## areaFALKLANDS           -3.30426    0.12338 -26.780  < 2e-16 ***
## areaHIMI               -19.86734  330.88658  -0.060    0.952
## areaKERGUELEN           -3.72655    0.20951 -17.787  < 2e-16 ***
## areaPEMI                -1.67525    0.14751 -11.357  < 2e-16 ***
## areaSOUTH GEORGIA       -1.73335    0.11347 -15.275  < 2e-16 ***
## total_area              -0.80246    0.07052 -11.379  < 2e-16 ***
## mean_depth_vessel       -0.24397    0.02836  -8.604  < 2e-16 ***
## density                 -0.31227    0.04069  -7.674 1.67e-14 ***
## prop_day_trotline_cach   0.01615    0.03224   0.501    0.617
## ---
## Signif. codes:  0 '***' 0.001 '**' 0.01 '*' 0.05 '.' 0.1 ' ' 1
##
## (Dispersion parameter for binomial family taken to be 1)
##
##     Null deviance: 10138.7  on 407  degrees of freedom
## Residual deviance:  1569.5  on 397  degrees of freedom
## AIC: 2627.3
##
## Number of Fisher Scoring iterations: 15
```

Calculate pseudo r squared

```
pseudo_r2(glm_5.3)

## [1] 0.845202
```

AIC summary

```
model.sel(list(glm_5.0,glm_5.1,glm_5.2,glm_5.3))

## Model selection table
##     (Int) are     dns men_dpt_vss ttl_are      mbl prp_day_wnt
## 2 -0.7036   + -0.3196     -0.2407 -0.8042 -0.06387
## 1 -0.6271   + -0.3143     -0.2438 -0.7979
## 3 -0.6075   + -0.3103     -0.2432 -0.8012              0.02581
## 4 -0.6621   + -0.3123     -0.2440 -0.8025
##   prp_day_trt_cch df    logLik   AICc delta weight
## 2                 11 -1300.827 2624.3  0.00  0.576
## 1                 10 -1302.799 2626.2  1.83  0.231
```

```
## 3                  11 -1302.554 2627.8  3.45  0.102
## 4         0.01615 11 -1302.673 2628.0  3.69  0.091
## Models ranked by AICc(x)
```

Model with lowest AIC

```
glm_6.0 <- glm(cbind(count,count_total - count)~ area+total_area+mean_depth_v
essel+density+mobility, family = binomial(link = logit), data = datsc)
summary(glm_6.0)
```

```
## 
## Call:
## glm(formula = cbind(count, count_total - count) ~ area + total_area + 
##     mean_depth_vessel + density + mobility, family = binomial(link = logit
),
##     data = datsc)
## 
## Deviance Residuals: 
##     Min       1Q   Median       3Q      Max  
## -5.8582  -1.2519  -0.2447   0.8501  11.3511  
## 
## Coefficients:
##                    Estimate Std. Error z value Pr(>|z|)    
## (Intercept)        -0.70357    0.07103  -9.905  < 2e-16 ***
## areaCROZET          0.14849    0.10614   1.399   0.1618    
## areaFALKLANDS      -3.24792    0.12645 -25.685  < 2e-16 ***
## areaHIMI          -19.86714  330.82816  -0.060   0.9521    
## areaKERGUELEN      -3.66179    0.19301 -18.972  < 2e-16 ***
## areaPEMI           -1.64475    0.14791 -11.120  < 2e-16 ***
## areaSOUTH GEORGIA  -1.74703    0.08649 -20.199  < 2e-16 ***
## total_area         -0.80416    0.07000 -11.489  < 2e-16 ***
## mean_depth_vessel  -0.24073    0.02839  -8.479  < 2e-16 ***
## density            -0.31958    0.04059  -7.874 3.43e-15 ***
## mobility           -0.06387    0.03219  -1.984   0.0472 *  
## ---
## Signif. codes:  0 '***' 0.001 '**' 0.01 '*' 0.05 '.' 0.1 ' ' 1
## 
## (Dispersion parameter for binomial family taken to be 1)
## 
##     Null deviance: 10138.7  on 407  degrees of freedom
## Residual deviance:  1565.8  on 397  degrees of freedom
## AIC: 2623.7
## 
## Number of Fisher Scoring iterations: 15
```

Calculate pseudo r squared

```
pseudo_r2(glm_6.0)
```

```
## [1] 0.8455663
```

Six terms models

Model with proportion of days in winter

```
glm_6.1 <- glm(cbind(count,count_total - count)~ area+total_area+mean_depth_v
essel+density+mobility+prop_day_winter, family = binomial(link = logit), data
= datsc)
summary(glm_6.1)
```

```
##
## Call:
## glm(formula = cbind(count, count_total - count) ~ area + total_area +
##     mean_depth_vessel + density + mobility + prop_day_winter,
##     family = binomial(link = logit), data = datsc)
##
## Deviance Residuals:
##     Min       1Q   Median       3Q      Max
## -5.8479  -1.2515  -0.2361   0.8542  11.3587
##
## Coefficients:
##                     Estimate Std. Error z value Pr(>|z|)
## (Intercept)         -0.68645    0.07738  -8.871  < 2e-16 ***
## areaCROZET           0.13695    0.10814   1.266   0.2054
## areaFALKLANDS       -3.26344    0.12955 -25.191  < 2e-16 ***
## areaHIMI           -19.89586  330.60352  -0.060   0.9520
## areaKERGUELEN       -3.66154    0.19294 -18.977  < 2e-16 ***
## areaPEMI            -1.66712    0.15326 -10.878  < 2e-16 ***
## areaSOUTH GEORGIA  -1.79425    0.12113 -14.813  < 2e-16 ***
## total_area          -0.80669    0.07014 -11.502  < 2e-16 ***
## mean_depth_vessel  -0.24026    0.02840  -8.459  < 2e-16 ***
## density             -0.31625    0.04102  -7.709 1.27e-14 ***
## mobility            -0.06257    0.03227  -1.939   0.0525 .
## prop_day_winter      0.02064    0.03704   0.557   0.5773
## ---
## Signif. codes:  0 '***' 0.001 '**' 0.01 '*' 0.05 '.' 0.1 ' ' 1
##
## (Dispersion parameter for binomial family taken to be 1)
##
##     Null deviance: 10138.7  on 407  degrees of freedom
## Residual deviance:  1565.4  on 396  degrees of freedom
## AIC: 2625.3
##
## Number of Fisher Scoring iterations: 15
```

Calculate pseudo r squared

```
pseudo_r2(glm_6.1)

## [1] 0.8455969
```

Model with proportion of sets using trotlines-cachalotera

```r
glm_6.2 <- glm(cbind(count,count_total - count)~ area+total_area+mean_depth_v
essel+density+mobility+prop_day_trotline_cach, family = binomial(link = logit
), data = datsc)
summary(glm_6.2)
```

```
##
## Call:
## glm(formula = cbind(count, count_total - count) ~ area + total_area +
##     mean_depth_vessel + density + mobility + prop_day_trotline_cach,
##     family = binomial(link = logit), data = datsc)
##
## Deviance Residuals:
##     Min       1Q   Median       3Q      Max
## -5.9333  -1.2516  -0.2490   0.8473  11.2534
##
## Coefficients:
##                         Estimate Std. Error z value Pr(>|z|)
## (Intercept)             -0.75259    0.10231  -7.356 1.89e-13 ***
## areaCROZET               0.20250    0.13362   1.515   0.1296
## areaFALKLANDS           -3.23312    0.12831 -25.198  < 2e-16 ***
## areaHIMI               -19.80759  330.63090  -0.060   0.9522
## areaKERGUELEN           -3.59248    0.21920 -16.389  < 2e-16 ***
## areaPEMI                -1.63461    0.14872 -10.991  < 2e-16 ***
## areaSOUTH GEORGIA       -1.69627    0.11531 -14.711  < 2e-16 ***
## total_area              -0.81026    0.07059 -11.478  < 2e-16 ***
## mean_depth_vessel       -0.24084    0.02841  -8.479  < 2e-16 ***
## density                 -0.31704    0.04077  -7.777 7.43e-15 ***
## mobility                -0.06560    0.03227  -2.033   0.0421 *
## prop_day_trotline_cach   0.02165    0.03248   0.667   0.5051
## ---
## Signif. codes:  0 '***' 0.001 '**' 0.01 '*' 0.05 '.' 0.1 ' ' 1
##
## (Dispersion parameter for binomial family taken to be 1)
##
##     Null deviance: 10138.7  on 407  degrees of freedom
## Residual deviance:  1565.3  on 396  degrees of freedom
## AIC: 2625.2
##
## Number of Fisher Scoring iterations: 15
```

Calculate pseudo r squared

```r
pseudo_r2(glm_6.2)
```

```
## [1] 0.8456101
```

AIC summary

```r
model.sel(list(glm_6.0,glm_6.1,glm_6.2))
```

```
## Model selection table
##     (Int) are     dns men_dpt_vss      mbl ttl_are prp_day_wnt
## 1 -0.7036   + -0.3196     -0.2407 -0.06387 -0.8042
## 3 -0.7526   + -0.3170     -0.2408 -0.06560 -0.8103
## 2 -0.6864   + -0.3162     -0.2403 -0.06257 -0.8067     0.02064
##   prp_day_trt_cch df    logLik   AICc delta weight
## 1                 11 -1300.827 2624.3  0.00  0.545
## 3         0.02165 12 -1300.605 2626.0  1.68  0.235
## 2                 12 -1300.672 2626.1  1.81  0.220
## Models ranked by AICc(x)
```

FINAL MODEL without interactions

```
glm_7.0 <- glm(cbind(count,count_total - count)~ area+total_area+mean_depth_v
essel+density+mobility, family = binomial(link = logit), data = datsc)
summary(glm_7.0)
```

```
##
## Call:
## glm(formula = cbind(count, count_total - count) ~ area + total_area +
##     mean_depth_vessel + density + mobility, family = binomial(link = logit
),
##     data = datsc)
##
## Deviance Residuals:
##     Min       1Q   Median       3Q      Max
## -5.8582  -1.2519  -0.2447   0.8501  11.3511
##
## Coefficients:
##                    Estimate Std. Error z value Pr(>|z|)
## (Intercept)        -0.70357    0.07103  -9.905  < 2e-16 ***
## areaCROZET          0.14849    0.10614   1.399   0.1618
## areaFALKLANDS      -3.24792    0.12645 -25.685  < 2e-16 ***
## areaHIMI          -19.86714  330.82816  -0.060   0.9521
## areaKERGUELEN      -3.66179    0.19301 -18.972  < 2e-16 ***
## areaPEMI           -1.64475    0.14791 -11.120  < 2e-16 ***
## areaSOUTH GEORGIA  -1.74703    0.08649 -20.199  < 2e-16 ***
## total_area         -0.80416    0.07000 -11.489  < 2e-16 ***
## mean_depth_vessel  -0.24073    0.02839  -8.479  < 2e-16 ***
## density            -0.31958    0.04059  -7.874 3.43e-15 ***
## mobility           -0.06387    0.03219  -1.984   0.0472 *
## ---
## Signif. codes:  0 '***' 0.001 '**' 0.01 '*' 0.05 '.' 0.1 ' ' 1
##
## (Dispersion parameter for binomial family taken to be 1)
##
##     Null deviance: 10138.7  on 407  degrees of freedom
## Residual deviance:  1565.8  on 397  degrees of freedom
## AIC: 2623.7
##
## Number of Fisher Scoring iterations: 15
```

Calculate pseudo r squared

```
pseudo_r2(glm_7.0)

## [1] 0.8455663
```

Model validation

plots of residuals versus fitted values for check of linearity and homoscedasticity of variance

```
plot(fitted(glm_7.0), resid(glm_7.0), main="Residual Plot for linearity and h
omoscedasticity of variance",xlab="Fitted value",ylab="Residuals")
```

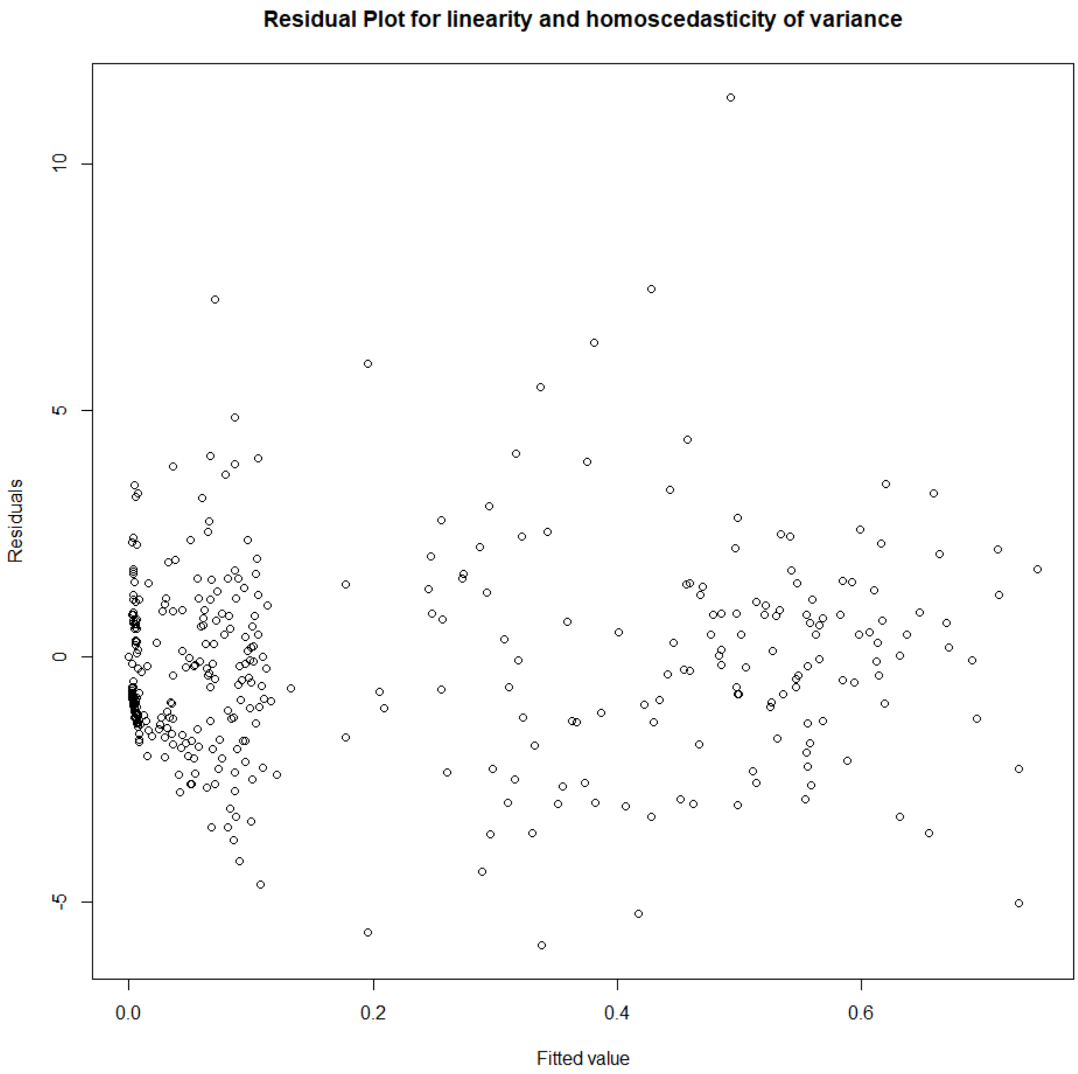

test for normality of the residuals

```
hist(resid(glm_7.0),xlab="Residuals",main="Normality of the Residuals")
```

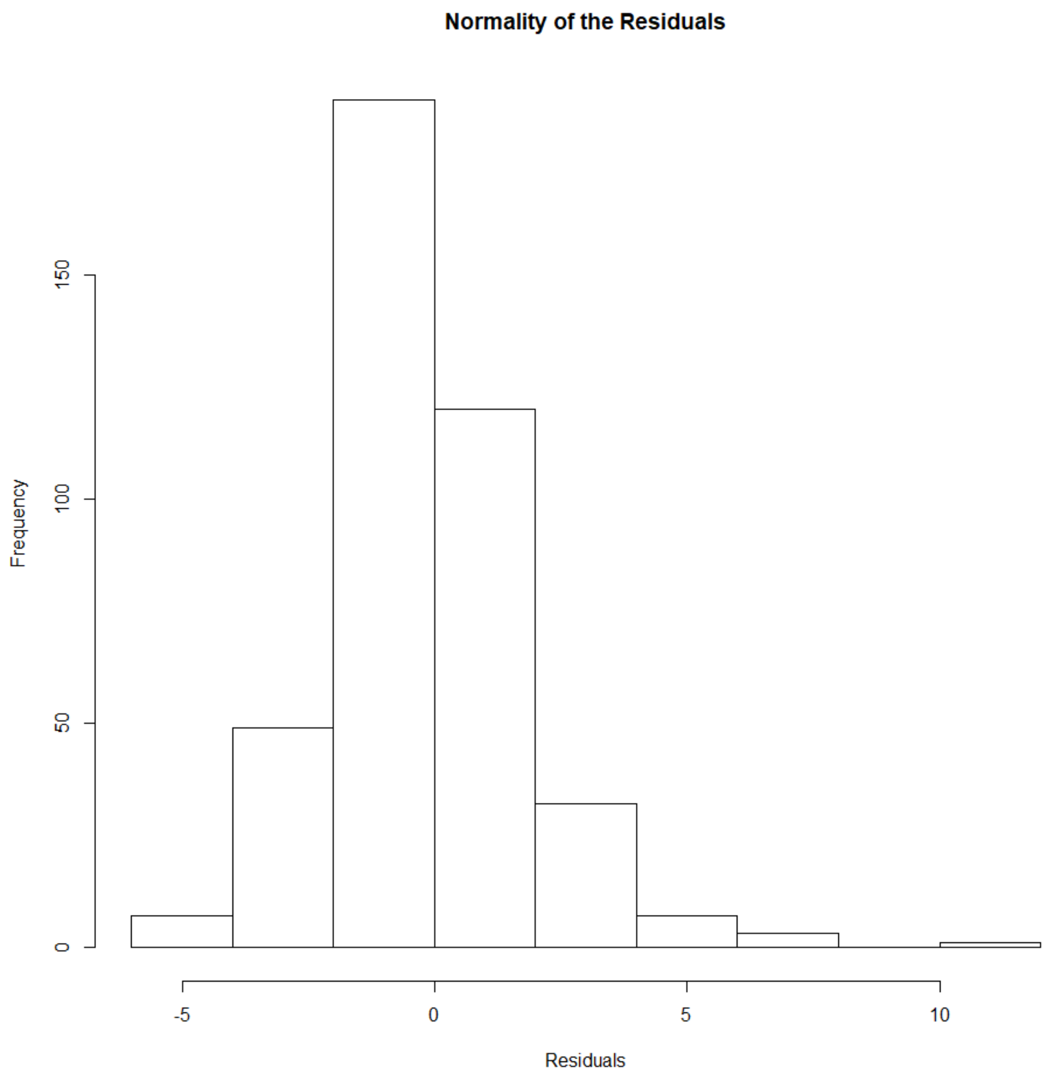

Interaction between Area and mobility

A reviewer requested we investigate including an interaction between fishery and mobility

```
glm_8.0 <- glm(cbind(count,count_total - count)~ area+total_area+mean_depth_v
essel+
                 density+mobility+area:mobility,
               family = binomial(link = logit), data = datsc)
summary(glm_8.0)

##
## Call:
## glm(formula = cbind(count, count_total - count) ~ area + total_area +
##     mean_depth_vessel + density + mobility + area:mobility, family = binom
ial(link = logit),
##     data = datsc)
##
```

```
## Deviance Residuals:
##     Min       1Q   Median       3Q      Max
## -5.6048  -1.2576  -0.2307   0.8490  11.2907
##
## Coefficients:
##                             Estimate Std. Error z value Pr(>|z|)
## (Intercept)                 -0.77910    0.08316  -9.368  < 2e-16 ***
## areaCROZET                   0.23786    0.11075   2.148  0.03174 *
## areaFALKLANDS               -3.16728    0.13624 -23.248  < 2e-16 ***
## areaHIMI                   -19.72223  454.23123  -0.043  0.96537
## areaKERGUELEN               -3.70691    0.22518 -16.462  < 2e-16 ***
## areaPEMI                    -1.52985    0.17923  -8.536  < 2e-16 ***
## areaSOUTH GEORGIA           -1.60065    0.11279 -14.192  < 2e-16 ***
## total_area                  -0.81507    0.07129 -11.433  < 2e-16 ***
## mean_depth_vessel           -0.25482    0.02995  -8.509  < 2e-16 ***
## density                     -0.29106    0.04291  -6.783 1.18e-11 ***
## mobility                    -0.16392    0.06079  -2.697  0.00700 **
## areaCROZET:mobility          0.05757    0.08026   0.717  0.47320
## areaFALKLANDS:mobility       0.43953    0.15156   2.900  0.00373 **
## areaHIMI:mobility            0.20045  706.99569   0.000  0.99977
## areaKERGUELEN:mobility       0.38279    0.21394   1.789  0.07358 .
## areaPEMI:mobility            0.18297    0.19546   0.936  0.34923
## areaSOUTH GEORGIA:mobility   0.22340    0.10232   2.183  0.02902 *
## ---
## Signif. codes:  0 '***' 0.001 '**' 0.01 '*' 0.05 '.' 0.1 ' ' 1
##
## (Dispersion parameter for binomial family taken to be 1)
##
##     Null deviance: 10138.7  on 407  degrees of freedom
## Residual deviance:  1552.4  on 391  degrees of freedom
## AIC: 2622.3
##
## Number of Fisher Scoring iterations: 15
```

Calculate pseudo r squared

```
pseudo_r2(glm_8.0)

## [1] 0.8468868
```

FINAL AIC selection table

Create the AIC selection table including `glm_8.0`, we also calculate all of the pseudo r squared statistics using the extra argument of `model.sel`.

```
(vessel_summary <- model.sel(list(glm_null, glm_1.1,glm_1.2,glm_1.3,glm_1.4,g
lm_1.5,glm_1.6,glm_1.7,glm_2.0,glm_2.1,glm_2.2,glm_2.3,glm_2.4,glm_2.5,glm_2.
6,glm_3.0,glm_3.1,glm_3.2,glm_3.3,glm_3.4,glm_3.5,glm_4.0,glm_4.1,glm_4.2,glm
_4.3,glm_4.4,glm_5.0,glm_5.1,glm_5.2,glm_5.3,glm_6.0,glm_6.1,glm_6.2, glm_8.0
), rank=AIC, extra=pseudo_r2))
```

```
## Model selection table
##      (Int) men_dpt_vss prp_day_trt_cch prp_day_wnt ttl_are     dns are
## 34 -0.7791     -0.2548                                 -0.8151 -0.2911   +
## 28 -0.7036     -0.2407                                 -0.8042 -0.3196   +
## 31 -0.7036     -0.2407                                 -0.8042 -0.3196   +
## 33 -0.7526     -0.2408         0.02165                 -0.8103 -0.3170   +
## 32 -0.6864     -0.2403                     0.02064 -0.8067 -0.3162   +
## 23 -0.6271     -0.2438                                 -0.7979 -0.3143   +
## 27 -0.6271     -0.2438                                 -0.7979 -0.3143   +
## 29 -0.6075     -0.2432                     0.02581 -0.8012 -0.3103   +
## 30 -0.6621     -0.2440         0.01615                 -0.8025 -0.3123   +
## 26 -0.5013     -0.2051                     0.06613 -0.7003           +
## 25 -0.6045     -0.2025                                 -0.6914           +
## 17 -0.5491     -0.2054                                 -0.6883           +
## 22 -0.5491     -0.2054                                 -0.6883           +
## 24 -0.6407     -0.2063         0.04156                 -0.7018           +
## 20 -1.0180                                         -0.8114 -0.2557   +
## 19 -0.9699                                         -0.7221           +
## 21 -0.8513                                 0.06802 -0.7303           +
## 13 -0.9012                                         -0.7182           +
## 16 -0.9012                                         -0.7182           +
## 18 -0.9837                     0.03690                 -0.7300           +
## 10 -0.3722     -0.2200                                               +
## 14 -0.8058                                                 -0.1690   +
## 15 -0.7982                                                           +
## 7  -0.7412                                                           +
## 9  -0.7412                                                           +
## 12 -0.7173                                 0.03102                   +
## 11 -0.7128                    -0.01309                               +
## 5  -2.1790                                         -1.5310
## 3  -2.0120                     0.31790
## 4  -1.9550                                -0.37520
## 6  -1.8860                                                  0.2248
## 2  -1.9470      0.1574
## 8  -1.9370
## 1  -1.9140
##          mbl are:mbl pseudo_r2 df    logLik     AIC   delta weight
## 34 -0.16390       +  0.846900 17 -1294.133  2622.3    0.00  0.335
## 28 -0.06387          0.845600 11 -1300.827  2623.7    1.39  0.167
## 31 -0.06387          0.845600 11 -1300.827  2623.7    1.39  0.167
## 33 -0.06560          0.845600 12 -1300.605  2625.2    2.94  0.077
## 32 -0.06257          0.845600 12 -1300.672  2625.3    3.08  0.072
## 23                   0.845200 10 -1302.799  2625.6    3.33  0.063
## 27                   0.845200 10 -1302.799  2625.6    3.33  0.063
## 29                   0.845200 11 -1302.554  2627.1    4.84  0.030
## 30                   0.845200 11 -1302.673  2627.3    5.08  0.026
## 26                   0.839500 10 -1331.719  2683.4   61.17  0.000
## 25 -0.04675          0.839400 10 -1332.287  2684.6   62.31  0.000
## 17                   0.839100  9 -1333.358  2684.7   62.45  0.000
## 22                   0.839100  9 -1333.358  2684.7   62.45  0.000
```

```
## 24                    0.839300 10 -1332.525  2685.0   62.78  0.000
## 20                    0.837900  9 -1339.889  2697.8   75.51  0.000
## 19 -0.06308           0.834100  9 -1359.096  2736.2  113.93  0.000
## 21                    0.834000  9 -1359.330  2736.7  114.39  0.000
## 13                    0.833700  8 -1361.054  2738.1  115.84  0.000
## 16                    0.833700  8 -1361.054  2738.1  115.84  0.000
## 18                    0.833800  9 -1360.398  2738.8  116.53  0.000
## 10                    0.829200  8 -1383.940  2783.9  161.61  0.000
## 14                    0.824700  8 -1406.694  2829.4  207.12  0.000
## 15 -0.05306           0.823100  8 -1414.865  2845.7  223.46  0.000
## 7                     0.822800  7 -1416.247  2846.5  224.23  0.000
## 9                     0.822800  7 -1416.247  2846.5  224.23  0.000
## 12                    0.822900  8 -1415.881  2847.8  225.50  0.000
## 11                    0.822800  8 -1416.162  2848.3  226.06  0.000
## 5                     0.404900  2 -3534.579  7073.2 4450.89  0.000
## 3                     0.066310  2 -5251.139 10506.3 7884.01  0.000
## 4                     0.039740  2 -5385.854 10775.7 8153.44  0.000
## 6                     0.018600  2 -5493.020 10990.0 8367.77  0.000
## 2                     0.009162  2 -5540.858 11085.7 8463.45  0.000
## 8  -0.13990           0.006428  2 -5554.721 11113.4 8491.18  0.000
## 1                     0.000000  1 -5587.305 11176.6 8554.34  0.000
## Models ranked by AIC(x)
```

Save a copy to file

```
write.csv(vessel_summary, "Killer_whale_vessel_data_AIC_summary.csv")
```

Coefficients of final model

Extract the coefficients of the final model and convert from logit space to natural numbers (i.e. probabilities) and the upper and lower confidence intervals

```
vsl_est <- plogis(summary(glm_8.0)$coefficients[,1]+summary(glm_8.0)$coeffici
ents[1,1])
vsl_lwr <- plogis((summary(glm_8.0)$coefficients[,1]+summary(glm_8.0)$coeffic
ients[1,1]-1.96*summary(glm_8.0)$coefficients[,2]))
vsl_upr <- plogis((summary(glm_8.0)$coefficients[,1]+summary(glm_8.0)$coeffic
ients[1,1]+1.96*summary(glm_8.0)$coefficients[,2]))
vsl_z_stat <- summary(glm_8.0)$coefficients[,3]
vsl_p_value <- summary(glm_8.0)$coefficients[,4]
```

Bind them together

```
(res_vessel <- cbind(vsl_est, vsl_lwr, vsl_upr, vsl_z_stat, vsl_p_value))
```

```
##                                   vsl_est     vsl_lwr    vsl_upr
## (Intercept)                  1.739045e-01 0.151716032 0.19857844
## areaCROZET                   3.678981e-01 0.319012966 0.41965799
## areaFALKLANDS                1.895817e-02 0.014580094 0.02461805
## areaHIMI                     1.248483e-09 0.000000000 1.00000000
## areaKERGUELEN                1.113993e-02 0.007193433 0.01721404
```

```
## areaPEMI                    9.038399e-02 0.065360469 0.12371969
## areaSOUTH GEORGIA           8.472988e-02 0.069086273 0.10352185
## total_area                  1.687978e-01 0.150089423 0.18931875
## mean_depth_vessel           2.623240e-01 0.251124383 0.27384040
## density                     2.553724e-01 0.239711499 0.27169090
## mobility                    2.802909e-01 0.256896888 0.30494112
## areaCROZET:mobility         3.270554e-01 0.293417942 0.36257033
## areaFALKLANDS:mobility      4.159137e-01 0.346009411 0.48937292
## areaHIMI:mobility           3.592433e-01 0.000000000 1.00000000
## areaKERGUELEN:mobility      4.021976e-01 0.306690275 0.50575090
## areaPEMI:mobility           3.552297e-01 0.273042485 0.44694587
## areaSOUTH GEORGIA:mobility 3.645421e-01 0.319458809 0.41213462
##                               vsl_z_stat   vsl_p_value
## (Intercept)                -9.368287e+00  7.371955e-21
## areaCROZET                  2.147727e+00  3.173548e-02
## areaFALKLANDS              -2.324776e+01 1.498641e-119
## areaHIMI                   -4.341893e-02  9.653676e-01
## areaKERGUELEN              -1.646209e+01  6.869022e-61
## areaPEMI                   -8.535771e+00  1.392242e-17
## areaSOUTH GEORGIA          -1.419197e+01  1.027357e-45
## total_area                 -1.143314e+01  2.855782e-30
## mean_depth_vessel          -8.508543e+00  1.761325e-17
## density                    -6.783252e+00  1.175001e-11
## mobility                   -2.696612e+00  7.004873e-03
## areaCROZET:mobility         7.172833e-01  4.731993e-01
## areaFALKLANDS:mobility      2.900061e+00  3.730898e-03
## areaHIMI:mobility           2.835265e-04  9.997738e-01
## areaKERGUELEN:mobility      1.789220e+00  7.357933e-02
## areaPEMI:mobility           9.360887e-01  3.492275e-01
## areaSOUTH GEORGIA:mobility  2.183230e+00  2.901884e-02
```

Create the estimates for Chile (which is zero in binomial regression model)

```
(areaChile <- c(plogis(0+summary(glm_8.0)$coefficients[1,1]),
              plogis(0+summary(glm_8.0)$coefficients[1,1]-1.96*summary(glm_8
.0)$coefficients[1,2]),
              plogis(0+summary(glm_8.0)$coefficients[1,1]+1.96*summary(glm_8
.0)$coefficients[1,2]),
              summary(glm_8.0)$coefficients[1,3], summary(glm_8.0)$coefficie
nts[1,4]))

## [1]  3.145135e-01  2.804758e-01  3.506687e-01 -9.368287e+00  7.371955e-21
```

Replace the intercept with Chilean values

```
res_vessel[1,] <- areaChile
```

Save as a csv

```
write.csv(res_vessel, "Killer_whale_final_model_vessel_est.csv")
```

B Models on *Pr(days)* at the fleet level

B.1. Data preparation

Import the data

```
setwd("C:/Users/LES_research211/Documents/003_ANALYSES/009_SOUTHERN_OCEAN_PAP
ER")
data=read.csv("summary_proportions_predictors_fleet_pemi_a.csv",h=T,sep=",")
str(data)
```

```
## 'data.frame':    912 obs. of  22 variables:
##  $ area                 : Factor w/ 7 levels "CHILE","CROZET",..: 6 6 6 5
5 5 5 5 5 4 ...
##  $ year                 : int  2017 2017 2017 2013 2013 2013 2016 2016 20
16 2011 ...
##  $ depredation          : int  0 0 0 0 0 0 0 0 0 0 ...
##  $ count                : int  366 130 133 2557 253 875 2532 248 838 493
...
##  $ count_total          : int  130 130 130 253 253 253 248 248 248 191 ..
.
##  $ Pr_inter             : num  1 1 1 1 1 1 1 1 1 1 ...
##  $ species              : Factor w/ 2 levels "killer whales",..: 2 2 2 1
1 1 1 1 1 1 ...
##  $ response             : Factor w/ 4 levels "Pr(area)","Pr(days)",..: 3
2 1 3 2 1 3 2 1 3 ...
##  $ area_yr              : Factor w/ 76 levels "CHILE 2006","CHILE 2007",.
.: 62 62 62 56 56 56 59 59 59 40 ...
##  $ total_area           : int  133 133 133 875 875 875 838 838 838 223 ..
.
##  $ length_season        : int  130 130 130 253 253 253 248 248 248 191 ..
.
##  $ mean_nb_vessels      : num  1 1 1 4.38 4.38 ...
##  $ mean_lat             : num  -45.3 -45.3 -45.3 -48.9 -48.9 ...
##  $ mean_depth           : num  1352 1352 1352 1199 1199 ...
##  $ prop_day_winter      : num  0.462 0.462 0.462 0.158 0.158 ...
##  $ prop_day_spring      : num  0 0 0 0.316 0.316 ...
##  $ prop_day_summer      : num  0.0692 0.0692 0.0692 0.2451 0.2451 ...
##  $ prop_day_autumn      : num  0.469 0.469 0.469 0.281 0.281 ...
##  $ prop_day_spanish     : num  0 0 0 0 0 0 0 0 0 0 ...
##  $ prop_day_autoline    : num  0 0 0 1 1 1 1 1 1 1 ...
##  $ prop_day_trotline_cach: num  0.485 0.485 0.485 0 0 ...
##  $ prop_day_trotline    : num  0.515 0.515 0.515 0 0 ...
```

Prepare the data for modelling

```
data$length_season = as.numeric(as.character(data$length_season))
data$total_area=as.numeric(as.character(data$total_area))
data$mobility=data$total_area/data$length_season
data$prop_day_trotline_cach=as.numeric(as.character(data$prop_day_trotline_ca
```

```
ch))
data <-subset(data,!is.na(mean_lat))
data <-subset(data,!is.na(mean_depth))
data$mean_lat=-data$mean_lat
data$density = data$mean_nb_vessels/data$total_area
```

Data selection

```
d1 <- droplevels(data[data$depredation==1 & data$species %in% "killer whales"
& data$response %in% "Pr(days)",
        c("area","year","count","count_total", "Pr_inter", "total_area","mean
_nb_vessels", "mean_lat", "mean_depth","mobility","prop_day_trotline_cach", "
prop_day_winter","density" )])
str(d1)
```

```
## 'data.frame':    74 obs. of  13 variables:
##  $ area                  : Factor w/ 7 levels "CHILE","CROZET",..: 1 2 2 1
2 2 2 2 2 2 ...
##  $ year                  : int  2006 2009 2004 2007 2012 2014 2010 2015 20
07 2006 ...
##  $ count                 : int  64 162 108 128 100 96 90 130 92 109 ...
##  $ count_total           : int  79 206 139 167 136 133 125 182 131 158 ...
##  $ Pr_inter              : num  0.81 0.786 0.777 0.766 0.735 ...
##  $ total_area            : num  141 237 159 173 192 201 216 246 201 204 ..
.
##  $ mean_nb_vessels       : num  2.45 2.35 1.39 1.59 2.21 ...
##  $ mean_lat              : num  55.7 46.1 46.1 55.4 46.2 ...
##  $ mean_depth            : num  1526 1091 1094 1593 1031 ...
##  $ mobility              : num  1.78 1.15 1.14 1.04 1.41 ...
##  $ prop_day_trotline_cach: num  0.848 0 0 0.749 0 ...
##  $ prop_day_winter       : num  0 0.311 0.353 0.018 0.11 ...
##  $ density               : num  0.01734 0.0099 0.00873 0.00917 0.01149 ...
```

Data exploration

```
hist(d1$mean_depth)
```

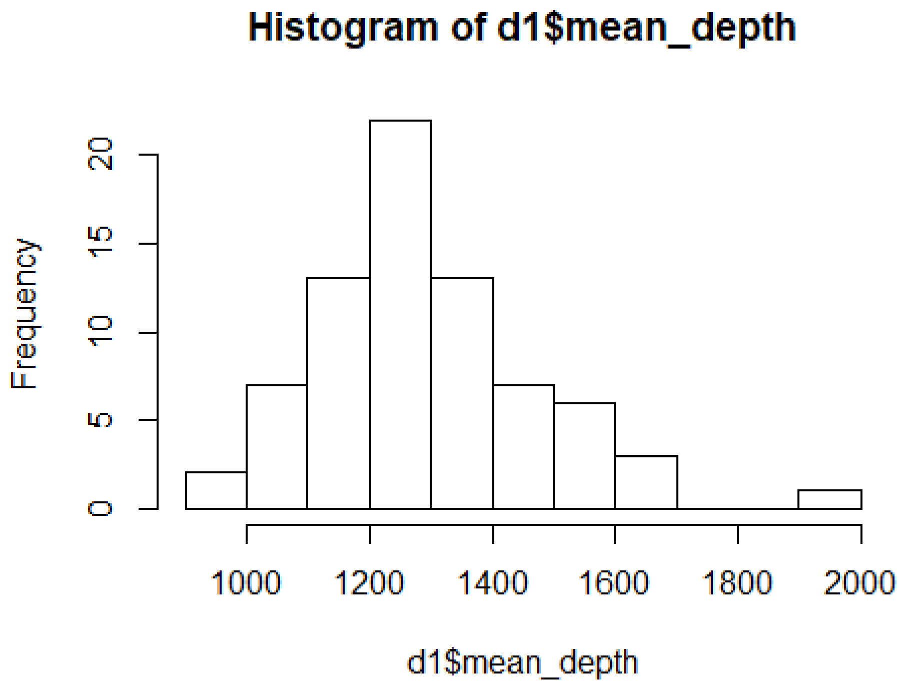


```
hist(d1$total_area)
```

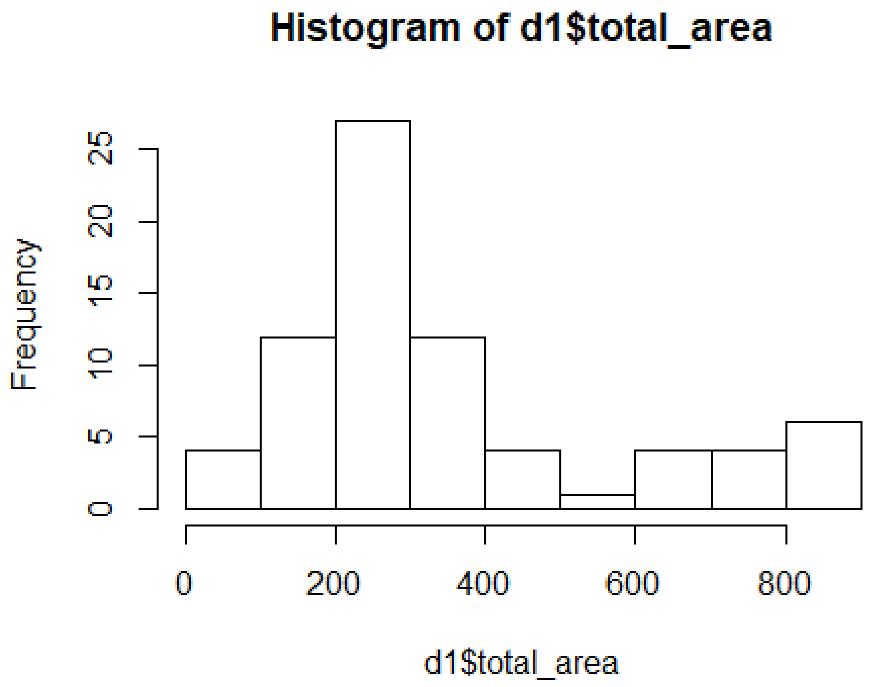


```
hist(d1$mobility)
```

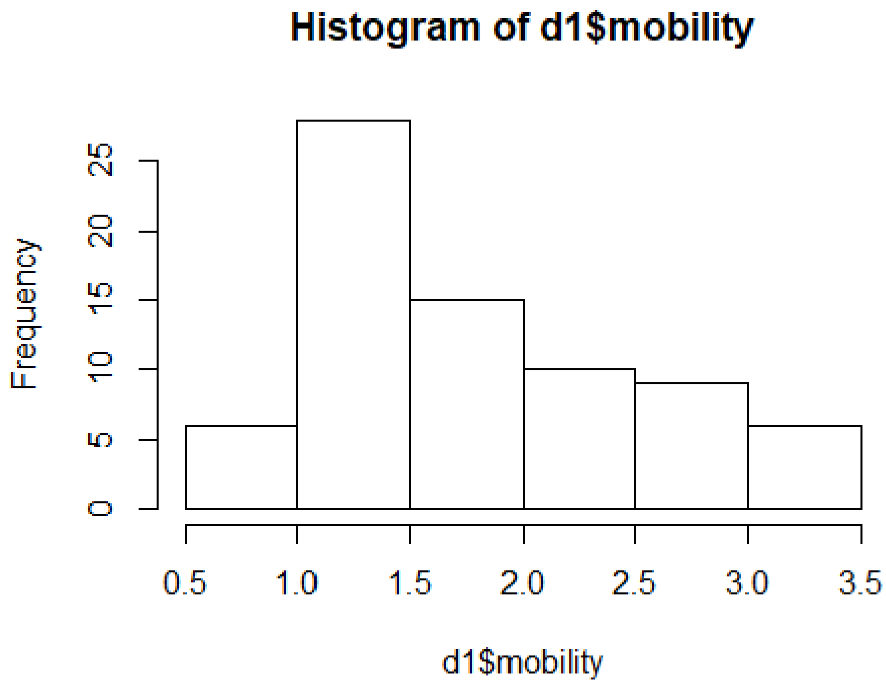


```
# exclude extreme high value
#d1 = subset(d1, d1$density<=0.035)

hist(d1$density)
```

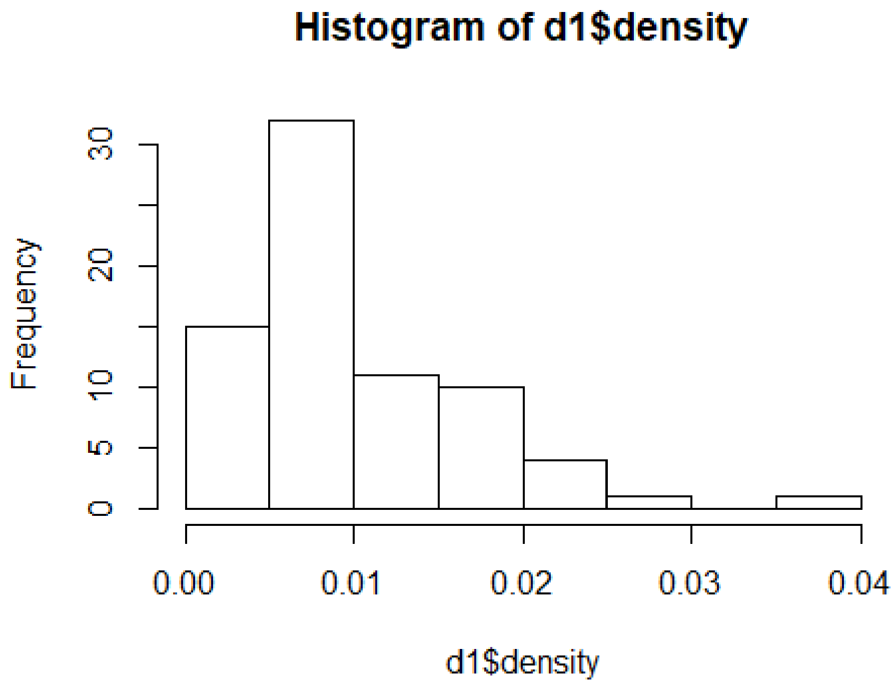


```
# exclude extreme high value
d1 = subset(d1, d1$density<=0.03)
```

Exploratory plots

```
p1 <- ggplot(d1,aes(total_area,Pr_inter,colour=area))+geom_point()+ggtitle("s
ize fishing area")
p2 <- ggplot(d1,aes(mean_depth,Pr_inter,colour=area))+geom_point()+ggtitle("d
epth")
p3 <- ggplot(d1,aes(density,Pr_inter,colour=area))+geom_point()+ggtitle("dens
ity")
p4 <- ggplot(d1,aes(prop_day_winter,Pr_inter,colour=area))+geom_point()+ggtit
le("effort in winter")
p5 <- ggplot(d1,aes(mobility,Pr_inter,colour=area))+geom_point()+ggtitle("mob
ility")
p6 <- ggplot(d1,aes(count_total,Pr_inter,colour=area))+geom_point()+ggtitle("
effort")


ggarrange(p1, p2,p3,p4,p5, common.legend=TRUE, legend = "right")
```

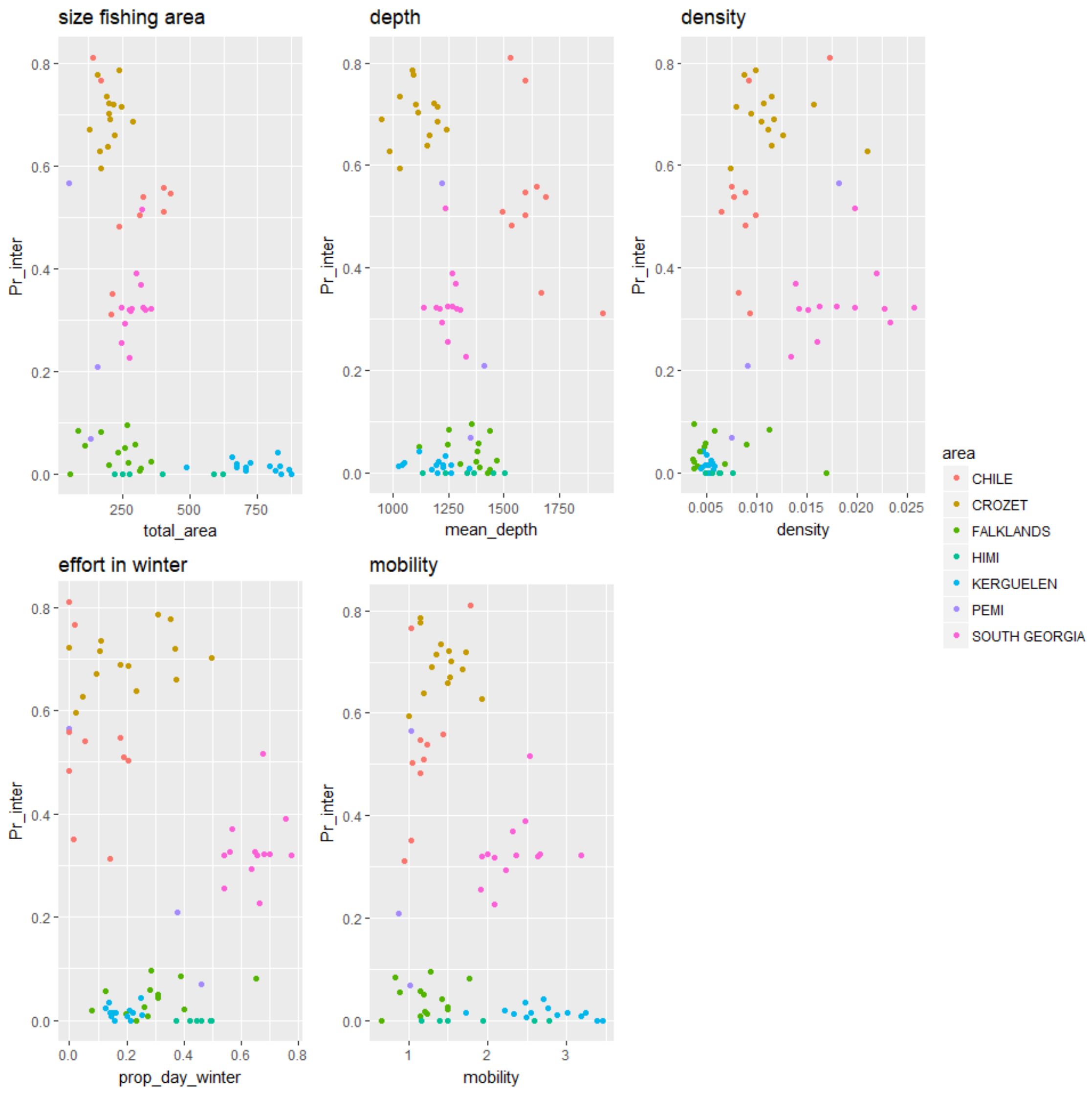

Pairwise plots

```
d1_explo <-droplevels(d1[,
        c(   "mean_depth","total_area","density", "count_total","mobility","pr
op_day_winter","prop_day_trotline_cach")])
pairs(d1_explo)
```

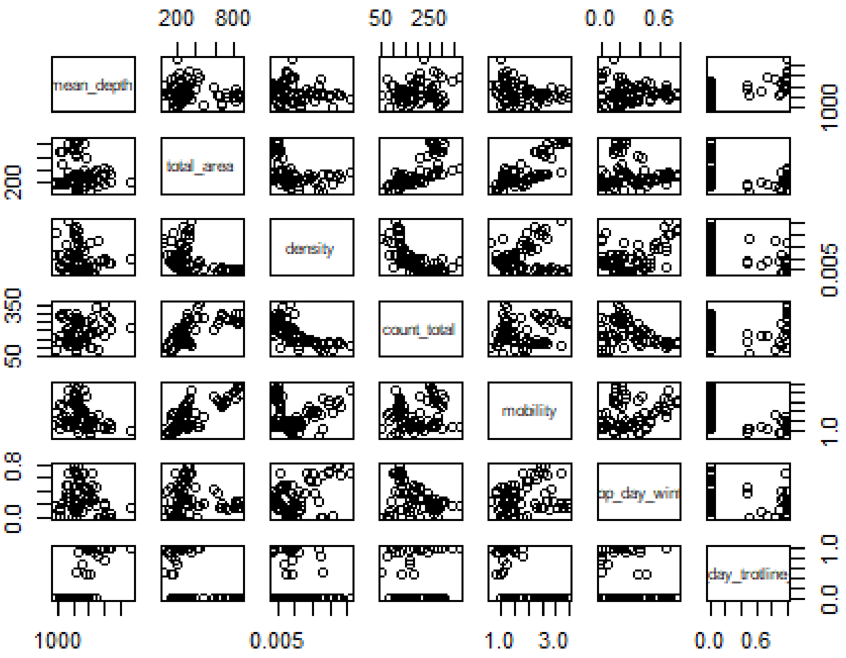

Pearson correlation

Calculate Pearson correlation coefficients for the above data

```
print(cor(d1_explo, method=c("pearson")), digits=4)

##                          mean_depth total_area  density count_total mobility
## mean_depth                   1.0000    -0.1568 -0.16876     0.20580 -0.32776
## total_area                  -0.1568     1.0000 -0.43097     0.70497  0.78957
## density                     -0.1688    -0.4310  1.00000    -0.69281  0.09026
## count_total                  0.2058     0.7050 -0.69281     1.00000  0.18197
## mobility                    -0.3278     0.7896  0.09026     0.18197  1.00000
## prop_day_winter             -0.1407    -0.1302  0.46705    -0.37471  0.27328
## prop_day_trotline_cach       0.7037    -0.3335 -0.14706     0.05914 -0.53103
##                          prop_day_winter prop_day_trotline_cach
## mean_depth                       -0.1407                0.70365
## total_area                       -0.1302               -0.33346
## density                           0.4671               -0.14706
## count_total                      -0.3747                0.05914
## mobility                          0.2733               -0.53103
## prop_day_winter                   1.0000               -0.31019
## prop_day_trotline_cach           -0.3102                1.00000
```

Re-scale continuous predictors

```
pvars <- c("total_area", "mean_depth","mobility","prop_day_trotline_cach", "p
rop_day_winter","density" )
```

```r
datsc <- d1
datsc[pvars] <- lapply(datsc[pvars],scale)
```

B.2. Model fitting

We fit a glm to the proportion of days with killer whale interaction using a binomial fmaily and a logit link function. The most parcimonious model is selected using a stepwise forward AIC selection

Null model

```r
glm_null <- glm(cbind(count,count_total - count)~ 1, family = binomial(link = logit), data = datsc)
summary(glm_null)
```

```
##
## Call:
## glm(formula = cbind(count, count_total - count) ~ 1, family = binomial(lin
k = logit),
##     data = datsc)
##
## Deviance Residuals:
##      Min        1Q    Median        3Q       Max
## -12.0568  -10.2755    0.1979    9.4833   16.2123
##
## Coefficients:
##             Estimate Std. Error z value Pr(>|z|)
## (Intercept) -1.10791    0.01961   -56.5   <2e-16 ***
## ---
## Signif. codes:  0 '***' 0.001 '**' 0.01 '*' 0.05 '.' 0.1 ' ' 1
##
## (Dispersion parameter for binomial family taken to be 1)
##
##     Null deviance: 6196  on 72  degrees of freedom
## Residual deviance: 6196  on 72  degrees of freedom
## AIC: 6493.8
##
## Number of Fisher Scoring iterations: 5
```

Calculate pseudo r squared for the Null model as a check

```r
pseudo_r2(glm_null)
```

```
## [1] 0
```

Single term models

Model with total area size

```r
glm_1.1 <- glm(cbind(count,count_total - count)~ total_area, family = binomia
l(link = logit), data = datsc)
summary(glm_1.1)
```

```
##
## Call:
## glm(formula = cbind(count, count_total - count) ~ total_area,
##     family = binomial(link = logit), data = datsc)
##
## Deviance Residuals:
##     Min       1Q   Median       3Q      Max
## -13.506   -7.536   -1.244    5.897   15.929
##
## Coefficients:
##             Estimate Std. Error z value Pr(>|z|)
## (Intercept) -1.14192    0.02260  -50.53   <2e-16 ***
## total_area  -1.12158    0.03165  -35.43   <2e-16 ***
## ---
## Signif. codes:  0 '***' 0.001 '**' 0.01 '*' 0.05 '.' 0.1 ' ' 1
##
## (Dispersion parameter for binomial family taken to be 1)
##
##     Null deviance: 6196.0  on 72  degrees of freedom
## Residual deviance: 4178.3  on 71  degrees of freedom
## AIC: 4478.1
##
## Number of Fisher Scoring iterations: 5
```

Calculate pseudo r squared

```
pseudo_r2(glm_1.1)

## [1] 0.3256453
```

Model with depth

```
glm_1.2 <- glm(cbind(count,count_total - count)~ mean_depth, family = binomia
l(link = logit), data = datsc)
summary(glm_1.2)

##
## Call:
## glm(formula = cbind(count, count_total - count) ~ mean_depth,
##     family = binomial(link = logit), data = datsc)
##
## Deviance Residuals:
##      Min        1Q    Median        3Q       Max
## -12.2611  -10.0629   -0.6332    8.4314   17.4021
##
## Coefficients:
##             Estimate Std. Error z value Pr(>|z|)
## (Intercept) -1.12489    0.01986 -56.648  < 2e-16 ***
## mean_depth   0.14567    0.01827   7.975 1.52e-15 ***
## ---
## Signif. codes:  0 '***' 0.001 '**' 0.01 '*' 0.05 '.' 0.1 ' ' 1
```

```
##
## (Dispersion parameter for binomial family taken to be 1)
##
##     Null deviance: 6196.0  on 72  degrees of freedom
## Residual deviance: 6133.1  on 71  degrees of freedom
## AIC: 6432.9
##
## Number of Fisher Scoring iterations: 5
```

Calculate pseudo r squared

```
pseudo_r2(glm_1.2)

## [1] 0.01015295
```

Model with mobility

```
glm_1.3 <- glm(cbind(count,count_total - count)~ mobility, family = binomial(
link = logit), data = datsc)
summary(glm_1.3)

##
## Call:
## glm(formula = cbind(count, count_total - count) ~ mobility, family = binom
ial(link = logit),
##     data = datsc)
##
## Deviance Residuals:
##     Min       1Q   Median       3Q      Max
## -15.203   -8.153   -1.343    7.266   12.236
##
## Coefficients:
##             Estimate Std. Error z value Pr(>|z|)
## (Intercept) -1.20975    0.02189  -55.25   <2e-16 ***
## mobility    -0.75849    0.02425  -31.28   <2e-16 ***
## ---
## Signif. codes:  0 '***' 0.001 '**' 0.01 '*' 0.05 '.' 0.1 ' ' 1
##
## (Dispersion parameter for binomial family taken to be 1)
##
##     Null deviance: 6196.0  on 72  degrees of freedom
## Residual deviance: 4981.5  on 71  degrees of freedom
## AIC: 5281.2
##
## Number of Fisher Scoring iterations: 5
```

Calculate pseudo r squared

```
pseudo_r2(glm_1.3)

## [1] 0.1960248
```

Model with proportion of sets using trotlines-cachalotera

```
glm_1.4 <- glm(cbind(count,count_total - count)~ prop_day_trotline_cach, fami
ly = binomial(link = logit), data = datsc)
summary(glm_1.4)
```

```
##
## Call:
## glm(formula = cbind(count, count_total - count) ~ prop_day_trotline_cach,
##     family = binomial(link = logit), data = datsc)
##
## Deviance Residuals:
##      Min        1Q    Median        3Q       Max
## -12.2635   -9.4959   -0.0483    7.0371   17.7135
##
## Coefficients:
##                        Estimate Std. Error z value Pr(>|z|)
## (Intercept)            -1.13786    0.02006  -56.73   <2e-16 ***
## prop_day_trotline_cach  0.30207    0.01829   16.52   <2e-16 ***
## ---
## Signif. codes:  0 '***' 0.001 '**' 0.01 '*' 0.05 '.' 0.1 ' ' 1
##
## (Dispersion parameter for binomial family taken to be 1)
##
##     Null deviance: 6196.0  on 72  degrees of freedom
## Residual deviance: 5929.2  on 71  degrees of freedom
## AIC: 6228.9
##
## Number of Fisher Scoring iterations: 5
```

Calculate pseudo r squared

```
pseudo_r2(glm_1.4)
```

```
## [1] 0.04307031
```

Model with proportion of days in winter

```
glm_1.5 <- glm(cbind(count,count_total - count)~ prop_day_winter, family = bi
nomial(link = logit), data = datsc)
summary(glm_1.5)
```

```
##
## Call:
## glm(formula = cbind(count, count_total - count) ~ prop_day_winter,
##     family = binomial(link = logit), data = datsc)
##
## Deviance Residuals:
##     Min       1Q   Median       3Q      Max
## -12.687   -9.848    1.502    8.380   16.582
##
## Coefficients:
```

```
##                  Estimate Std. Error z value Pr(>|z|)
## (Intercept)     -1.15092    0.02042 -56.376   <2e-16 ***
## prop_day_winter -0.23308    0.02371  -9.831   <2e-16 ***
## ---
## Signif. codes:  0 '***' 0.001 '**' 0.01 '*' 0.05 '.' 0.1 ' ' 1
##
## (Dispersion parameter for binomial family taken to be 1)
##
##     Null deviance: 6196.0  on 72  degrees of freedom
## Residual deviance: 6095.3  on 71  degrees of freedom
## AIC: 6395.1
##
## Number of Fisher Scoring iterations: 5
```

Calculate pseudo r squared

```
pseudo_r2(glm_1.5)

## [1] 0.01625031
```

Model with density of vessels

```
glm_1.6 <- glm(cbind(count,count_total - count)~ density, family = binomial(l
ink = logit), data = datsc)
summary(glm_1.6)

##
## Call:
## glm(formula = cbind(count, count_total - count) ~ density, family = binomi
al(link = logit),
##     data = datsc)
##
## Deviance Residuals:
##     Min       1Q   Median       3Q      Max
## -11.231   -8.076   -4.767    6.847   15.361
##
## Coefficients:
##             Estimate Std. Error z value Pr(>|z|)
## (Intercept) -0.99223    0.02082  -47.65   <2e-16 ***
## density      0.79638    0.02271   35.07   <2e-16 ***
## ---
## Signif. codes:  0 '***' 0.001 '**' 0.01 '*' 0.05 '.' 0.1 ' ' 1
##
## (Dispersion parameter for binomial family taken to be 1)
##
##     Null deviance: 6196.0  on 72  degrees of freedom
## Residual deviance: 4859.4  on 71  degrees of freedom
## AIC: 5159.2
##
## Number of Fisher Scoring iterations: 5
```

Calculate pseudo r squared

```
pseudo_r2(glm_1.6)

## [1] 0.2157165
```

Model with the fishery

```
glm_1.7 <- glm(cbind(count,count_total - count)~ area, family = binomial(link
= logit), data = datsc)
summary(glm_1.7)

##
## Call:
## glm(formula = cbind(count, count_total - count) ~ area, family = binomial(
link = logit),
##     data = datsc)
##
## Deviance Residuals:
##     Min       1Q   Median       3Q      Max
## -6.1653  -0.9885  -0.1395   0.8544   6.6311
##
## Coefficients:
##                     Estimate Std. Error z value Pr(>|z|)
## (Intercept)          0.06979    0.04054   1.721   0.0852 .
## areaCROZET           0.76630    0.06304  12.156   <2e-16 ***
## areaFALKLANDS       -3.25265    0.11228 -28.970   <2e-16 ***
## areaHIMI           -21.07363  633.65054  -0.033   0.9735
## areaKERGUELEN       -4.19903    0.13528 -31.040   <2e-16 ***
## areaPEMI            -1.38546    0.13459 -10.294   <2e-16 ***
## areaSOUTH GEORGIA   -0.77294    0.06616 -11.684   <2e-16 ***
## ---
## Signif. codes:  0 '***' 0.001 '**' 0.01 '*' 0.05 '.' 0.1 ' ' 1
##
## (Dispersion parameter for binomial family taken to be 1)
##
##     Null deviance: 6196.03  on 72  degrees of freedom
## Residual deviance:  342.46  on 66  degrees of freedom
## AIC: 652.22
##
## Number of Fisher Scoring iterations: 15
```

Calculate pseudo r squared

```
pseudo_r2(glm_1.7)

## [1] 0.9447298
```

AIC summary

```
model.sel(list(glm_null, glm_1.1,glm_1.2,glm_1.3,glm_1.4,glm_1.5,glm_1.6,glm_
1.7))
```

```
## Model selection table
##      (Int) ttl_are men_dpt     mbl prp_day_trt_cch prp_day_wnt    dns are
## 8  0.06979                                                              +
## 2 -1.14200  -1.122
## 7 -0.99220                                                    0.7964
## 4 -1.21000                 -0.7585
## 5 -1.13800                                 0.3021
## 6 -1.15100                                            -0.2331
## 3 -1.12500          0.1457
## 1 -1.10800
##   df    logLik   AICc   delta weight
## 8  7  -319.109  653.9    0.00      1
## 2  2 -2237.042 4478.3 3824.31      0
## 7  2 -2577.603 5159.4 4505.44      0
## 4  2 -2638.608 5281.4 4627.45      0
## 5  2 -3112.464 6229.1 5575.16      0
## 6  2 -3195.552 6395.3 5741.33      0
## 3  2 -3214.442 6433.1 5779.11      0
## 1  1 -3245.896 6493.8 5839.91      0
## Models ranked by AICc(x)
```

Model with lowest AIC

```
glm_2.0 <- glm(cbind(count,count_total - count)~ area, family = binomial(link
= logit), data = datsc)
summary(glm_2.0)
```

```
##
## Call:
## glm(formula = cbind(count, count_total - count) ~ area, family = binomial(
link = logit),
##     data = datsc)
##
## Deviance Residuals:
##     Min       1Q   Median       3Q      Max
## -6.1653  -0.9885  -0.1395   0.8544   6.6311
##
## Coefficients:
##                     Estimate Std. Error z value Pr(>|z|)
## (Intercept)          0.06979    0.04054   1.721   0.0852 .
## areaCROZET           0.76630    0.06304  12.156   <2e-16 ***
## areaFALKLANDS       -3.25265    0.11228 -28.970   <2e-16 ***
## areaHIMI           -21.07363  633.65054  -0.033   0.9735
## areaKERGUELEN       -4.19903    0.13528 -31.040   <2e-16 ***
## areaPEMI            -1.38546    0.13459 -10.294   <2e-16 ***
## areaSOUTH GEORGIA  -0.77294    0.06616 -11.684   <2e-16 ***
## ---
## Signif. codes:  0 '***' 0.001 '**' 0.01 '*' 0.05 '.' 0.1 ' ' 1
##
## (Dispersion parameter for binomial family taken to be 1)
```

```
##
##     Null deviance: 6196.03  on 72  degrees of freedom
## Residual deviance:  342.46  on 66  degrees of freedom
## AIC: 652.22
##
## Number of Fisher Scoring iterations: 15
```

Calculate pseudo r squared

```
pseudo_r2(glm_2.0)

## [1] 0.9447298
```

Two terms models

Model with total area size

```
glm_2.1 <- glm(cbind(count,count_total - count)~ area+ total_area, family = b
inomial(link = logit), data = datsc)
summary(glm_2.1)

##
## Call:
## glm(formula = cbind(count, count_total - count) ~ area + total_area,
##     family = binomial(link = logit), data = datsc)
##
## Deviance Residuals:
##     Min       1Q   Median       3Q      Max
## -6.1909  -1.0073  -0.1340   0.8434   6.6014
##
## Coefficients:
##                     Estimate Std. Error z value Pr(>|z|)
## (Intercept)         0.068630   0.042477   1.616    0.106
## areaCROZET          0.762719   0.074283  10.268   <2e-16 ***
## areaFALKLANDS      -3.254674   0.114454 -28.436   <2e-16 ***
## areaHIMI          -21.064027 631.561375  -0.033    0.973
## areaKERGUELEN      -4.184858   0.206132 -20.302   <2e-16 ***
## areaPEMI           -1.391475   0.149898  -9.283   <2e-16 ***
## areaSOUTH GEORGIA  -0.773649   0.066615 -11.614   <2e-16 ***
## total_area         -0.007301   0.080138  -0.091    0.927
## ---
## Signif. codes:  0 '***' 0.001 '**' 0.01 '*' 0.05 '.' 0.1 ' ' 1
##
## (Dispersion parameter for binomial family taken to be 1)
##
##     Null deviance: 6196.03  on 72  degrees of freedom
## Residual deviance:  342.45  on 65  degrees of freedom
## AIC: 654.21
##
## Number of Fisher Scoring iterations: 15
```

Calculate pseudo r squared

```
pseudo_r2(glm_2.1)

## [1] 0.9447312
```

Model with depth

```
glm_2.2 <- glm(cbind(count,count_total - count)~ area+ mean_depth, family = b
inomial(link = logit), data = datsc)
summary(glm_2.2)

##
## Call:
## glm(formula = cbind(count, count_total - count) ~ area + mean_depth,
##     family = binomial(link = logit), data = datsc)
##
## Deviance Residuals:
##     Min       1Q   Median       3Q      Max
## -4.5347  -1.2036  -0.0003   0.9819   6.2610
##
## Coefficients:
##                      Estimate Std. Error z value Pr(>|z|)
## (Intercept)           0.66851    0.10071   6.638 3.19e-11 ***
## areaCROZET           -0.14926    0.15391  -0.970    0.332
## areaFALKLANDS        -3.74960    0.13721 -27.327  < 2e-16 ***
## areaHIMI            -22.59420 1031.90673  -0.022    0.983
## areaKERGUELEN        -5.00710    0.18518 -27.040  < 2e-16 ***
## areaPEMI             -1.86152    0.15364 -12.116  < 2e-16 ***
## areaSOUTH GEORGIA    -1.44190    0.12260 -11.761  < 2e-16 ***
## mean_depth           -0.32889    0.05072  -6.484 8.91e-11 ***
## ---
## Signif. codes:  0 '***' 0.001 '**' 0.01 '*' 0.05 '.' 0.1 ' ' 1
##
## (Dispersion parameter for binomial family taken to be 1)
##
##     Null deviance: 6196.03  on 72  degrees of freedom
## Residual deviance:  299.88  on 65  degrees of freedom
## AIC: 611.64
##
## Number of Fisher Scoring iterations: 16
```

Calculate pseudo r squared

```
pseudo_r2(glm_2.2)

## [1] 0.951601
```

Model with mobility

```r
glm_2.3 <- glm(cbind(count,count_total - count)~ area+ mobility, family = bin
omial(link = logit), data = datsc)
summary(glm_2.3)
```

```
##
## Call:
## glm(formula = cbind(count, count_total - count) ~ area + mobility,
##     family = binomial(link = logit), data = datsc)
##
## Deviance Residuals:
##     Min       1Q   Median       3Q      Max
## -5.6665  -1.1796  -0.0007   0.8480   6.8974
##
## Coefficients:
##                     Estimate Std. Error z value Pr(>|z|)
## (Intercept)          0.26460    0.07363   3.594 0.000326 ***
## areaCROZET           0.69875    0.06647  10.513  < 2e-16 ***
## areaFALKLANDS       -3.27613    0.11259 -29.098  < 2e-16 ***
## areaHIMI           -21.29767  621.90805  -0.034 0.972681
## areaKERGUELEN       -4.69548    0.20932 -22.432  < 2e-16 ***
## areaPEMI            -1.31645    0.13634  -9.656  < 2e-16 ***
## areaSOUTH GEORGIA   -1.13616    0.13280  -8.556  < 2e-16 ***
## mobility             0.22660    0.07144   3.172 0.001515 **
## ---
## Signif. codes:  0 '***' 0.001 '**' 0.01 '*' 0.05 '.' 0.1 ' ' 1
##
## (Dispersion parameter for binomial family taken to be 1)
##
##     Null deviance: 6196.03  on 72  degrees of freedom
## Residual deviance:  332.38  on 65  degrees of freedom
## AIC: 644.14
##
## Number of Fisher Scoring iterations: 15
```

Calculate pseudo r squared

```r
pseudo_r2(glm_2.3)
```

```
## [1] 0.9463558
```

Model with proportion of sets using trotlines-cachalotera

```r
glm_2.4 <- glm(cbind(count,count_total - count)~ area+ prop_day_trotline_cach
, family = binomial(link = logit), data = datsc)
summary(glm_2.4)
```

```
##
## Call:
## glm(formula = cbind(count, count_total - count) ~ area + prop_day_trotline
_cach,
##     family = binomial(link = logit), data = datsc)
```

```
##
## Deviance Residuals:
##     Min       1Q   Median       3Q      Max
## -6.1358  -0.9971  -0.1395   0.8585   6.4157
##
## Coefficients:
##                         Estimate Std. Error z value Pr(>|z|)
## (Intercept)              0.17794    0.15509   1.147  0.25126
## areaCROZET               0.61771    0.21513   2.871  0.00409 **
## areaFALKLANDS           -3.32212    0.14993 -22.158  < 2e-16 ***
## areaHIMI               -21.22223  633.65057  -0.033  0.97328
## areaKERGUELEN           -4.34763    0.24618 -17.660  < 2e-16 ***
## areaPEMI                -1.46027    0.16982  -8.599  < 2e-16 ***
## areaSOUTH GEORGIA       -0.92153    0.21606  -4.265    2e-05 ***
## prop_day_trotline_cach  -0.06377    0.08827  -0.722  0.47001
## ---
## Signif. codes:  0 '***' 0.001 '**' 0.01 '*' 0.05 '.' 0.1 ' ' 1
##
## (Dispersion parameter for binomial family taken to be 1)
##
##     Null deviance: 6196.03  on 72  degrees of freedom
## Residual deviance:  341.94  on 65  degrees of freedom
## AIC: 653.7
##
## Number of Fisher Scoring iterations: 15
```

Calculate pseudo r squared

```
pseudo_r2(glm_2.4)

## [1] 0.9448138
```

Model with proportion of days in winter

```
glm_2.5 <- glm(cbind(count,count_total - count)~ area+ prop_day_winter, famil
y = binomial(link = logit), data = datsc)
summary(glm_2.5)

##
## Call:
## glm(formula = cbind(count, count_total - count) ~ area + prop_day_winter,
##     family = binomial(link = logit), data = datsc)
##
## Deviance Residuals:
##     Min       1Q   Median       3Q      Max
## -6.1181  -1.1031  -0.1316   0.7982   6.5487
##
## Coefficients:
##                    Estimate Std. Error z value Pr(>|z|)
## (Intercept)         0.03776    0.06241   0.605    0.545
## areaCROZET          0.78427    0.06849  11.450  < 2e-16 ***
```

```
## areaFALKLANDS      -3.22424    0.11983 -26.906  < 2e-16 ***
## areaHIMI          -21.01848  633.56659  -0.033    0.974
## areaKERGUELEN      -4.18604    0.13664 -30.636  < 2e-16 ***
## areaPEMI           -1.34541    0.14700  -9.153  < 2e-16 ***
## areaSOUTH GEORGIA  -0.68600    0.14477  -4.739 2.15e-06 ***
## prop_day_winter    -0.03441    0.05099  -0.675    0.500
## ---
## Signif. codes:  0 '***' 0.001 '**' 0.01 '*' 0.05 '.' 0.1 ' ' 1
##
## (Dispersion parameter for binomial family taken to be 1)
##
##     Null deviance: 6196  on 72  degrees of freedom
## Residual deviance:  342  on 65  degrees of freedom
## AIC: 653.76
##
## Number of Fisher Scoring iterations: 15
```

Calculate pseudo r squared

```
pseudo_r2(glm_2.5)

## [1] 0.9448033
```

Model with density of vessels

```
glm_2.6 <- glm(cbind(count,count_total - count)~ area+ density, family = bino
mial(link = logit), data = datsc)
summary(glm_2.6)

##
## Call:
## glm(formula = cbind(count, count_total - count) ~ area + density,
##     family = binomial(link = logit), data = datsc)
##
## Deviance Residuals:
##     Min       1Q   Median       3Q      Max
## -6.3318  -1.1896  -0.0006   0.8376   6.5198
##
## Coefficients:
##                    Estimate Std. Error z value Pr(>|z|)
## (Intercept)         0.11052    0.04191   2.637 0.008366 **
## areaCROZET          0.69009    0.06594  10.465  < 2e-16 ***
## areaFALKLANDS      -3.14725    0.11522 -27.315  < 2e-16 ***
## areaHIMI          -20.98850  634.18430  -0.033 0.973599
## areaKERGUELEN      -4.07770    0.13884 -29.369  < 2e-16 ***
## areaPEMI           -1.43102    0.13537 -10.571  < 2e-16 ***
## areaSOUTH GEORGIA  -1.10414    0.10875 -10.153  < 2e-16 ***
## density             0.19139    0.04938   3.876 0.000106 ***
## ---
## Signif. codes:  0 '***' 0.001 '**' 0.01 '*' 0.05 '.' 0.1 ' ' 1
##
```

```
## (Dispersion parameter for binomial family taken to be 1)
##
##     Null deviance: 6196.03  on 72  degrees of freedom
## Residual deviance:  327.42  on 65  degrees of freedom
## AIC: 639.19
##
## Number of Fisher Scoring iterations: 15
```

Calculate pseudo r squared

```
pseudo_r2(glm_2.6)

## [1] 0.9471558
```

AIC summary

```
model.sel(list(glm_2.0,glm_2.1,glm_2.2,glm_2.3,glm_2.4,glm_2.5,glm_2.6))

## Model selection table
##     (Int) are   ttl_are men_dpt    mbl prp_day_trt_cch prp_day_wnt    dns
## 3 0.66850   +           -0.3289
## 7 0.11050   +                                                      0.1914
## 4 0.26460   +                   0.2266
## 1 0.06979   +
## 5 0.17790   +                                -0.06377
## 6 0.03776   +                                            -0.03441
## 2 0.06863   + -0.007301
##   df   logLik  AICc delta weight
## 3  8 -297.822 613.9  0.00      1
## 7  8 -311.594 641.4 27.54      0
## 4  8 -314.072 646.4 32.50      0
## 1  7 -319.109 653.9 40.05      0
## 5  8 -318.849 655.9 42.05      0
## 6  8 -318.882 656.0 42.12      0
## 2  8 -319.105 656.5 42.57      0
## Models ranked by AICc(x)
```

Model with lowest AIC

```
glm_3.0 <- glm(cbind(count,count_total - count)~ area+ mean_depth, family = b
inomial(link = logit), data = datsc)
summary(glm_3.0)

##
## Call:
## glm(formula = cbind(count, count_total - count) ~ area + mean_depth,
##     family = binomial(link = logit), data = datsc)
##
## Deviance Residuals:
##     Min       1Q   Median       3Q      Max
## -4.5347  -1.2036  -0.0003   0.9819   6.2610
##
```

```
## Coefficients:
##                     Estimate Std. Error z value Pr(>|z|)    
## (Intercept)          0.66851    0.10071   6.638 3.19e-11 ***
## areaCROZET          -0.14926    0.15391  -0.970    0.332    
## areaFALKLANDS       -3.74960    0.13721 -27.327  < 2e-16 ***
## areaHIMI           -22.59420 1031.90673  -0.022    0.983    
## areaKERGUELEN       -5.00710    0.18518 -27.040  < 2e-16 ***
## areaPEMI            -1.86152    0.15364 -12.116  < 2e-16 ***
## areaSOUTH GEORGIA   -1.44190    0.12260 -11.761  < 2e-16 ***
## mean_depth          -0.32889    0.05072  -6.484 8.91e-11 ***
## ---
## Signif. codes:  0 '***' 0.001 '**' 0.01 '*' 0.05 '.' 0.1 ' ' 1
## 
## (Dispersion parameter for binomial family taken to be 1)
## 
##     Null deviance: 6196.03  on 72  degrees of freedom
## Residual deviance:  299.88  on 65  degrees of freedom
## AIC: 611.64
## 
## Number of Fisher Scoring iterations: 16
```

Calculate pseudo r squared

```
pseudo_r2(glm_3.0)

## [1] 0.951601
```

Three terms models

Model with density of vessels

```
glm_3.1 <- glm(cbind(count,count_total - count)~ area+ mean_depth+ density, f
amily = binomial(link = logit), data = datsc)
summary(glm_3.1)

## 
## Call:
## glm(formula = cbind(count, count_total - count) ~ area + mean_depth + 
##     density, family = binomial(link = logit), data = datsc)
## 
## Deviance Residuals: 
##     Min       1Q   Median       3Q      Max  
## -4.2886  -1.3523  -0.0003   0.9517   6.2183  
## 
## Coefficients:
##                     Estimate Std. Error z value Pr(>|z|)    
## (Intercept)          0.64324    0.10103   6.367 1.93e-10 ***
## areaCROZET          -0.11967    0.15406  -0.777   0.4373    
## areaFALKLANDS       -3.63499    0.14409 -25.228  < 2e-16 ***
## areaHIMI           -22.49157 1034.19178  -0.022   0.9826    
## areaKERGUELEN       -4.85468    0.19437 -24.976  < 2e-16 ***
```

```
## areaPEMI            -1.85277    0.15405 -12.027  < 2e-16 ***
## areaSOUTH GEORGIA   -1.60579    0.13947 -11.513  < 2e-16 ***
## mean_depth          -0.29995    0.05184  -5.786 7.21e-09 ***
## density              0.12796    0.05099   2.510   0.0121 *
## ---
## Signif. codes:  0 '***' 0.001 '**' 0.01 '*' 0.05 '.' 0.1 ' ' 1
##
## (Dispersion parameter for binomial family taken to be 1)
##
##     Null deviance: 6196.03  on 72  degrees of freedom
## Residual deviance:  293.58  on 64  degrees of freedom
## AIC: 607.34
##
## Number of Fisher Scoring iterations: 16
```

Calculate pseudo r squared

```
pseudo_r2(glm_3.1)

## [1] 0.9526178
```

Model with total area size

```
glm_3.2 <- glm(cbind(count,count_total - count)~ area+ mean_depth+ total_area
, family = binomial(link = logit), data = datsc)
summary(glm_3.2)

##
## Call:
## glm(formula = cbind(count, count_total - count) ~ area + mean_depth +
##     total_area, family = binomial(link = logit), data = datsc)
##
## Deviance Residuals:
##     Min       1Q   Median       3Q      Max
## -4.5436  -1.2069  -0.0003   0.9779   5.9439
##
## Coefficients:
##                     Estimate Std. Error z value Pr(>|z|)
## (Intercept)          0.66733    0.10064   6.631 3.33e-11 ***
## areaCROZET          -0.20311    0.16380  -1.240    0.215
## areaFALKLANDS       -3.78216    0.14168 -26.695  < 2e-16 ***
## areaHIMI           -22.57032 1031.45980  -0.022    0.983
## areaKERGUELEN       -4.87740    0.22971 -21.233  < 2e-16 ***
## areaPEMI            -1.93420    0.17169 -11.266  < 2e-16 ***
## areaSOUTH GEORGIA   -1.46148    0.12422 -11.765  < 2e-16 ***
## mean_depth          -0.33482    0.05103  -6.561 5.34e-11 ***
## total_area          -0.07660    0.08051  -0.951    0.341
## ---
## Signif. codes:  0 '***' 0.001 '**' 0.01 '*' 0.05 '.' 0.1 ' ' 1
##
## (Dispersion parameter for binomial family taken to be 1)
```

```
##
##     Null deviance: 6196.03  on 72  degrees of freedom
## Residual deviance:  298.98  on 64  degrees of freedom
## AIC: 612.74
##
## Number of Fisher Scoring iterations: 16
```

Calculate pseudo r squared

```
pseudo_r2(glm_3.2)

## [1] 0.9517469
```

Model with proportion of days in winter

```
glm_3.3 <- glm(cbind(count,count_total - count)~ area+ mean_depth+ prop_day_w
inter, family = binomial(link = logit), data = datsc)
summary(glm_3.3)

##
## Call:
## glm(formula = cbind(count, count_total - count) ~ area + mean_depth +
##     prop_day_winter, family = binomial(link = logit), data = datsc)
##
## Deviance Residuals:
##     Min       1Q   Median       3Q      Max
## -4.4474  -1.1745  -0.0004   0.9607   6.1692
##
## Coefficients:
##                     Estimate Std. Error z value Pr(>|z|)
## (Intercept)          0.63402    0.11067   5.729 1.01e-08 ***
## areaCROZET          -0.13096    0.15591  -0.840    0.401
## areaFALKLANDS       -3.71930    0.14297 -26.015  < 2e-16 ***
## areaHIMI           -22.53410 1031.75816  -0.022    0.983
## areaKERGUELEN       -4.99407    0.18602 -26.847  < 2e-16 ***
## areaPEMI            -1.81915    0.16367 -11.115  < 2e-16 ***
## areaSOUTH GEORGIA   -1.34619    0.17647  -7.628 2.38e-14 ***
## mean_depth          -0.32960    0.05078  -6.491 8.52e-11 ***
## prop_day_winter     -0.03838    0.05091  -0.754    0.451
## ---
## Signif. codes:  0 '***' 0.001 '**' 0.01 '*' 0.05 '.' 0.1 ' ' 1
##
## (Dispersion parameter for binomial family taken to be 1)
##
##     Null deviance: 6196.03  on 72  degrees of freedom
## Residual deviance:  299.31  on 64  degrees of freedom
## AIC: 613.08
##
## Number of Fisher Scoring iterations: 16
```

Calculate pseudo r squared

```
pseudo_r2(glm_3.3)

## [1] 0.9516927
```

Model with proportion of sets using trotlines-cachalotera

```
glm_3.4 <- glm(cbind(count,count_total - count)~ area+ mean_depth+ prop_day_t
rotline_cach, family = binomial(link = logit), data = datsc)
summary(glm_3.4)

##
## Call:
## glm(formula = cbind(count, count_total - count) ~ area + mean_depth +
##     prop_day_trotline_cach, family = binomial(link = logit),
##     data = datsc)
##
## Deviance Residuals:
##     Min       1Q   Median       3Q      Max
## -4.5346  -1.2022  -0.0003   0.9817   6.2351
##
## Coefficients:
##                         Estimate Std. Error z value Pr(>|z|)
## (Intercept)             6.810e-01  1.737e-01   3.921 8.82e-05 ***
## areaCROZET             -1.663e-01  2.468e-01  -0.674    0.500
## areaFALKLANDS          -3.757e+00  1.638e-01 -22.940  < 2e-16 ***
## areaHIMI               -2.261e+01  1.032e+03  -0.022    0.983
## areaKERGUELEN          -5.024e+00  2.685e-01 -18.712  < 2e-16 ***
## areaPEMI               -1.870e+00  1.815e-01 -10.303  < 2e-16 ***
## areaSOUTH GEORGIA      -1.459e+00  2.320e-01  -6.290 3.17e-10 ***
## mean_depth             -3.284e-01  5.101e-02  -6.438 1.21e-10 ***
## prop_day_trotline_cach -7.875e-03  8.931e-02  -0.088    0.930
## ---
## Signif. codes:  0 '***' 0.001 '**' 0.01 '*' 0.05 '.' 0.1 ' ' 1
##
## (Dispersion parameter for binomial family taken to be 1)
##
##     Null deviance: 6196.03  on 72  degrees of freedom
## Residual deviance:  299.87  on 64  degrees of freedom
## AIC: 613.64
##
## Number of Fisher Scoring iterations: 16
```

Calculate pseudo r squared

```
pseudo_r2(glm_3.4)

## [1] 0.9516023
```

Model with mobility

```r
glm_3.5 <- glm(cbind(count,count_total - count)~ area+ mean_depth+mobility, f
amily = binomial(link = logit), data = datsc)
summary(glm_3.5)

##
## Call:
## glm(formula = cbind(count, count_total - count) ~ area + mean_depth +
##     mobility, family = binomial(link = logit), data = datsc)
##
## Deviance Residuals:
##     Min       1Q   Median       3Q      Max
## -4.6024  -1.2328  -0.0004   0.9382   6.4839
##
## Coefficients:
##                       Estimate Std. Error z value Pr(>|z|)
## (Intercept)            0.79644    0.11350   7.017 2.26e-12 ***
## areaCROZET            -0.16634    0.15450  -1.077   0.2816
## areaFALKLANDS         -3.74385    0.13691 -27.346  < 2e-16 ***
## areaHIMI             -22.74433 1013.94487  -0.022   0.9821
## areaKERGUELEN         -5.34749    0.23176 -23.074  < 2e-16 ***
## areaPEMI              -1.78931    0.15655 -11.430  < 2e-16 ***
## areaSOUTH GEORGIA     -1.69950    0.16173 -10.508  < 2e-16 ***
## mean_depth            -0.31578    0.05113  -6.176 6.58e-10 ***
## mobility               0.17675    0.07175   2.463   0.0138 *
## ---
## Signif. codes:  0 '***' 0.001 '**' 0.01 '*' 0.05 '.' 0.1 ' ' 1
##
## (Dispersion parameter for binomial family taken to be 1)
##
##     Null deviance: 6196.0  on 72  degrees of freedom
## Residual deviance:  293.8  on 64  degrees of freedom
## AIC: 607.57
##
## Number of Fisher Scoring iterations: 16
```

Calculate pseudo r squared

```r
pseudo_r2(glm_3.5)

## [1] 0.952582
```

AIC summary

```r
model.sel(list(glm_3.0, glm_3.1,glm_3.2,glm_3.3,glm_3.4,glm_3.5))

## Model selection table
##    (Int) are men_dpt   dns ttl_are prp_day_wnt prp_day_trt_cch    mbl df
## 2 0.6432   + -0.3000 0.128                                              9
## 6 0.7964   + -0.3158                                          0.1767  9
## 1 0.6685   + -0.3289                                                    8
## 3 0.6673   + -0.3348       -0.0766                                      9
```

```
## 4 0.6340   + -0.3296                  -0.03838                         9
## 5 0.6810   + -0.3284                               -0.007875         9
##     logLik  AICc delta weight
## 2 -294.672 610.2  0.00  0.450
## 6 -294.783 610.4  0.22  0.403
## 1 -297.822 613.9  3.69  0.071
## 3 -297.370 615.6  5.40  0.030
## 4 -297.538 615.9  5.73  0.026
## 5 -297.818 616.5  6.29  0.019
## Models ranked by AICc(x)
```

Model with lowest AIC

```
glm_4.0 <- glm(cbind(count,count_total - count)~ area+ mean_depth+density, fa
mily = binomial(link = logit), data = datsc)
summary(glm_4.0)
```

```
##
## Call:
## glm(formula = cbind(count, count_total - count) ~ area + mean_depth +
##     density, family = binomial(link = logit), data = datsc)
##
## Deviance Residuals:
##     Min       1Q   Median       3Q      Max
## -4.2886  -1.3523  -0.0003   0.9517   6.2183
##
## Coefficients:
##                      Estimate Std. Error z value Pr(>|z|)
## (Intercept)           0.64324    0.10103   6.367 1.93e-10 ***
## areaCROZET           -0.11967    0.15406  -0.777   0.4373
## areaFALKLANDS        -3.63499    0.14409 -25.228  < 2e-16 ***
## areaHIMI            -22.49157 1034.19178  -0.022   0.9826
## areaKERGUELEN        -4.85468    0.19437 -24.976  < 2e-16 ***
## areaPEMI             -1.85277    0.15405 -12.027  < 2e-16 ***
## areaSOUTH GEORGIA    -1.60579    0.13947 -11.513  < 2e-16 ***
## mean_depth           -0.29995    0.05184  -5.786 7.21e-09 ***
## density               0.12796    0.05099   2.510   0.0121 *
## ---
## Signif. codes:  0 '***' 0.001 '**' 0.01 '*' 0.05 '.' 0.1 ' ' 1
##
## (Dispersion parameter for binomial family taken to be 1)
##
##     Null deviance: 6196.03  on 72  degrees of freedom
## Residual deviance:  293.58  on 64  degrees of freedom
## AIC: 607.34
##
## Number of Fisher Scoring iterations: 16
```

Calculate pseudo r squared

```
pseudo_r2(glm_4.0)
```

```
## [1] 0.9526178
```

Four terms models

Model with proportion of days in winter

```
glm_4.1 <- glm(cbind(count,count_total - count)~ area+ mean_depth+density+ pr
op_day_winter, family = binomial(link = logit), data = datsc)
summary(glm_4.1)
```

```
##
## Call:
## glm(formula = cbind(count, count_total - count) ~ area + mean_depth +
##     density + prop_day_winter, family = binomial(link = logit),
##     data = datsc)
##
## Deviance Residuals:
##     Min       1Q   Median       3Q      Max
## -4.3964  -1.3407  -0.0003   0.9257   6.1071
##
## Coefficients:
##                      Estimate Std. Error z value Pr(>|z|)
## (Intercept)           0.60142    0.11118   5.409 6.33e-08 ***
## areaCROZET           -0.09710    0.15618  -0.622   0.5341
## areaFALKLANDS        -3.59651    0.15019 -23.947  < 2e-16 ***
## areaHIMI            -22.41711 1034.00053  -0.022   0.9827
## areaKERGUELEN        -4.83586    0.19551 -24.734  < 2e-16 ***
## areaPEMI             -1.80328    0.16364 -11.020  < 2e-16 ***
## areaSOUTH GEORGIA    -1.49453    0.18606  -8.033 9.54e-16 ***
## mean_depth           -0.30016    0.05190  -5.784 7.30e-09 ***
## density               0.13073    0.05110   2.558   0.0105 *
## prop_day_winter      -0.04595    0.05085  -0.904   0.3662
## ---
## Signif. codes:  0 '***' 0.001 '**' 0.01 '*' 0.05 '.' 0.1 ' ' 1
##
## (Dispersion parameter for binomial family taken to be 1)
##
##     Null deviance: 6196.03  on 72  degrees of freedom
## Residual deviance:  292.77  on 63  degrees of freedom
## AIC: 608.53
##
## Number of Fisher Scoring iterations: 16
```

Calculate pseudo r squared

```
pseudo_r2(glm_4.1)
```

```
## [1] 0.9527495
```

Model with mobility

```r
glm_4.2 <- glm(cbind(count,count_total - count)~ area+ mean_depth+density+mob
ility, family = binomial(link = logit), data = datsc)
summary(glm_4.2)
```

```
##
## Call:
## glm(formula = cbind(count, count_total - count) ~ area + mean_depth +
##     density + mobility, family = binomial(link = logit), data = datsc)
##
## Deviance Residuals:
##     Min       1Q   Median       3Q      Max
## -4.4105  -1.2843  -0.0004   0.9298   6.3777
##
## Coefficients:
##                      Estimate Std. Error z value Pr(>|z|)
## (Intercept)           0.73538    0.12035   6.110 9.95e-10 ***
## areaCROZET           -0.14011    0.15511  -0.903    0.366
## areaFALKLANDS        -3.66534    0.14615 -25.080  < 2e-16 ***
## areaHIMI            -22.61566 1019.85528  -0.022    0.982
## areaKERGUELEN        -5.12510    0.27368 -18.726  < 2e-16 ***
## areaPEMI             -1.80780    0.15724 -11.497  < 2e-16 ***
## areaSOUTH GEORGIA   -1.72423    0.16288 -10.586  < 2e-16 ***
## mean_depth           -0.30042    0.05198  -5.779 7.50e-09 ***
## density               0.08794    0.05865   1.499    0.134
## mobility              0.11622    0.08209   1.416    0.157
## ---
## Signif. codes:  0 '***' 0.001 '**' 0.01 '*' 0.05 '.' 0.1 ' ' 1
##
## (Dispersion parameter for binomial family taken to be 1)
##
##     Null deviance: 6196.03  on 72  degrees of freedom
## Residual deviance:  291.57  on 63  degrees of freedom
## AIC: 607.33
##
## Number of Fisher Scoring iterations: 16
```

Calculate pseudo r squared

```r
pseudo_r2(glm_4.2)
```

```
## [1] 0.9529426
```

Model with proportion of sets using trotlines-cachalotera

```r
glm_4.3 <- glm(cbind(count,count_total - count)~ area+ mean_depth+density+pro
p_day_trotline_cach, family = binomial(link = logit), data = datsc)
summary(glm_4.3)
```

```
##
## Call:
## glm(formula = cbind(count, count_total - count) ~ area + mean_depth +
```

```
##     density + prop_day_trotline_cach, family = binomial(link = logit),
##     data = datsc)
##
## Deviance Residuals:
##     Min       1Q   Median       3Q      Max
## -4.2871  -1.3526  -0.0003   0.9510   6.1523
##
## Coefficients:
##                            Estimate Std. Error z value Pr(>|z|)
## (Intercept)                 0.67477    0.17411   3.876 0.000106 ***
## areaCROZET                 -0.16269    0.24733  -0.658 0.510673
## areaFALKLANDS              -3.65394    0.16801 -21.749  < 2e-16 ***
## areaHIMI                  -22.53528 1034.12411  -0.022 0.982614
## areaKERGUELEN              -4.89739    0.27324 -17.924  < 2e-16 ***
## areaPEMI                   -1.87434    0.18204 -10.296  < 2e-16 ***
## areaSOUTH GEORGIA          -1.65069    0.24549  -6.724 1.77e-11 ***
## mean_depth                 -0.29862    0.05216  -5.725 1.03e-08 ***
## density                     0.12863    0.05111   2.517 0.011836 *
## prop_day_trotline_cach     -0.01992    0.08962  -0.222 0.824057
## ---
## Signif. codes:  0 '***' 0.001 '**' 0.01 '*' 0.05 '.' 0.1 ' ' 1
##
## (Dispersion parameter for binomial family taken to be 1)
##
##     Null deviance: 6196.03  on 72  degrees of freedom
## Residual deviance:  293.53  on 63  degrees of freedom
## AIC: 609.29
##
## Number of Fisher Scoring iterations: 16
```

Calculate pseudo r squared

```
pseudo_r2(glm_4.3)

## [1] 0.9526258
```

Model with total area size

```
glm_4.4 <- glm(cbind(count,count_total - count)~ area+ mean_depth+density+tot
al_area, family = binomial(link = logit), data = datsc)
summary(glm_4.4)

##
## Call:
## glm(formula = cbind(count, count_total - count) ~ area + mean_depth +
##     density + total_area, family = binomial(link = logit), data = datsc)
##
## Deviance Residuals:
##     Min       1Q   Median       3Q      Max
## -4.3592  -1.3578  -0.0003   0.9508   6.1045
##
```

```
## Coefficients:
##                     Estimate Std. Error z value Pr(>|z|)
## (Intercept)          0.64380    0.10102   6.373 1.86e-10 ***
## areaCROZET          -0.14043    0.16604  -0.846    0.398
## areaFALKLANDS       -3.65119    0.15206 -24.012  < 2e-16 ***
## areaHIMI           -22.48233 1031.78211  -0.022    0.983
## areaKERGUELEN       -4.81279    0.23119 -20.818  < 2e-16 ***
## areaPEMI            -1.87964    0.17377 -10.817  < 2e-16 ***
## areaSOUTH GEORGIA   -1.60728    0.13950 -11.522  < 2e-16 ***
## mean_depth          -0.30316    0.05270  -5.753 8.78e-09 ***
## density              0.12354    0.05266   2.346    0.019 *
## total_area          -0.02780    0.08312  -0.334    0.738
## ---
## Signif. codes:  0 '***' 0.001 '**' 0.01 '*' 0.05 '.' 0.1 ' ' 1
##
## (Dispersion parameter for binomial family taken to be 1)
##
##     Null deviance: 6196.03  on 72  degrees of freedom
## Residual deviance:  293.47  on 63  degrees of freedom
## AIC: 609.23
##
## Number of Fisher Scoring iterations: 16
```

Calculate pseudo r squared

```
pseudo_r2(glm_4.4)

## [1] 0.9526359
```

AIC summary

```
model.sel(list(glm_4.0,glm_4.1,glm_4.2,glm_4.3,glm_4.4))

## Model selection table
##    (Int) are     dns men_dpt prp_day_wnt    mbl prp_day_trt_cch ttl_are df
## 1 0.6432   + 0.12800 -0.3000                                              9
## 3 0.7354   + 0.08794 -0.3004             0.1162                          10
## 2 0.6014   + 0.13070 -0.3002    -0.04595                                 10
## 5 0.6438   + 0.12350 -0.3032                                  -0.0278 10
## 4 0.6748   + 0.12860 -0.2986                            -0.01992         10
##     logLik  AICc delta weight
## 1 -294.672 610.2  0.00  0.378
## 3 -293.666 610.9  0.68  0.269
## 2 -294.264 612.1  1.88  0.148
## 5 -294.616 612.8  2.58  0.104
## 4 -294.647 612.8  2.64  0.101
## Models ranked by AICc(x)
```

FINAL MODEL

```
glm_5.0 <- glm(cbind(count,count_total - count)~ area+ mean_depth+density, fa
mily = binomial(link = logit), data = datsc, na.action = "na.fail")
summary(glm_5.0)
```

```
##
## Call:
## glm(formula = cbind(count, count_total - count) ~ area + mean_depth +
##     density, family = binomial(link = logit), data = datsc, na.action = "n
a.fail")
##
## Deviance Residuals:
##     Min       1Q   Median       3Q      Max
## -4.2886  -1.3523  -0.0003   0.9517   6.2183
##
## Coefficients:
##                       Estimate Std. Error z value Pr(>|z|)
## (Intercept)            0.64324    0.10103   6.367 1.93e-10 ***
## areaCROZET            -0.11967    0.15406  -0.777   0.4373
## areaFALKLANDS         -3.63499    0.14409 -25.228  < 2e-16 ***
## areaHIMI             -22.49157 1034.19178  -0.022   0.9826
## areaKERGUELEN         -4.85468    0.19437 -24.976  < 2e-16 ***
## areaPEMI              -1.85277    0.15405 -12.027  < 2e-16 ***
## areaSOUTH GEORGIA     -1.60579    0.13947 -11.513  < 2e-16 ***
## mean_depth            -0.29995    0.05184  -5.786 7.21e-09 ***
## density                0.12796    0.05099   2.510   0.0121 *
## ---
## Signif. codes:  0 '***' 0.001 '**' 0.01 '*' 0.05 '.' 0.1 ' ' 1
##
## (Dispersion parameter for binomial family taken to be 1)
##
##     Null deviance: 6196.03  on 72  degrees of freedom
## Residual deviance:  293.58  on 64  degrees of freedom
## AIC: 607.34
##
## Number of Fisher Scoring iterations: 16
```

Calculate pseudo r squared

```
pseudo_r2(glm_5.0)
```

```
## [1] 0.9526178
```

Model validation

plots of residuals versus fitted values for check of linearity and homoscedasticity of variance

```
plot(fitted(glm_5.0), resid(glm_5.0), main="Residual Plot for linearity and h
omoscedasticity of variance",xlab="Fitted value",ylab="Residuals")
```

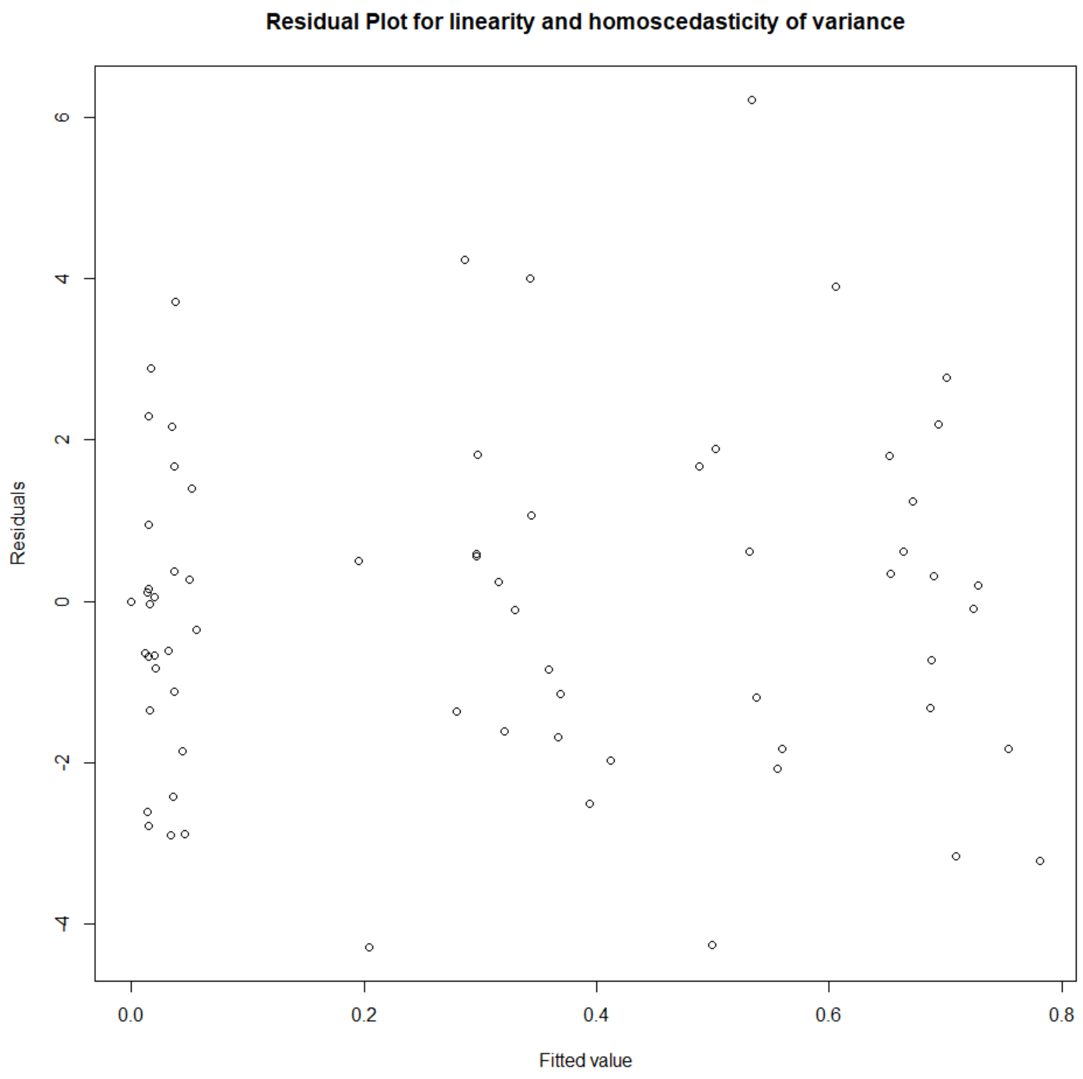

test for normality of the residuals

```
hist(resid(glm_5.0),xlab="Residuals",main="Normality of the Residuals")
```

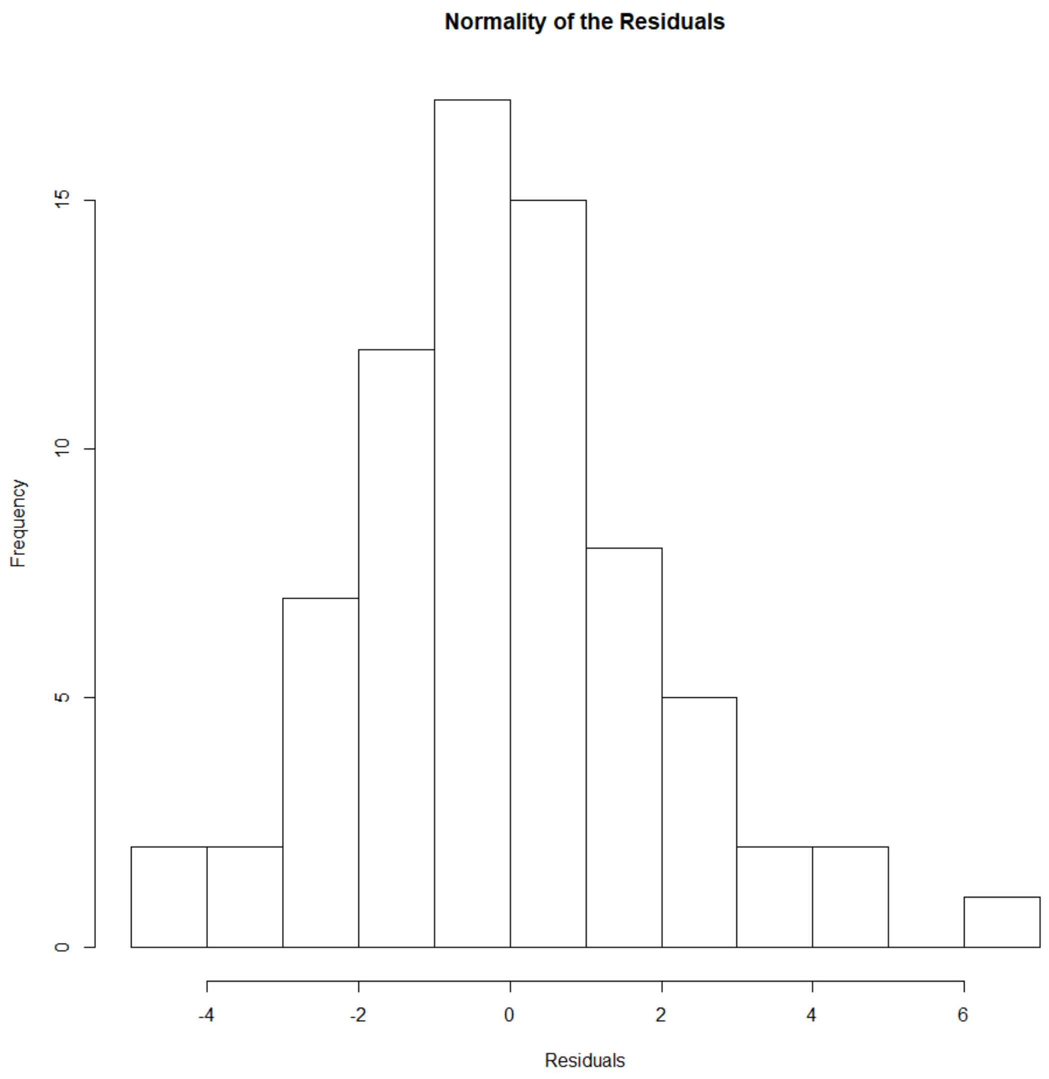

Interaction between Area and mobility

Since mobility isn't selected in the final model it isn't appropriate to consider an interaction between it and fishery (Area)

FINAL AIC SELECTION

Create the AIC selection table, we also calculate all of the pseudo r squared statistics using the extra argument of `model.sel`.

```
(fleet_summary <- model.sel(list(glm_null, glm_1.1,glm_1.2,glm_1.3,glm_1.4,glm_1.5,glm_1.6,glm_1.7,glm_2.0,glm_2.1,glm_2.2,glm_2.3,glm_2.4,glm_2.5,glm_2.6,glm_3.0,glm_3.1,glm_3.2,glm_3.3,glm_3.4,glm_3.5,glm_4.0,glm_4.1,glm_4.2,glm_4.3,glm_4.4, glm_5.0), rank=AIC, extra=pseudo_r2))
```

```
## Model selection table
##       (Int)   ttl_are men_dpt     mbl prp_day_trt_cch prp_day_wnt     dns
## 24  0.73540           -0.3004  0.1162                             0.08794
## 17  0.64320           -0.3000                                     0.12800
## 22  0.64320           -0.3000                                     0.12800
## 27  0.64320           -0.3000                                     0.12800
## 21  0.79640           -0.3158  0.1767
## 23  0.60140           -0.3002                            -0.04595 0.13070
## 26  0.64380 -0.027800 -0.3032                                     0.12350
## 25  0.67480           -0.2986               -0.019920             0.12860
## 11  0.66850           -0.3289
## 16  0.66850           -0.3289
## 18  0.66730 -0.076600 -0.3348
## 19  0.63400           -0.3296                            -0.03838
## 20  0.68100           -0.3284               -0.007875
## 15  0.11050                                                       0.19140
## 12  0.26460                     0.2266
## 8   0.06979
## 9   0.06979
## 13  0.17790                                 -0.063770
## 14  0.03776                                              -0.03441
## 10  0.06863 -0.007301
## 2  -1.14200 -1.122000
## 7  -0.99220                                                       0.79640
## 4  -1.21000                    -0.7585
## 5  -1.13800                                  0.302100
## 6  -1.15100                                              -0.23310
## 3  -1.12500             0.1457
## 1  -1.10800
##    are pseudo_r2 na.action df    logLik    AIC   delta weight
## 24   +   0.95290           10  -293.666  607.3    0.00  0.152
## 17   +   0.95260            9  -294.672  607.3    0.01  0.151
## 22   +   0.95260            9  -294.672  607.3    0.01  0.151
## 27   +   0.95260   na.fail  9  -294.672  607.3    0.01  0.151
## 21   +   0.95260            9  -294.783  607.6    0.23  0.135
## 23   +   0.95270           10  -294.264  608.5    1.20  0.084
## 26   +   0.95260           10  -294.616  609.2    1.90  0.059
## 25   +   0.95260           10  -294.647  609.3    1.96  0.057
## 11   +   0.95160            8  -297.822  611.6    4.31  0.018
## 16   +   0.95160            8  -297.822  611.6    4.31  0.018
## 18   +   0.95170            9  -297.370  612.7    5.41  0.010
## 19   +   0.95170            9  -297.538  613.1    5.74  0.009
## 20   +   0.95160            9  -297.818  613.6    6.30  0.006
## 15   +   0.94720            8  -311.594  639.2   31.86  0.000
## 12   +   0.94640            8  -314.072  644.1   36.81  0.000
## 8    +   0.94470            7  -319.109  652.2   44.89  0.000
## 9    +   0.94470            7  -319.109  652.2   44.89  0.000
## 13   +   0.94480            8  -318.849  653.7   46.37  0.000
## 14   +   0.94480            8  -318.882  653.8   46.43  0.000
## 10   +   0.94470            8  -319.105  654.2   46.88  0.000
```

```
## 2       0.32560          2 -2237.042 4478.1 3870.75  0.000
## 7       0.21570          2 -2577.603 5159.2 4551.87  0.000
## 4       0.19600          2 -2638.608 5281.2 4673.88  0.000
## 5       0.04307          2 -3112.464 6228.9 5621.60  0.000
## 6       0.01625          2 -3195.552 6395.1 5787.77  0.000
## 3       0.01015          2 -3214.442 6432.9 5825.55  0.000
## 1       0.00000          1 -3245.896 6493.8 5886.46  0.000
## Models ranked by AIC(x)
```

Save a copy to file

```
write.csv(fleet_summary, "Killer_whale_fleet_data_AIC_summary.csv")
```

Coefficients of final model

Extract the coefficients of the final model and convert from logit space to natural numbers (i.e. probabilities) and the upper and lower confidence intervals

```
flt_est <- plogis(summary(glm_5.0)$coefficients[,1]+summary(glm_5.0)$coeffici
ents[1,1])
flt_lwr <- plogis((summary(glm_5.0)$coefficients[,1]+summary(glm_5.0)$coeffic
ients[1,1]-1.96*summary(glm_5.0)$coefficients[,2]))
flt_upr <- plogis((summary(glm_5.0)$coefficients[,1]+summary(glm_5.0)$coeffic
ients[1,1]+1.96*summary(glm_5.0)$coefficients[,2]))
flt_z_stat <- summary(glm_5.0)$coefficients[,3]
flt_p_value <- summary(glm_5.0)$coefficients[,4]
```

Bind them together

```
(res_fleet <- cbind(flt_est, flt_lwr, flt_upr, flt_z_stat, flt_p_value))

##                        flt_est    flt_lwr    flt_upr   flt_z_stat
## (Intercept)       7.835517e-01 0.74809131 0.81525319   6.36658411
## areaCROZET        6.279833e-01 0.55517820 0.69540987  -0.77675624
## areaFALKLANDS     4.780024e-02 0.03646866 0.06242465 -25.22800516
## areaHIMI          3.246338e-10 0.00000000 1.00000000  -0.02174796
## areaKERGUELEN     1.460843e-02 0.01002688 0.02123850 -24.97632217
## areaPEMI          2.297848e-01 0.18072061 0.28749524 -12.02673278
## areaSOUTH GEORGIA 2.763688e-01 0.22514906 0.33421487 -11.51344464
## mean_depth        5.849901e-01 0.56012890 0.60942588  -5.78603395
## density           6.837807e-01 0.66178745 0.70498130   2.50971081
##                     flt_p_value
## (Intercept)        1.932843e-10
## areaCROZET         4.373026e-01
## areaFALKLANDS     1.974825e-140
## areaHIMI           9.826490e-01
## areaKERGUELEN     1.105731e-137
## areaPEMI           2.571363e-33
## areaSOUTH GEORGIA  1.128778e-30
## mean_depth         7.206766e-09
## density            1.208301e-02
```

Create the estimates for Chile (which is zero in binomial regression model)

```
(areaChile <- c(plogis(0+summary(glm_5.0)$coefficients[1,1]),
               plogis(0+summary(glm_5.0)$coefficients[1,1]-1.96*summary(glm_5
.0)$coefficients[1,2]),
               plogis(0+summary(glm_5.0)$coefficients[1,1]+1.96*summary(glm_5
.0)$coefficients[1,2]),
               summary(glm_5.0)$coefficients[1,3], summary(glm_5.0)$coefficie
nts[1,4]))

## [1] 6.554861e-01 6.095011e-01 6.987326e-01 6.366584e+00 1.932843e-10
```

Replace the intercept with Chilean values

```
res_fleet[1,] <- areaChile
```

Save as a csv

```
write.csv(res_fleet, "Killer_whale_final_model_fleet_est.csv")
```

Session information

Print the session information

```
sessionInfo()

## R version 3.4.1 (2017-06-30)
## Platform: x86_64-w64-mingw32/x64 (64-bit)
## Running under: Windows 10 x64 (build 17134)
##
## Matrix products: default
##
## locale:
## [1] LC_COLLATE=English_Australia.1252  LC_CTYPE=English_Australia.1252
## [3] LC_MONETARY=English_Australia.1252 LC_NUMERIC=C
## [5] LC_TIME=English_Australia.1252
##
## attached base packages:
## [1] stats     graphics  grDevices utils     datasets  methods   base
##
## other attached packages:
##  [1] Rmisc_1.5       plyr_1.8.4      lattice_0.20-35 ggpubr_0.1.6
##  [5] magrittr_1.5    ggplot2_2.2.1   MuMIn_1.15.6    merTools_0.3.0
##  [9] dplyr_0.7.2     arm_1.9-3       MASS_7.3-47     lmerTest_2.0-33
## [13] lme4_1.1-15     Matrix_1.2-10
##
## loaded via a namespace (and not attached):
##  [1] Rcpp_0.12.12       mvtnorm_1.0-6      tidyr_0.7.0
##  [4] assertthat_0.2.0   rprojroot_1.2      digest_0.6.12
##  [7] psych_1.7.5        mime_0.5           R6_2.2.2
## [10] backports_1.1.0    acepack_1.4.1      stats4_3.4.1
## [13] evaluate_0.10.1    coda_0.19-1        rlang_0.1.2
```

```
## [16] lazyeval_0.2.0      minqa_1.2.4         data.table_1.10.4
## [19] nloptr_1.0.4        rpart_4.1-11        DT_0.2
## [22] checkmate_1.8.3     rmarkdown_1.6       labeling_0.3
## [25] splines_3.4.1       stringr_1.2.0       foreign_0.8-69
## [28] htmlwidgets_0.9     munsell_0.4.3       shiny_1.0.5
## [31] broom_0.4.2         compiler_3.4.1      httpuv_1.3.5
## [34] pkgconfig_2.0.1     base64enc_0.1-3     mnormt_1.5-5
## [37] htmltools_0.3.6     nnet_7.3-12         tibble_1.3.4
## [40] gridExtra_2.2.1     htmlTable_1.9       Hmisc_4.0-3
## [43] grid_3.4.1          xtable_1.8-2        nlme_3.1-131
## [46] gtable_0.2.0        scales_0.5.0        stringi_1.1.5
## [49] reshape2_1.4.3      bindrcpp_0.2        latticeExtra_0.6-28
## [52] cowplot_0.8.0       Formula_1.2-2       blme_1.0-4
## [55] RColorBrewer_1.1-2  tools_3.4.1         glue_1.1.1
## [58] purrr_0.2.3         abind_1.4-5         parallel_3.4.1
## [61] survival_2.41-3     yaml_2.1.14         colorspace_1.3-2
## [64] cluster_2.0.6       knitr_1.17          bindr_0.1
```
